# Unraveling the Potential of Small Molecule Heparin Glycomimetics in Neuroregenerative Therapeutics

**DOI:** 10.1021/jacs.5c13142

**Published:** 2025-12-08

**Authors:** Melis Özkan, Giada Cellot, Sujeet Pawar, Deepika Sardana, Ivana Barravecchia, Laura Ballerini, Debora Angeloni, Silvestro Micera, Francesco Stellacci

**Affiliations:** † Institute of Materials, 27218 École Polytechnique Fédérale de Lausanne (EPFL), Lausanne 1015, Switzerland; ‡ Bertarelli Foundation Chair in Translational Neural Engineering, Center for Neuroprosthetics, 19005 École Polytechnique Fédérale de Lausanne (EPFL), Lausanne 1015, Switzerland; § 19040 International School for Advanced Studies (SISSA), Neuroscience Area, Via Bonomea 265, Trieste 34136, Italy; ∥ The Institute of Biorobotics, Scuola Superiore Sant’Anna, Piazza Martiri della Libertà 33, Pisa 56127, Italy; ⊥ Health Science Interdisciplinary Center, Scuola Superiore Sant’Anna, Via G. Moruzzi, 1, Pisa 56124, Italy; ▼ Department of Excellence in Robotics & AI, Scuola Superiore Sant’Anna, Piazza Martiri della Libertà 33, Pisa 56127, Italy; ∇ Institute of Bioengineering, École Polytechnique Fédérale de Lausanne (EPFL), Lausanne 1015, Switzerland; ○ Global Health Institute, École Polytechnique Fédérale de Lausanne (EPFL), Lausanne 1015, Switzerland; 9 University of Trieste, Department of Life Sciences, Via Giorgieri 5, 34127 Trieste, Italy

## Abstract

Heparin and heparan sulfate (HS) glycosaminoglycans (GAGs) are essential regulators of neurotrophic signaling. However, their therapeutic applications are hindered by structural heterogeneity, batch variability, and anticoagulant activity. Thus, there is a need for well-defined glycomimetics that replicate the function of native HS in regenerative medicine. Here, we synthesized HS glycomimetics through a modular strategy that enables the installation of sulfate groups at the designated positions along the sugar backbone. These glycomimetics selectively bind and stabilize neurotrophins, such as fibroblast growth factors (FGF-1, FGF-2) and nerve growth factor (NGF), in a sulfation-dependent manner with dissociation constants in the low micromolar range. They exhibit no anticoagulant activity, a crucial prerequisite for clinical translation. We show that our lead compound has neuritogenic ability because in two neuronal cell models, PC12 and SH-SY5Y, it enhances NGF-mediated neural maturation when immobilized on a surface. Furthermore, in primary rat hippocampal neurons, it promotes FGF-2-mediated neurite outgrowth and spontaneous synaptic activity. Our findings show that HS glycomimetics have the potential for regenerative therapies.

## Graphical abstract

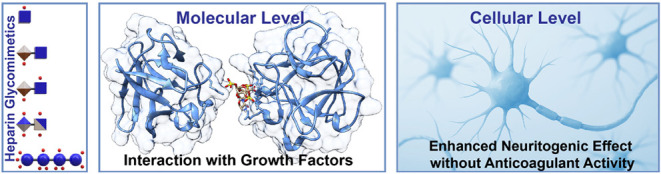

## 1 Introduction

Heparin/heparan sulfate (HS) glycosaminoglycans (GAGs) represent a prominent class of biomolecules capable of encoding functional information, analogous to nucleic acids and proteins. The structural and biological diversity of HS GAGs arises from variations in chain length, net charge, nontemplated sulfation patterns, and epimerization states, all of which are tightly regulated at both the cellular and systemic levels.^1^ The unique sulfation profiles of HS account for their interactions with various endogenous proteins. These interactions induce conformational changes within proteins, modulate their activity, and stabilize receptor–ligand complexes, therefore enabling cellular signaling.^2^ Additionally, HS sequesters proteins to regulate their spatial and temporal availability and therefore prolongs their functional lifespan within the extracellular matrix (ECM).^3^

Heparin and heparan sulfate are closely related GAGs composed of repeating disaccharide units of uronic acid and d-glucosamine, yet they differ profoundly in their fine structure and therefore biological role. Their chains consist of alternating uronic acid-(1→4)-d-glucosamine units, in which the uronic acid may occur as β-d-glucuronic acid (GlcA) or its C5 epimer, α-l-iduronic acid (IdoA). The GlcA residues may undergo enzymatic epimerization to IdoA during biosynthesis, introducing conformational flexibility that modulates protein recognition and binding. The glucosamine residues can be *N*-sulfated (GlcNS) or *N*-acetylated (GlcNAc), with additional *O*-sulfation occurring at the 2-, 3-, and 6-positions.^4^ Heparin, synthesized and stored in mast cell granules, is a highly sulfated polysaccharide enriched in IdoA2S-GlcNS6S repeating units, which can constitute up to 70–80% of its disaccharides.^5^ In contrast, heparan is a less sulfated polysaccharide ubiquitously expressed on cell surfaces and within the ECM as part of proteoglycans.^6^ It contains a higher proportion of GlcA and GlcNAc residues, resulting in elevated GlcA/IdoA and GlcNAc/GlcNS ratios and a lower overall sulfate content (typically 0.2–0.7 *O*-sulfates per disaccharide compared to ∼2.4 in heparin).^7^ The functional importance of sulfation motifs in HS GAGs is well-established.^8^ Defined sulfation sequences have been shown to regulate the activity of growth factors (GFs), such as fibroblast GFs (FGFs),^9,10^ and chemokines^11^ by dictating binding affinity and downstream signaling. Notably, the anticoagulant activity of heparin is also a direct result of specific sulfation patterns that enhance binding to antithrombin III (AT-III), thereby accelerating the inactivation of coagulation factors IIa and Xa.^12,13^ However, these features that endow HS with its biological potency also create challenges.

Despite its biological relevance, the therapeutic use of HS is constrained by its structural heterogeneity, nontemplated biosynthesis, and batch-to-batch variability. These issues complicate the elucidation of structure–activity relationships (SAR) and the physiological effects of carbohydrate–protein interactions. Furthermore, they raise safety concerns in clinical settings,^14^ as exemplified by the 2007–2008 heparin contamination crisis, which led to severe adverse reactions and fatalities worldwide.^15,16^ These shortcomings of native HS emphasize the rising demand for chemically defined HS glycomimetics. Structural studies suggest that specific subsets of HS subunits could be sufficient to exert relevant effects, as evidenced by the FDA’s endorsement of the Fondaparinux drug, a synthetic HS pentasaccharide administered as an anticoagulant. Recent synthetic advances, encompassing automated assembly,^17^ chemoenzymatic techniques^18,19^ and elegant synthetic routes^20–23^ have expanded access to well-defined HS GAG libraries with precise structural and functional attributes.^24^

A promising, yet underexplored direction in this field involves designing HS glycomimetics that selectively preserve GF-binding while eliminating anticoagulant motifs. Such molecules could decouple therapeutic effects from adverse ones, a key design criterion for regenerative medicine, where stimulating GF signaling must not compromise hemostatic balance.^25^

This need is particularly urgent in neural tissue regeneration, where the intrinsic limits of nervous system repair hamper recovery.^26^ In this context, HS is a central modulator of the ECM cues that control neural growth, survival, and plasticity.^27^ Injury, however, interferes with these signaling processes, and hence, the recovery remains suboptimal.^28^ Conventional approaches, such as exogenous GF therapies, have been implemented to accelerate nerve repair, given the vital functions of GFs in promoting cell survival and differentiation.^29^ However, their efficacy suffers from rapid degradation, low bioavailability, and inadequate retention at injury sites.^30,31^ To address these issues, biomaterial-based approaches incorporating synthetic glycomimetics have attracted interest for their ability to bind to GFs and improve their bioactivity with spatiotemporal control. Notably, glycopolymers functionalized with HS-mimicking disaccharides have been shown to bind to FGF-2 and also augment neural specification in embryonic stem cells, indicating the importance of synthetic sugars in developmental processes.^32,33^ On the other hand, although these polymers interact strongly with proteins through multivalency, their heavy negative charge necessitates delicate optimization to maintain selective therapeutic action while preventing anticoagulant activity.^34^

In parallel, previous studies on HS disaccharides^35,36^ and tetrasaccharides^37^ have presented key aspects of various FGF and chemokine binding characteristics. While these libraries of small-molecule sugars enabled the systematic investigation of their interactions with proteins and, therefore, the identification of candidates with high specificity, their application has remained largely confined to binding assays, with limited exploration of how such interactions affect biological activity in relevant cellular systems. As a result, a critical next step is to develop chemically defined HS glycomimetics that not only exhibit targeted neurotrophin binding but also translate these interactions into functional outcomes in neural models without triggering anticoagulant effects. This capability is essential for unlocking the therapeutic potential of glycomimetics in regenerative medicine.

In this study, we synthesized five small-molecule HS glycomimetics featuring distinct sulfation patterns. Within this library, we identified a promising candidate possessing a strong to moderate binding affinity for neurotrophins: FGF-1 (*K*_D_ = 0.78 μM), FGF-2 (*K*_D_ = 0.67 μM), and NGF (*K*_D_ = 2.6 μM and 468 μM, two-site binding model), while showing no detectable interaction with AT-III. With circular dichroism (CD) spectroscopy and thermal denaturation studies, we determined that our HS glycomimetics increased the melting temperatures of FGF-1 and FGF-2. We also performed Factor Xa and Factor IIa activity assays and aPTT measurements, revealing that they do not disrupt the coagulation pathways. Our findings are supported by molecular modeling studies. We evaluated cellular responses by employing two established neuronal cell models, PC12 and SH-SY5Y cells, that represent complementary stages of neuronal differentiation and maturation. Here, we show that our lead compound, when immobilized on a surface, enhances NGF-mediated neural maturation in both cell lines, comparable to its native HS counterpart. We also show that it has neuritogenic activity in rat hippocampal neurons when present in cell media together with FGF-2. These results establish a link between molecular design and regenerative function, showing that synthetic HS glycomimetics can selectively engage neurotrophic pathways while avoiding anticoagulant activity. This work advances the therapeutic potential of structurally precise HS glycomimetics in neural tissue regeneration.

## 2 Results and Discussion

### 2.1 Design and Chemical Synthesis of Small Molecule HS Glycomimetics

The chemical structures of native HS GAGs and synthetic HS glycomimetics developed in this study are shown in Figure 1. Our design strategy was guided by structural biology insights, particularly X-ray cocrystal structures of HS-derived oligosaccharides in complex with GFs.^38^ These studies have established that sulfation positions, uronic acid composition, and oligosaccharide length collectively govern HS-protein interactions.

**Figure 1:**
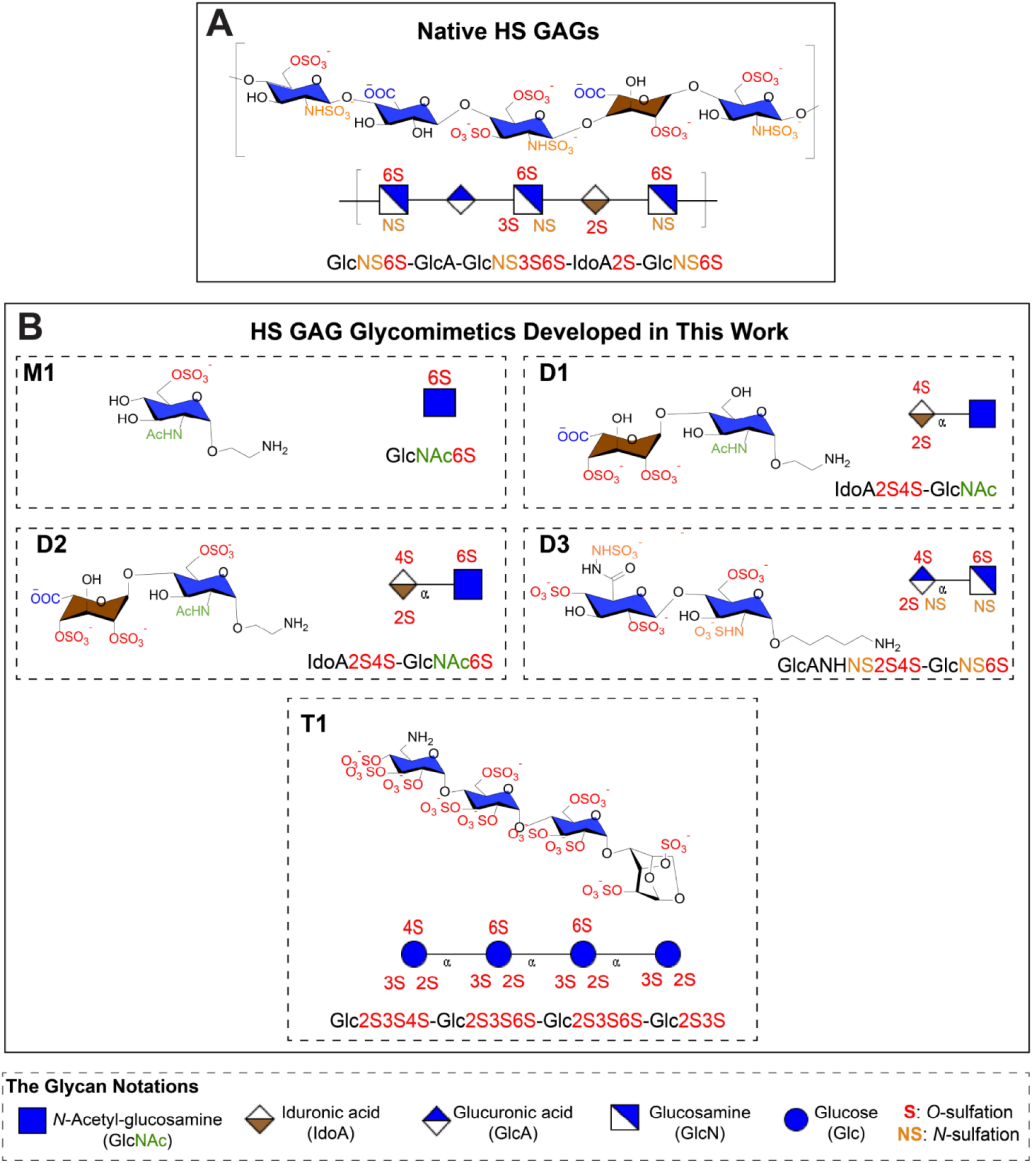
Chemical structures of (A) native HS GAGs and (B) the HS glycomimetics developed in this study.

Leveraging these principles, we synthesized a five-molecule library of mono- (**M1**), di- (**D1**, **D2**, **D3**), and tetrasaccharide (**T1**) sugars to dissect the contributions of individual sulfation motifs systematically. Among these, sulfation at the 6-*O*-position in GlcNAc has been characterized as a critical determinant for ionic interactions with proteins such as FGFs, promoting the formation of stable HS-FGF-receptor complexes within the ECM.^39,40^ To directly examine the role of this modification, we prepared a monosaccharide analog, **M1** (GlcNAc6S), bearing this key sulfation feature as a starting point.

Building upon this minimal unit, we next incorporated an IdoA moiety, which introduces the conformational flexibility necessary for accommodating GF-binding interfaces. The 2-*O*-sulfation of IdoA strengthens electrostatic complementarity and favors the ^2^S_0_ puckered conformation of the iduronate ring,^41^ a hallmark of biologically active HS-protein complexes.^42^ Recently, it was discovered that the 4-*O*-sulfated IdoA disaccharide modulates endothelial cell proliferation, migration, and angiogenesis, highlighting the importance of sulfation patterns in regulating cellular behaviors.^43^ Motivated by this discovery, we incorporated this modification into our IdoA-based disaccharides to investigate its potential effects on neural growth.

To further examine the effect of GlcNAc sulfation in a disaccharide context, we synthesized two derivatives: **D1** (IdoA2S4S-GlcNAc), lacking 6-*O*-sulfation, and **D2** (IdoA2S4S-GlcNAc6S), which retains it.

Given the established role of *N*- and 6-*O*-sulfation on glucosamine (GlcN) residues for high-affinity HS-protein interactions, we designed **D3** (GlcANS6S-GlcNS6S) to interrogate the combined contribution of these modifications.^44,45^ In addition to sulfation, oligosaccharide length mediates binding avidity via multivalent interactions with targets such as FGFs and AT-III, which typically engage extended sulfated domains. To explore these length-dependent effects, we synthesized the fully sulfated tetrasaccharide **T1** (Glc2S3S-Glc2S3S6S-Glc2S3S6S-Glc2S3S), chosen for its synthetic feasibility and structural coherence with our HS glycomimetic library, serving as a representative model of a densely sulfated oligosaccharide.^46^ Taken together, this set of well-defined HS epitopes enables targeted investigation of the molecular determinants of HS-protein recognition and offers critical insights into minimal structural requirements underlying biological activity.

The synthesis of HS glycomimetics was accomplished through a modular strategy employing a set of orthogonal protecting groups, which enable regioselective sulfation at the predefined positions along the carbohydrate backbone. As a representative example, the synthetic route to **D2** is outlined in Scheme 1, while the preparation of other targets is elaborated in the Supporting Information (SI). For the synthesis of **D2**, to access the acceptor, GlcNAc was first glycosylated with 2-chloroethanol, followed by sequential azidation, 4,6-benzylidene acetal installation, and benzylation. First, trifluoroacetic acid (TFA)-mediated cleavage of the 4,6-*O*-benzylidene acetal was performed, followed by protection with a 4,6-*O*-(2-naphthylmethylbenzylidene) group. Subsequent acid-catalyzed ring-opening of this 4,6-*O*-(2-naphthylmethylbenzylidene) group selectively afforded the 6-*O*-(2-naphthylmethyl) ether, yielding the acceptor for **D2** synthesis. A common thioethyl l-idosyl donor for **D1** and **D2**, was synthesized from 1,2-*O*-(1-methylethylidene)-3-*O*-(phenylmethyl)-α-d-glucofuranose in eight steps. Silver triflate (AgOTf) and *N*-iodosuccinimide (NIS)-promoted glycosylation of the acceptor by the thioglycoside donor yielded the core disaccharide (Scheme 1, compound 26), which was further deprotected and sulfated to afford the target compound.

**Scheme 1:**
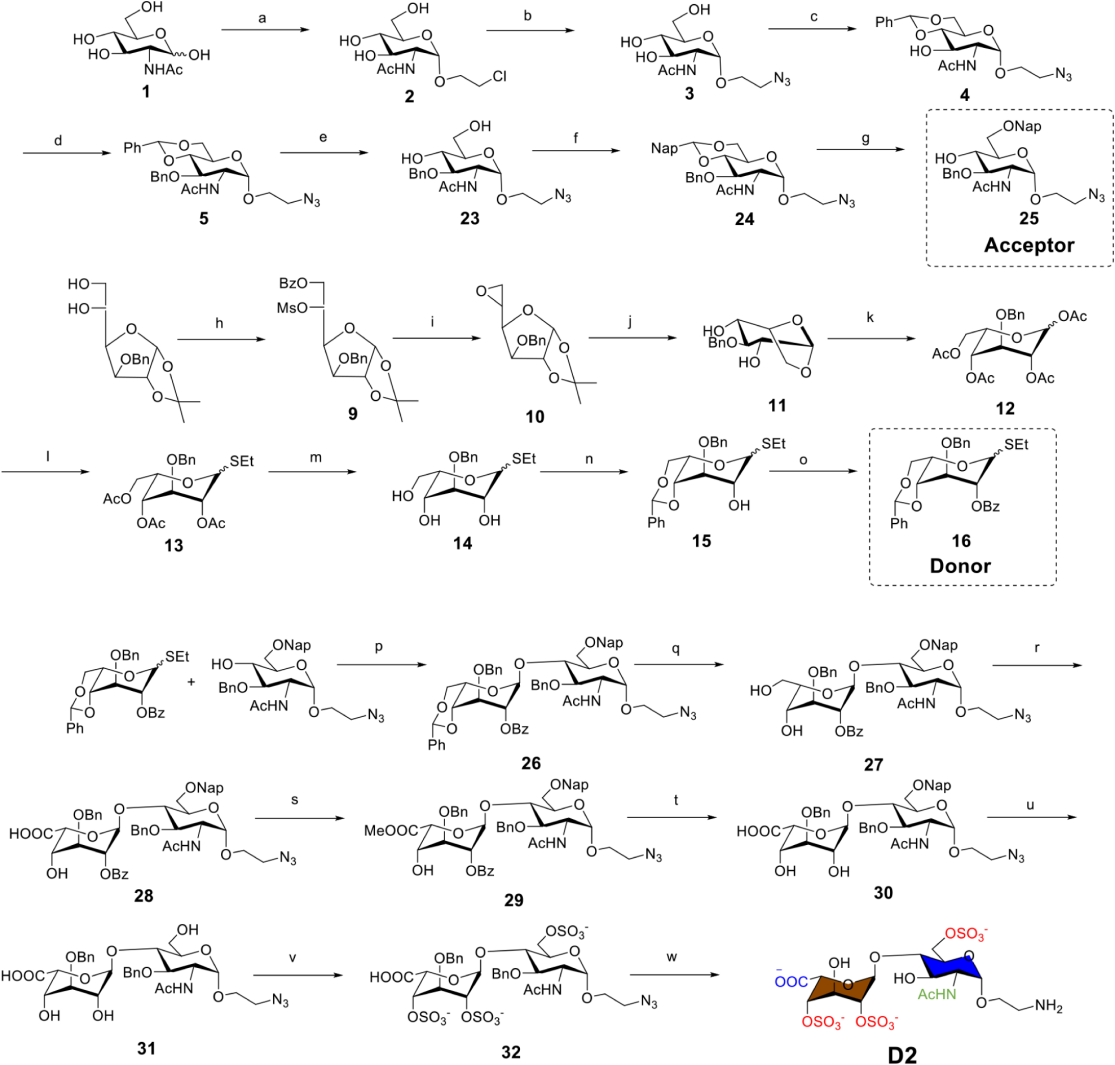
Modular Synthesis of Disaccharide D2:^a^

a Reagents and conditions: (a) 2-chloroethanol, AcCl, 70 °C- R.T., 8 h, 60%; (b) NaN_3_, DMF, 60 °C, 12 h, 62%; (c) PhCH­(OMe)_2_, *p*TsOH, DMF, 60 °C, 12 h, 80%; (d) NaH (60% dispersion in mineral oil), BnBr, DMF, THF, 0 °C – R.T. 1 h, under N_2_, 86%; (e) TFA, H_2_O, DCM, 30 °C, 9 h, 61%; (f) NapCH­(OMe)_2_, *p*TsOH, MeCN, 50 °C, 12 h, 65%; (g) NaBH_3_CN, 2 N HCl/Et_2_O, R.T., 6 h, 75%; (h) (i) BzCl, DCM, Py, 0 °C, 2 h; (ii) MsCl, R.T., 12 h, 52%; (i): *t*-BuOH, KOtBu, DCM, 0 °C, 16 h, 60%; (j) (i) 1,4-dioxane, H_2_SO_4_, reflux, 120 °C, 22 h; (ii) NaOH, 0 °C, 59%; (k): Ac_2_O, TFA, 0 °C, 24 h, 84%; (l) BF_3_.Et_2_O, EtSH, DCM, 0 °C - R.T., 2.5 h, 60%; (m): NaOMe, MeOH/DCM, R.T., 5 h, 80%; (n) PhCH­(OMe)_2_, CSA, DMF, R.T., 12 h, 80%; (o): BzCl, Py, 0 °C – R.T., 12 h, 82%; (p) AgOTf, NIS, molecular sieves (4 Å), DCM, 0 °C, 30 min, 33%; (q) TFA/H_2_O, DCM, R.T., 4 h, 38%; (r) TEMPO, BAIB, DCM/H_2_O, R.T., 6 h; (s) MeI, KHCO_3_, DMF, R.T., 12 h, 45%; (t) LiOH, H_2_O_2_, THF, 40°C, 12 h; (u) DDQ, DCM/MeOH, R.T., 6 h, 52%; (v) SO_3_.EtN_3_, DMF, 12 h, 61%; (w) Pd­(OH)_2_/C, MeOH/H_2_O, under H_2_, R.T., 24 h, 50%. Abbreviations: **Ac**: acetyl; **Bn**: benzyl; **Bz**: benzoyl; **Me**: methyl; **Ms**: mesyl; **Nap**: 2-naphthylmethyl; **Ph**: phenyl; **SEt:** thioethyl. Detailed synthetic schemes and procedures for all compounds, along with their corresponding spectral data, are provided in the Supporting Information (SI).

With this structurally diverse library in hand, we next sought to evaluate how sulfation pattern and oligosaccharide length govern selective protein binding.

### 2.2 Dissecting Protein-HS Glycomimetic Binding Kinetics

To systematically probe the protein-binding profiles of our synthetic glycomimetics, we selected four representative HS-interacting proteins: FGF-1, FGF-2, NGF, and AT-III. These targets span both desired neurotrophic proteins and a known off-target mediator of anticoagulation. FGF-1 and FGF-2 are heparin-binding GFs with critical roles in cell proliferation, migration, and neural development.^47^ NGF regulates neuronal differentiation and survival,^48^ while AT-III represents a key challenge for translational HS-based therapies due to its role in coagulation.^49^ This panel enables simultaneous evaluation of glycomimetic selectivity and therapeutic specificity.

We evaluated the binding affinity of native HS and synthetic HS glycomimetics (**M1**, **D1**, **D2**, **D3**, and **T1**) to these proteins using biolayer interferometry (BLI).^50^ The terminal amino groups of the sugars were immobilized onto the sensor surface using standard amine coupling chemistry, and binding assays were performed by introducing the proteins as analytes at varying concentrations (Figure 2A). The equilibrium dissociation constants (*K*_D_) were determined using the steady-state affinity curve fitting. The measured *K*_D_ values for the native HS-protein interactions (Figure 2A) were as follows: FGF-1 (*K*_D_ = 273 nM), FGF-2 (*K*_D_ = 81.0 nM), NGF (1 and 10 μM, two-site binding model), and AT-III (*K*_D_ = 0.4 μM). These values are consistent with previously reported data.^51–54^ The synthetic HS glycomimetics exhibited a concentration-dependent increase in response for all proteins, except for AT-III. T1 showed the best binding affinities, but was the only one that bound to AT-III as well (Table S4.1 in SI). The best response was that of **D2**, with good *K*_D_ values for FGF-1 (*K*_D_ = 0.78 μM), FGF-2 (*K*_D_ = 0.67 μM), and NGF (*K*_D_ = 2.6 μM and 468 μM, two-site binding model), but no measurable binding to AT-III (Figure 2A).

**Figure 2:**
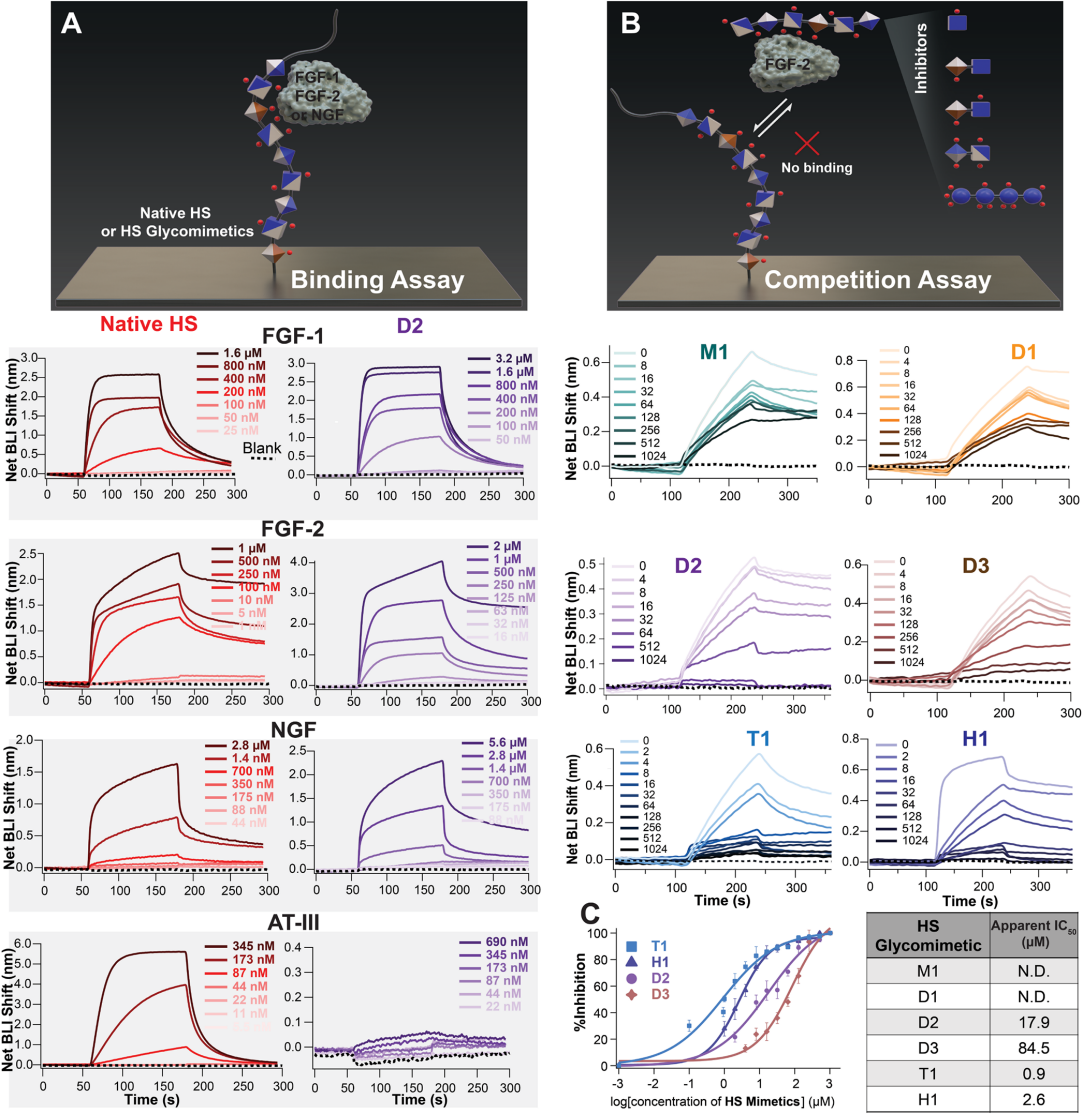
Characterization of the molecular interactions between HS glycomimetics and proteins: (A) BLI binding assays where sugars were immobilized onto the surface of the sensor probe, and the proteins at different concentrations were employed as analytes. Representative BLI sensorgrams showing binding responses for the interaction between proteins (FGF-1, FGF-2, NGF, AT-III) and sugars (native HS compared with **D2**). (B) BLI competition assays where native HS was immobilized onto the surface of the sensor probe, and the FGF-2 was premixed with HS glycomimetics to compete with native HS for FGF-2 binding. Representative BLI sensorgrams showing FGF-2 binding of the various concentrations of HS glycomimetics (μM) in competition with the immobilized native HS. (C) Inhibition curves of **T1**, **H1**, **D2**, and **D3** based on competition assays and apparent half-maximal inhibitory concentration (IC_50_(*t*)) values, calculated as the inhibitor concentration that reduced the FGF-2 response by 50% at a fixed association time (*t* = 240 s). Data represent the mean ± SD of three independent experiments performed under identical conditions. ND stands for not determined.

We next examined the functional relevance of the synthetic HS glycomimetics by testing their ability to compete with native HS for FGF-2 binding using competition assays. Although direct binding assays measure the intrinsic affinity of HS glycomimetics for FGF-2, they are limited in representing the complex, multimolecular environment of biological systems. *In vivo*, FGF-2 activity is tightly regulated by its interactions with GAGs, which are abundant on cell surfaces and in the ECM.^55^ These interactions stabilize FGF-2 and facilitate its dimerization with FGF receptors (FGFRs), a requirement for effective signal transduction.^9,56,57^ Thus, therapeutic candidates targeting FGF-2 must be evaluated not only for direct binding but also for their ability to modulate or disrupt GAG-mediated interactions.^58^ Therefore, we performed competition assays in which native HS was immobilized on the sensor surface. Solutions containing a fixed concentration of FGF-2 (50 nM) were premixed with varying concentrations of HS glycomimetics, functioning as inhibitors in this context, (**M1**, **D1**, **D2**, **D3**, and **T1**) and also with the heparin hexasaccharide (**H1**) (GlcNS6S-GlcA-GlcNS3S6S-IdoA2S-GlcNS6S-GlcA), a well-studied HS glycomimetic in the literature, as a positive control (Figure 2B). Our synthetic HS glycomimetics **D2**, **D3**, and **T1** demonstrated the ability to compete with immobilized native HS for FGF-2 binding, resulting in a dose-dependent reduction in signal intensity, whereas **M1** and **D1** had a much weaker effect even at the highest concentrations tested. The progressive decrease in signal intensity with increasing inhibitor concentration directly reflects competition with immobilized HS for FGF-2 binding.

To enable meaningful comparison among glycomimetics in their ability to compete with native HS, the inhibitory potency of each compound was expressed as a time-dependent apparent IC_50_ value, IC_50_(*t*), representing the inhibitor concentration needed to reduce the FGF-2 response by 50% at a fixed association time (*t* = 240 s). Although these apparent IC_50_(*t*) values do not represent equilibrium constants, they provide a robust and internally consistent measure of relative inhibition, as all the inhibitors were analyzed under identical conditions. Under these conditions, the heparin hexasaccharide **H1** had an IC_50_ (*t* = 240 s) of 2.61 μM. Among our HS mimics, **T1**, a fully sulfated tetrasaccharide, showed the most potent inhibition (IC_50_ = 0.91 μM), outperforming **H1** due to its higher sulfation level. Conversely, while **D3** has a higher sulfation level than **D2**, its inhibitory activity (IC_50_ = 84.5 μM) was lower than D2’s (IC_50_ = 17.9 μM), highlighting the importance of specific monomeric units in FGF-2 recognition. In contrast, **M1** and **D1** exhibited minimal inhibition within the same concentration range.

### 2.3 Molecular Modeling

To gain structural insight into the molecular basis of glycomimetic-protein interactions, we performed molecular docking studies of glycomimetics with GFs. Molecular modeling aimed to complement our experimental findings by visualizing the binding poses, electrostatic contacts, and conformational preferences that underlie the observed interactions. It provides a predictive framework for deciphering GAG-protein interactions, particularly in light of the difficulties associated with resolving their structures by crystallography.^59^ However, accurately capturing these interactions remains challenging due to the inherent conformational flexibility of GAGs, their high charge density, and the typically weak surface complementarity at GAG-binding interfaces. In spite of these complexities, molecular docking has been successfully applied to map GAG-binding sites and propose interaction modes, offering structural perspectives on protein recognition of carbohydrate ligands.^60^ In this study, we employed molecular docking to approximate the interactions between our rationally designed HS glycomimetics and key neurotrophic proteins, including FGF-1, FGF-2, and NGF, to rationalize our experimental findings. Our approach involved an initial broad sampling to identify favorable binding regions, followed by refined docking to optimize binding poses based on predicted binding energy. This strategy allowed us to pinpoint the most thermodynamically favorable configurations for subsequent validation.

To examine the interactions with FGF-1, we used the crystal structure of a heparin hexasaccharide bound to two FGF-1 molecules (PDB ID: 2AXM)^61^ as a reference structure, and we docked our synthetic HS glycomimetics to the available FGF-1 structure. To improve prediction accuracy and allow direct sugar-protein side chain interactions, all crystallographic water molecules were removed, as they may mediate or compete with binding interactions. Results indicated that our synthetic HS glycomimetics occupied binding sites similar to those of native HS-engaging key residues such as lysine residues, K112, K113, K118, K128, and an arginine residue, R122. Notably, while both FGF-1 molecules in the reference structure interacted with heparin at a common binding region, they exhibited distinct sets of interacting residues when binding our compounds, featuring the adaptability of HS glycomimetics within the FGF-1 binding pocket. The most favorable binding pose of compound **D2** with FGF-1 is depicted in Figure 3A, with interacting residues mapped within 5 Å of the ligand. Binding interactions for the rest of the compounds in the library are provided in Figure S2.1–S5.

**Figure 3:**
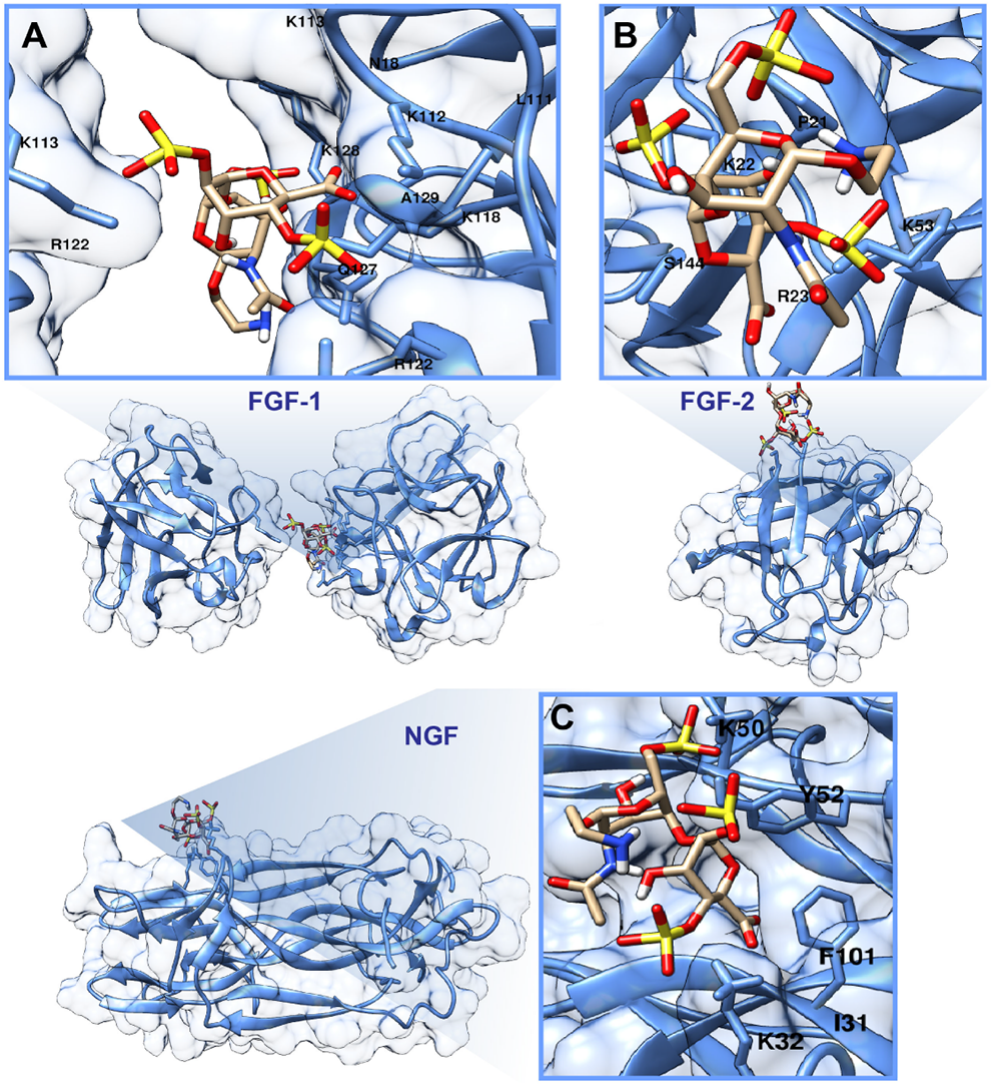
Predicted binding poses of glycomimetic **D2** with neurotrophic proteins based on molecular docking: (A) FGF-1 and (B) FGF-2, with **D2** docked into the canonical heparin-binding site. Residues within 5 Å of the ligand are shown in the zoomed upper panels; the full protein context is shown in the lower panels. (C) NGF bound to **D2**, with the left panel depicting the full protein surface and the right panel highlighting residues within 5 Å. **D2** is shown in stick representation with atoms colored as follows: C-tan, O-red, S-yellow, and N-dark blue.

To model the interactions of our HS glycomimetics with FGF-2, we used the crystal structure of a heparin tetrasaccharide bound to FGF-2 (PDB ID: 1BFB)^9^ as a structural reference (Figure S2.11). In contrast to FGF-1, the native HS ligand in this structure engages a single FGF-2 molecule. Our docking studies revealed that several critical binding interactions, such as those involving K136 and R141, were conserved in compounds **D3** (Figure S2.9) and **T1** (Figure S2.10). However, additional residues were implicated in interactions with other synthetic compounds, suggesting potential binding adaptations distinct from native HS. Given that the reference crystal structure includes additional contacts with three adjacent FGF-2 molecules, it is plausible that these intermolecular interactions, along with crystallographic water effects, influence the conformation of the native ligand, thereby modulating its binding profile. The most favorable binding pose of compound **D2** with FGF-2 is presented in Figure 3B.

Expanding our investigation beyond FGFs, we explored the binding interactions of HS glycomimetics with NGF, a critical modulator of neuronal survival and regeneration.^62^ Unlike FGF signaling, reports detailing small-molecule ligands that modulate NGF activity remain scarce, underscoring the need for further exploration. To this end, we performed molecular docking using the NMR-resolved structure of recombinant human NGF.^63^ Previous work identified a basic domain on native β-NGF that can interact with heparin, albeit with very low affinity.^64^ Our docking results reveal that some residues, particularly K32 and I31, remain conserved in binding across four of the five compounds examined, except for **M1**, which binds with other alternative residues within NGF. The most favorable binding pose of compound **D2** with NGF is depicted in Figure 3C, while binding interactions for additional compounds are provided in Figures S2.12–S16.

### 2.4 Structural and Functional Implications of Protein-HS Glycomimetic Interactions

To select a lead compound, we complemented the affinity data with a series of studies evaluating the structural and functional implications of protein-HS glycomimetic interactions. We assessed the therapeutic safety profile of our HS glycomimetics. We also employed circular dichroism (CD) spectroscopy to assess secondary structural changes in protein conformation upon binding to native HS and HS glycomimetics. Since native HS alone produces only a minimal signal, it was ensured that its contribution would not interfere with protein-sugar complex spectra.^65^ Therefore, only the far-UV CD spectra of bare proteins and their sugar complexes were recorded. The spectra of FGF-1 and FGF-2 displayed characteristic features of β-sheet-rich proteins, with a minimum around 205–207 nm and a broad maximum centered at 228 nm. Upon incubation with sugars (1:3 molar ratio), significant spectral changes were observed, indicating sugar-induced alterations in secondary structure (Figure 4A). In contrast, AT-III, which exhibited a broad minimum with strong negative ellipticity centered at 215 nm, showed no spectral changes upon binding to HS glycomimetics. However, in the presence of native HS, ellipticity became more negative, suggesting an increase in β-sheet content or stabilization of existing β-sheets.

**Figure 4:**
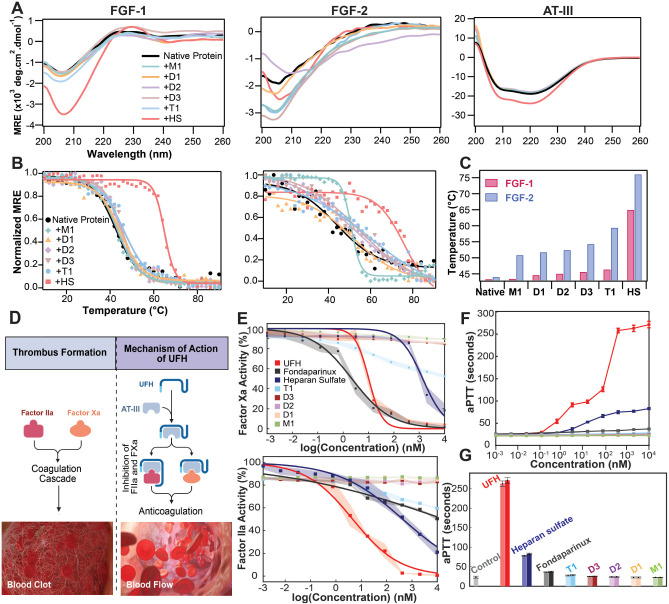
Structural and functional analysis of proteins in the presence of sugars: Far-UV circular dichroism (CD) spectra and thermal denaturation profiles of native proteins and proteins upon incubation with sugars: (A) far-UV CD spectra, from left to right: FGF-1, FGF-2, and AT-III. (B) The temperature-induced unfolding of FGFs is represented as a change in protein stability, with 1 indicating a fully folded state and 0 indicating an unfolded state. Markers denote variations in ellipticity at λ=225 nm, as obtained from CD spectra of a specific protein at temperatures from 10 to 90 °C. Solid lines denote sigmoidal fits to the experimental data. (C) Melting temperatures (*T*_m_) of specific samples defined as the temperature at which half of the protein remains unfolded. Determination of anticoagulant properties of sugars: (D) Schematic representation of anticoagulant activity. Upon binding to AT-III, UFH enhances its interaction with coagulation factors IIa or Xa, leading to rapid inactivation of FIIa and FXa, which is crucial for blocking clot formation, created in Biorender. (E) AT-III-mediated Factor Xa and Factor IIa activity measured by chromogenic substrate assays. Each data point represents the mean of three separate measurements, with data presented as mean ± SD. (F) Activated partial thromboplastin time (aPTT) measurements: sugars were added directly to pooled rat plasma at the specified concentrations and then assayed for the aPTT. Each data point represents the mean of three independent measurements, with data presented as mean ± SD. (G) Comparison of sugars: the control sample contained no added sugar. Light-shaded bars correspond to 2 μM and dark-shaded bars to 10 μM concentrations of each sample. Data represent mean ± SD (*n* = 3).

We also evaluated the thermal stability of the proteins and their sugar complexes by CD-based thermal denaturation experiments. The spectra were recorded across a temperature range of 10–90 °C (Figure 4B). For all proteins except AT-III, the unfolding profiles were analyzed to determine the melting temperature (*T*_m_), representing the midpoint of the transition from the folded to the unfolded state, an indicator of structural integrity and resistance to thermal denaturation.^66^ The calculated values are compiled in Table 1.

**Table 1:** Melting Temperatures (*T*_m_) of FGF Samples in the Presence of HS Glycomimetics

	FGF-1 Sample *T*_m_ (°C)	FGF-2 Sample *T*_m_ (°C)
**Native Protein**	43.2 ± 0.4	43.9 ± 1.7
**+M1**	43.3 ± 0.4	50.7 ± 0.2
**+D1**	44.5 ± 0.4	51.6 ± 1.9
**+D2**	45.0 ± 0.4	52.4 ± 1.2
**+D3**	45.3 ± 0.3	54.2 ± 1.2
**+T1**	46.3 ± 0.5	59.3 ± 2.2
**+HS**	64.9 ± 0.2	75.9 ± 1.9

For FGF-1, the native protein had a *T*_m_ of 43.2 °C, and binding to **M1** resulted in a negligible increase (43.3 °C). However, increasing sulfation led to progressively higher *T*_m_ values, with **D1** (44.5 °C), **D2** (45.0 °C), **D3** (45.3 °C), and **T1** (46.3 °C) (Figure 4C and Table 1), likely due to enhanced electrostatic interactions and hydrogen bonding. The most pronounced stabilization was observed with native HS, which increased *T*_m_ to 64.9 °C, emphasizing its strong affinity and structural reinforcement. A similar but more distinct stabilization effect was observed for FGF-2 (Figure S3.1 and Table 1), where the native protein had a *T*_m_ of 43.9 °C, comparable to that of FGF-1. Sugar binding resulted in a marked increase in thermal stability, with *T*_m_ values reaching 50.7 °C (**M1**), 51.6 °C (**D1**), 52.4 °C (**D2**), 54.2 °C (**D3**), and 59.3 °C (**T1**). The greatest stabilization was again seen with native HS (*T*_m_ = 75.9 °C), indicating strong interactions that significantly enhance the thermal stability of FGF-2. The greater stabilization effect observed in FGF-2 compared to FGF-1 suggests fundamental differences in their sugar-binding properties, binding site architecture, or conformational flexibility. Sugar binding may not only strengthen local interactions but also induce global structural rigidity, reducing structural fluctuations that contribute to unfolding. The thermal denaturation profile of NGF was also examined. Unlike FGFs, the unfolding behavior of NGF was more complex, rendering a precise determination of *T*_m_ difficult, likely due to heterogeneous structural transitions (Figure S3.2).

We examined whether the HS glycomimetics affect blood coagulation to ensure their safety and specificity in therapeutic contexts. A major endogenous inhibitor of the coagulation cascade, AT-III deactivates Factor Xa (FXa) and Factor IIa (thrombin, FIIa), both of which are necessary for the formation of fibrin clots.^67,68^ To benchmark our compounds, we compared their behavior with unfractionated heparin (UFH), Fondaparinux, and native heparan sulfate, which represent a spectrum of anticoagulant potencies. UFH produces anticoagulant effects by binding to AT-III, and this interaction increases its inhibitory activity against both FIIa and FXa (Figure 4D). On the other hand, Fondaparinux, a synthetic HS mimicking pentasaccharide which has received clinical approval as an anticoagulant drug, can inhibit only FXa, but not FIIa. In contrast, heparan sulfate displays only weak anticoagulant properties compared to UFH, reflecting its lower sulfation degree and partial engagement of AT-III and heparin cofactor II (HCII).^69^

We conducted absorbance-based chromogenic tests measuring AT-III-mediated FIIa and FXa activity to determine whether our HS glycomimetics exhibit undesired anticoagulant properties. Active coagulation factors cleave chromogenic peptide substrates, therefore producing *p*-nitroaniline, a yellow-colored product with a detectable absorbance signal at λ = 405 nm. Inhibitors like heparin-AT-III complexes decrease enzyme activity and so lower absorbance readings. As anticipated, UFH suppressed both FIIa and FXa, while Fondaparinux selectively inhibited FXa. Heparan sulfate showed weak inhibition of both enzymes; however, it has been previously reported to exert a significant antithrombotic effect *in vivo* in a rabbit stasis thrombosis model.^70^ Our HS glycomimetics, on the other hand, did not block either coagulation factor, implying they do not disrupt regular clotting processes (Figure 4E).

To complement these findings, we performed an activated partial thromboplastin time (aPTT) assay in citrated pooled rat plasma to assess the overall impact of the compounds on the intrinsic coagulation pathway. This global clotting assay measures the time required for fibrin formation and is sensitive to anticoagulant effects.^71^ Figure 4F,G compare the effects of UFH, heparan sulfate, Fondaparinux, and the synthetic glycomimetics when supplemented into citrated plasma. The control (no sugar) sample exhibited a baseline clotting time of 22 s. As expected, UFH produced a strong, concentration-dependent anticoagulant response, prolonging the mean clotting time to 271 s at the highest concentration tested, while heparan sulfate and Fondaparinux induced weaker but measurable effects (83 and 37 s, respectively). In contrast, our synthetic glycomimetics, **T1** (28.5 s), **D3** (25.3 s), **D2** (24 s), **D1** (22.5 s), and **M1** (22 s), showed average clotting times indistinguishable from the control, confirming the absence of intrinsic anticoagulant activity. These quantitative data demonstrate that while native heparan sulfate retains weak but detectable anticoagulant activity, the designed glycomimetics do not perturb physiological coagulation. The results further confirm that selective sulfation patterns can decouple GF recognition from anticoagulant function, an essential prerequisite for their translational application in regenerative medicine. Though IC_50_ values were undetermined, **T1**, the fully sulfated tetrasaccharide, showed slight interactions with FIIa and FXa and increased clotting time from 22 to 28.5 s. These interactions are in line with nonspecific electrostatic binding, a feature of highly sulfated oligosaccharides known to engage different proteins via charge-based, rather than specific, biologically targeted interactions. Thus, to avoid potential off-target effects associated with highly sulfated analogues, we selected the disaccharide **D2** for subsequent biological experiments. **D2** exhibited robust growth factor stabilization without any anticoagulant activity, thereby fulfilling the key safety and efficacy criteria for a regenerative medicine lead.

### 2.5 Surface-Immobilized HS Glycomimetics Promote Neural Maturation

We next examined the NGF-induced neuritogenic potential of the HS glycomimetics to determine whether they could potentiate neurotrophin-driven neuronal differentiation. To mimic the native role of HS proteoglycans in the ECM, which spatially sequester and present growth factors, we immobilized **D2** and also native HS as a positive control onto culture substrates and evaluated its capacity to modulate neural maturation in two well-established neuronal cell models of different species origins, rat-derived PC12 cells and human-derived SH-SY5Y cells. These models represent distinct stages of neuronal differentiation and maturation, and the choice enabled us to compare cellular responses across species, thereby enhancing the translational relevance of our findings. PC12 cells respond to NGF by exiting the cell cycle and extending neurites, while SH-SY5Y cells serve as a model for later-stage maturation and neurite elongation.^72,73^

In this context, the immobilization of **D2** onto culture substrates provided a means to investigate how its presentation as an ECM-like component influences neurotrophic responses, particularly in combination with NGF. In both cell lines, **D2** and native HS were applied to the substrate prior to seeding, thereby mimicking its natural role as a structural component of the ECM. This approach allowed for the assessment of how immobilized **D2** modulates cellular responses by altering the local presentation of growth factors. NGF was added to both **D2**-coated, native HS-coated, and uncoated conditions at a uniform concentration (50 ng/mL), enabling direct comparison of morphological outcomes under identical soluble signaling environments (Figure 5A).

**Figure 5:**
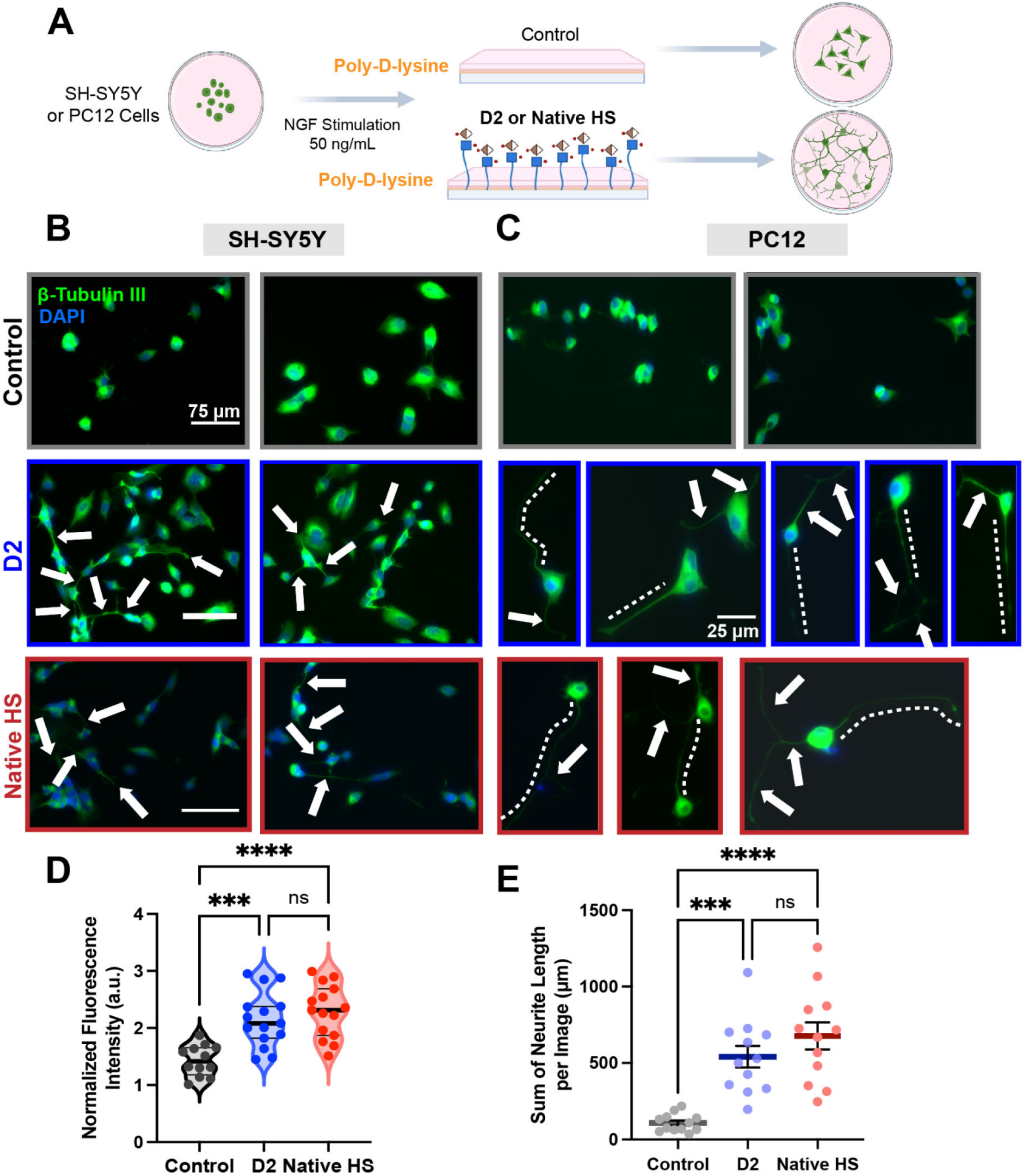
Immobilized glycomimetic D2 enhances NGF-mediated neuronal maturation in PC12 and SH-SY5Y cell models, comparable to native HS. (A) Schematic representation of the experimental workflow. SH-SY5Y and PC12 cells were seeded on culture substrates either uncoated (control) or coated with immobilized glycomimetic **D2** or native HS and stimulated with NGF (50 ng/mL) to assess effects on neuronal maturation. Representative immunofluorescence micrographs of (B) SH-SY5Y and (C) PC12 cells stained with anti-β-tubulin III (green) and DAPI (blue) to visualize neurites and nuclei, respectively. Longest neurites are marked with white dashed lines; white arrows indicate additional neuritic projections. (D) Quantification of normalized fluorescence intensity in SH-SY5Y cells. Each dot represents an individual image measurement. Mean intensities are indicated by black solid horizontal lines at 1.43 for control, 2.15 for **D2**-coated, and 2.29 for native HS-coated surfaces. (E) Quantification of total neurite outgrowth in PC12 cells. Solid dots represent individual images. Values are expressed as mean ± SEM. Horizontal lines show the average sum of neurite length per image: 108 μm for control, 541 μm for **D2**-coated, and 676 μm for native HS-coated surfaces, based on randomly selected images per group. Statistical significance determined using a one-way ANOVA test: ns *p* > 0.05, ***p < 0.001, and ****p < 0.0001.

In PC12 cells, which serve as a classical model for NGF-induced neuronal differentiation, the presence of both immobilized **D2** and native HS coatings led to notable morphological changes relative to control conditions (Figure 5C). Cells grown on either substrate exhibited more pronounced neuritic differentiation. Morphological observations revealed a higher number of neurite-like projections. Immunofluorescence staining with anti-β-tubulin III conjugated to Alexa Fluor 488 enabled visualization of these features, selectively highlighting neuronal processes and allowing for a detailed qualitative assessment of neurite formation and organization. Fluorescence imaging showed that PC12 cells on **D2**- and native HS-coated surfaces developed more extensive and complex neuritic networks compared to controls. Neurites appeared longer, more branched, and more numerous per cell, indicating that surface-bound **D2** and native HS actively support and enhance NGF-mediated differentiation. Quantitative image analysis (NeuronJ, ImageJ) showed a substantial increase in total neurite outgrowth per image from 108 μm in the control to 541 μm on **D2**-coated and 676 μm on HS-coated substrates based on randomly selected images per condition (Figure 5E). Importantly, statistical analysis revealed no significant difference between **D2** and native HS conditions, demonstrating that the synthetic glycomimetic reproduces the neurotrophic efficacy of native HS with comparable magnitude. Although images used for quantification were selected from well-dispersed regions, we also observed instances of cell clustering in both control and **D2** groups (Figure S6.1, Supporting Information). Approximately 20% of cells appeared in aggregates across conditions. These clusters exhibited neuritic projections for the **D2** group. These results highlight the potent neuritogenic effect of immobilized **D2**.

Mechanistically, these observations indicate that **D2** mimics the functional role of native HS by spatially organizing and stabilizing NGF at the cell–substrate interface, where it functions to recruit, stabilize, and present GFs to their cognate receptors.^27^ By anchoring NGF at the substrate interface, **D2** may emulate this natural HS function, spatially controlling the concentration of NGF and facilitating its interaction with TrkA receptors on the cell surface. This enrichment likely amplifies receptor activation and downstream signaling cascades that drive morphological differentiation.

Similarly, in SH-SY5Y cells, which are frequently used as a model of neuronal maturation, both **D2**-and native HS-coated surfaces supported increased neurite elongation and a greater proportion of neurite-bearing cells in the presence of NGF compared to the control groups (Figure 5B). Quantification of neurite-associated β-tubulin III fluorescence intensity revealed a significant increase in signal intensity for both conditions relative to NGF alone, with mean normalized values of 2.15 for **D2**, 2.29 for native HS, and 1.43 for the control (Figure 5D). Statistical analysis showed no significant difference between **D2** and native HS, confirming that the synthetic glycomimetic replicates the neurotrophic efficacy of endogenous HS within this cellular context.

These findings further reinforce the concept that sulfated glycans act as instructive molecular scaffolds, where the precise arrangement of sulfate groups and monosaccharide units encodes distinct biological outcomes. In this framework, **D2** captures the pro-regenerative architecture of native HS while offering chemical precision and safety. Similar principles have been demonstrated with different GAGs.^74^ For example, substrates bearing specific chondroitin sulfate (CS) variants, CS-A and CS-B, selectively guided neurite extension, while neurons actively avoided CS-C regions, demonstrating that sulfation motifs alone can provide directional cues for neuronal processes even in the absence of core proteins.^75^ In another study, CS-E has been shown to inhibit axon regeneration.^76^ In these systems, the sulfation pattern dictated not only GFs but also neurite orientation, revealing the instructive capacity of immobilized GAGs.^77^

### 2.6 Functional Validation in Primary Neurons: Probing Growth Factor Stabilization and Synergistic Bioactivity

We next sought to explore whether this molecular interaction translates into enhanced functional outcomes in a physiologically relevant system. In native tissue, ECM not only binds but also preserves growth factors, prolonging their signaling competence. We hypothesized that **D2**, by mimicking this ECM role, could similarly stabilize and potentiate FGF-2 signaling in primary neuronal cultures. Using dissociated rat hippocampal neurons, we tested whether coadministration of **D2** and FGF-2 would result in a synergistic enhancement of neuronal outgrowth and synaptic activity, beyond the effect of FGF-2 alone. This allowed us to probe the efficacy of glycomimetic-GF complexes in a complex, developmentally relevant cellular environment.

In the pursuit of this, we used rat dissociated hippocampal neurons as an *in vitro* model system. We assessed the impact of HS glycomimetics on neuronal morphology, using immunofluorescence imaging and quantitative morphometric analysis, and on synaptic functionality through electrophysiological monitoring of spontaneous neuronal activity.

During the first day of differentiation *in vitro* (Table S7.1 in SI), neurons were treated for 24 h with either control medium, glycomimetic **D2** (1 μM), FGF-2 (40 ng/mL) alone, or a combination of FGF-2 and **D2** (40 ng/mL and 1 μM, respectively). After treatments, cultures were fixed and immuno-labeled with antibodies against the neuronal marker β-Tubulin III to study cell morphology. As shown in representative images of Figure 6A, neurons treated with **D2**+FGF-2 exhibited a more elaborate neurite arborization compared to other conditions, suggesting a synergistic effect between the **D2** and the GF. Quantitative analysis further corroborated these observations (Figure 6B–C). While neurons treated with a control medium (3.6 ± 0.22 neurites per neuron) and **D2** alone (3.8 ± 0.23 neurites per neuron) exhibited no significant differences (*p* > 0.05), FGF-2 treatment alone significantly increased the number of neurites emerging from the cell body (5.4 ± 0.28 neurites per neuron, *p* < 0.05 vs control and **D2** alone). The combination of **D2**+FGF-2 resulted in an even greater enhancement, with an average of 6.8 ± 0.38 neurites per neuron, significantly surpassing all other conditions (*p* < 0.05 vs control, **D2** alone, and FGF-2 alone; Figure 6B). A similar trend was observed for neurite length (Figure 6C). While neurons in control (38 ± 2 μm), **D2** alone (43 ± 3 μm), and FGF-2 alone (45 ± 4 μm) groups presented comparable neurite extensions (*p* > 0.05), **D2**+FGF-2 treatment significantly increased the longest neurite length to 59 ± 5 μm in comparison to controls (*p* < 0.05 control vs **D2**+FGF-2). This effect was particularly noteworthy, as FGF-2 alone failed to induce significant neurite elongation, suggesting that **D2** plays a distinct role in modulating FGF-2 signaling to promote neurite growth.

**Figure 6:**
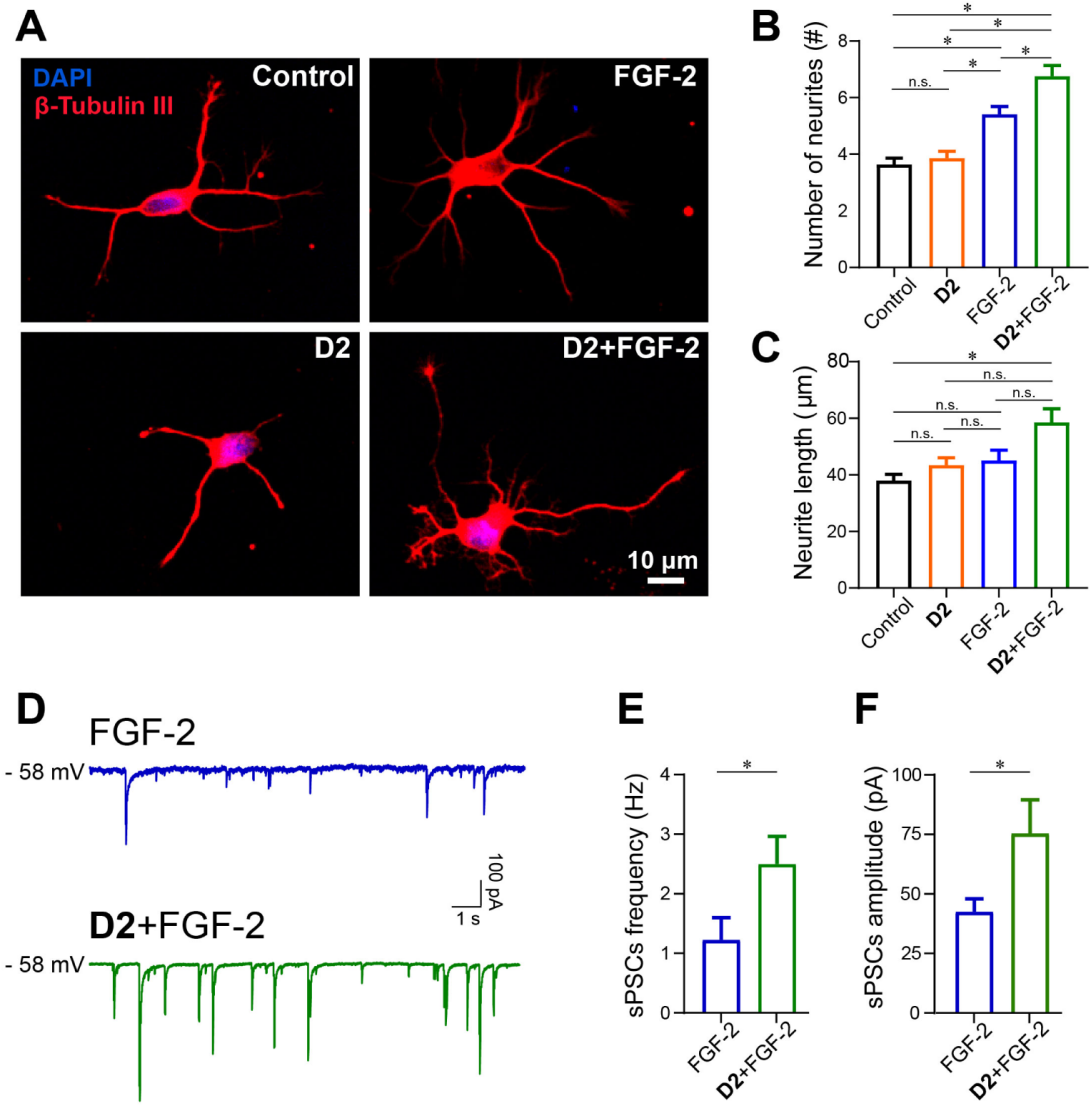
Neuritogenic Activityactivity of HS Glycomimeticsglycomimetics in dissociated hippocampal cultures after 24 h of treatment. (A) Representative immunofluorescence micrographs of hippocampal cultures at 1 day of in vitro differentiation, where nuclei are labeled with DAPI (in blue) and cells are marked with antibodies against the neuronal marker β-tubulin III (in red) for control, **D2**, FGF-2, and **D2**+FGF-2 treatments. Bar plots for (B) the number of neurites emerging from the cell body and (C) the length of the longest neurite, for the different experimental conditions. For each condition, the parameters were measured in 30 optical fields from 6 different cultures. *n* = 105 cells in Control, *n* = 95 cells for **D2**, *n* = 103 cells in FGF-2 and *n* = 103 cells in **D2**+FGF-2. (D) Exemplificative voltage-clamp recordings at the holding potential of −58 mV from neurons at 8 days of in vitro differentiation, treated with FGF-2 or **D2**+FGF-2. Bar plots showing (E) the mean values of sPSCs frequency and (F) amplitude for the different treatments. *n* = 18 cells for FGF-2 and *n* = 21 cells for **D2**+FGF-2. Values are expressed as mean ± SEM. *p < 0.05.

In another set of experiments, we investigated the impact of **D2**+FGF-2, with respect to that of FGF-2 alone, on the formation of functionally active neuronal networks. To this aim, we used hippocampal neurons developed *in vitro* for 1 week, from which, through the single-cell patch clamp technique, we recorded in voltage clamp mode spontaneous synaptic activity. As shown by the electrophysiological traces in Figure 6D, after 24 h long-lasting treatment with FGF-2 alone or **D2**+FGF-2, neurons exhibited the occurrence of spontaneous postsynaptic currents (sPSCs), generated by neurotransmitter release from presynaptic terminals and activation of their receptors expressed on postsynaptic cells.^78^ While neurons treated with FGF-2 alone presented sPSCs of fluctuating amplitude (in average 42 ± 5 pA) at a frequency of 1.23 ± 0.38 Hz, the coapplication of **D2** with the growth factor induced a statistically significant enhancement of both these values (for **D2**+FGF-2, sPSCs frequency: 2.52 ± 0.47 Hz, and sPSCs amplitude: 75 ± 14 pA, *p* < 0.05 FGF-2 vs **D2**+FGF-2, Figure 6E–F).

From the mechanistic insights and biological implications point of view, these findings indicate that **D2** functions as a molecular stabilizer or potentiator of FGF-2 signaling, leading to an enhanced neurotrophic effect of neurite arborization and elongation.^79,80^ The selective enhancement of both neurite branching and elongation suggests that **D2** may extend the bioavailability of FGF-2, facilitate receptor clustering, or modulate downstream signaling cascades.^81–83^ Neurons exhibiting more numerous and longer neurites or presenting modified firing properties could contribute to the formation of a network with enhanced neuronal activity,^84^ in agreement with our electrophysiological recordings showing stronger synaptic communication in **D2**+FGF-2-treated samples compared to those exposed to FGF-2 alone. Given that HS proteoglycans naturally act as growth factor coreceptors, it is likely that **D2** mimics these functions by stabilizing the growth factor-receptor complex or preventing FGF-2 degradation. Due to the fact that **D2** alone had no effect on neuronal morphology, it implies that it acts as a context-dependent modulator, exerting its effects only in the presence of FGF-2. The results of these studies have significant implications for applications *in vivo*. GFs like FGF-2 have extremely short half-lives in physiological environments, causing rapid degradation and limited bioavailability. Regenerative medicine faces this challenge because exogenous administration of GFs fails to achieve sustained therapeutic effects due to their instability.^85,86^ By binding and stabilizing FGF-2, **D2** could prolong its activity *in vivo*, lowering the need for repeated dosing and enhancing therapeutic outcomes. This could be particularly valuable in nerve injury and neurodegenerative disease models, where continuous trophic support is essential for regeneration.

## 3 Conclusions

In summary, we reported a compact, yet structurally focused library of HS glycomimetics featuring systematically varied sulfation patterns (2-*O*, 6-*O*, and *N*-sulfation) prepared using a modular synthetic strategy. Comprehensive biophysical characterizations of molecular protein-sugar interactions revealed that these unique motifs serve as recognition elements for neurotrophic proteins FGF-1, FGF-2, and NGF. BLI experiments showed that our synthetic analogs exhibit selective, sulfation-dependent binding profiles to GFs with dissociation constants in the submicromolar range. CD spectroscopy and thermal denaturation experiments confirmed that the thermal stability of FGF proteins is reinforced upon glycomimetic binding, quantified by the increment in melting temperatures by up to 8.5 °C. Molecular modeling corroborated the experimental binding profiles and disclosed defined interactions of HS glycomimetics with the target proteins. Importantly, the absence of Factor IIa and Xa inhibition in chromogenic assays, together with unaltered aPTT values, confirms their hemocompatibility. We assessed the neuritogenic activity of our lead glycomimetic using neuronal models, PC12 and SH-SY5Y cells. In both cell lines, immobilized glycomimetic significantly enhanced NGF-driven neurite outgrowth and neuronal maturation as demonstrated by increased neurite length and greater structural complexity comparable to native HS. These quantitative results confirm the glycomimetic’s ability to mimic ECM-bound HS and effectively modulate neurotrophic signaling. Extending these findings to a more translationally relevant model, we also evaluated rat hippocampal neurons, where the glycomimetic potentiated FGF-2-mediated neurite outgrowth and supported neural network formation with enhanced synaptic activity, effects that significantly surpassed those induced by FGF-2 alone, thereby coupling molecular recognition with favorable functional cellular outcomes. Overall, our multiscale investigation, spanning from molecular to cellular levels, positions HS glycomimetics as a versatile platform for accomplishing orthogonal regulation of GF signaling and maintaining hemostatic balance. This framework can be adapted to target multiple GFs, paving the way for advancements across diverse regenerative medicine applications.

## Supplementary Material



## Notes

Experimental details, materials, methods, detailed synthetic procedures for HS glycomimetics, additional figures including molecular modeling, CD spectra, BLI analysis, in vitro experiments with primary neurons, and the spectral data of compounds (^1^H NMR,^13^C NMR, 2D NMR, ESI-MS) (PDF)

## Abbreviations

AgOTf: silver trifluoromethanesulfonate
aPTT: activated partial thromboplastin time
AT-III: antithrombin III
BLI: biolayer interferometry
CD: circular dichroism
ECM: extracellular matrix
FIIa: Factor IIa (thrombin)
FXa: Factor Xa
FGF-1: fibroblast growth factor-1, acidic FGF
FGF-2: fibroblast growth factor-2, basic FGF
FGFR: FGF receptors
GAG: glycosaminoglycan
GFs: growth factors
Glc: glucose
GlcA: glucuronic acid
GlcN: glucosamine
GlcNAc: *N*-acetyl-glucosamine
HS: heparin/heparan sulfate
IC_50_(*t*): the apparent half-maximal inhibitory concentration
IdoA: l-iduronic acid
*K*_D_: equilibrium dissociation constant
MRE: mean residue ellipticity
NGF: β-nerve growth factor protein
NIS: *N*-Iodosuccinimide
sPSCs: spontaneous postsynaptic currents
*T*_m_: melting temperature
UFH: unfractionated heparin
λ: wavelength
