# Supplementary material for: Unraveling the Potential of Small Molecule Heparin Glycomimetics in Neuroregenerative Therapeutics
Source: J Am Chem Soc. 2025 Dec 8;147(50):46023–38. doi: 10.1021/jacs.5c13142 (PMC12715788; doi:10.1021/jacs.5c13142)

Supporting Information (SI) for
Unraveling the Potential of Small Molecule Heparin Glycomimetics in Neuroregenerative Therapeutics

Melis Özkan[1,2]*, Giada Cellot[3,9], Sujeet Pawar[1,9], Deepika Sardana[1], Ivana Barravecchia[4], Laura Ballerini[3], Debora Angeloni[4,5], Silvestro Micera[2,4,6,7]*, Francesco Stellacci[1,7,8]*

[1]Institute of Materials, École Polytechnique Fédérale de Lausanne (EPFL), 1015, Lausanne, Switzerland.

[2]Bertarelli Foundation Chair in Translational Neural Engineering, Center for Neuroprosthetics, École Polytechnique Fédérale de Lausanne (EPFL),1015, Lausanne, Switzerland.

[3]Neuron Physiology and Technology Laboratory, Neuroscience Area, International School for Advanced Studies (SISSA), Via Bonomea 265, 34136, Trieste, Italy.

[4]The Institute of Biorobotics, Scuola Superiore Sant'Anna, Piazza Martiri della Libertà 33, 56127, Pisa, Italy.

[5]Health Science Interdisciplinary Center, Scuola Superiore Sant'Anna, Via G. Moruzzi, 1, 56124, Pisa, Italy.

[6]Department of Excellence in Robotics & AI, Scuola Superiore Sant'Anna, Piazza Martiri della Libertà 33, 56127, Pisa, Italy.

[7]Institute of Bioengineering, École Polytechnique Fédérale de Lausanne (EPFL), 1015, Lausanne, Switzerland.

[8]Global Health Institute, École Polytechnique Fédérale de Lausanne (EPFL), 1015, Lausanne, Switzerland.

[9]Giada Cellot and Sujeet Pawar contributed equally to this work and are joint second authors.

*To whom correspondence should be addressed.

Email addresses: melis.ozkan@epfl.ch, silvestro.micera@epfl.ch, francesco.stellacci@epfl.ch.

Table of Contents

Abbreviations

1. Chemical Syntheses

1.1. General Materials and Methods for Synthetic Procedures

1.2. Synthesis of Small Molecule Heparin Glycomimetics

2. Molecular Modeling

3. Circular Dichroism (CD) Experiments

4. Biolayer Interferometry (BLI) Experiments

4.1. Binding Assays

4.2. Competition Assays

5. Determination of the Anticoagulant Activity of Sugars

5.1. AT-III-Mediated Inhibition of FXa and FIIa

5.2. Activated Partial Thromboplastin Time (aPTT) Measurements

6. Testing HS Glycomimetics on Neural Cell Lines

6.1. Neural Cell Culture and Differentiation

6.1.1. PC12 Cells

6.1.2. SH-SY5Y Cells

6.2. Treatment of Cells on Glycomimetic Coated Surfaces

6.3. Immunofluorescence Labelling

6.4. Image Analysis

7. Testing HS Glycomimetics on Dissociated Hippocampal Cultures

7.1. Preparation of Dissociated Hippocampal Cultures

7.2. Treatment of Dissociated Hippocampal Cultures

7.3. Immunofluorescence Labelling

7.4. Electrophysiological recordings and analysis

7.5. Statistical Analysis

8. References

9. Spectral Data

Abbreviations

$(NH_4)_4Ce(SO_4)_4.2H_2O$, cerium ammonium sulfate dihydrate; **$(NH_4)_6Mo_7O_{24}.4H_2O$**, ammonium heptamolybdate tetrahydrate; **Ac**, acetyl; **Ac_2O**, acetic anhydride; **AcCl**, acetyl chloride; **AcOH**, acetic acid; **AgOTf**, silver trifluoromethanesulfonate; **$AlCl_3$**, aluminium chloride; **APCI,** atmospheric pressure chemical ionization; **aPTT**, activated partial thromboplastin time; **AT-III**, antithrombin III; **BAIB**, (diacetoxyiodo)benzene; **$BF_3.Et_2O$**, boron trifluoride diethyl etherate; **BLI**, biolayer interferometry; **Bn**, benzyl; **BnBr**, benzyl bromide; **Bu**, butyl; **Bu_2SnO**, dibutyltin(IV) oxide; **Bz**, benzoyl; **BzCl**, benzoyl chloride; **$CaCl_2$**, calcium chloride; **Cbz**, benzyloxycarbonyl; **CCl_3CN**, trichloroacetonitrile; **CD**, circular dichroism; **$CDCl_3$**, deuterated chloroform; **$CHCl_3$**, chloroform; **CMPI,** 2-Chloro-1-methylpyridinium iodide; **COSY**, homonuclear correlation spectroscopy; **CSA**, camphor-10-sulfonic acid; **CsF,** cesium fluoride; **D_2O**, deuterated water; **DABCO,** 1,4-Diazabicyclo[2.2.2]octane; **DBU**, 1,8-Diazabicyclo[5.4.0]undec-7-ene; **DCM**, dichloromethane; **DDQ**, 2,3-dichloro-5,6-dicyano-*p*-benzoquinone; **DIV**, days *in vitro*; **DMAP**, 4-Dimethylaminopyridine; **DMF**, *N*,*N*-dimethyl formamide; **DMSO**, dimethyl sulfoxide; **EDC**, 1-(3-dimethylaminopropyl)-ethylcarbodiimide; **Et**, ethyl; **Et_2O**, diethyl ether; **Et_3N**, triethylamine; **Et_3SiH**, triethylsilane; **EtOAc**, ethyl acetate; **EtOH**, ethanol; **EtSH**, ethanethiol; **ESI**, electrospray ionization; **FBS**, fetal bovine serum; **FGF-1**, fibroblast growth factor-1, acidic FGF; **FGF-2**, fibroblast growth factor-2, basic FGF; **FIIa**, Factor IIa (thrombin); **FXa**, Factor Xa; **Fmoc**, fluorenylmethoxycarbonyl; **GAG**, glycosaminoglycan; **GlcNAc**, *N*-acetyl-D-glucosamine; **H_2O**, water; **H_2O_2**, hydrogen peroxide; **H_2SO_4**, sulfuric acid; **$HC(OMe)_3$**, trimethyl orthoformate; **HCl**, hydrochloric acid; **Hex**, Hexane; **HMBC**, heteronuclear multiple bond correlation spectroscopy; **HRMS**, high resolution mass spectrometry; **HS**, heparin sulfate; **HSQC**, heteronuclear single quantum coherence spectroscopy; **IdoA**, L-Iduronic acid; **$KHCO_3$**, potassium bicarbonate; **KOH**, potassium hydroxide; **KO*t*Bu**, potassium tert-butoxide; **Lev**, levulinoyl; **LiOH**, lithium hydroxide; **Me**, methyl; **$Me_3N.BH_3$**, borane trimethylamine complex; **MeCN**, acetonitrile; **MeI**, Iodomethane; **$MeOCH_2CH_2OH$**, 2-Methoxyethanol; **MeOD**, deuterated methanol; **MeOH**, methanol; **MES**, (2-(N-morpholino)ethanesulfonic acid); **$MgSO_4$**, magnesium sulfate; **MRE**, mean residue ellipticity; **MS,** mesyl; **MsCl**, methanesulfonyl chloride; **MW**, molecular weight; **$N_2H_4.HOAc$,** hydrazine acetate; **N_3**, azide; **$Na_2S_2O_3$**, sodium thiosulfate; **Na_2SO_4**, sodium sulfate; **$NaBH_3CN$**, sodium cyanoborohydride; **NaH**, sodium hydride; **$NaHCO_3$**, sodium bicarbonate; **NaN_3**, sodium azide; **NaOH**, sodium hydroxide; **NaOMe**, sodium methoxide; **Nap**, 2-naphthylmethyl ether; **$NapCH(OMe)_2$**, 2-(Dimethoxymethyl)naphthalene; **NGF**, β-nerve growth factor protein; **NHS**, *N*-Hydroxysuccinimide; **NIS**, *N*-Iodosuccinimide; **NMR**, nuclear magnetic resonance; **PBS**, Phosphate-buffered saline; **$Pd(OH)_2/C$**, palladium hydroxide over carbon; **PDL,** poly-D-lysine; **PFA,** Paraformaldehyde; **Ph**, phenyl; **$PhBCl_2$**, dichlorophenylborane; **$PhCH(OMe)_2$**,

benzaldehyde dimethyl acetal; **PMe_3**, trimethylphosphine; **PPh_3**, triphenylphosphine; **ppm**, parts per million; ***p*TsOH**, *p*-Toluenesulfonic acid; **Py**, pyridine; **Q-TOF**, quadruple-time-of-flight; **RMSD**, root-mean-square deviation; **rpm**, rotation per minute; **R.T.**, room temperature; **SEM,** standard error of the mean; **SEt**, thioethyl; **$SnCl_4$**, tin(IV) chloride; **$SO_3.Et_3N$**, sulfur trioxide/ triethylamine complex; **SO_3.Py**, sulfur trioxide/pyridine complex; ***t*-BuOH**, tert-butyl alcohol; **TBS**, tris-buffered saline; **TEMPO**, 2,2,6,6-tetramethylpiperidine 1-oxyl; **TFA**, trifluoroacid; **THF**, tetrahydrofuran; **TLC**, thin layer chromatography; **T_m**, melting temperature; **TMS**, tetramethylsilane; **TMSOTf**, trimethylsilyl trifluoromethanesulfonate; **Tol**, toluene; **TrocCl**, 2,2,2-trichloroethoxycarbonyl chloride; **Ts**, tosyl; **TsCl**, *p*-toluenesulfonyl chloride; **UFH**, unfractionated heparin; **v/v**, volume/volume, **wt.%,** weight percentage; **λ,** wavelength.

1. Experimental Section

1.1. General Materials and Methods for Synthetic Procedures

The reactions were carried out in oven-dried glassware under an argon atmosphere using anhydrous solvents, unless otherwise stated. H_2O was obtained using the Milli-Q® EQ 7000 purification system (resistivity 18.2 MΩ.cm at 25 °C). Molecular sieves (3 or 4 Å, 8 to 12 mesh size) for the reactions were pre-activated by crushing finely under high vacuum at 300 °C overnight initially, followed by an additional 3 hours at 300 °C right before use. All other commercial reagents were used as received unless otherwise specified. The reactions requiring elevated temperatures were set on DrySyn® Prodigy oil-free heating systems (Asynt Ltd., the UK). All the reactions were monitored by TLC using silica gel 60 F_{254} pre-coated plates (0.25 mm) by Merck. Spots were visualized by UV or the Hanessian's stain, a solution of $(NH_4)_6Mo_7O_{24}.4H_2O$ (12.5 g, 10.1 mmol) and $(NH_4)_4Ce(SO_4)_4.2H_2O$ (5.0 g, 7.9 mmol) in 10% of 500 mL H_2SO_4, followed by charring at around 200 °C. SiliCycle™ SiliaFlash™ Irregular Silica Gel, P60, 40 to 63 μm, 60 Å was used for column chromatography. Sephadex® LH-20 (140-550 mesh) or Bio-Gel P-2 Gel (Cat. #1504118), Bio-Rad, extra fine polyacrylamide beads (<45 μm wet bead size, 100-1800 MW fractionation range), were used for the size exclusion chromatography. Fractions or reaction mixtures containing the sulfated compound were concentrated under reduced pressure, ensuring the water bath temperature of the rotary evaporator was kept below 25 °C, otherwise, below 40 °C. All the compounds were thoroughly dried under a Schlenk line before the next steps. NMR spectra (^1H, ^{13}C, COSY, HSQC, HMBC) were recorded by Bruker Avance NEO 400 MHz or Bruker Avance III™ HD 600 MHz spectrometer at 25 °C. Chemical shifts were reported in parts per million (ppm) relative to TMS (δ = 0 ppm) or D_2O (δ = 4.79 ppm) as internal standards. The NMR data is presented in the following format: chemical shift, multiplicity (s = singlet, d = doublet, t = triplet, dd = doublet of

doublets, m = multiplet and/or multiple resonances), coupling constant in Hertz (Hz), and integration. HRMS was performed on the 6530 Accurate-Mass Q-TOF LC/MS (Agilent Technologies) or the Thermo Scientific™ Orbitrap Exploris™ 240 mass spectrometer equipped with the Triversa NanoMate® nanochip-based ESI ion source.

1.2. Synthesis of Small Molecule Heparin Glycomimetics

1.2.1. Synthesis of GlcNAc(6S)O$(CH_2)_2NH_2$ (M1)

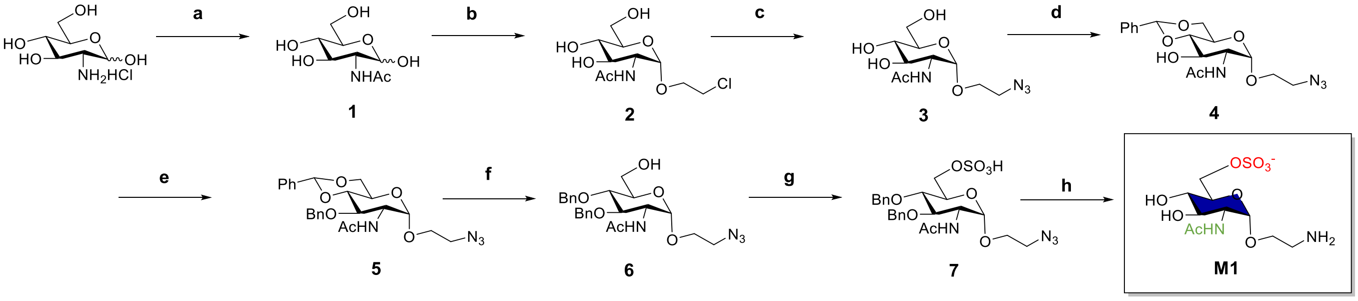

Scheme S1. Synthetic route for M1. Reagents and conditions: **(a):** Et_3N, AcCl, , MeOH, R.T., 12h, 85%; **(b):** 2-chloroethanol, AcCl, 70 °C - R.T., 8h, 60%; **(c):** NaN_3, DMF, 60 °C, 12h, 62%; **(d):** $PhCH(OMe)_2$, *p*TsOH, DMF, 60 °C, 12h, 80%; **(e):** NaH (60% dispersion in mineral oil), BnBr, DMF, THF, 0 °C - R.T. 1h, under N_2, 86%; **(f):** Et_3SiH, $PhBCl_2$, DCM, molecular sieves (4 Å), -78 °C, 15 min, 68%; **(g):** SO_3.Py, DMF, 60 °C, 12h, 78%; **(h):** $Pd(OH)_2$/C, MeOH/H_2O, under H_2, R.T., 24h, 51%.

1.2.1.1. Synthesis of 2-acetamido-2-deoxy-α-D-glucopyranoside (1):

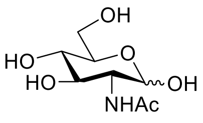

1

GlcNAc was obtained from the *N*-acetylation reaction. D-(+)-Glucosamine hydrochloride (20 g, 92.8 mmol) was dissolved in anhydrous MeOH (200 mL) under argon. The solution was neutralized by the addition of Et_3N (12.9 mL, 92.8 mmol) and cooled down to 0 °C, followed by the dropwise addition of AcCl (7.9 mL, 111.4 mmol), and stirred for a few hours in the ice bath. The reaction was allowed to warm to R.T. and stirred overnight. H_2O was added and stirred for an additional half an hour, followed by filtration over sintered glass. Solvents were removed under reduced pressure to afford compound **1** (17.4 g, 78.8 mmol, 85%) as a white solid.

1.2.1.2. Synthesis of 2-chloroethyl-2-acetamido-2-deoxy-α-D-glucopyranoside (2):

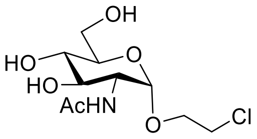

2

For the linker attachment reaction, the solution of compound **1** (17.4 g, 78.8 mmol) in 2-chloroethanol (145.4 mL, 2.2 mmol) was cooled down to 0 °C, and AcCl was added to the solution dropwise (6.8 mL, 94.6 mmol) under an argon atmosphere. The reaction mixture was heated to 70 °C for 4 hours, then stirred at R.T. for an additional 4 hours. The solution was concentrated under reduced pressure, and the resulting brownish oily crude was dissolved in EtOH. After decolorization with charcoal and filtration, the residue was

concentrated and purified by silica gel chromatography (MeOH/DCM, 0 to 15%, v/v) to yield the pale-yellow solid compound **2** (13.4 g, 47.2 mmol, 60%).

^{1}H-NMR (400 MHz, D_2O) δ 4.87 (d, *J* = 3.6 Hz, 1H), 3.96 – 3.65 (m, 9H), 3.47 – 3.34 (m, 1H), 1.97 (s, 3H).

^{13}C-NMR (101 MHz, D_2O) δ 174.44, 97.09, 72.04, 70.88, 69.95, 68.23, 60.53, 53.64, 43.57, 21.91.

HRMS (ESI/QTOF) m/z: $[M + Na]^+$ Calculated for $C_{10}H_{18}ClNNaO_6^+$ 306.0715; Found 306.0718.

1.2.1.3. Synthesis of 2-azidoethyl-2-acetamido-2-deoxy-α-D-glucopyranoside (3):

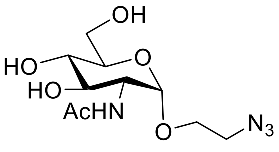

3

For chloro-to-azido conversion, compound **2** (13.4 g, 47.2 mmol) was dissolved in anhydrous DMF (150 mL) under argon. NaN_3 (6.1 g, 94.4 mmol) was added to the reaction solution and then heated to 60 °C for 12 hours. After the completion, the reaction mixture was washed with ice-cold H_2O. The aqueous layer was extracted with EtOAc (3 x 100 mL). The organic layer was dried over Na_2SO_4, filtered, and concentrated under reduced pressure. The residue was purified by column chromatography on silica gel (MeOH/DCM, 0 to 15%, v/v) to yield white solid azido compound **3** (8.5 g, 29.3 mmol, 62%).

^{1}H-NMR (400 MHz, MeOD-d4) δ 4.77 (d, *J* = 3.6 Hz, 2H), 3.89 – 3.79 (m, 1H), 3.82 – 3.71 (m, 3H), 3.72 (d, *J* = 2.3 Hz, 1H), 3.68 – 3.44 (m, 6H), 3.36 (t, *J* = 4.9 Hz, 3H), 3.27 (dd, *J* = 9.8, 8.8 Hz, 2H), 2.02 – 1.93 (m, 1H), 1.89 (s, 5H).

^{13}C-NMR (101 MHz, MeOD-d4) δ 172.35, 97.58, 72.69, 71.27, 71.24, 70.83, 68.16, 66.38, 61.27, 53.99, 50.34, 48.25, 42.67, 21.27, 19.92.

HRMS (ESI/QTOF) m/z: $[M + Na]^+$ Calculated for $C_{10}H_{18}N_4NaO_6^+$ 313.1119; Found 313.1128.

1.2.1.4. Synthesis of 2-azidoethyl-4,6-*O*-benzylidene-2-acetamido-2-deoxy-α-D-glucopyranoside (4):

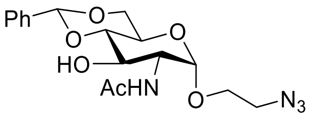

4

Compound **3** (8.5 g, 29.3 mmol) was dissolved in anhydrous DMF (90 mL) under an argon atmosphere. $PhCH(OMe)_2$ (8.9 g, 58.6 mmol) and *p*TsOH monohydrate (285.3 mg, 1.5 mmol) were added to the reaction solution under an argon environment and stirred at 60 °C for 12 hours. After the completion, the reaction was quenched with a few drops of Et_3N. The reaction mixture was washed with ice-cold H_2O, and the aqueous layer was extracted with EtOAc (3 x 100 mL). The organic layer was dried over Na_2SO_4, filtered, and concentrated under reduced pressure. The residue was purified by

column chromatography on silica gel (MeOH/DCM, 0 to 5%, v/v) to yield white solid azido compound **4** (8.0 g, 21.1 mmol, 80%).

^{1}H-NMR (400 MHz, $CDCl_3$) δ 7.51 (dq, *J* = 5.5, 2.8 Hz, 6H), 7.38 (dq, *J* = 8.2, 2.7 Hz, 9H), 6.06 (d, *J* = 8.8 Hz, 2H), 5.57 (s, 2H), 4.95 – 4.84 (m, 2H), 4.39 – 4.07 (m, 7H), 4.00 – 3.88 (m, 4H), 3.88 – 3.69 (m, 6H), 3.69 – 3.47 (m, 8H), 3.39 – 3.29 (m, 4H), 2.95 (d, *J* = 2.3 Hz, 1H), 2.92 – 2.87 (m, 1H), 2.17 – 1.87 (m, 11H).

^{13}C-NMR (101 MHz, $CDCl_3$) δ 172.21, 137.01, 129.27, 126.35, , 101.98, 100.74, 98.18, 81.92, 77.36, 71.18, 70.08, 66.36, 62.89, 58.86, 53.87, 50.71, 50.52, 36.51, 23.22.

HRMS (ESI/QTOF) m/z: $[M + H]^+$ Calculated for $C_{17}H_{23}N_4O_6^+$ 379.1612; Found 379.1619.

1.2.1.5. Synthesis of 2-azidoethyl-3-*O*-benzyl-4,6-*O*-benzylidene-2-acetamido-2-deoxy-α-D-glucopyranoside (5):

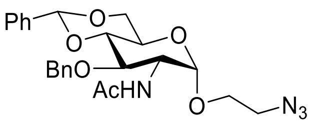

5

Compound **4** (8.0 g, 21.1 mmol) was dissolved in a mixture of anhydrous THF (75 mL) and DMF (15 mL) thoroughly. The solution was cooled down to 0 °C under a nitrogen environment. NaH (2.53 g, 63.3 mmol, 60% dispersion in mineral oil) was added and stirred for half an hour. Then, BnBr (3 mL, 25.3 mmol) was added while stirring, and the ice bath was removed. The reaction was allowed to warm up to R.T. for ~1 hour. After the completion, the reaction was quenched with MeOH, diluted with EtOAc, and washed with cold H_2O. The aqueous layer was extracted with EtOAc (3 x 150 mL). The combined organic layers were dried over Na_2SO_4, filtered, and concentrated under reduced pressure. The residue was purified by column chromatography on silica gel (EtOAc/DCM, 0 to 10%, v/v) to give white solid compound **5** (8.6 g, 18.4 mmol, 86%).

^{1}H-NMR (400 MHz, $CDCl_3$) δ 7.58 – 7.49 (m, 4H), 7.48 – 7.39 (m, 6H), 7.33 (qd, *J* = 6.8, 2.5 Hz, 12H), 5.63 (s, 2H), 5.40 (d, *J* = 9.1 Hz, 2H), 4.98 – 4.83 (m, 4H), 4.66 (d, *J* = 12.3 Hz, 2H), 4.42 – 4.30 (m, 3H), 4.29 (d, *J* = 3.8 Hz, 1H), 3.99 – 3.84 (m, 4H), 3.84 – 3.79 (m, 3H), 3.76 (dd, *J* = 10.2, 8.6 Hz, 2H), 3.62 (ddd, *J* = 10.8, 8.2, 2.8 Hz, 2H), 3.51 (ddd, *J* = 13.4, 8.2, 2.9 Hz, 2H), 3.29 (ddd, *J* = 13.4, 5.3, 2.8 Hz, 2H), 1.92 (s, 5H), 1.28 (s, 8H), 1.10 (s, 1H), 0.94 – 0.83 (m, 6H).

^{13}C-NMR (101 MHz, $CDCl_3$) δ 170.06, 138.32, 137.28, 129.03, 126.01, 101.35, 98.44, 82.63, 77.34, 77.23, 77.02, 76.71, 75.55, 74.16, 68.91, 67.37, 63.18, 52.33, 50.47, 29.71, 23.27.

HRMS (ESI/QTOF) m/z: $[M + H]^+$ Calculated for $C_{24}H_{29}N_4O_6^+$ 469.2082; Found 469.2092.

1.2.1.6. Synthesis of 2-azidoethyl-3,4-*O*-benzyl-2-acetamido-2-deoxy-α-D-glucopyranoside (6):

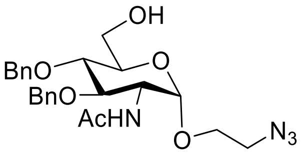

6

Compound **5** (8.6 g, 18.4 mmol) was dissolved in DCM (100 mL) and stirred with pre-activated molecular sieves (27 g) for half an hour. The temperature was brought down to -78 °C, and the mixture was stirred at this temperature for an additional half an hour. Afterward, Et_3SiH (5.9 mL, 36.8 mmol) and $PhBCl_2$ (7.2 mL, 55.2 mmol) were added. The mixture was stirred for 15 min, and it was quenched with Py, followed by dilution with DCM. The molecular sieves were filtered off over a pad of celite, and the mixture was washed with sat. $NaHCO_3$. The organic layer was dried over Na_2SO_4, filtered, and concentrated under reduced pressure. The residue was purified by column chromatography on silica gel (EtOAc/Hex, 0 to 40%, v/v) to give compound **6** (5.9 g, 12.3 mmol, 68%).

^{1}H-NMR (400 MHz, MeOD) δ 7.63 (d, *J* = 7.3 Hz, 1H), 7.52 – 7.45 (m, 2H), 7.28 (d, *J* = 2.4 Hz, 1H), 7.27 – 7.21 (m, 7H), 7.21 – 7.10 (m, 16H), 4.83 (dd, *J* = 23.6, 11.2 Hz, 10H), 4.75 – 4.65 (m, 9H), 4.65 – 4.58 (m, 3H), 4.58 – 4.46 (m, 2H), 4.02 (ddd, *J* = 30.3, 10.6, 3.7 Hz, 2H), 3.91 – 3.43 (m, 16H), 3.43 – 3.23 (m, 5H), 2.08 – 1.99 (m, 1H), 1.90 (d, *J* = 2.9 Hz, 1H), 1.82 (d, *J* = 7.7 Hz, 5H), 1.19 (d, *J* = 3.9 Hz, 1H).

HRMS (ESI/QTOF) m/z: $[M + Na]^+$ Calculated for $C_{24}H_{30}N_4NaO_6^+$ 493.2058; Found 493.2053.

1.2.1.7. Synthesis of 2-azidoethyl-3,4-*O*-benzyl-6-*O*-sulfate-2-acetamido-2-deoxy-α-D-glucopyranoside (7):

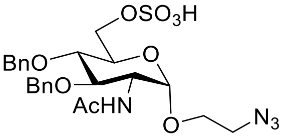

7

For the 6-*O*-sulfation reaction, compound **6** (5.9 g, 12.3 mmol) was dissolved in DMF (60 mL). $SO_3 \cdot Py$ (19.6 g, 123 mmol) was added to the reaction solution and kept at 60 °C for 12h. After the completion, $MeOH/Et_3N$ (50%, v/v) was added and stirred for half an hour. The reaction mixture was filtered, and the solvents were removed under reduced pressure. The residue was purified by column chromatography on silica gel ($MeOH/CHCl_3$, 0 to 20%, v/v) to give the sulfated compound **7** (5.4 g, 9.8 mmol, 78%).

^{1}H-NMR (400 MHz, MeOD) δ 7.41 – 7.34 (m, 1H), 7.34 – 7.23 (m, 3H), 4.87 (s, 5H), 4.81 – 4.69 (m, 1H), 4.37 – 4.22 (m, 1H), 4.00 – 3.78 (m, 1H), 3.70 – 3.60 (m, 1H), 3.51 (t, *J* = 4.9 Hz, 1H), 3.22 (q, *J* = 7.3 Hz, 2H), 1.94 (s, 1H), 1.33 (t, *J* = 7.3 Hz, 3H).

^{13}C-NMR (101 MHz, MeOD) δ 138.70, 138.25, 127.92, 127.90, 127.29, 127.26, 127.16, 97.57, 80.19, 78.14, 74.59, 74.57, 69.95, 66.36, 66.15, 53.04, 50.27, 48.09, 46.56, 21.34, 7.84.

HRMS (ESI/QTOF) m/z: $[M + H]^+$ Calculated for $C_{24}H_{31}N_4O_9S^+$ 551.1806; Found 551.1803.

1.2.1.8. Synthesis of 2-aminoethyl-6-*O*-sulfate-2-acetamido-2-deoxy-α-D-glucopyranoside (M1):

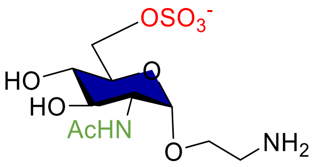

M1

For the debenzylation reaction, compound **7** (5.4 g, 9.8 mmol) and 20% $Pd(OH)_2/C$ (8.1 g, 1.5x of compound 7 by weight) were dispersed in $MeOH/H_2O$ (50%, v/v) under a hydrogen environment and stirred at R.T. for 24 hours. The next day, the reaction mixture was filtered, and the residue was washed with H_2O. After removing water, the crude product was purified by size exclusion chromatography using a P-2 Gel column using Milli-Q H_2O as an eluent. The fractions having the product were freeze-dried to yield the final monosaccharide (**M1**) (1.7 g, 5.0 mmol, 51%).

^{1}H-NMR (400 MHz, D_2O) δ 4.85 (dd, *J* = 7.3, 3.6 Hz, 2H), 4.30 (ddd, *J* = 11.3, 7.6, 2.2 Hz, 2H), 4.15 (ddd, *J* = 11.4, 6.1, 2.1 Hz, 2H), 4.09 – 3.94 (m, 2H), 3.94 – 3.90 (m, 2H), 3.87 (ddt, *J* = 10.0, 5.8, 2.6 Hz, 2H), 3.75 – 3.55 (m, 4H), 3.46 (ddd, *J* = 11.5, 9.0, 2.5 Hz, 2H), 3.41 – 3.30 (m, 1H), 3.22 (t, *J* = 5.1 Hz, 2H), 3.13 (q, *J* = 7.3 Hz, 3H), 1.98 (s, 5H), 1.21 (t, *J* = 7.3 Hz, 6H).

^{13}C-NMR (101 MHz, D_2O) δ 174.41, 98.08, 94.65, 71.06, 70.92, 69.73, 66.84, 65.77, 63.95, 62.84, 58.75, 55.22, 53.47, 53.20, 46.91, 46.67, 39.14, 21.93, 20.58, 14.20, 8.23.

HRMS (ESI/QTOF) m/z: $[M + H]^+$ Calculated for $C_{10}H_{21}N_2O_9S^+$ 345.0962; Found 345.0953.

1.2.2. Synthesis of IdoA(2,4S)-1,4-GlcNAcO(CH_2)$_2$NH$_2$ (D1)

1.2.2.1. Synthesis of Acceptor for D1, 2-azidoethyl-3,6-*O*-benzyl-2-acetamido-2-deoxy-α-D-glucopyranoside (8):

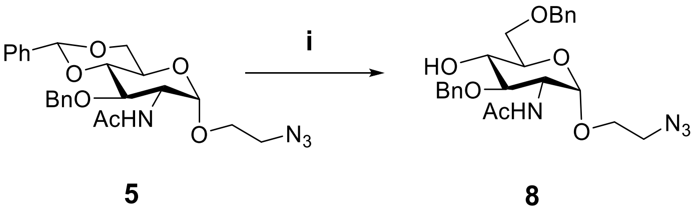

Scheme S2. Synthetic route for the acceptor of **D1**. Reagents and conditions: **(i):** $AlCl_3$, $Me_3N.BH_3$, H_2O, THF, 4 °C - R.T., 12h, 80%.

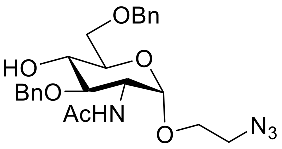

8

For the ring-opening reaction, compound **5** (2.5 g, 5.3 mmol) was mixed with $Me_3N.BH_3$ (1.6 g, 21,2 mmol) in THF (30 mL) and stirred at 4 °C for half an hour under argon. $AlCl_3$ (4.2 g, 31.5 mmol) was dissolved in Et_2O and added to the reaction mixture dropwise at this temperature. Once $AlCl_3$ was completely dissolved, H_2O (193 μL, 10.6 mmol) was added, and the ice bath was removed. The reaction was stirred at R.T. for 12 hours. The reaction was quenched with dilute HCl and washed with brine and water. The

aqueous layer was extracted with EtOAc (3 x 50 mL), dried over Na_2SO_4, filtered, and concentrated under reduced pressure. The residue was purified by column chromatography on silica gel (EtOAc/DCM, 0 to 30%, v/v) to yield compound **8** (2.0 g, 4.3 mmol, 80%).

^{1}H-NMR (400 MHz, $CDCl_3$) δ 7.46 – 7.27 (m, 10H), 5.49 (d, *J* = 9.3 Hz, 1H), 5.32 (s, 1H), 4.94 – 4.85 (m, 1H), 4.76 (s, 1H), 4.72 (d, *J* = 11.8 Hz, 1H), 4.64 (d, *J* = 12.0 Hz, 1H), 4.58 (d, *J* = 12.0 Hz, 1H), 4.29 (ddd, *J* = 10.6, 9.2, 3.6 Hz, 1H), 3.93 (ddd, *J* = 10.7, 5.4, 2.9 Hz, 1H), 3.87 – 3.70 (m, 4H), 3.74 – 3.59 (m, 2H), 3.63 – 3.57 (m, 1H), 3.56 – 3.40 (m, 2H), 3.30 (ddd, *J* = 13.4, 5.4, 2.7 Hz, 1H), 2.73 (s, 1H), 1.91 (s, 3H), 1.96 – 1.85 (m, 1H), 1.80 – 1.67 (m, 2H), 1.65 (s, 1H).

^{13}C-NMR (101 MHz, $CDCl_3$) δ 170.06, 138.33, 126.01, 98.03, 79.59, 77.35, 71.97, 70.62, 67.24, 62.07, 53.44, 51.74, 50.52, 44.92, 29.93, 26.51, 23.33.

HRMS (ESI/QTOF) m/z: $[M + H]^+$ Calculated for $C_{24}H_{31}N_4O_6^+$ 471.2238; Found 471.2239.

1.2.2.2. Synthesis of thioethyl L-idosyl Donor

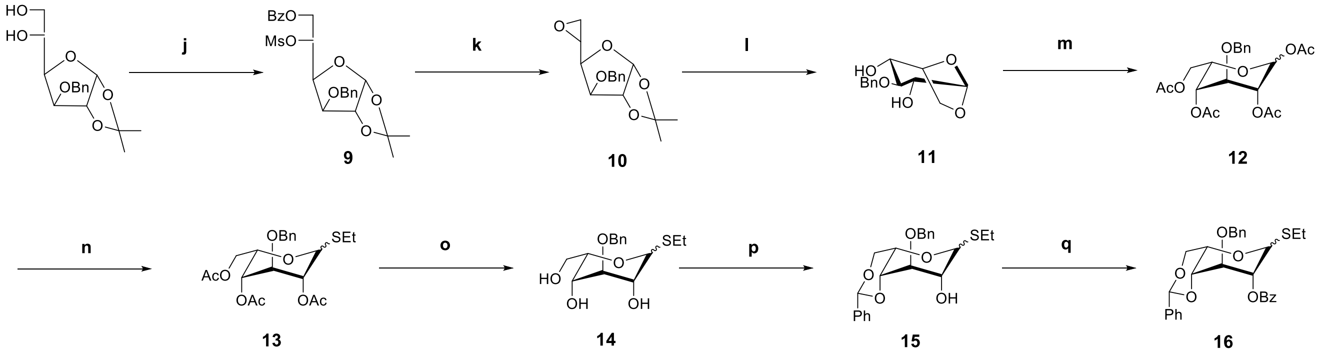

Scheme S3. Synthetic route for the thioethyl L-idosyl donor. Reagents and conditions: **(j): (i):** BzCl, DCM, Py, 0 °C, 2h; **(ii):** MsCl, R.T., 12h, 52%; **(k):** *t*-BuOH, KO*t*Bu, DCM, 0 °C, 16h, 60%; **(l): (i):** 1,4-dioxane, H_2SO_4, reflux, 120 °C, 22h **(ii):** NaOH, 0 °C, 59%; **(m):** Ac_2O, TFA, 0 °C, 24h, 84%; **(n):** $BF_3.Et_2O$, EtSH, DCM, 0 °C - R.T., 2.5h, 60%; **(o):** NaOMe, MeOH/DCM, R.T., 5h, 80%; **(p):** $PhCH(OMe)_2$, CSA, DMF, R.T., 12h, 80%; **(q):** BzCl, Py, 0 °C - R.T., 12h, 82%.

1.2.2.2.1. Synthesis of 6-*O*-Benzoyl-3-*O*-benzyl-1,2-*O*-isopropylidene-5-*O*-mesyl-α-D-glucofuranose (9):

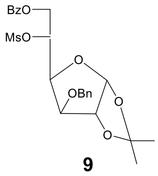

9

A solution of 1,2-*O*-(1-Methylethylidene)-3-*O*-(phenylmethyl)-α-D-glucofuranose (40 g, 128.8 mmol) in DCM (400 mL) was prepared, and Py (100 mL) was added at 0 °C under an argon atmosphere. BzCl (16.6 mL, 142.8 mmol) was then added dropwise to the mixture. After stirring at 0 °C for 2 hours, MsCl (14.8 mL, 192 mmol) was added, and the reaction mixture was allowed to gradually warm to R.T., where it was kept for 18 hours. The reaction was quenched with H_2O (200 mL), and the aqueous layer was extracted with EtOAc (3 x 200 mL). The combined organic layers were washed sequentially with half-saturated $NaHCO_{3(aq)}$, dried over Na_2SO_4,

filtered, and concentrated under reduced pressure. The residue was purified by column chromatography on silica gel (EtOAc/Hex, 0 to 30%, v/v) to yield compound **9** (33.2 g, 52%) as a white solid.

^{1}H-NMR (400 MHz, $CDCl_3$) δ 8.14 – 8.07 (m, 2H), 7.64 – 7.53 (m, 1H), 7.45 (dd, *J* = 20.4, 7.5 Hz, 4H), 7.37 (t, *J* = 7.3 Hz, 2H), 7.35 – 7.28 (m, 1H), 5.95 (d, *J* = 3.6 Hz, 1H), 5.45 (ddd, *J* = 8.1, 6.3, 2.0 Hz, 1H), 4.96 (dd, *J* = 12.7, 2.0 Hz, 1H), 4.74 (d, *J* = 11.0 Hz, 1H), 4.70 – 4.61 (m, 2H), 4.58 – 4.47 (m, 2H), 4.18 (d, *J* = 3.1 Hz, 1H), 3.02 (s, 3H), 1.35 (s, 3H).

^{13}C-NMR (101 MHz, $CDCl_3$) δ 165.98, 137.15, 133.31, 133.15, 132.94, 130.08, 129.75, 112.31, 105.30, 82.17, 78.29, 78.17, 75.35, 72.37, 68.02, 64.10, 60.40, 39.13, 26.87, 26.84, 21.07, 14.23.

HRMS (ESI/QTOF) m/z: $[M+Na]^+$ Calculated for $C_{24}H_{28}NaO_9S^+$ 515.1346; Found 515.1344.

1.2.2.2.2. Synthesis of 3-*O*-Benzyl-5,6-*O*-epoxyl-1,2-*O*-isopropylidene-β-L-idopyranose (10):

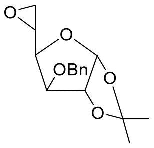

10

Compound **9** (33.2 g, 67.4 mmol) was dissolved in *t*-BuOH (166 mL) and DCM (332 mL). The reaction flask was immersed in an ice bath, and KO*t*Bu (16.6 g, 1 mmol) was added to the mixture. After stirring for 16 hours, H_2O (100 mL) was added to quench the reaction, and the solvent mixture was evaporated under reduced pressure. The residue was diluted with EtOAc (300 mL), and the resulting solution was washed with brine (2 × 200mL). The mixture was dried over $MgSO_4$, filtered, and concentrated in vacuo to give a residue, which was purified by column chromatography on silica gel (EtOAc/Hex, 0 to 25%, v/v) to yield the epoxide compound **10** (11.9 g, 40.7 mmol, 60%).

^{1}H-NMR (400 MHz, $CDCl_3$) δ 7.42 – 7.26 (m, 3H), 6.02 (d, *J* = 3.8 Hz, 1H), 4.76 (d, *J* = 12.2 Hz, 1H), 4.66 (d, *J* = 3.8 Hz, 1H), 4.54 (d, *J* = 12.2 Hz, 1H), 3.99 (d, *J* = 3.6 Hz, 1H), 3.83 (dd, *J* = 6.1, 3.5 Hz, 1H), 3.30 (ddd, *J* = 6.1, 4.3, 2.7 Hz, 1H), 2.82 – 2.75 (m, 1H), 2.56 (dd, *J* = 4.9, 2.7 Hz, 1H), 1.47 (s, 3H), 1.34 (s, 3H).

^{13}C-NMR (101 MHz, $CDCl_3$) δ 171.16, 137.27, 133.26, 130.07, 128.54, 111.95, 105.47, 82.70, 77.38, 76.74, 71.94, 65.34, 60.40, 50.20, 43.19, 26.86, 21.06, 14.21.

HRMS (ESI/QTOF) m/z: $[M + Na]^+$ Calculated for $C_{16}H_{20}NaO_5^+$ 315.1203; Found 315.12100.

1.2.2.2.3. Synthesis of 1,6-Anhydro-3-*O*-benzyl-β-L-idopyranose (11):

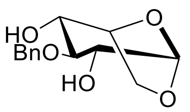

11

Compound **10** (11.9 g, 40.6 mmol) was dissolved in 1,4-dioxane (24 mL), 2N $H_2SO_{4(aq)}$ (24 mL) was added to the solution, and the mixture was refluxed for 22 hours. The reaction flask was immersed in an ice bath, and 3N $NaOH_{(aq)}$ (16 mL) was added to neutralize the solution. H_2O (16 mL) was added, and the crude target material was extracted with EtOAc (3 × 50 mL). The combined organic layers were washed with brine, dried over $MgSO_4$, filtered, and concentrated in vacuo. Purification of the residue through column chromatography (EtOAc/Hex, 50%, v/v) on silica gel provided the 2,4-diol anhydro compound **11** (6.06 g, 24 mmol, 59%).

^{1}H-NMR (400 MHz, $CDCl_3$) δ 9.44 (s, 2H), 7.25 (dddd, *J* = 10.8, 8.0, 6.9, 3.6 Hz, 4H), 7.24 – 7.14 (m, 1H), 7.15 (d, *J* = 3.6 Hz, 2H), 6.45 – 6.38 (m, 2H), 5.19 (d, *J* = 1.9 Hz, 1H), 4.82 (d, *J* = 11.5 Hz, 1H), 4.69 (d, *J* = 11.5 Hz, 1H), 4.58 (s, 4H), 4.40 – 4.28 (m, 1H), 4.06 – 3.93 (m, 1H), 3.80 (ddd, *J* = 8.2, 4.3, 1.1 Hz, 1H), 3.66 – 3.51 (m, 2H), 3.41 (t, *J* = 8.0 Hz, 1H), 2.66 (t, *J* = 6.4 Hz, 6H), 2.55 – 2.47 (m, 6H), 2.10 (s, 9H).

^{13}C-NMR (101 MHz, $CDCl_3$) δ 207.48, 177.96, 161.10, 157.83, 152.15, 140.56, 138.34, 136.51, 130.05, 128.73, 127.04, 112.00, 110.08, 105.33, 89.13, 78.40, 71.03, 66.57, 57.25, 37.74, 29.76, 21.02, 14.11.

HRMS (ESI/QTOF) m/z: $[M + Na]^+$ Calculated for $C_{13}H_{16}NaO_5^+$ 275.0890; Found 275.0892.

1.2.2.2.4. Synthesis of 1,2,4,6-*O*-Tetra-*O*-acetyl-3-*O*-benzyl-α/β-L-idopyranoside (12):

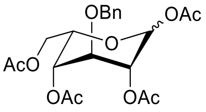

12

To a stirred suspension of 1,6-anhydro sugar compound **11** (6.06 g, 24 mmol) in Ac_2O (120 mL) under argon at 0 °C was added TFA (3 mL). The mixture was then stirred at R.T. for 24 hours, followed by quenching with MeOH (40 mL) and concentrated under reduced pressure. A silica column chromatography (EtOAc/Hex, 50%, v/v) of the crude material afforded tetraacetate compound **12** as a colorless oil (8.84 g, 20.2 mmol, 84%).

^{1}H-NMR (400 MHz, $CDCl_3$) δ 7.44 – 7.36 (m, 1H), 7.36 (q, *J* = 3.1 Hz, 1H), 7.36 – 7.28 (m, 1H), 6.14 – 6.07 (m, 1H), 5.11 – 5.03 (m, 1H), 5.00 – 4.92 (m, 1H), 4.91 – 4.82 (m, 1H), 4.80 – 4.49 (m, 3H), 4.31 – 4.07 (m, 2H), 3.94 – 3.70 (m, 1H), 2.20 – 2.00 (m, 13H).

^{13}C-NMR (101 MHz, $CDCl_3$) δ 175.82, 170.59, 169.89, 138.04, 128.42, 99.21, 91.29, 77.36, 71.69, 66.73, 62.25, 22.18, 20.56, 14.19.

HRMS (ESI/QTOF) m/z: $[M + Na]^+$ Calculated for $C_{21}H_{26}NaO_{10}^+$ 461.1418; Found 461.1425.

1.2.2.2.5. Synthesis of Ethyl2,4,6-Tri-*O*-acetyl-3-*O*-benzyl-1-thio-α/β-L-idopyranoside (13):

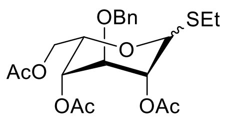

13

$BF_3{\cdot}Et_2O$ (8.6 mL, 307.2 mmol) and EtSH (1.9 mL, 24 mmol) were added to the solution of compound **12** (8.84 g, 20.2 mmol) in anhydrous DCM (400 mL) at 0 °C under argon. The reaction mixture was then stirred at this temperature for 2.5 hours, washed with saturated $NaHCO_3$, dried over $MgSO_4$, and concentrated under reduced pressure. The crude material obtained was purified by silica column chromatography (EtOAc/Hex, 30%, v/v) to yield the thioglycoside compound **13** (5.3 g, 12.0 mmol, 60%) as a pale-yellow syrup.

^{1}H-NMR (400 MHz, $CDCl_3$) δ 7.38 (ddt, *J* = 12.0, 6.1, 4.9 Hz, 6H), 7.36 – 7.24 (m, 2H), 5.32 (d, *J* = 1.3 Hz, 1H), 5.11 – 4.97 (m, 2H), 4.87 (ddd, *J* = 6.5, 5.0, 2.3 Hz, 3H), 4.87 – 4.72 (m, 2H), 4.75 – 4.57 (m, 2H), 4.31 – 4.09 (m, 4H), 3.94 – 3.81 (m, 1H), 3.73 (td, *J* = 2.7, 1.3 Hz, 1H), 2.82 – 2.55 (m, 3H), 2.17 – 2.00 (m, 17H), 1.30 (dt, *J* = 23.5, 7.3 Hz, 5H).

^{13}C-NMR (101 MHz, $CDCl_3$) δ 197.27, 171.15, 168.58, 138.05, 99.21, 90.15, 82.39, 74.43, 71.71, 69.17, 60.39, 47.89, 27.59, 21.05, 21.01, 15.11.

HRMS (ESI/QTOF) m/z: $[M + Na]^+$ Calculated for $C_{21}H_{28}NaO_8S^+$ 463.1397; Found 463.1409.

1.2.2.2.6. Synthesis of Ethyl-3-*O*-benzyl-1-thio-α/β-L-idopyranoside (14):

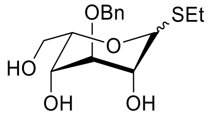

14

For the de-*O*-acetylation reaction, to a solution of compound **13** (5.3 g, 12.0 mmol) in MeOH/DCM (3/1, v/v), NaOMe (130 mg, 2.4 mmol) was added under argon. The reaction mixture was stirred for 5 hours at R.T. and quenched with Amberlite® IR 120 (H^+ form) till the pH was around 7. The mixture was filtered through a pad of celite to remove the resin. The filtrate was concentrated under reduced pressure. The residue was purified by silica column chromatography (EtOAc/Hex, 50%, v/v) to give the deacetylated compound **14** (3.03 g, 9.6 mmol, 80%).

^{1}H-NMR (400 MHz, $CDCl_3$) δ 7.43 – 7.27 (m, 6H), 5.34 (s, 1H), 4.80 – 4.67 (m, 2H), 4.61 – 4.49 (m, 2H), 4.42 (dt, *J* = 3.6, 2.3 Hz, 1H), 4.09 – 3.96 (m, 2H), 4.00 – 3.91 (m, 2H), 3.91 – 3.78 (m, 1H), 3.76 – 3.61 (m, 1H), 3.25 (dt, *J* = 28.0, 6.1 Hz, 1H), 2.83 – 2.57 (m, 2H), 1.32 (td, *J* = 7.4, 2.9 Hz, 3H).

^{13}C-NMR (101 MHz, $CDCl_3$) δ 138.39, 137.59, 137.50, 128.65, 101.86, 86.85, 84.04, 83.87, 77.41, 76.78, 75.51, 74.53, 72.33, 72.09, 65.79, 64.48, 27.00, 25.34, 15.25, 15.20.

HRMS (ESI/QTOF) m/z: $[M + Na]^+$ Calculated for $C_{15}H_{22}NaO_5S^+$ 337.1080; Found 337.1087.

1.2.2.2.7. Synthesis of Ethyl-3-*O*-benzyl-4,6-*O*-benzylidene-1-thio-α/β-L-idopyranoside (15):

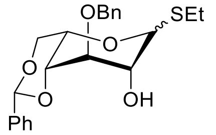

15

For the benzylidene protection reaction, to a solution of compound **14** (3.03 g, 9.6 mmol) in DMF (50 mL), $PhCH(OMe)_2$ (7.3 g, 48 mmol) and CSA (2.23 g, 9.6 mmol) were added under an argon environment. The reaction mixture was stirred at R.T. for 12 hours. The complete reaction was quenched by a few drops of Et_3N and washed with ice-cold H_2O. The aqueous layer was extracted with EtOAc (3 x 50 mL). The organic layer was dried over Na_2SO_4, filtered, and concentrated under reduced pressure. The residue was purified by column chromatography on silica gel (EtOAc/Hex, 0 to 50%, v/v) to yield benzylidene compound **15** (3.1 g, 7.7 mmol, 80%).

^{1}H-NMR (400 MHz, $CDCl_3$) δ 10.06 (s, 1H), 7.95 – 7.88 (m, 2H), 7.71 – 7.63 (m, 1H), 7.57 (dd, *J* = 8.3, 7.0 Hz, 2H), 7.53 – 7.39 (m, 9H), 7.38 (pd, *J* = 5.4, 2.0 Hz, 12H), 7.33 (s, 1H), 5.55 (s, 2H), 5.41 (s, 2H), 4.80 (d, *J* = 11.9 Hz, 2H), 4.75 – 4.61 (m, 1H), 4.58 (d, *J* = 11.9 Hz, 2H), 4.44 – 4.30 (m, 3H), 4.31 (d, *J* = 1.7 Hz, 1H), 4.18 – 4.02 (m, 5H), 4.00 – 3.92 (m, 2H), 3.90 – 3.76 (m, 1H), 3.77 (d, *J* = 2.9 Hz, 2H), 2.86 – 2.60 (m, 5H), 1.35 (t, *J* = 7.4 Hz, 7H).

^{13}C-NMR (101 MHz, $CDCl_3$) δ 192.40, 137.41, 129.76, 129.27, 129.01, 125.98, 101.58, 86.14, 82.97, 77.34, 72.22, 70.18, 68.85, 27.10, 25.28, 15.09.

HRMS (ESI/QTOF) m/z: $[M + Na]^+$ Calculated for $C_{22}H_{26}NaO_5S^+$ 425.1393; Found 425.1391.

1.2.2.2.8. Synthesis of Ethyl-2-*O*-benzoyl-3-*O*-benzyl-4,6-*O*-benzylidene-1-thio-L-idopyranoside (16):

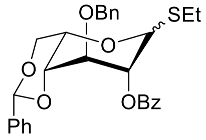

16

For the conversion of -OH to -OBz, compound **15** (3.1 g, 7.7 mmol) was dissolved in Py (35 mL). BzCl (1.79 mL, 15.4 mmol) was added to the solution of compound **15** at 0 °C. Then, the reaction mixture was allowed to gradually warm to R.T., where it was kept for 12 hours. After the reaction was complete, the mixture was diluted with EtOAc, washed with H_2O. The aqueous layer was extracted with EtOAc (3 x 50 mL). The organic layer was dried over Na_2SO_4, filtered, and concentrated *in vacuo*. The residue was purified by column chromatography on silica gel (EtOAc/Hex, 0 to 20%, v/v) to yield benzoyl protected compound **16** (3.2 g, 6.3 mmol, 82%).

^{1}H-NMR (400 MHz, $CDCl_3$) δ 8.03 – 7.96 (m, 2H), 7.56 – 7.28 (m, 10H), 7.28 – 7.20 (m, 2H), 5.59 (d, *J* = 5.8 Hz, 2H), 5.38 (dd, *J* = 2.5, 1.3 Hz, 1H), 4.93 (d, *J* = 11.8 Hz, 1H), 4.67 (d, *J* = 11.8 Hz, 1H), 4.42 (s, 1H), 4.37 (d, *J* = 12.6 Hz, 1H), 4.20 (dd, *J* = 12.5, 1.9 Hz, 1H), 4.09 (s, 1H), 3.87 (s, 1H), 2.82 – 2.62 (m, 2H), 1.36 (t, *J* = 7.4 Hz, 3H).

^{13}C-NMR (101 MHz, $CDCl_3$) δ 132.99, 130.24, 128.80, , 126.45, 101.15, 80.53, 77.33, 74.26, 72.74, 68.51, 25.56, 14.87.

HRMS (ESI/QTOF) m/z: $[M + Na]^+$ Calculated for $C_{29}H_{30}NaO_6S^+$ 529.1655; Found 529.1659.

1.2.2.3. Synthesis of D1

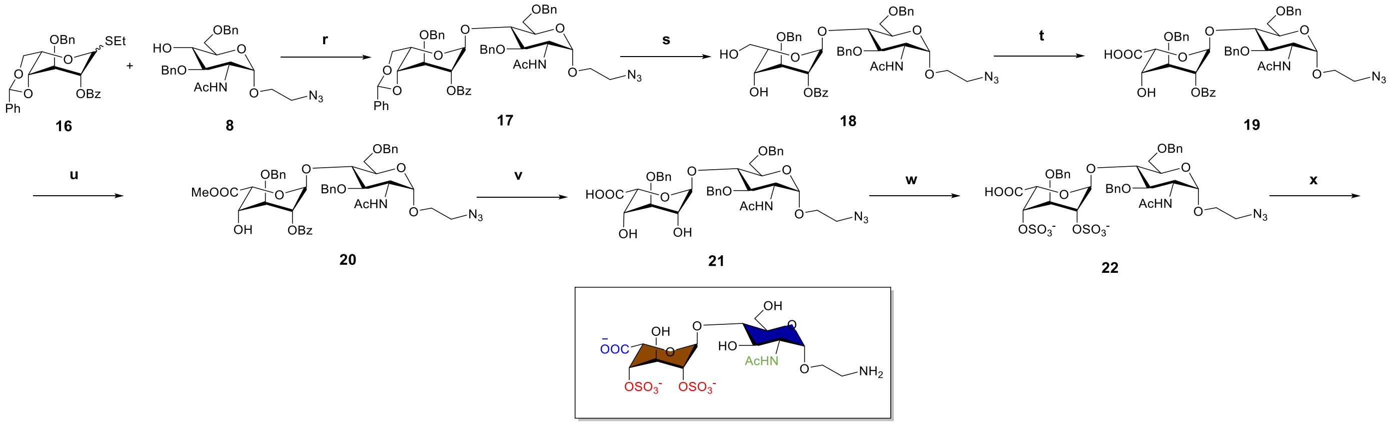

D1

Scheme S4. Synthetic route for **D1**. Reagents and conditions: **(r):** AgOTf, NIS, molecular sieves 4Å, DCM, 0 °C, 30 min, 63%; **(s):** TFA/H_2O, DCM, R.T., 4h, 43%; **(t):** TEMPO, BAIB, DCM/H_2O, R.T., 6h; **(u):** MeI, $KHCO_3$, DMF, R.T., 12h, 51%; **(v):** LiOH, H_2O_2, THF, 40°C, 12h; **(w):** $SO_3.EtN_3$, DMF, 12h, 70%; **(x):** $Pd(OH)_2/C$, MeOH:H_2O, under H_2, R.T., 24h, 54%.

1.2.2.3.1. Synthesis of 2-azidoethyl-*O*-(2-*O*-benzoyl-3-*O*-benzyl-4,6-*O*-benzylidene-α-L-idopyranosyl)-(1→4)-*O*-2-acetamido-3,6-*O*-benzyl-2-deoxy-α-D-glucopyranoside (17):

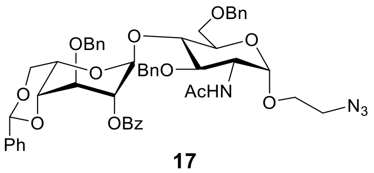

17

For the glycosylation reaction, thioethyl L-idosyl donor **16** (5 g, 9.9 mmol) and GlcNAc acceptor **8** (2.8 g, 5.9 mmol) were dissolved in anhydrous DCM (100 mL) and stirred with pre-activated molecular sieves (10 g) for 1 hour at R.T. under an argon environment. The reaction mixture was then cooled down to 0 °C, followed by the addition of promoters, NIS (4.5 g, 19.8 mmol) and AgOTf (254.3 mg, 0.99 mmol). The reaction mixture was stirred at 0 °C for half an hour. After the completion, it was quenched with Et_3N (~1 mL). The mixture was diluted with DCM and washed with $Na_2S_2O_{3(aq)}$ to decolorize, brine, and H_2O. The organic phase was dried over $MgSO_4$, filtered, and concentrated under reduced pressure. The residue was purified by silica gel column chromatography (EtOAc/Hex, 0 to 70%, v/v) to yield the compound **17** (5.7 g, 6.2 mmol, 63%).

^{1}H-NMR (400 MHz, $CDCl_3$) δ 7.90 – 7.83 (m, 2H), 7.49 – 7.36 (m, 3H), 7.39 – 7.03 (m, 17H), 5.51 – 5.35 (m, 1H), 5.30 (s, 1H), 5.17 (dd, *J* = 3.2, 1.7 Hz, 1H), 5.11 (s, 1H), 4.91 – 4.71 (m, 2H), 4.71 – 4.57 (m, 1H), 4.60 – 4.47 (m, 2H), 4.44 (s, 2H), 4.40 – 4.30 (m, 1H), 4.07 – 3.95 (m, 2H), 3.84 (dq, *J* = 13.5, 2.2 Hz, 3H), 3.82 – 3.48 (m, 6H), 3.42 (tdd, *J* = 14.8, 8.0, 2.8 Hz, 1H), 3.27 – 3.09 (m, 2H), 2.63 (s, 1H), 1.79 (d, *J* = 12.9 Hz, 3H).

^{13}C-NMR (101 MHz, $CDCl_3$) δ 177.15, 170.04, 169.89, 165.82, 138.36, 130.10, 129.53, 126.02, 100.74, 98.44, 82.62, 79.61, 78.74, 70.66, 70.13, 59.77, 52.38, 29.58, 23.34.

HRMS (ESI/QTOF) m/z: $[M + Na]^+$ Calculated for $C_{51}H_{54}N_4NaO_{12}^+$ 937.3630; Found 937.3626.

1.2.2.3.2. Synthesis of 2-azidoethyl-*O*-(2-*O*-benzoyl-3-*O*-benzyl-α-L-idopyranosyl)-(1→4)-*O*-2-acetamido-3,6-*O*-benzyl-2-deoxy-α-D-glucopyranoside (18):

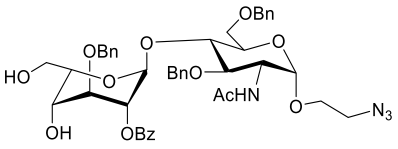

18

For the ring-opening reaction of the disaccharide, compound **17** (5.7 g, 6.2 mmol) was dissolved in DCM (60 mL). TFA (6 mL) and H_2O (600 µL) were added, and the reaction mixture was stirred at R.T. for 4 hours. After the completion, it was diluted with DCM, washed with H_2O. The aqueous layer was extracted with DCM. The organic layer was dried over $MgSO_4$, filtered, and concentrated under reduced pressure. The residue was purified by silica gel column chromatography (MeOH/DCM, 0 to 5%, v/v) to yield the compound **18** (2.2 g, 2.7 mmol, 43%).

^{1}H-NMR (400 MHz, $CDCl_3$) δ 8.50 (s, 3H), 7.93 (s, 1H), 7.99 – 7.83 (m, 2H), 7.58 – 7.46 (m, 1H), 7.45 – 7.28 (m, 3H), 7.31 – 7.19 (m, 15H), 7.22 – 7.11 (m, 2H), 7.10 (ddd, *J* = 8.4, 6.4, 4.9 Hz, 2H), 5.59 – 5.46 (m, 1H), 5.16 (s, 1H), 5.14 – 5.03 (m, 1H), 4.85 – 4.37 (m, 8H), 4.35 – 4.25 (m, 0H), 4.25 – 4.13 (m, 1H), 4.06 – 3.95 (m, 1H), 3.83 (ddd, *J* = 10.8, 5.6, 2.8 Hz, 1H), 3.80 – 3.68 (m, 3H), 3.70 (s, 15H), 3.68 – 3.48 (m, 3H), 3.48 – 3.32 (m, 2H), 3.23 (ddt, *J* = 19.4, 13.5, 2.6 Hz, 1H), 2.91 (s, 1H), 2.83 (s, 1H), 2.66 (s, 11H), 2.57 (s, 1H), 2.18 – 2.02 (m, 1H), 1.87 – 1.75 (m, 3H), 1.18 (s, 1H).

^{13}C-NMR (101 MHz, $CDCl_3$) δ 177.59, 165.44, 138.25, 133.65, 130.09, 126.39, 97.72, 79.47, 73.78, 71.85, 70.71, 68.53, 52.60, 50.55, 29.57, 23.15.

HRMS (ESI/QTOF) m/z: $[M + Na]^+$ Calculated for $C_{44}H_{50}N_4NaO_{12}^+$ 849.3317; Found 849.3313.

1.2.2.3.3. Synthesis of 2-azidoethyl-*O*-(2-*O*-benzoyl-3-*O*-benzyl-α-L-idopyranosyluronate)-(1→4)-*O*-2-acetamido-3,6-*O*-benzyl-2-deoxy-α-D-glucopyranoside (19):

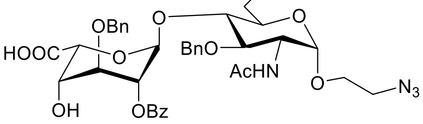

19

For the oxidation reaction, compound **18** (2.2 g, 2.7 mmol) was dissolved in DCM (30 mL) and H_2O (15 mL) followed by the addition of TEMPO (127 mg, 0.81 mmol) and BAIB (2.2 g, 6.8 mmol). The reaction mixture was stirred at R.T. for 6 hours. Upon completion, the mixture was diluted with DCM and washed with $Na_2S_2O_{3(aq)}$ to decolorize and with H_2O. The organic phase was dried over $MgSO_4$, filtered, and concentrated under reduced pressure. The crude carboxyl compound **19** was directly used for the next step without any purification.

1.2.2.3.4. Synthesis of 2-azidoethyl-*O*-(methyl-2-*O*-benzoyl-3-*O*-benzyl-α-L-idopyranosyluronate)-(1→4)-*O*-2-acetamido-3,6-*O*-benzyl-2-deoxy-α-D-glucopyranoside (20):

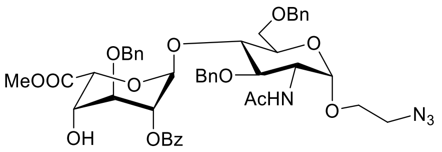

20

For the methylation reaction, compound **19** was dissolved in anhydrous DMF (30 mL) under an argon environment and followed by the addition of MeI (425 µL, 6.8 mmol) and $KHCO_3$ (270 mg, 2.7 mmol). The reaction mixture was stirred at R.T. for 12 hours. After the completion, the mixture was diluted with DCM and washed with ice-cold H_2O. The aqueous phase was extracted with DCM. The organic phase was dried over $MgSO_4$, filtered, and concentrated under reduced pressure. The residue was purified by silica gel column chromatography (MeOH/DCM, 0 to 5%, v/v) to yield the methylated compound **20** (1.1 g, 1.3 mmol, 51%).

^{1}H-NMR (400 MHz, $CDCl_3$) δ 7.91 – 7.84 (m, 3H), 7.53 – 7.45 (m, 1H), 7.38 – 7.22 (m, 6H), 7.24 – 7.16 (m, 4H), 7.20 – 7.00 (m, 15H), 5.34 (d, *J* = 9.3 Hz, 1H), 5.25 (s, 1H), 5.13 (t, *J* = 2.4 Hz, 1H), 4.97 (d, *J* = 2.2 Hz, 1H), 4.79 – 4.66 (m, 2H), 4.66 – 4.56 (m, 2H), 4.45 (s, 2H), 4.39 (d, *J* = 11.8 Hz, 1H), 4.31 – 4.20 (m, 1H), 4.02 (t, *J* = 9.3 Hz, 1H), 3.97 (t, *J* = 3.1 Hz, 1H), 3.83 (t, *J* = 3.6 Hz, 1H), 3.80 – 3.65 (m, 3H), 3.59 (t, *J* = 9.9 Hz, 2H), 3.55 – 3.30 (m, 2H), 3.40 (s, 3H), 3.18 (ddd, *J* = 13.5, 5.7, 2.8 Hz, 1H), 2.81 (s, 2H), 2.76 (s, 2H), 2.61 (s, 1H), 2.56 (s, 2H), 2.26 (s, 5H), 2.26 – 2.10 (m, 1H), 2.05 (s, 2H), 1.64 (s, 3H), 1.24 – 1.11 (m, 1H).

^{13}C-NMR (101 MHz, $CDCl_3$) δ 207.00, 169.99, 165.17, 162.59, 138.23, 129.92, 126.05, 98.03, 78.20, 73.67, 67.26, 53.87, 36.48, 31.75, 23.30, 21.48, 19.75.

HRMS (ESI/QTOF) m/z: $[M + Na]^+$ Calculated for $C_{45}H_{50}N_4NaO_{13}^+$ 877.3267; Found 877.3241.

1.2.2.3.5. Synthesis of 2-azidoethyl-*O*-(3-*O*-benzyl-α-L-idopyranosyluronate)-(1→4)-*O*-2-acetamido-3,6-*O*-benzyl-2-deoxy-α-D-glucopyranoside (21):

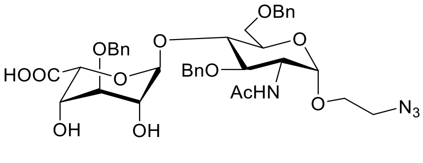

21

For the deprotection of Bz and demethylation reaction, compound **20** (1.1 g, 1.3 mmol) was dissolved in THF (30 mL). LiOH (1.6 g, 65 mmol) and H_2O_2 (13.3 mL, 130 mmol, 30% solution in H_2O containing a stabilizer). The reaction was heated to 40 °C for 12 hours. After the completion, the reaction mixture was concentrated *in vacuo* to obtain the deprotected crude product **21,** and then proceeded to the next step without purification.

HRMS (ESI/QTOF) m/z: $[M]^-$ Calculated for $C_{37}H_{43}N_4O_{12}^-$ 735.2883; Found 735.2877.

1.2.2.3.6. Synthesis of 2-azidoethyl-*O*-(2,4-*O*-sulfate-3-*O*-benzyl-α-L-idopyranosyluronate)-(1→4)-*O*-2-acetamido-3,6-*O*-benzyl-2-deoxy-α-D-glucopyranoside (22):

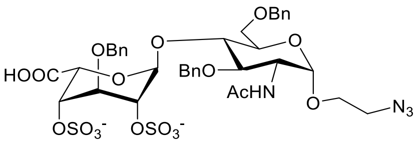

22

For the *O*-sulfation reaction of crude product **21**, $SO_3.EtN_3$ (4.7 g, 26 mmol) was added to the solution of **21** in DMF (20 mL), and kept at 60 °C for 12h. After the completion, MeOH/Et_3N (50%, v/v) was added and stirred for half an hour. The reaction mixture was filtered, and the solvents were removed under reduced pressure. The residue was purified by column chromatography on silica gel (MeOH/$CHCl_3$, 0 to 20%, v/v) to give the sulfated compound **22** (840 mg, 0.94 mmol, 70%).

1.2.2.3.7. Synthesis of 2-aminoethyl-*O*-(2,4-*O*-sulfate-α-L-idopyranosyluronate)-(1→4)-*O*-2-acetamido-2-deoxy-α-D-glucopyranoside (D1):

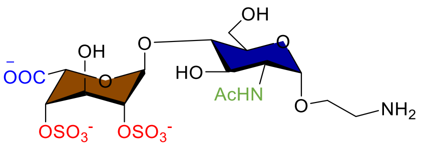

D1

For the debenzylation reaction, compound **22** (840 mg, 0.94 mmol) and 20% $Pd(OH)_2$/C (1.3 g, 1.5x of compound **22** by weight) were dispersed in MeOH/H_2O (50%, v/v) under a hydrogen environment and stirred at R.T. for 24 hours. The next day, the reaction mixture was filtered through a pad of celite, and the residue was washed with H_2O. After removing H_2O, the crude product was purified by size exclusion chromatography using a P-2 Gel column using Milli-Q H_2O as an eluent. The fractions having the product were freeze-dried to yield the final disaccharide (**D1**) (305 mg, 0.51 mmol, 54%).

^{1}H-NMR (600 MHz, D_2O) δ 4.82 (s, 0H), 3.89 (s, 1H), 3.74 (s, 5H), 3.20 (s, 1H), 3.12 (q, *J* = 7.3 Hz, 4H), 1.96 (d, *J* = 3.7 Hz, 1H), 1.83 (s, 1H), 1.71 (s, 3H), 1.42 (s, 12H), 1.20 (t, *J* = 7.3 Hz, 6H).

1.2.3. Synthesis of IdoA(2,4S)-1,4-GlcNAc(6S)O(CH_2)$_2$$NH_2$ (D2):

1.2.3.1. Synthesis of Acceptor for D2 (25):

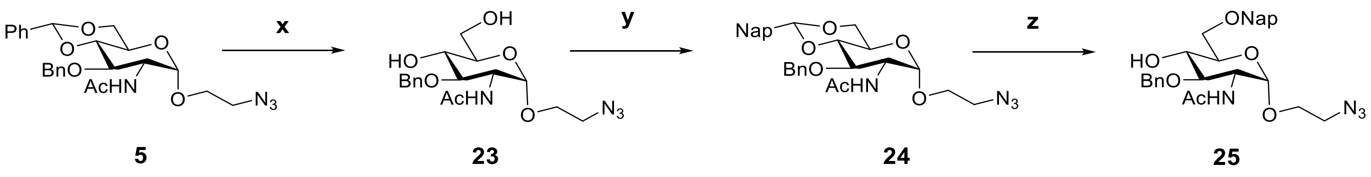

Scheme S5. Synthetic route for the acceptor of **D2**. Reagents and conditions: **(x):** TFA, H_2O, DCM, 30 °C, 9h, 61%; **(y):** NapCH$(OMe)_2$, *p*TsOH, MeCN, 50 °C , 12 h, 65%; **(z):** $NaBH_3CN$, 2N HCl/Et_2O, R.T., 6 h, 75%.

1.2.3.1.1. Synthesis of 2-azidoethyl-3-*O*-benzyl-2-acetamido-2-deoxy-α-D-glucopyranoside (23):

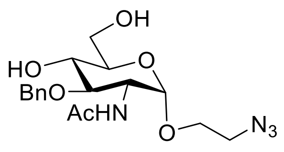

23

For the ring-opening reaction, compound **5** (5.6 g, 12 mmol) was dissolved in DCM (60 mL). TFA (6 mL) and H_2O (600 µL) were added, and the reaction mixture was stirred at 30 °C for 9 hours. After the completion, the reaction was quenched by the slow addition of saturated $NaHCO_{3(aq)}$, followed by diluting with DCM and washing with H_2O. The aqueous layer was extracted with DCM. The organic layer was dried over $MgSO_4$, filtered, and concentrated under reduced pressure. The residue was purified by silica gel column chromatography (MeOH/EtOAc, 0 to 10%, v/v) to yield the compound **23** (2.8 g, 7.4 mmol, 61%).

^{1}H-NMR (400 MHz, $CDCl_3$) δ 7.42 – 7.27 (m, 6H), 5.53 (d, J = 9.3 Hz, 1H), 4.87 (d, J = 3.6 Hz, 1H), 4.78 (d, J = 11.8 Hz, 1H), 4.71 (d, J = 11.8 Hz, 1H), 4.26 (ddd, J = 10.5, 9.3, 3.6 Hz, 1H), 3.97 – 3.75 (m, 4H), 3.75 – 3.56 (m, 3H), 3.50 (ddd, J = 13.4, 8.1, 2.8 Hz, 1H), 3.29 (ddd, J = 13.4, 5.3, 2.6 Hz, 1H), 2.92 – 2.83 (m, 2H), 1.89 (s, 3H).

^{13}C-NMR (101 MHz, $CDCl_3$) δ 170.43, 138.23, 128.68, 98.01, 79.71, 76.72, 74.29, 72.02, 70.60, 67.22, 62.01, 52.02, 50.50, 23.27.

HRMS (ESI/QTOF) m/z: $[M + H]^+$ Calculated for $C_{17}H_{25}N_4O_6^+$ 381.1769; Found 381.1761.

1.2.3.1.2. Synthesis of 2-azidoethyl-3-*O*-benzyl-4,6-*O*-(2-naphthylmethylbenzylidene)-2-acetamido-2-deoxy-α-D-glucopyranoside (24):

For the synthesis of compound **24**, first, $NapCH(OMe)_2$ protecting reagent was prepared as shown in Scheme 5.1 below.

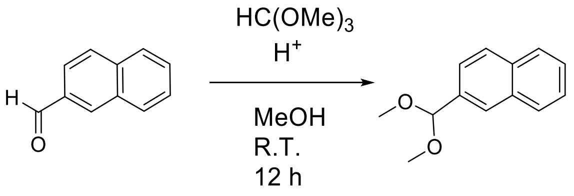

Scheme S5.1. Synthetic route for the $NapCH(OMe)_2$.

2-naphthaldehyde (4.0 g, 25.6 mmol) was dissolved in MeOH (80 mL), followed by the addition of $HC(OMe)_3$ (8.2 g, 78 mmol) and Amberlite IR 120 (H^+) resin (4.0 g). The reaction was stirred at R.T. for 12 hours. After the completion, the resin was filtered through a pad of celite, and the filtrate was neutralized by adding saturated $NaHCO_{3(aq)}$. It was washed with H_2O and diluted with DCM. The aqueous layer was extracted in DCM and dried over $MgSO_4$, filtered, and concentrated under reduced pressure. The residue appears as a yellowish, transparent, clear liquid.

HRMS (ESI/QTOF) m/z: $[M + Na]^+$ Calculated for $C_{13}H_{14}NaO_2^+$ 225.0886; Found 225.0896.

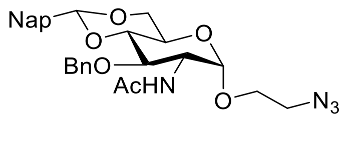

24

Compound **23** (2.8 g, 7.4 mmol) was dissolved in MeCN (40 mL). $NapCH(OMe)_2$ (3.0 g, 14.8 mmol) and *p*TsOH monohydrate (282 mg, 1.48 mmol) were added. The reaction was stirred at 50 °C for 12 hours. Upon completion, the reaction was quenched by the addition of a few drops of Et_3N. The MeCN was evaporated *in vacuo*. The residue was purified by silica gel column chromatography (MeOH/DCM, 0 to 10%, v/v) to yield the compound **24** as a white powder (2.5 g, 4.8 mmol, 65%).

^{1}H-NMR (400 MHz, $CDCl_3$) δ 8.01 (s, 2H), 8.00 – 7.94 (m, 1H), 7.90 (td, *J* = 9.2, 6.0 Hz, 4H), 7.72 – 7.58 (m, 2H), 7.57 – 7.50 (m, 3H), 7.35 (s, 1H), 7.34 (s, 4H), 7.28 (s, 10H), 5.79 (s, 1H), 5.43 (d, *J* = 9.1 Hz, 1H), 4.99 – 4.85 (m, 3H), 4.80 – 4.65 (m, 2H), 4.44 – 4.30 (m, 3H), 4.00 – 3.74 (m, 7H), 3.74 – 3.59 (m, 2H), 3.58 – 3.47 (m, 2H), 3.30 (ddd, *J* = 13.3, 5.1, 2.6 Hz, 1H), 1.93 (s, 4H).

HRMS (ESI/QTOF) m/z: $[M + Na]^+$ Calculated for $C_{28}H_{30}N_4NaO_6^+$ 541.2058; Found 541.2062.

1.2.3.1.3. Synthesis of 2-azidoethyl-3-*O*-benzyl-6-*O*-(2-naphthylmethyl)-2-acetamido-2-deoxy-α-D-glucopyranoside (25):

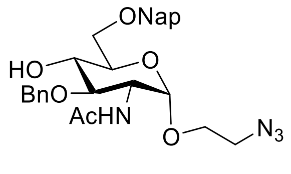

25

Compound **24** (2.5 g, 4.8 mmol) was dissolved in THF (40 mL). $NaBH_3CN$ (3.0 g, 48 mmol) was added. A 2N HCl/Et_2O solution was added dropwise to maintain the reaction mixture at a pH of 1. After stirring at R.T. for 6 hours, the reaction mixture was diluted with EtOAc, washed with $NaHCO_{3(aq)}$ and brine, dried over $MgSO_4$, and concentrated under reduced pressure. The crude material was then purified by silica gel column chromatography (EtOAc/Hex, 66%, v/v) to yield the compound **25** as a viscous liquid (1.9 g, 3.7 mmol, 75%).

^{1}H-NMR (400 MHz, $CDCl_3$) δ 7.92 (d, *J* = 4.3 Hz, 2H), 7.87 – 7.73 (m, 6H), 7.54 (ddd, *J* = 8.5, 6.0, 1.8 Hz, 2H), 7.48 – 7.38 (m, 4H), 7.28 (s, 2H), 7.24 (s, 10H), 7.18 – 7.06 (m, 2H), 5.68 (s, 2H), 5.36 (d, *J* = 9.2 Hz, 1H), 4.87 (s, 1H), 4.85 – 4.81 (m, 2H), 4.81 – 4.71 (m, 1H), 4.60 (s, 1H), 4.58 (d, *J* = 3.7 Hz, 1H), 4.36 – 4.23 (m, 3H), 4.23 – 4.16 (m, 1H), 4.16 – 4.02 (m, 1H), 4.02 – 3.84 (m, 2H), 3.84 – 3.69 (m, 8H), 3.69 – 3.64 (m, 1H), 3.61 – 3.35 (m, 4H), 3.20 (ddd, *J* = 13.5, 5.2, 2.7 Hz, 2H), 2.37 – 2.23 (m, 1H), 2.13 (s, 1H), 2.05 – 1.93 (m, 1H), 1.84 (s, 5H), 1.55 (s, 1H).

^{13}C-NMR (101 MHz, $CDCl_3$) δ 170.80, 138.34, 129.05, 123.68, 101.62, 98.47, 95.71, 82.82, 82.72, 77.37, 65.71, 58.65, 52.38, 52.35, 35.66, 29.34, 23.28, 21.55.

HRMS (nanochip-ESI/LTQ-Orbitrap) m/z: $[M + H]^+$ Calculated for $C_{28}H_{33}N_4O_6^+$ 521.2395; Found 521.2382.

1.2.3.2. Synthesis of D2:

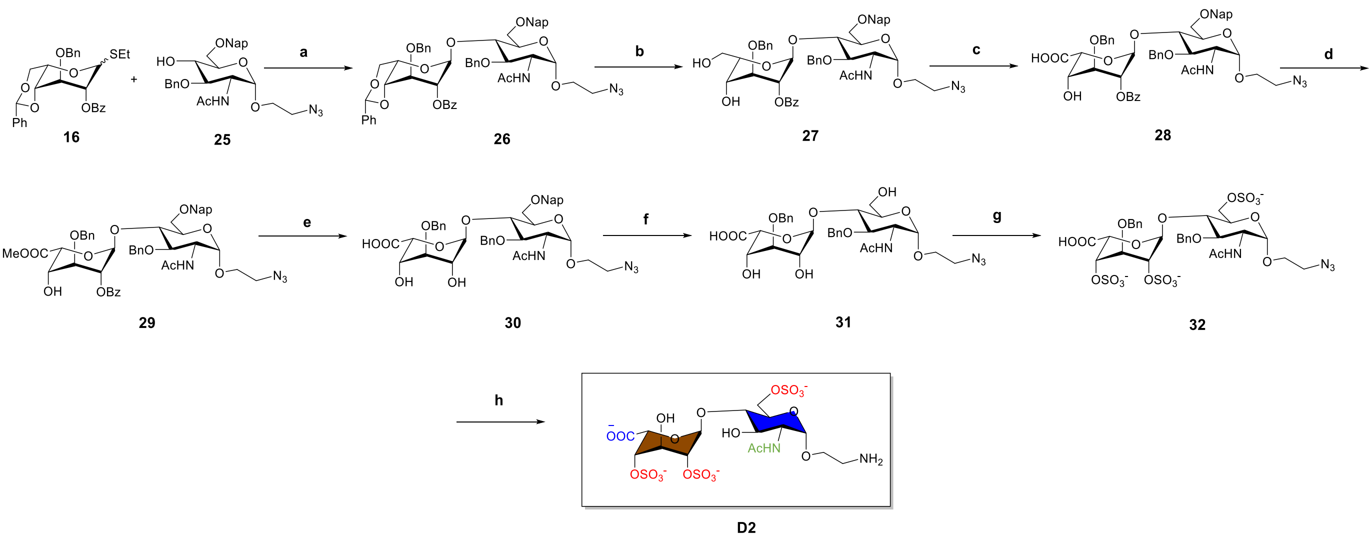

Scheme S6. Synthetic route for **D2**. Reagents and conditions: **(a):** AgOTf, NIS, molecular sieves (4 Å), DCM, 0 °C, 30 min, 33%; **(b):** TFA/H_2O, DCM, R.T., 4h, 38%; **(c):** TEMPO, BAIB, DCM/H_2O, R.T., 6h; **(d):** MeI, $KHCO_3$, DMF, R.T., 12h, 45%; **(e):** LiOH, H_2O_2, THF, 40°C, 12h; **(f):** DDQ, DCM/MeOH, R.T., 6h, 52%; **(g):** $SO_3.EtN_3$, DMF, 12h, 61%; **(h):** $Pd(OH)_2/C$, MeOH: H_2O, under H_2, R.T., 24, 50%.

1.2.3.2.1. Synthesis of 2-azidoethyl-*O*-(2-*O*-benzoyl-3-*O*-benzyl-4,6-*O*-benzylidene-α-L-idopyranosyl)-(1→4)-*O*-2-acetamido-3-*O*-benzyl-6-*O*-(2-naphthylmethyl)-2-deoxy-α-D-glucopyranoside (26):

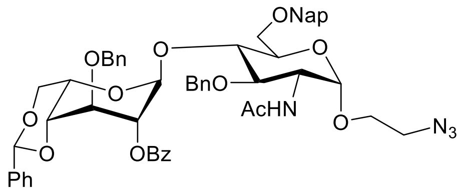

26

For the glycosylation reaction, thioethyl L-idosyl donor **16** (13.5 g, 27 mmol) and GlcNAc acceptor **25** (8.43 g, 16.2 mmol) were dissolved in anhydrous DCM (150 mL) and stirred with pre-activated molecular sieves (13.5 g) for 1 hour at R.T. under an argon environment. The reaction mixture was then cooled down to 0 °C, followed by the addition of promoters, NIS (12 g, 53 mmol) and AgOTf (694 mg, 2.7 mmol). The reaction mixture was stirred at 0 °C for half an hour. After the completion, it was quenched with Et_3N (~1 mL). The mixture was diluted with DCM and washed with $Na_2S_2O_{3(aq)}$ to decolorize, brine, and H_2O. The organic phase was dried over $MgSO_4$, filtered, and concentrated under reduced pressure. The residue was purified by silica gel column chromatography (EtOAc/Hex, 0 to 70%, v/v) to yield the compound **26** (8.5 g, 8.8 mmol, 33%)

^{1}H-NMR (400 MHz, $CDCl_3$) δ 7.95 – 7.74 (m, 6H), 7.76 – 7.56 (m, 1H), 7.59 – 7.50 (m, 1H), 7.54 – 7.47 (m, 2H), 7.50 – 7.36 (m, 2H), 7.40 – 7.23 (m, 6H), 7.26 – 7.14 (m, 2H), 5.02 – 4.62 (m, 6H), 4.62 – 4.39 (m, 2H), 4.39 – 4.22 (m, 1H), 4.26 – 4.10 (m, 1H), 4.14 – 3.96 (m, 2H), 4.00 – 3.70 (m, 4H), 3.73 – 3.62 (m, 1H), 3.66 – 3.47 (m, 1H), 3.36 – 3.17 (m, 1H), 2.86 – 2.60 (m, 1H), 2.63 – 2.37 (m, 1H), 2.38 (s, 1H), 2.13 (d, J = 5.4 Hz, 2H), 2.10 – 1.90 (m, 1H), 1.88 (s, 1H), 1.74 (s, 1H), 1.33 – 1.18 (m, 2H), 1.05 (t, *J* = 7.6 Hz, 1H).

^{13}C-NMR (101 MHz, $CDCl_3$) δ 173.86, 170.93, 165.88, 138.88,125.67, 101.62, 95.31, 82.82, 78.76, 59.83, 58.81, 58.65, 52.47, 52.26, 51.30, 50.56, 35.78, 35.69, 23.33, 21.99, 4.84.

HRMS (ESI/QTOF) m/z: $[M + Na]^+$ Calculated for $C_{55}H_{56}N_4NaO_{12}^+$ 987.3787; Found 987.3790.

1.2.3.2.2. D2

From the protected disaccharide **26** to **D2**, the same deprotection steps used for **D1** were followed, except for the 6-*O*-(2-naphthylmethyl) group, which was cleaved using DDQ.[1]

^{1}H-NMR (600 MHz, D_2O) δ 5.87 (d, *J* = 4.6 Hz, 2H), 5.41 (d, *J* = 3.7 Hz, 2H), 4.50 – 4.41 (m, 4H), 4.27 (t, *J* = 3.8 Hz, 2H), 4.24 (s, 3H), 3.81 (dt, *J* = 10.3, 5.6 Hz, 3H), 3.75 (d, *J* = 9.7 Hz, 1H), 3.73 (s, 3H), 3.66 (p, *J* = 10.1 Hz, 3H), 3.60 – 3.54 (m, 2H), 2.73 (s, 2H), 2.74 – 2.69 (m, 1H), 2.68 (dd, *J* = 13.8, 5.3 Hz, 2H), 1.96 (s, 7H).

^{13}C-NMR (151 MHz, D_2O) δ 174.67, 169.15, 144.71, 106.57, 101.45, 96.95, 78.40, 75.17, 72.33, 66.54, 63.45, 55.38, 40.03, 22.10.

1.2.4. Synthesis of D3

1.2.4.1. Acceptor for D3

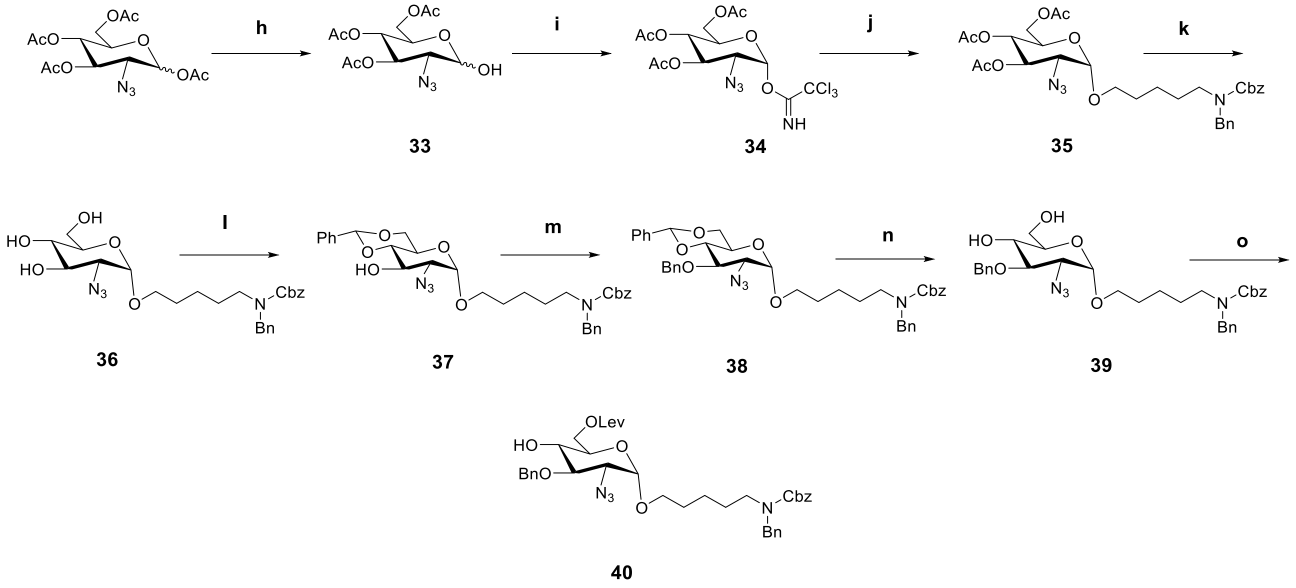

Scheme S7. Synthetic route for the acceptor of **D3**. Reagents and conditions: **(h):** N_2H_4.HOAc, DMF, R.T., 2h, 92%; **(i):** CCl_3CN, DBU, DCM, 0 °C - R.T., 12h, 77%; **(j):** *N*-benzyl-*N*-

benzyloxycarbonyl-5-aminopentan-1-ol, DCM/Et_2O, molecular sieves (3 Å), -20 °C - R.T., 2-3h, 78%; **(k):** NaOMe, MeOH, R.T., 12h, 90%; **(l):** $PhCH(OMe)_2$, CSA, MeCN, R.T., 12h, 81%; **(m):** NaH (60% dispersion in mineral oil), BnBr, DMF, 0 °C - R.T., 12h, 80%; **(n):** AcOH/H_2O, 90 °C, 1h, 71%; **(o):** Levulinic acid, CMPI, DABCO, DCM, -20 °C - R.T., 2-3h, 69%.

1.2.4.1.1. Synthesis of 3,4,6-tri-*O*-acetyl-2-azido-2-deoxy-α/β-D-glucopyranose (33):

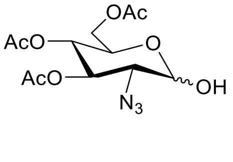

33

1,3,4,6-Tetra-*O*-acetyl-2-azido-2-deoxy-β-D-glucopyranose (10.0 g, 26.8 mmol) was dissolved in DMF (100 mL). N_2H_4.HOAc (3.0 g, 32.2 mmol) was added at R.T. under an argon atmosphere. The reaction mixture was stirred for 2 hours. After the completion, it was diluted with DCM and washed with ice-cold H_2O. The aqueous layer was extracted with DCM. The organic layer was dried over $MgSO_4$ and concentrated under reduced pressure. The crude material was then purified by silica gel column chromatography (EtOAc/petroleum ether, 33%, v/v) to yield the compound **33** as a white fluffy solid (8.2 g, 24.8 mmol, 92%).

1.2.4.1.2. Synthesis of 3,4,6-tri-*O*-acetyl-2-azido-2-deoxy-α/β-D-glucopyranosyl trichloroacetimidate (34):

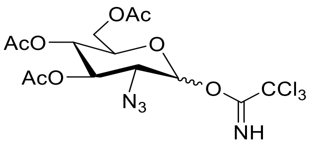

34

Compound **33** (8.2 g, 24.8 mmol) is dissolved in anhydrous DCM (50 mL). CCl_3CN (15 mL, 148.8 mmol) was added and stirred at 0 °C. Afterward, DBU (2.3 mL, 15 mmol) was added, and the reaction mixture was stirred at R.T. overnight. After the completion, it was diluted with DCM and washed with H_2O. The aqueous layer was extracted with DCM. The organic layer was dried over $MgSO_4$ and concentrated under reduced pressure. The crude material was then purified by silica gel column chromatography (EtOAc/Hex, 0 to 50%, v/v and a few drops of Et_3N) to yield the compound **34** as a white solid (9.0 g, 18.9 mmol, 77%).

^{1}H-NMR (400 MHz, $CDCl_3$) δ 8.84 (d, *J* = 11.7 Hz, 2H), 6.50 (d, *J* = 3.6 Hz, 1H), 5.73 (d, *J* = 8.4 Hz, 1H), 5.57 – 5.46 (m, 1H), 5.21 – 5.06 (m, 3H), 4.37 – 4.18 (m, 3H), 4.17 – 4.05 (m, 3H), 3.90 – 3.75 (m, 2H), 2.15 – 2.01 (m, 18H), 1.26 (t, *J* = 7.1 Hz, 1H).

^{13}C-NMR (101 MHz, $CDCl_3$) δ 171.15, 170.75, 160.66, 138.65, 111.22, 96.38, 94.01, 90.51, 90.16, 88.43, 77.37, 72.67, 72.62, 70.65, 70.10, 67.92, 67.90, 67.30, 65.58, 63.23, 62.55, 61.46, 61.39, 60.63, 60.39, 21.04, 20.99, 20.78, 20.69, 20.66, 20.64, 20.57, 20.56, 14.19.

HRMS (ESI/QTOF) m/z: $[M + Na]^+$ Calculated for $C_{14}H_{17}Cl_3N_4NaO_8^+$ 497.0004; Found 497.0011.

1.2.4.1.3. Synthesis of *N*-benzyl-*N*-benzyloxycarbonyl-5-aminopentyl-3,4,6-tri-*O*-acetyl-2-azido-2-deoxy-α-D-glucopyranoside (35):

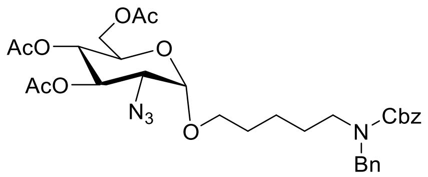

35

Compound **34** (9.0 g, 18.9 mmol) was co-evaporated with *N*-benzyl-*N*-benzyloxycarbonyl-5-aminopentan-1-ol (6.7 g, 28.4 mmol) in anhydrous Tol (150 mL) and dried under a Schlenk line overnight. It was dissolved in a mixture of anhydrous DCM (120 mL) and Et_2O (400 mL) under an argon atmosphere. Freshly activated molecular sieves (3 Å, 18 g) were added, and the reaction mixture was stirred at R.T. for 1 hour. Afterwards, the temperature was brought to -20 °C and TMSOTf (171 µL, 0.95 mmol) was added slowly. It was stirred for another 1 hour at -20 °C and allowed to reach R.T. followed by quenching with Py (500 µL). The reaction mixture was filtered through a pad of celite and washed with $NaHCO_{3(aq)}$ and brine. The aqueous phase was extracted with DCM, and the organic phase was dried over $MgSO_4$ and concentrated under reduced pressure. The crude material was then purified by silica gel column chromatography (EtOAc/Tol, 0 to 50%, v/v) to yield the compound **35** as a colorless viscous liquid (9.5 g, 14.8 mmol, 78%).

HRMS (ESI/QTOF) m/z: $[M + Na]^+$ Calculated for $C_{32}H_{40}N_4NaO_{10}^+$ 663.2637; Found 663.2668.

1.2.4.1.4. Synthesis of *N*-benzyl-*N*-benzyloxycarbonyl-5-aminopentyl-*O*-2-azido-2-deoxy-α-D-glucopyranoside (36):

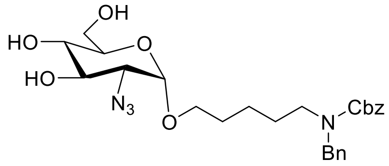

36

For the deacetylation reaction, compound **35** (9.5 g, 14.8 mmol) was dissolved in anhydrous MeOH (100 mL). NaOMe (162 mg, 3 mmol) was added, and the reaction mixture was stirred at R.T. for 12 hours. After the completion, the reaction mixture was neutralized with Dowex 50W X8 ion exchange resin (H^+ form). The resin was filtered through a pad of celite. The filtrate was concentrated *in vacuo*. The crude material was then purified by silica gel column chromatography (MeOH/DCM, 0 to 10%, v/v) to yield the compound **36** (6.9 g, 13.4 mmol, 90%).

1.2.4.1.5. Synthesis of *N*-Benzyl-*N*-benzyloxycarbonyl-5-aminopentyl-4,6-benzylidene-*O*-2-azido-2-deoxy-α-D-glucopyranoside (37):

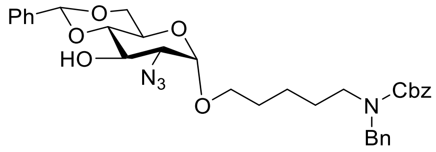

37

For the benzylidene protection, compound **36** (6.9 g, 13.4 mmol) was dissolved in MeCN (70 mL). $PhCH(OMe)_2$ (2.96 mL, 20.1 mmol) and CSA (232 mg, 1 mmol) were added, and the reaction mixture was stirred at R.T. overnight. After the completion, the reaction mixture was neutralized with Et_3N and directly concentrated under reduced pressure. The crude material was then purified by silica gel column chromatography (EtOAc/Hex, 0 to 30%, v/v) to yield the compound **37** (6.5 g, 10.8 mmol, 81%).

1.2.4.1.6. Synthesis of *N*-Benzyl-*N*-benzyloxycarbonyl-5-aminopentyl-*O*-2-azido-3-*O*-benzyl-4,6-*O*-benzylidene-2-deoxy-α-D-glycopyranoside (38):

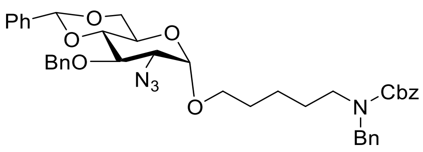

38

Compound **37** (6.5 g, 10.8 mmol) was dissolved in anhydrous DMF (80 mL). The solution was cooled down to 0 °C. NaH (1.2 g, 30.0 mmol, 60% dispersion in mineral oil) was added and stirred for half an hour. Then, BnBr (1.5 mL, 13.0 mmol) was added while stirring, and the ice bath was removed. The reaction was allowed to warm up to R.T. and stirred overnight. After the completion, the reaction was quenched with AcOH (400 μL), diluted with EtOAc, and washed with cold H_2O. The aqueous layer was extracted with EtOAc (3 x 100 mL). The combined organic layers were dried over Na_2SO_4, filtered, and concentrated under reduced pressure. The residue was purified by column chromatography on silica gel (EtOAc/Hex, 0 to 30%, v/v) to give compound **38** (6.0 g, 8.7 mmol, 80%).

^{1}H NMR (400 MHz, $CDCl_3$) δ 7.52 (tt, *J* = 7.5, 2.4 Hz, 2H), 7.45 – 7.25 (m, 13H), 5.60 (d, *J* = 9.8 Hz, 1H), 5.20 (d, *J* = 11.5 Hz, 2H), 4.96 (dd, *J* = 19.1, 11.2 Hz, 1H), 4.83 (t, *J* = 9.4 Hz, 2H), 4.56 – 4.50 (m, 2H), 4.40 – 4.26 (m, 1H), 4.10 (t, *J* = 9.4 Hz, 1H), 3.89 (s, 1H), 3.83 – 3.62 (m, 2H), 3.48 – 3.28 (m, 2H), 3.24 (s, 1H).

^{13}C-NMR (101 MHz, $CDCl_3$) δ 137.90, 129.05, 128.55, 128.43, 128.38, 128.30, 128.18, 127.88, 127.85, 125.99, 101.41, 98.55, 82.89, 77.34, 77.23, 77.02, 76.71, 76.06, 75.05, 68.94, 67.17, 62.97, 62.73, 29.07, 23.31.

1.2.4.1.7. Synthesis of *N*-Benzyl-*N*-benzyloxycarbonyl-5-aminopentyl-*O*-2-azido-3-*O*-benzyl-2-deoxy-α-D-glycopyranoside (39):

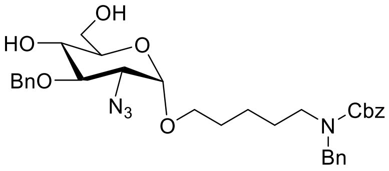

39

For the ring-opening reaction, compound **38** (6.0 g, 8.7 mmol), AcOH (70 mL), and H_2O (7 mL) were mixed, and the reaction mixture was stirred at 90 °C for 1 hour. The reaction mixture was co-evaporated with Tol, and the resulting residue was purified by silica gel chromatography using (EtOAc/Hex, 0 to 40%, v/v) to give compound **39** (3.7 g, 6.12 mmol, 71%).

1.2.4.1.8. Synthesis of *N*-Benzyl-*N*-benzyloxycarbonyl-5-aminopentyl-*O*-2-azido-2-deoxy-3-*O*-benzyl-6-*O*-levulinoyl-α-D-glucopyranoside (40) (Acceptor):

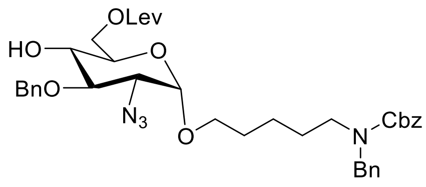

40

Compound **39** (3.7 g, 6.12 mmol) was dissolved in anhydrous DCM (50 mL) under an argon atmosphere. Levulinic acid (0.77 g, 6.7 mmol) and CMPI (3.9 g, 15.3 mmol) were added, and the reaction mixture was stirred for 15 minutes. Then the reaction mixture was cooled down to -20 °C and DABCO (2.8 g, 25 mmol) was added at that temperature. Later, the reaction mixture was allowed to warm to R.T. and kept stirring for 2 hours. Upon completion, the reaction mixture was filtered through a pad of celite, diluted with EtOAc, and washed with brine and H_2O. The aqueous layer was extracted with EtOAc. The combined organic layers were dried over $MgSO_4$, filtered, and concentrated *in vacuo*. The resulting crude material was purified by silica gel chromatography using (EtOAc/Hex, 0 to 70%, v/v) to give compound **40** (3.0 g, 4.27 mmol, 69%).

1.2.4.2. Donor for D3

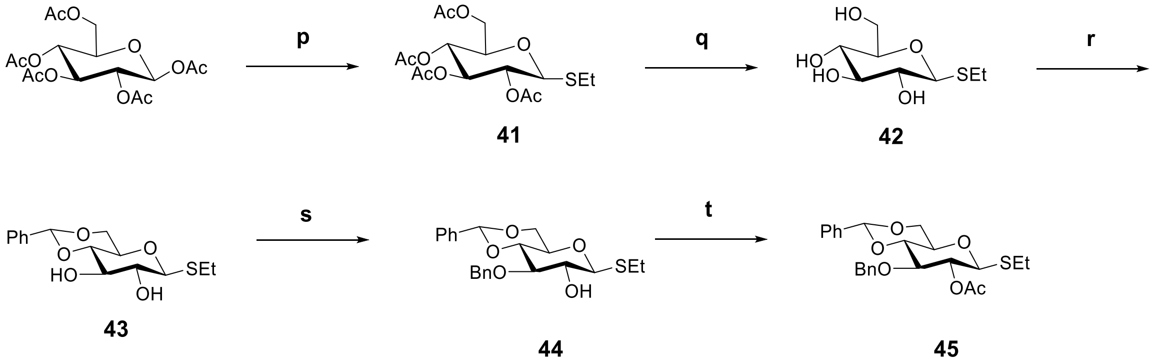

Scheme S8. Synthetic route for the donor of **D3**. Reagents and conditions: **(p):** EtSH, $SnCl_4$, DCM, R.T., 16h, 82%; **(q):** NaOMe, MeOH, R.T., 12h, 91%; **(r):** $PhCH(OMe)_2$, CSA, DMF, 50 °C, 3h, 78%; **(s): i):** Bu_2SnO, MeOH, reflux at 90 °C, 12h; **ii)** BnBr, CsF, DMF, 60 °C, 12h, 51%; **(t):** Ac_2O, Py, R.T., 6h, 87%.

1.2.4.2.1. Synthesis of Ethyl 2,3,4,6-tetra-*O*-acetyl-β-D-thioglucopyranoside (41):

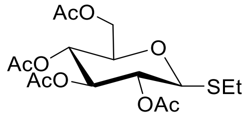

41

1,2,3,4,6-penta-*O*-acetyl-β-D-glucopyranose (10.0 g, 25.6 mmol) was dissolved in anhydrous DCM (100 mL) under an argon environment, followed by the addition of EtSH (2.26 mL, 30.72 mmol) and anhydrous $SnCl_4$ (450 µL, 3.84 mmol). The reaction mixture was stirred at R.T. for 16 hours. After completion, it was diluted with DCM and washed with $NaHCO_{3(aq)}$ and brine. The aqueous phase was extracted with DCM, and the organic phase was dried over $MgSO_4$ and concentrated under reduced pressure. The crude material was then purified by silica gel column chromatography (EtOAc/Hex, 0 to 30%, v/v) to yield the compound **41** (8.28 g, 21.1 mmol, 82%).

^{1}H-NMR (600 MHz, $CDCl_3$) δ 5.25 (t, *J* = 9.4 Hz, 1H), 5.14 – 5.02 (m, 2H), 4.52 (d, *J* = 10.1 Hz, 1H), 4.27 (dd, *J* = 12.3, 5.0 Hz, 1H), 4.16 (dd, *J* = 12.4, 2.4 Hz, 1H), 3.73 (ddd, *J* = 10.1, 5.0, 2.4 Hz, 1H), 2.80 – 2.66 (m, 2H), 2.09 (d, *J* = 11.6 Hz, 6H), 2.04 (d, *J* = 10.8 Hz, 6H), 1.30 (t, *J* = 7.4 Hz, 3H), 1.27 (s, 1H).

^{13}C-NMR (151 MHz, $CDCl_3$) δ 170.69, 170.23, 169.44, 83.54, 77.23, 76.81, 75.89, 73.93, 69.86, 68.36, 62.19, 24.20, 20.74, 14.83.

HRMS (APCI/QTOF) m/z: $[M + Na]^+$ Calculated for $C_{16}H_{24}NaO_9S^+$ 415.1033; Found 415.1035.

1.2.4.2.2. Synthesis of Ethyl-α-D-thioglucopyranoside (42):

Compound **41** (8.28 g, 21.1 mmol) was dissolved in anhydrous MeOH (90 mL) under an argon atmosphere. NaOMe (228 mg, 4.22 mmol) was added to the reaction mixture, and it was stirred at R.T. for 12 hours. Afterward, Dowex 50W X8 ion-exchange resin (H^+ form, ~2.0 g) was added, and the reaction mixture was stirred for an additional hour. The resin was filtered through a pad of celite. The filtrate was concentrated *in vacuo* to yield the compound **42** (4.2 g, 18.7 mmol, 91%). Compound **42** was directly used for the benzylidene protection reaction without further purification.

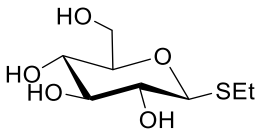

42

HRMS (APCI/QTOF) m/z: $[M + Na]^+$ Calculated for $C_8H_{16}NaO_5S^+$ 247.0611; Found 247.0607.

1.2.4.2.3. Synthesis of Ethyl-4,6-*O*-benzylidene-α-D-thioglucopyranoside (43):

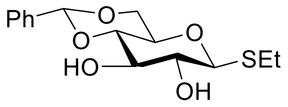

43

Compound **42** (4.2 g, 18.7 mmol) was dissolved in anhydrous DMF (50 mL) under an argon atmosphere. $PhCH(OMe)_2$ (3.37 mL, 22.44 mmol) and CSA (868.8 mg, 3.74 mmol) were added, and the reaction mixture was stirred at 50 °C for 2 hours. After the completion, the reaction was quenched with Et_3N. It was diluted with EtOAc, washed with $NaHCO_{3(aq)}$ and ice-cold H_2O. The aqueous phase was extracted with EtOAc, and the combined organic layers were dried over $MgSO_4$ and concentrated under reduced pressure. The crude material was then purified by silica gel column chromatography (EtOAc/Hex, 0 to 50%, v/v) to yield the compound **43** as a white solid (4.56 g, 14.6 mmol, 78%).

^{1}H-NMR (600 MHz, $CDCl_3$) δ 7.52 (dd, *J* = 7.1, 2.4 Hz, 2H), 7.43 – 7.37 (m, 3H), 7.30 – 7.26 (m, 10H), 4.50 (d, *J* = 9.7 Hz, 1H), 4.38 (dd, *J* = 10.5, 4.8 Hz, 1H), 3.88 (dd, *J* = 9.8, 7.7 Hz, 1H), 3.58 – 3.51 (m, 2H), 2.83 – 2.77 (m, 1H), 2.76 (d, *J* = 2.3 Hz, 1H), 2.59 (d, *J* = 2.2 Hz, 1H), 1.59 – 1.54 (m, 28H), 1.36 (td, *J* = 7.5, 0.9 Hz, 3H), 1.27 (s, 1H).

^{13}C-NMR (151 MHz, $CDCl_3$) δ 129.34, 128.38, 126.27, 101.97, 86.70, 80.39, 77.23, 77.02, 76.81, 74.60, 73.22, 70.60, 68.62, 24.83, 15.33.

HRMS (ESI/QTOF) m/z: $[M + Na]^+$ Calculated for $C_{15}H_{20}NaO_5S^+$ 335.0924; Found 335.0931.

1.2.4.2.4. Synthesis of Ethyl-3-*O*-benzyl-4,6-*O*-benzylidene-α-D-thioglucopyranoside (44):

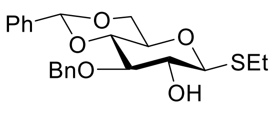

44

Compound **43** (4.56 g, 14.6 mmol) was dissolved in anhydrous MeOH (50 mL) under an argon atmosphere. Bu_2SnO (7.27 g, 29.2 mmol) was added and the reaction mixture was refluxed at 90 °C for 12 hours. MeOH was evaporated under reduced pressure, and the crude material was dried under a Schlenk line thoroughly for ~6 hours. The resulting residue was dissolved in anhydrous DMF (60 mL), followed by the addition of BnBr (3.47 mL, 29.2 mmol) and CsF (3.33 g, 21.9 mmol), and stirred at 60 °C for 12 hours. After the completion, the reaction mixture was washed with ice-cold H_2O. The aqueous phase was extracted with EtOAc, and the combined organic layers were dried over $MgSO_4$ and concentrated under reduced pressure. The crude material was then purified by silica gel column chromatography (EtOAc/Hex, 0 to 30%, v/v) to yield the compound **44** (2.99 g, 7.43 mmol, 51%).

^1H-NMR (600 MHz, $CDCl_3$) δ 7.53 – 7.48 (m, 2H), 7.44 – 7.27 (m, 9H), 5.60 (s, 1H), 5.00 (d, *J* = 11.6 Hz, 1H), 4.84 (d, *J* = 11.6 Hz, 1H), 4.49 (d, *J* = 9.7 Hz, 1H), 4.38 (dd, *J* = 10.5, 5.0 Hz, 1H), 3.80 (t, *J* = 10.3 Hz, 1H), 3.72 (dt, *J* = 24.7, 9.1 Hz, 2H), 3.61 (ddd, *J* = 10.0, 8.0, 2.0 Hz, 1H), 3.57 – 3.49 (m, 1H), 2.77 (qd, *J* = 7.5, 6.1 Hz, 2H), 2.55 (d, *J* = 2.1 Hz, 1H), 1.34 (t, *J* = 7.4 Hz, 3H), 1.28 (s, 1H).

HRMS (ESI/QTOF) m/z: $[M + Na]^+$ Calculated for $C_{22}H_{26}NaO_5S^+$ 425.1393; Found 425.1407.

1.2.4.2.5. Synthesis of Ethyl-2-*O*-acetyl-3-*O*-benzyl-4,6-*O*-benzylidene-α-D-thioglucopyranoside (45):

For the *O*-acetylation reaction, compound **44** (2.99 g, 7.43 mmol) was dissolved in Py (12 mL), and Ac_2O (6 mL) was added. The reaction mixture was stirred at R.T. for 6 hours. After the

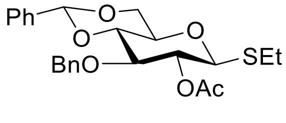

45

completion, the mixture was diluted with EtOAc, washed with $NaHCO_{3(aq)}$ and ice-cold H_2O. The aqueous phase was extracted with EtOAc, and the combined organic layers were dried over $MgSO_4$ and concentrated under reduced pressure. The crude material was then purified by silica gel column chromatography (EtOAc/Hex, 0 to 20%, v/v) to yield the compound **45** (2.88 g, 6.48 mmol, 87%).

^{1}H-NMR (600 MHz, $CDCl_3$) δ 7.52 (dd, *J* = 7.8, 1.9 Hz, 2H), 7.45 – 7.36 (m, 3H), 7.36 – 7.27 (m, 5H), 5.61 (s, 1H), 5.11 – 5.04 (m, 1H), 4.90 (d, *J* = 12.0 Hz, 1H), 4.71 (d, *J* = 12.0 Hz, 1H), 4.49 (d, *J* = 10.0 Hz, 1H), 4.40 (dd, *J* = 10.5, 5.0 Hz, 1H), 3.81 (t, *J* = 10.2 Hz, 1H), 3.81 – 3.74 (m, 2H), 3.55 – 3.48 (m, 1H), 2.79 – 2.66 (m, 2H), 2.04 (d, *J* = 0.6 Hz, 3H), 1.27 (t, *J* = 7.4 Hz, 3H).

^{13}C-NMR (151 MHz, $CDCl_3$) δ 169.49, 138.17, 137.15, 129.06, 128.34, 128.30, 127.89, 127.71, 126.01, 101.25, 84.23, 81.57, 79.70, 77.24, 77.03, 76.81, 74.36, 71.24, 70.68, 68.63, 23.92, 20.93, 14.81.

HRMS (APCI/QTOF) m/z: $[M + Na]^+$ Calculated for $C_{24}H_{28}NaO_6S^+$ 467.1499; Found 467.1497.

1.2.4.3. Synthesis of GlcANHNS(2,4S)-1,4-GlcNS(6S)O$(CH_2)_5NH_2$ (D3):

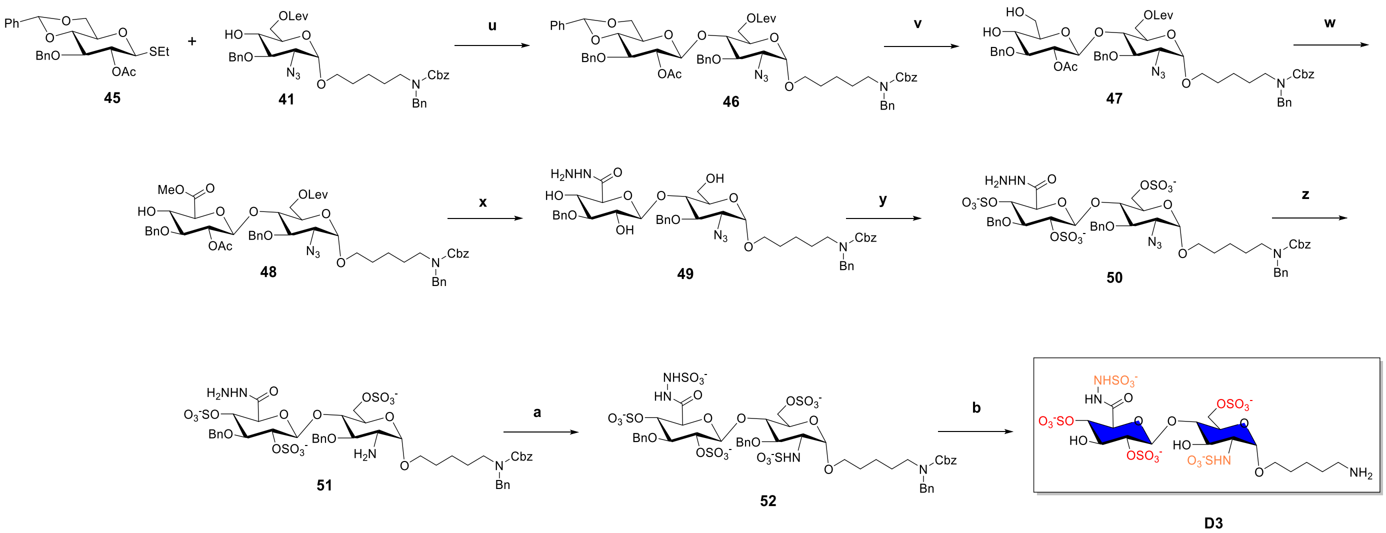

Scheme S9. Synthetic route for **D3**. Reagents and conditions: **(u):** NIS, TMSOTf, molecular sieves (4 Å), DCM, 0°C, 30 min, 82%; **(v):** TFA/H_2O, DCM, R.T., 4h, 53%; **(w): (i):** TEMPO, BAIB, DCM/H_2O, R.T., 3-4h; **(ii):** MeI, $KHCO_3$, DMF, R.T., 4h, 71%; **(x):** N_2H_4.HOAc, MeOH/DCM, R.T., 6h, 65%; **(y):** SO_3.Py, DMF, 60°C, 12h, 84%; **(z):** PMe_3, THF, $NaOH_{(aq)}$, R.T., 2-3h, 69%; **(a):** SO_3.Py, MeOH/Et_3N, $NaOH_{(aq)}$, R.T., 14h, 55%; **(b):** $Pd(OH)_2$/C, MeOH: H_2O, under H_2, R.T., 24h, 76%.

1.2.4.3.1. Synthesis of *N*-benzyl-*N*-benzyloxycarbonyl-5-aminopentyl *O*-(2-*O*-acetyl-3-*O*-benzyl-4,6-*O*-benzylidene-β-D-glucopyranoside)-(1→4)-*O*-2-azido-3-*O*-benzyl-6-*O*-levulinoyl-2-deoxy-D-glucopyranoside (46):

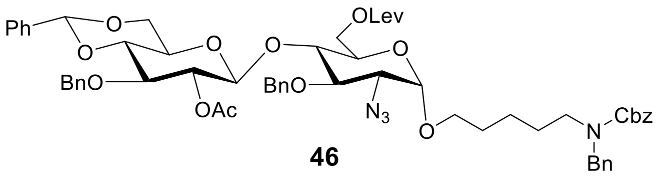

46

For the glycosylation reaction, donor **45** (2.28 g, 5.12 mmol) and acceptor **41** (3.0 g, 4.27 mmol) were dissolved in anhydrous DCM (60 mL) and stirred with pre-activated molecular sieves (4 Å, ~6 g) for 1 hour at R.T. under an argon environment. The reaction mixture was then cooled down to 0 °C, followed by the addition of promoters, NIS (1.44 g, 6.4 mmol) and TMSOTf (78.2

μL, 0.43 mmol). The reaction mixture was stirred at 0 °C for half an hour. After the completion, it was quenched with Et_3N (~500 μL). The mixture was diluted with DCM and washed with $Na_2S_2O_{3(aq)}$ to decolorize, brine, and H_2O. The organic phase was dried over $MgSO_4$, filtered, and concentrated under reduced pressure. The residue was purified by silica gel column chromatography (EtOAc/Tol, 0 to 70%, v/v) to yield the compound **46** (4.59 g, 4.23 mmol, 82%).

^{1}H-NMR (600 MHz, $CDCl_3$) δ 7.53 (d, *J* = 2.1 Hz, 1H), 7.54 – 7.45 (m, 3H), 7.48 – 7.36 (m, 10H), 7.38 – 7.28 (m, 9H), 7.27 (s, 1H), 7.20 (d, *J* = 7.4 Hz, 1H), 5.79 (t, *J* = 9.0 Hz, 1H), 5.64 (s, 1H), 5.49 (s, 1H), 5.23 (d, *J* = 9.2 Hz, 1H), 5.18 (s, 1H), 5.09 – 5.01 (m, 2H), 4.91 (dd, *J* = 23.9, 12.0 Hz, 2H), 4.83 (d, *J* = 17.8 Hz, 1H), 4.80 – 4.63 (m, 4H), 4.53 (d, *J* = 9.6 Hz, 2H), 4.34 (dd, *J* = 10.5, 5.0 Hz, 1H), 4.30 (s, 2H), 4.15 – 4.07 (m, 1H), 3.95 (t, *J* = 9.4 Hz, 1H), 3.91 (s, 1H), 3.89 – 3.75 (m, 4H), 3.71 (t, *J* = 9.3 Hz, 1H), 3.53 (dtd, *J* = 26.2, 9.7, 5.0 Hz, 2H), 3.39 – 3.31 (m, 2H), 3.30 (s, 1H), 3.24 (s, 1H), 2.91 – 2.81 (m, 1H), 2.76 – 2.58 (m, 5H), 2.53 (ddd, *J* = 17.1, 6.5, 4.8 Hz, 1H), 2.18 (s, 2H), 2.10 (d, *J* = 6.3 Hz, 1H), 2.04 (s, 2H), 1.93 (s, 3H), 1.65 (s, 1H), 1.37 (s, 1H), 1.31 (s, 1H).

HRMS (ESI/QTOF) m/z: $[M + Na]^+$ Calculated for $C_{60}H_{68}N_4NaO_{15}^+$ 1107.4573; Found 1107.4592.

1.2.4.3.2. Synthesis of *N*-benzyl-*N*-benzyloxycarbonyl-5-aminopentyl *O*-(2-*O*-acetyl-3-*O*-benzyl-β-D-glucopyranoside)-(1→4)-*O*-2-azido-3-*O*-benzyl-6-*O*-levulinoyl-2-deoxy-D-glucopyranoside (47):

For the ring-opening reaction of the disaccharide, compound **46** (4.59 g, 4.23 mmol) was dissolved in DCM (50 mL). TFA (5 mL) and H_2O (500 μL) were added, and the reaction mixture

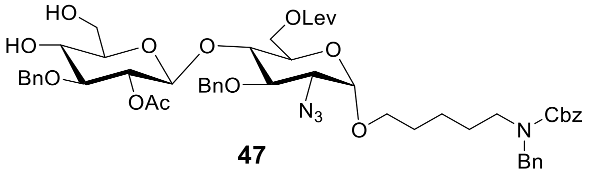

47

was stirred at R.T. for 4 hours. Upon completion, it was diluted with DCM, washed with H_2O. The aqueous layer was extracted with DCM. The organic layer was dried over $MgSO_4$, filtered, and concentrated under reduced pressure. The residue was purified by silica gel column chromatography (EtOAc/Tol, 0 to 50%, v/v) to yield the compound **47** (2.2 g, 2.2 mmol, 53%).

1.2.4.3.3. Synthesis of *N*-benzyl-*N*-benzyloxycarbonyl-5-aminopentyl-*O*-(methyl-2-*O*-acetyl-3-*O*-benzyl-β-D-glucopyranoside)-(1→4)-*O*-2-azido-2-deoxy-3-*O*-benzyl-6-*O*-levulinoyl-α-D-glucopyranoside (48):

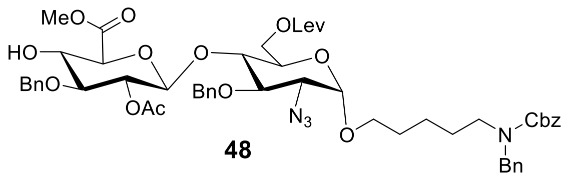

48

For the oxidation reaction, compound **47** (2.2 g, 2.2 mmol) was dissolved in DCM (20 mL) and H_2O (10 mL), followed by the addition of TEMPO (68.8 mg, 0.44 mmol) and BAIB (1.56 g, 4.84 mmol). The reaction mixture was stirred at R.T. for 3-4 hours. Upon completion, the mixture was diluted with DCM and washed with $Na_2S_2O_{3(aq)}$ to decolorize and with H_2O. The organic phase was dried over $MgSO_4$, filtered, and concentrated under reduced pressure. The crude carboxyl compound was directly proceeded to the next step without any purification. For the methylation reaction, crude material was dissolved in anhydrous DMF (30 mL) under an argon environment and followed by the addition of MeI (205 µL, 3.3 mmol) and $KHCO_3$ (550.6 mg, 5.5 mmol). The reaction mixture was stirred at R.T. for 12 hours. After the completion, the mixture was diluted with DCM and washed with ice-cold H_2O. The aqueous phase was extracted with DCM. The organic phase was dried over $MgSO_4$, filtered, and concentrated under reduced pressure. The residue was purified by silica gel column chromatography (EtOAc/Tol, 0 to 40%, v/v) to yield the methylated compound **48** (1.63 g, 1.59 mmol, 71%).

1.2.4.3.4. Synthesis of *N*-benzyl-*N*-benzyloxycarbonyl-5-aminopentyl-(hydrazine-3-*O*-benzyl-β-D-glucopyranoside)-(1→4)-*O*-2-azido-2-deoxy-3-*O*-benzyl-α-D-glucopyranoside (49):

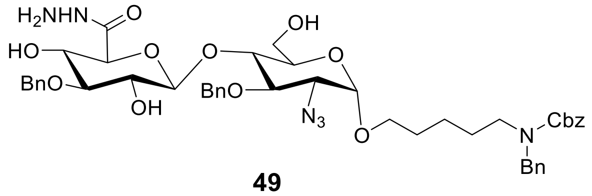

49

Compound **48** (1.63 g, 1.59 mmol) was dissolved in anhydrous MeOH (2 mL) and DCM (18 mL). N_2H_4.HOAc (1.5 g, 16 mmol) was added to the reaction solution, and it was stirred at R.T. for 6 hours. After the completion, it was concentrated under reduced pressure, and the crude material was purified using Sephadex® LH-20 and Milli-Q H_2O as an eluent to yield compound **49** (910 mg, 1.02 mmol, 65%).

1.2.4.3.5. Synthesis of *N*-benzyl-*N*-benzyloxycarbonyl-5-aminopentyl-(hydrazine-2,4-*O*-sulfate-3-*O*-benzyl-β-D-glucopyranoside)-(1→4)-*O*-2-azido-2-deoxy-6-*O*-sulfate-3-*O*-benzyl-α-D-glucopyranoside (50):

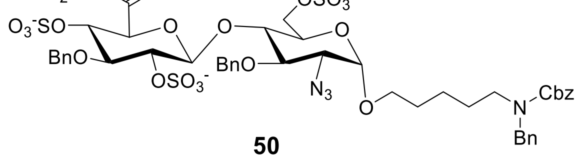

50

For the *O*-sulfation reaction of compound **49** (910 mg, 1.02 mmol), SO_3.Py (4.8 g, 30.6 mmol) was added to the solution of **49** in DMF (10 mL). The reaction mixture was stirred at 60 °C for 12h. After the completion, MeOH/Et_3N (50%, v/v) was added and stirred for half an hour. The reaction mixture was filtered, and the solvents were removed under reduced pressure. The residue was first purified

using Sephadex® LH-20 (MeOH/H_2O, 20 to 80%, v/v). Afterwards, it was passed through an ion-exchange column of Dowex® 50W X8 Na^+ resin using Milli-Q H_2O as an eluent. Fractions having the compound were lyophilized to afford the *O*-sulfated compound **50** (966 mg, 0.86 mmol, 84%).

1.2.4.3.6. Synthesis of *N*-benzyl-*N*-benzyloxycarbonyl-5-aminopentyl-(hydrazine-2,4-*O*-sulfate-3-*O*-benzyl-β-D-glucopyranoside)-(1→4)-*O*-2-amino-2-deoxy-6-*O*-sulfate-3-*O*-benzyl-α-D-glucopyranoside (51):

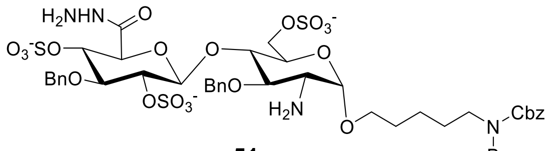

51

To convert azide to amine, Staudinger reduction was followed. Compound **50** (966 mg, 0.86 mmol) was dissolved in THF (10 mL). PMe_3 (654 mg, 8.6 mmol) and then aqueous NaOH solution (0.172 mmol in 5 mL H2O) were added to the reaction mixture. It was stirred at R.T. for 2-3 hours. The residue was purified using Sephadex® LH-20 using Milli-Q H_2O as an eluent. Fractions containing the compound were freeze-dried to give compound **51** (651 mg, 0.59 mmol, 69%).

1.2.4.3.7. Synthesis of *N*-benzyl-*N*-benzyloxycarbonyl-5-aminopentyl-(amino-sulfamino-2,4-*O*-sulfate-3-*O*-benzyl-β-D-glucopyranoside)-(1→4)-*O*-2-sulfamino-2-deoxy-6-*O*-sulfate-3-*O*-benzyl-α-D-glucopyranoside (52):

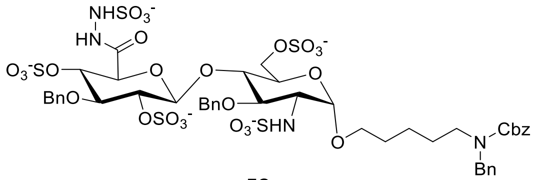

52

For the *N*-sulfation reaction, compound **51** (651 mg, 0.59 mmol) was dissolved in MeOH (10 mL) and Et_3N (3.3 mL) under an argon environment. SO_3.Py (939 mg, 5.9 mmol) was added to the reaction solution. The pH was adjusted to pH 11 by adding $NaOH_{(aq)}$. Three additional portions of SO_3.Py (939 mg, 5.9 mmol) were added to the solution after 30 min, 1 hour, and 2 hours, while the pH was maintained at 11. The reaction mixture was stirred for 12 hours. After the completion, the reaction mixture was co-evaporated with H_2O. The crude material was first purified by a Dowex 50W X8 resin (Na^+ form) ion exchange column with Milli-Q H_2O as an eluent. Fractions having the product were freeze-dried. The residue was dissolved in H_2O and purified by a reverse-phase C18 silica gel column (H_2O/MeCN, from 98/2 to 85/15, v/v). The portions having the compound were lyophilized to yield the compound **52** (410 mg, 0.33 mmol, 55%).

1.2.4.3.8. Synthesis of 5-aminopentyl-(amino-sulfamino-2,4-*O*-sulfate-β-D-glucopyranoside)-(1→4)-*O*-2-sulfamino-2-deoxy-6-*O*-sulfate-α-D glucopyranoside (D3):

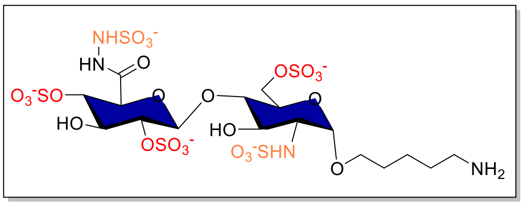

D3

For the debenzylation reaction, compound **52** (410 mg, 0.33 mmol) and 20% $Pd(OH)_2/C$ (615 mg, 1.5x of compound **52** by weight) were dispersed in MeOH/H_2O (50%, v/v) under a hydrogen environment and stirred at R.T. for 24 hours. The next day, the reaction mixture was filtered over a pad of celite, and the residue was washed with H_2O. After removing H_2O, the crude product was purified by size exclusion chromatography using a P-2 Gel column with Milli-Q H_2O as an eluent. The fractions having the product were freeze-dried to yield the final disaccharide (**D3**) (211 mg, 0.25 mmol, 76%).

1.2.5. T1

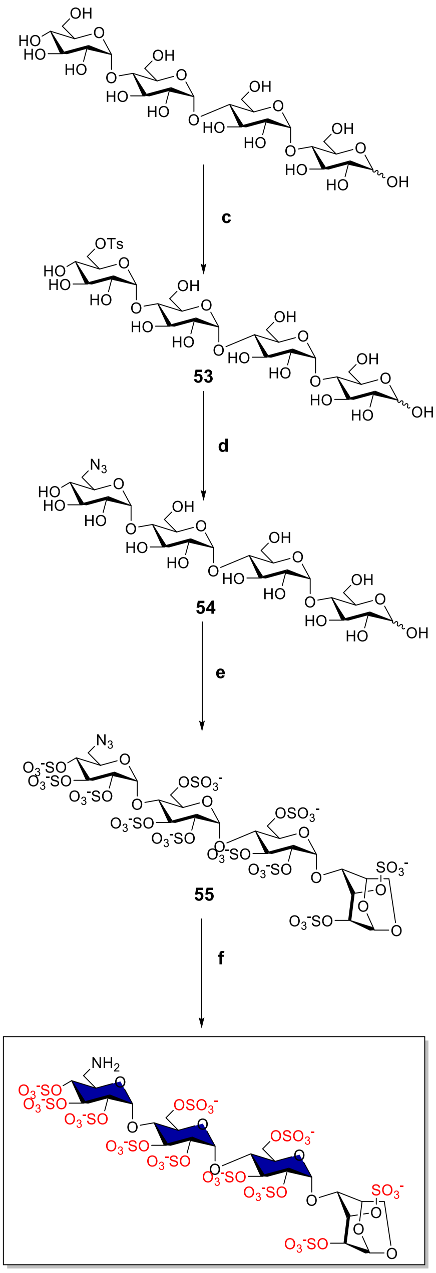

T1

Scheme S10. Synthetic route for **T1**. Reagents and conditions: **(c):** TsCl, Py, DCM, 0 °C to R.T., 18h, 80%; **(d):** NaN_3, DMF, 60 °C, 24h; **(e):** $SO_3.Et_3N$, DMF, 120 °C, 10 min; **(f):** $Pd(OH)_2/C$, $MeOH:H_2O$, under H_2, R.T., 24h, 35% overall.

1.2.5.1. Synthesis of 53

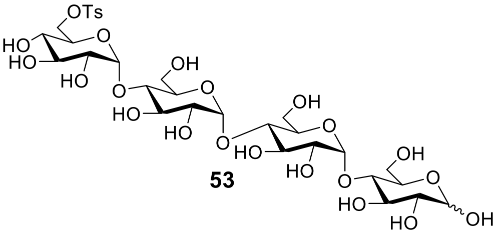

53

For the tosylation reaction, maltotetraose (1.0 g, 1.5 mmol) was dissolved in anhydrous DCM (10 mL) under an argon environment. Py (~500 µL) was added, and the reaction mixture was cooled down to 0 °C. TsCl (429 mg, 2.25 mmol) was slowly added, and the reaction was allowed to warm up to R.T. and stirred for 18 hours. Upon completion,

the reaction mixture was directly concentrated under reduced pressure to get crude compound **53.**

^{1}H-NMR (400 MHz, DMF) δ 9.04 – 8.97 (m, 2H), 8.56 (tt, *J* = 7.8, 1.6 Hz, 1H), 8.10 – 8.01 (m, 2H), 7.73 – 7.66 (m, 1H), 7.31 – 7.13 (m, 1H), 6.20 (s, 6H), 5.19 – 5.03 (m, 1H), 3.89 – 3.67 (m, 2H), 3.53 – 3.32 (m, 1H), 2.32 (s, 1H).

HRMS (ESI/QTOF) m/z: [M + Na]$^+$ Calculated for $C_{31}H_{48}NaO_{23}S^+$ 843.2199; Found 843.2175.

1.2.5.2. Synthesis of 54

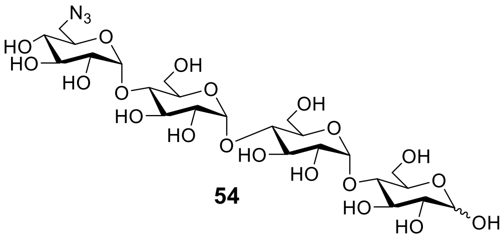

54

To convert OTs to N_3, the crude **53** was dissolved in anhydrous DMF (20 mL). NaN_3 (146 mg, 2.25 mmol) was added, and the reaction mixture was stirred at 60°C for 24 hours. Upon completion, the reaction mixture was concentrated under reduced pressure and dried. The crude product was purified by size exclusion chromatography using a P-2 Gel column, using Milli-Q H_2O as an eluent. The fractions of the product were freeze-dried to yield compound **54**.

^{1}H-NMR (400 MHz, D_2O) δ 7.78 (d, *J* = 8.2 Hz, 1H), 7.65 – 7.58 (m, 6H), 7.45 (t, *J* = 7.0 Hz, 1H), 7.29 (d, *J* = 8.3 Hz, 6H), 5.35 – 5.22 (m, 5H), 5.15 (d, *J* = 3.8 Hz, 1H), 4.57 (d, *J* = 8.0 Hz, 1H), 4.31 (s, 1H), 3.88 (td, *J* = 9.4, 4.7 Hz, 4H), 3.76 (s, 9H), 3.87 – 3.57 (m, 9H), 3.57 (s, 2H), 3.57 – 3.48 (m, 5H), 3.52 – 3.43 (m, 1H), 3.34 (t, *J* = 9.4 Hz, 2H), 3.19 (dd, *J* = 9.5, 8.0 Hz, 1H), 2.64 (s, 5H), 2.42 – 2.37 (m, 1H), 2.32 (s, 10H).

HRMS (ESI/QTOF) m/z: [M + K]$^+$ Calculated for $C_{24}H_{41}KN_3O_{20}^+$ 730.6883; Found 730.2731.

1.2.5.3. Synthesis of 55

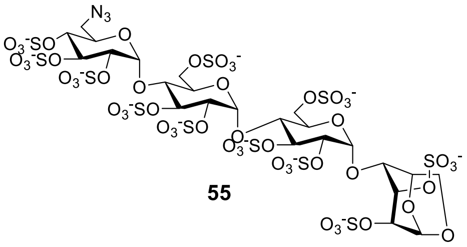

55

For the *O*-sulfation reaction, crude compound **54** was dissolved in anhydrous DMF (30 mL) in an argon atmosphere and $SO_3.Et_3N$ (8.3 g, 60 mmol) was added. The reaction mixture was stirred at 120°C for 10 minutes. After completion, it was concentrated in vacuo and dried. The crude product was purified by size exclusion chromatography using a P-2 Gel column and Milli-Q H_2O as an eluent. The fractions containing the product were freeze-dried to yield compound **55**.

1.2.5.4. Synthesis of T1

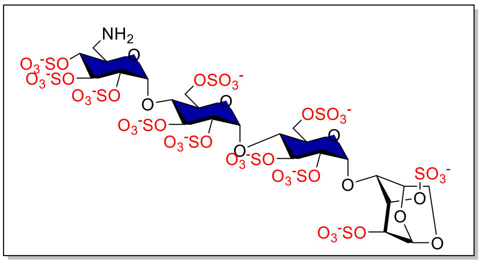

T1

For the conversion of azido to amino, compound **55** and 20% $Pd(OH)_2/C$ (1.3 g, 1.5x of compound 55 by weight) were dispersed in $MeOH/H_2O$ (50%, v/v) under a hydrogen environment and stirred at R.T. for 24 hours. The next day, the reaction mixture was filtered, and the residue was washed with H_2O. After removing water, the crude product was purified by size exclusion chromatography using a P-2 Gel column using Milli-Q H_2O as an eluent. The fractions of the product were freeze-dried to yield the final tetrasaccharide (**T1**) (770 mg, 0.50 mmol, 35% overall yield).

1.3. Materials

Heparin sodium salt (or unfractionated heparin (UFH)) was purchased from Biosynth (CAS # [9041-08-1]). Heparan sulfate sodium salt from bovine kidney was purchased from Merck HS (CAS # [57459-72-0]). Hexamer (**H1**) used in BLI competition assays was purchased from Glycan Therapeutics (CAS # GT24-NH2-023). Fondaparinux sodium, used as a clinical standard for determining the anticoagulant properties, was purchased from Biosynth (CAS # [114870-03-0]). Recombinant proteins, including FGF-1 (CAS # 10013-HNAE), FGF-2 (CAS # 10014-HNAE), and NGF (CAS # 11050-HNAC), were purchased from Sino Biological, Inc., and AT-III (CAS # SAE0102-25UG) was purchased from Merck.

2. Molecular Modelling

The molecular docking of our HS glycomimetics to the selected protein targets was performed using AutoDock 4.2. The 2D structures of our compounds were initially drawn using ChemDraw Ultra 14.0 and subsequently converted into PDB format using Chem3D. The corresponding 3D structures were subjected to geometry optimization via energy minimization using the MM2 force field with 10,000 iterations at a time step of 2 femtoseconds. The structures were further energy-minimized using the hybrid B3LYP functional (6-31G(d) basis set). The conformationally optimized structures were then employed for molecular docking studies. Since no experimental/starting coordinates were available, AutoDock 4.2 was used to dock our HS glyomimetics to selected protein targets. The receptor structures were retrieved from the RCSB Protein Data Bank, specifically: FGF-1 (PDB ID: 2AXM), FGF-2 (PDB ID: 1BFB), and NGF (PDB ID:6YW8). Atomic charges were assigned using AutoDockTools, and the grid box was centered on the protein, with a grid spacing of 0.375 Å. The Lamarckian Genetic Algorithm (LGA) was used for docking following standard protocols. Each docking run included 2,500,000 energy evaluations, a population size of 150, a mutation rate of 0.02, and allowed up to 27,000 generations. A total of 50 independent docking runs were conducted to explore favorable binding conformations. The resulting poses were clustered based on a root-

mean-square deviation (RMSD) threshold of 0.5 Å. Final visualization and analysis of binding conformations were performed using Autodock tools, VMD, and UCSF Chimera.

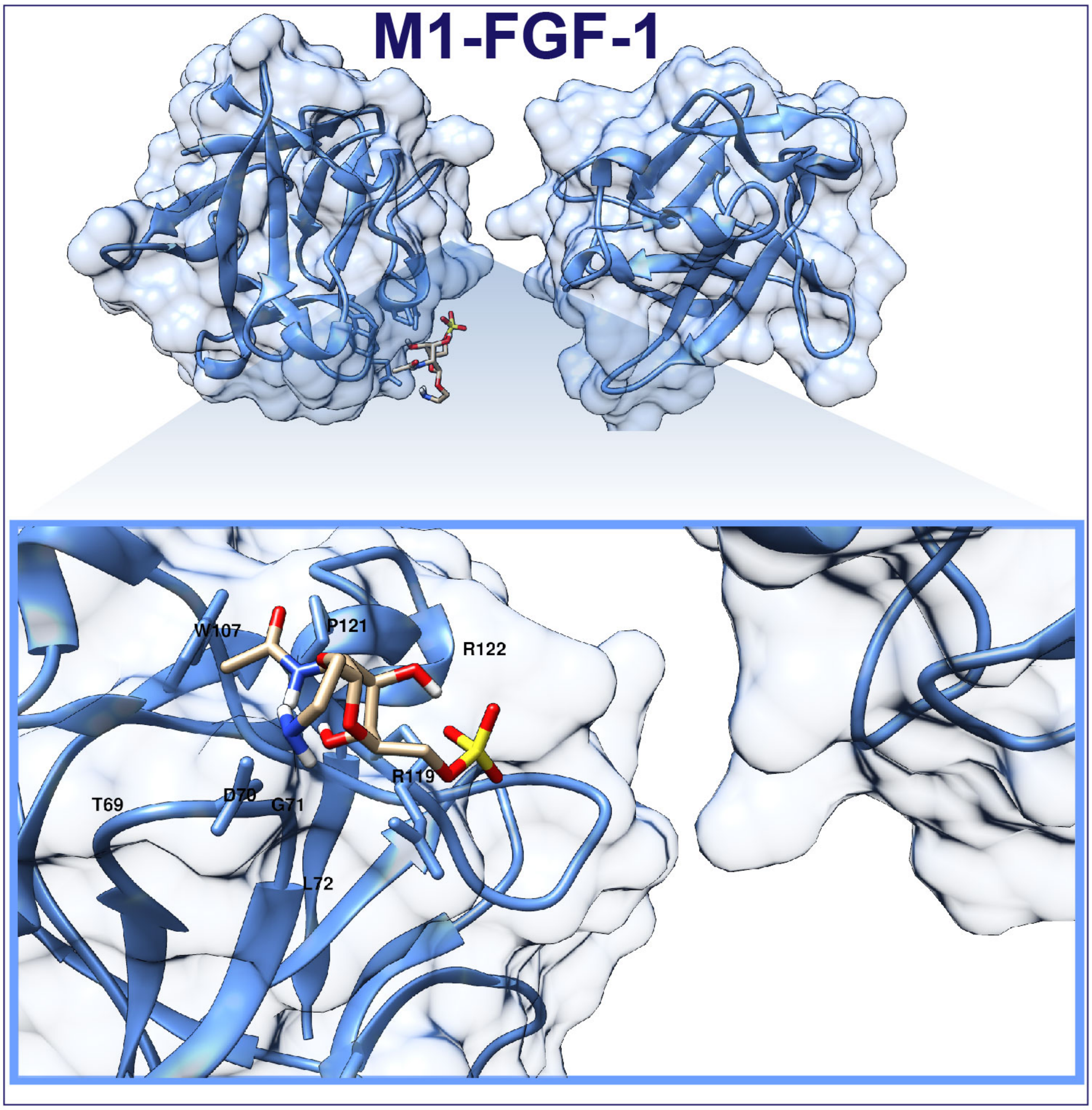

Figure S2.1. M1-FGF-1

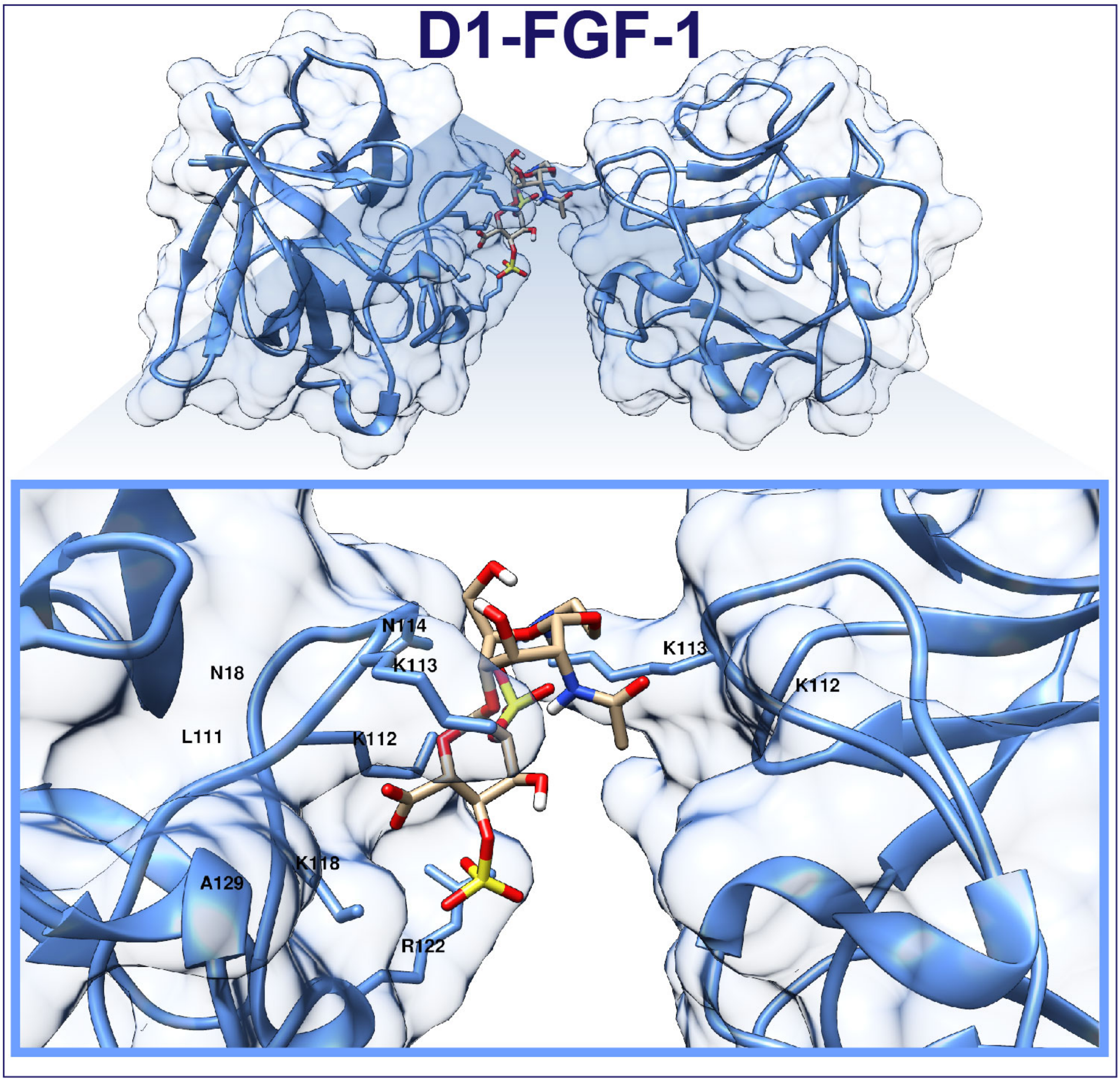

Figure S2.2. D1-FGF-1

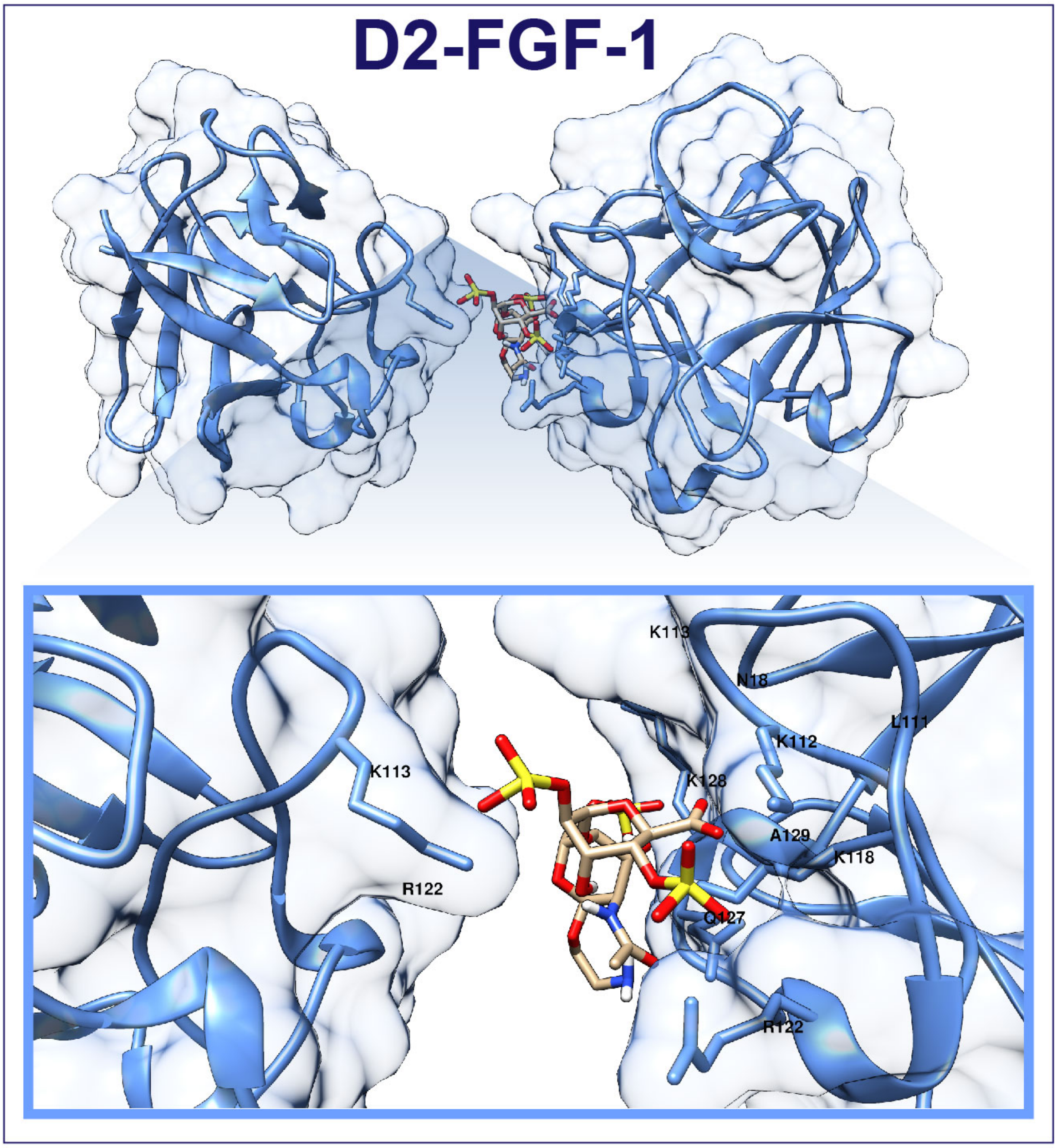

Figure S2.3. D2-FGF-1

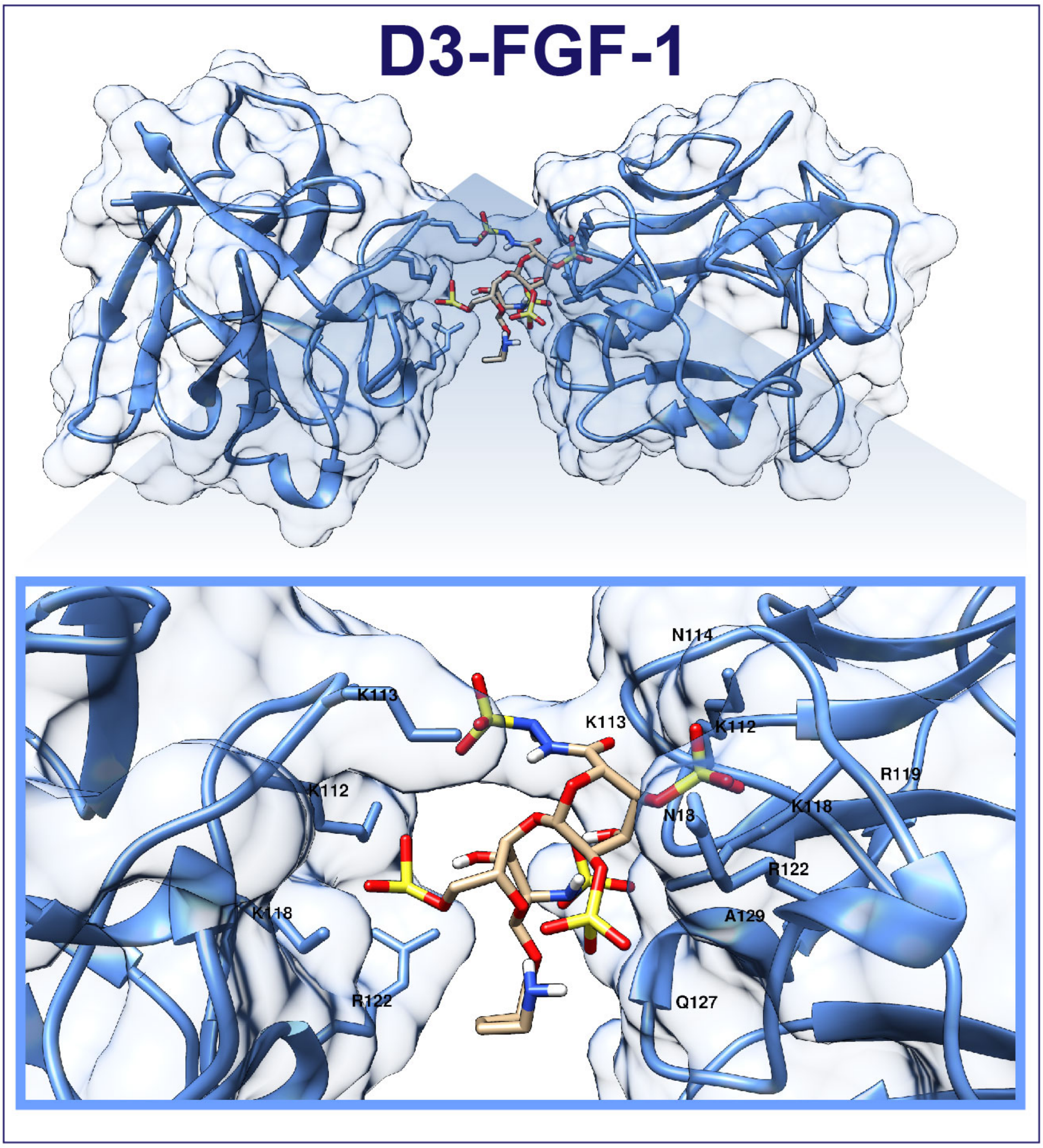

Figure S2.4. D3-FGF-1

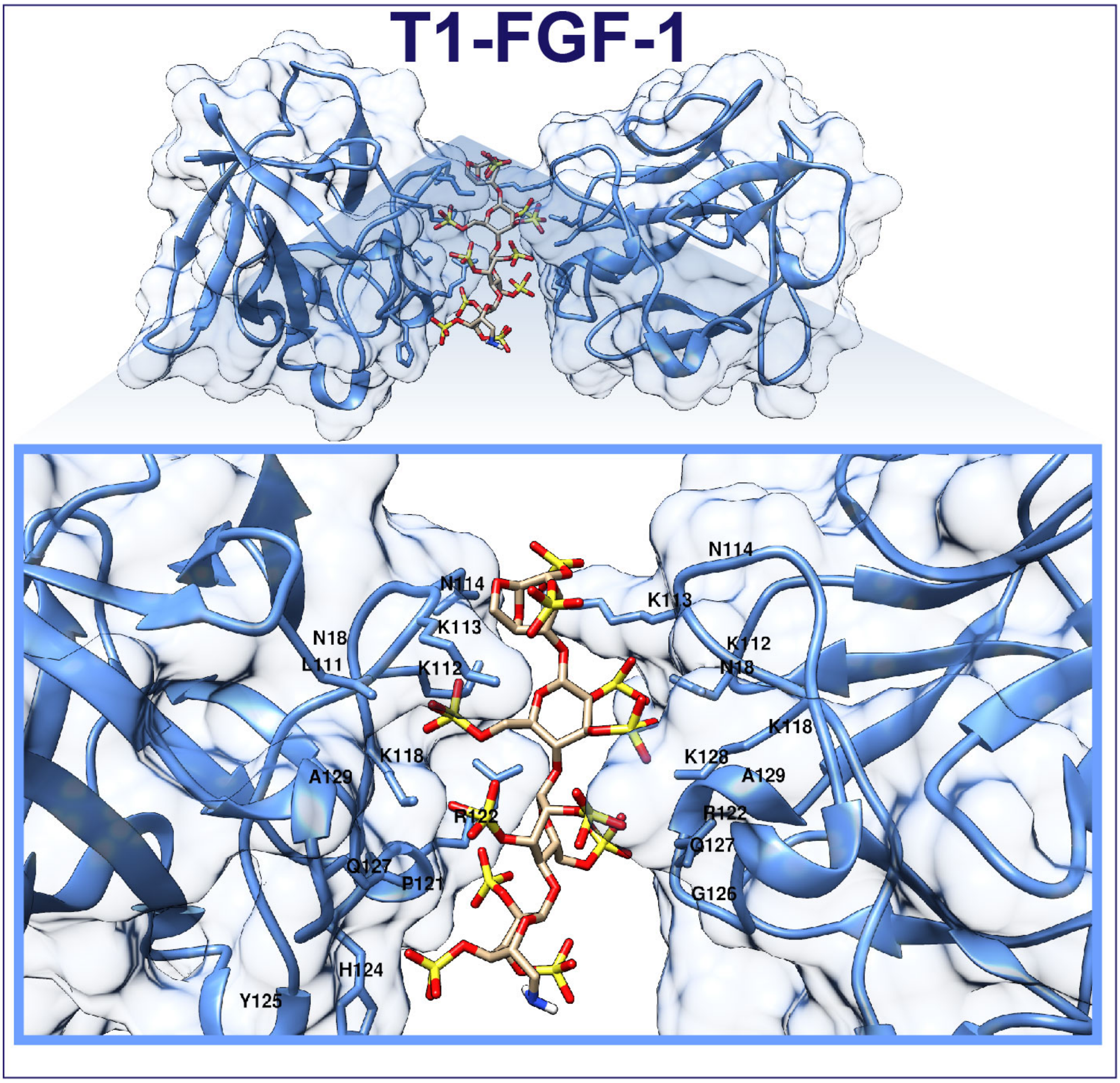

Figure S2.5. T1-FGF-1

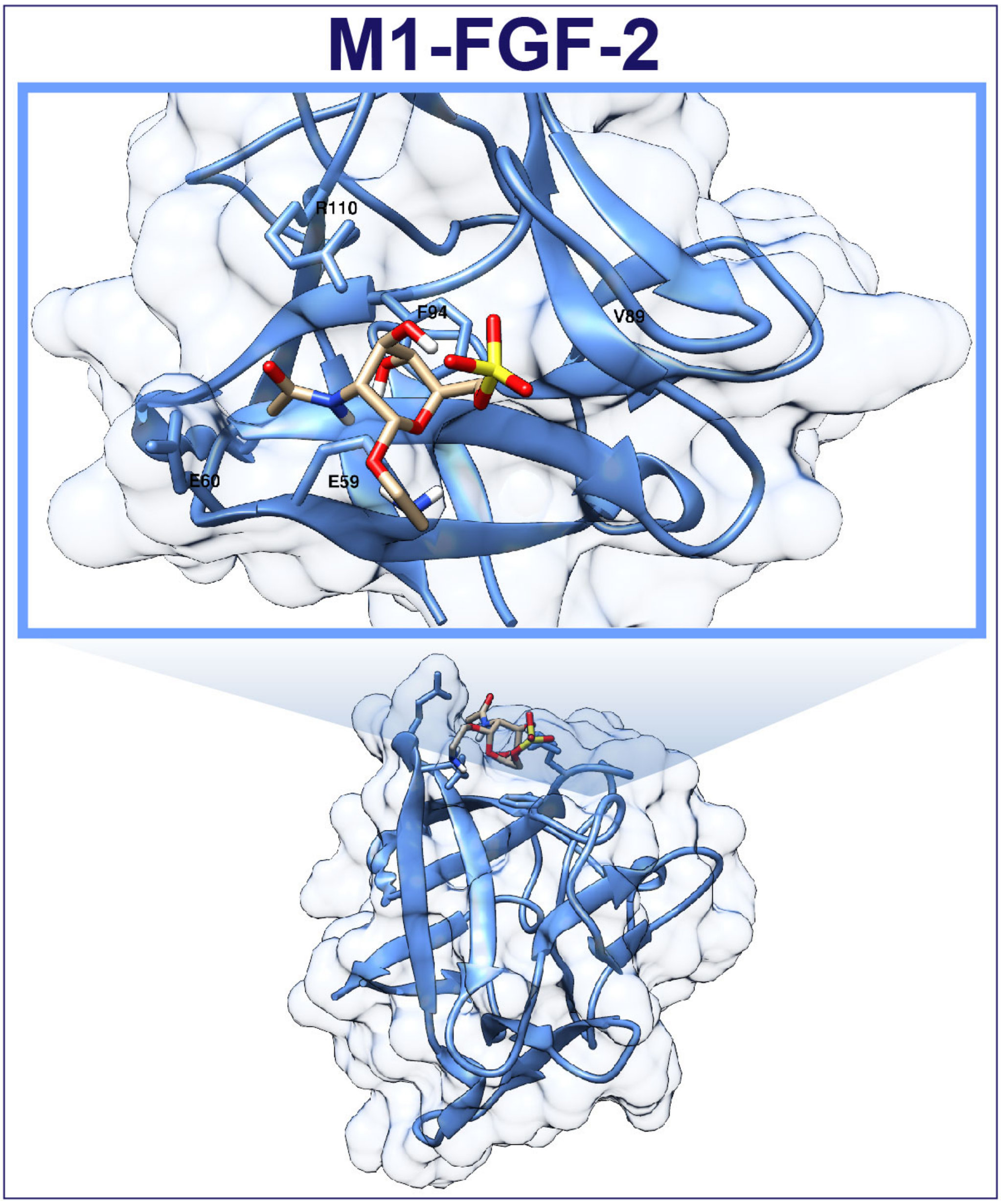

Figure S2.6. M1-FGF-2

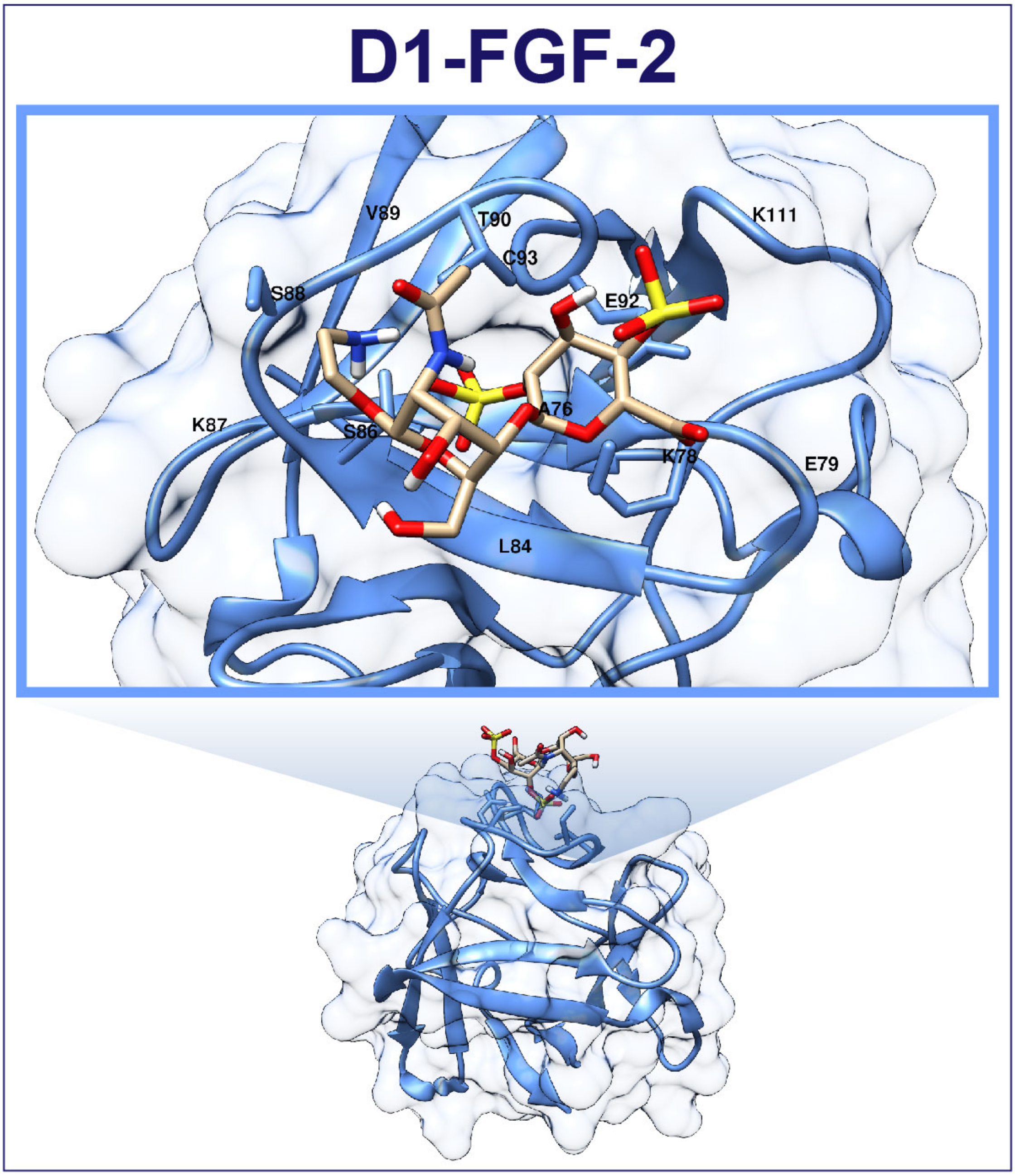

Figure S2.7. D1-FGF-2

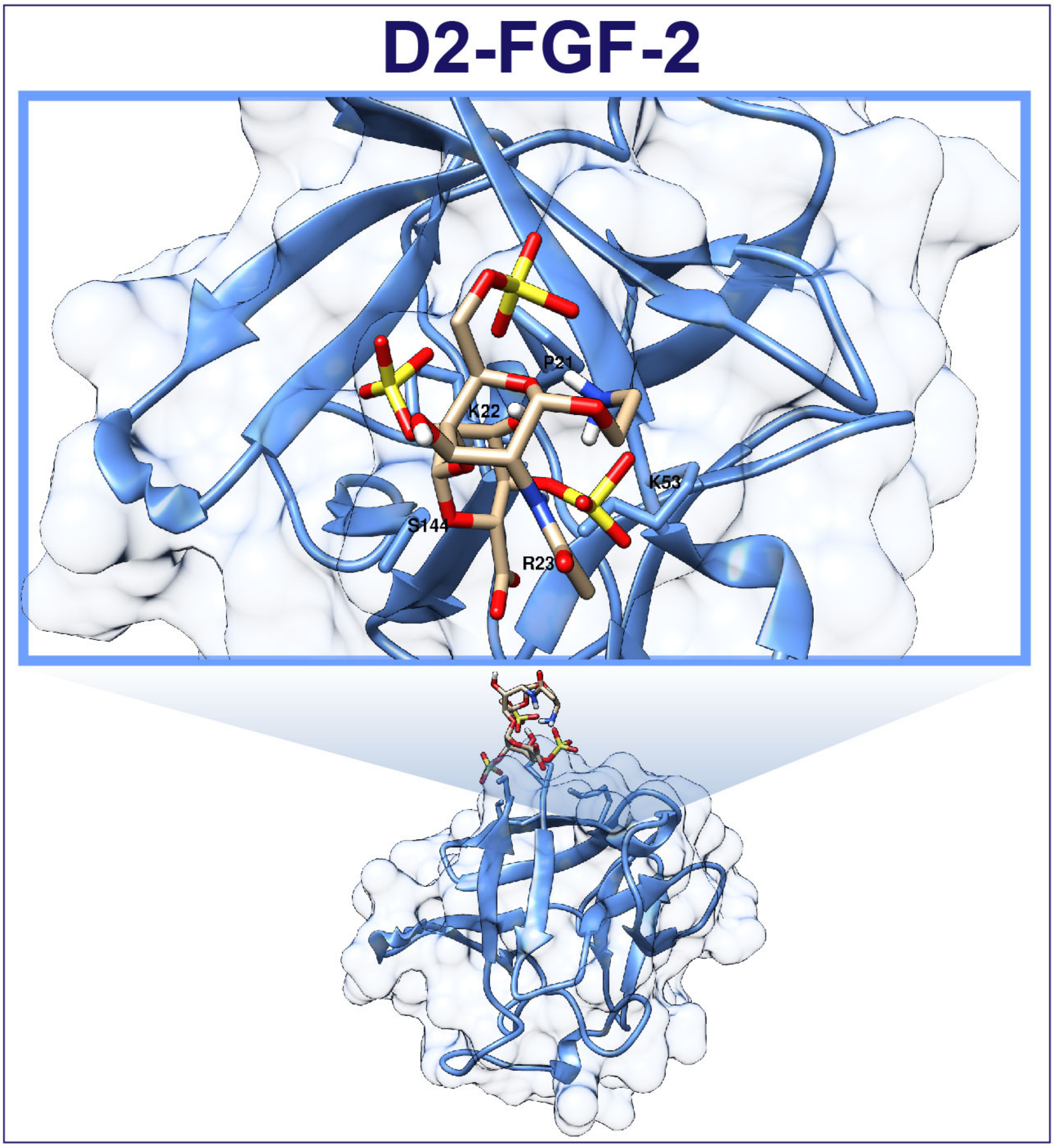

Figure S2.8. D2-FGF-2

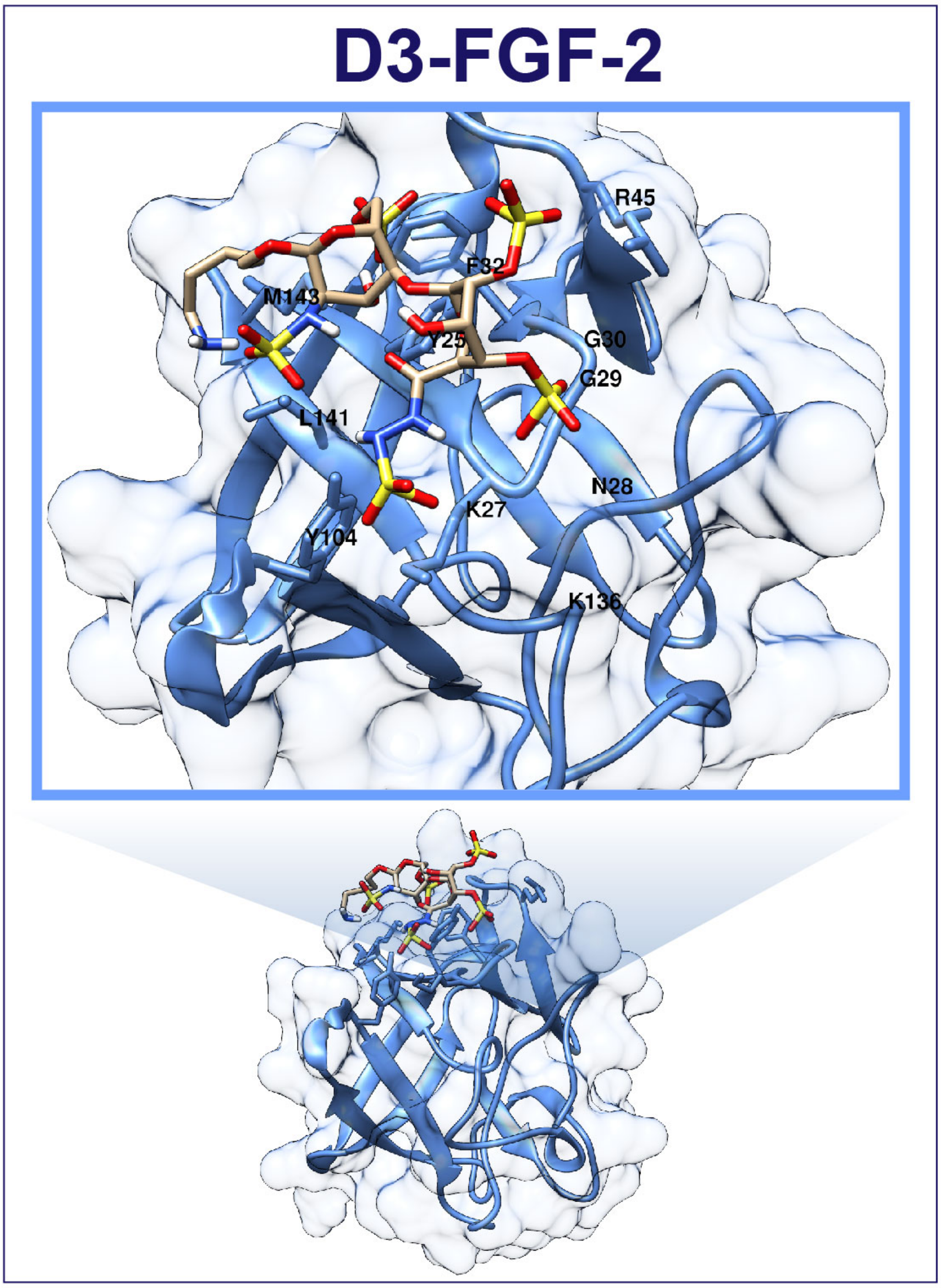

Figure S2.9. D3-FGF-2

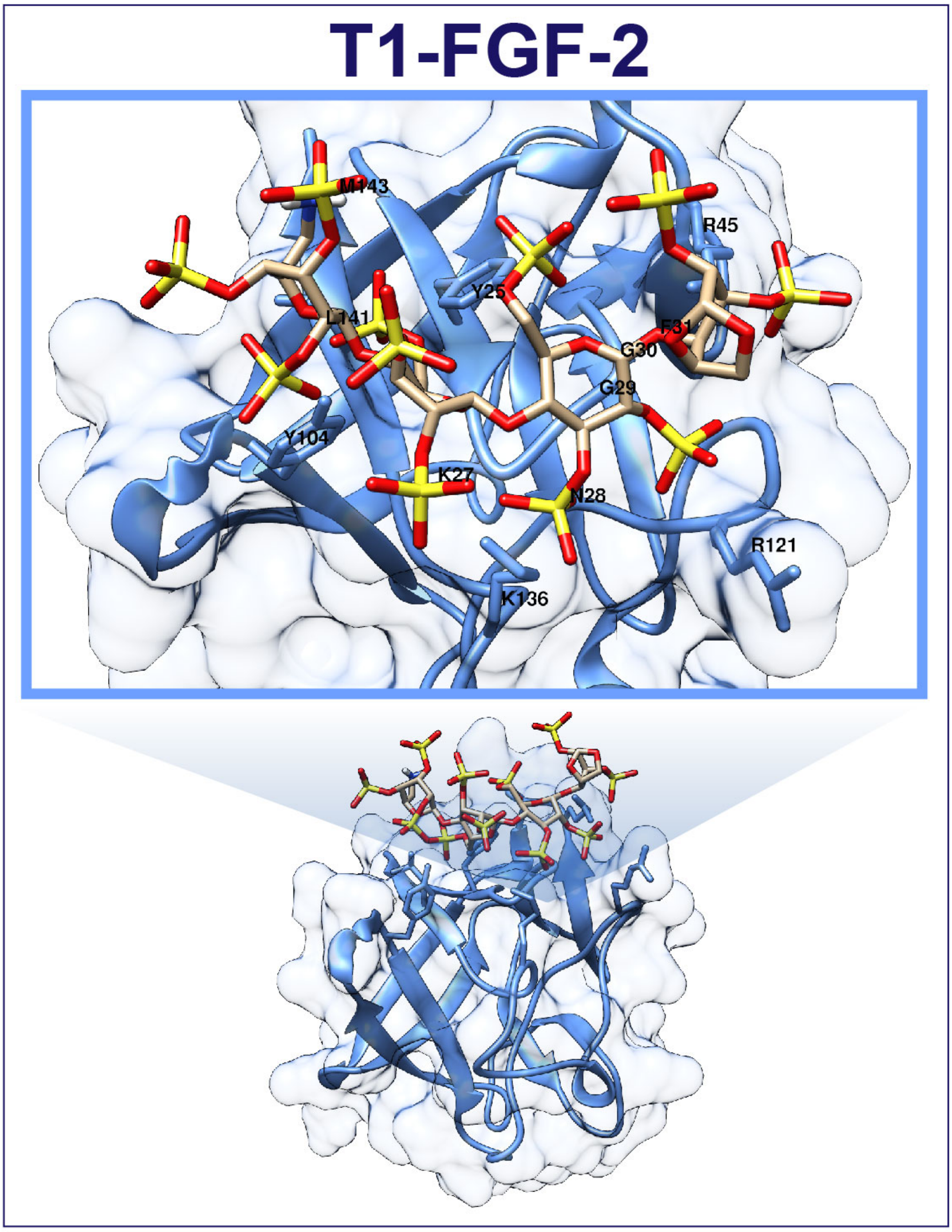

Figure S2.10. T1-FGF-2

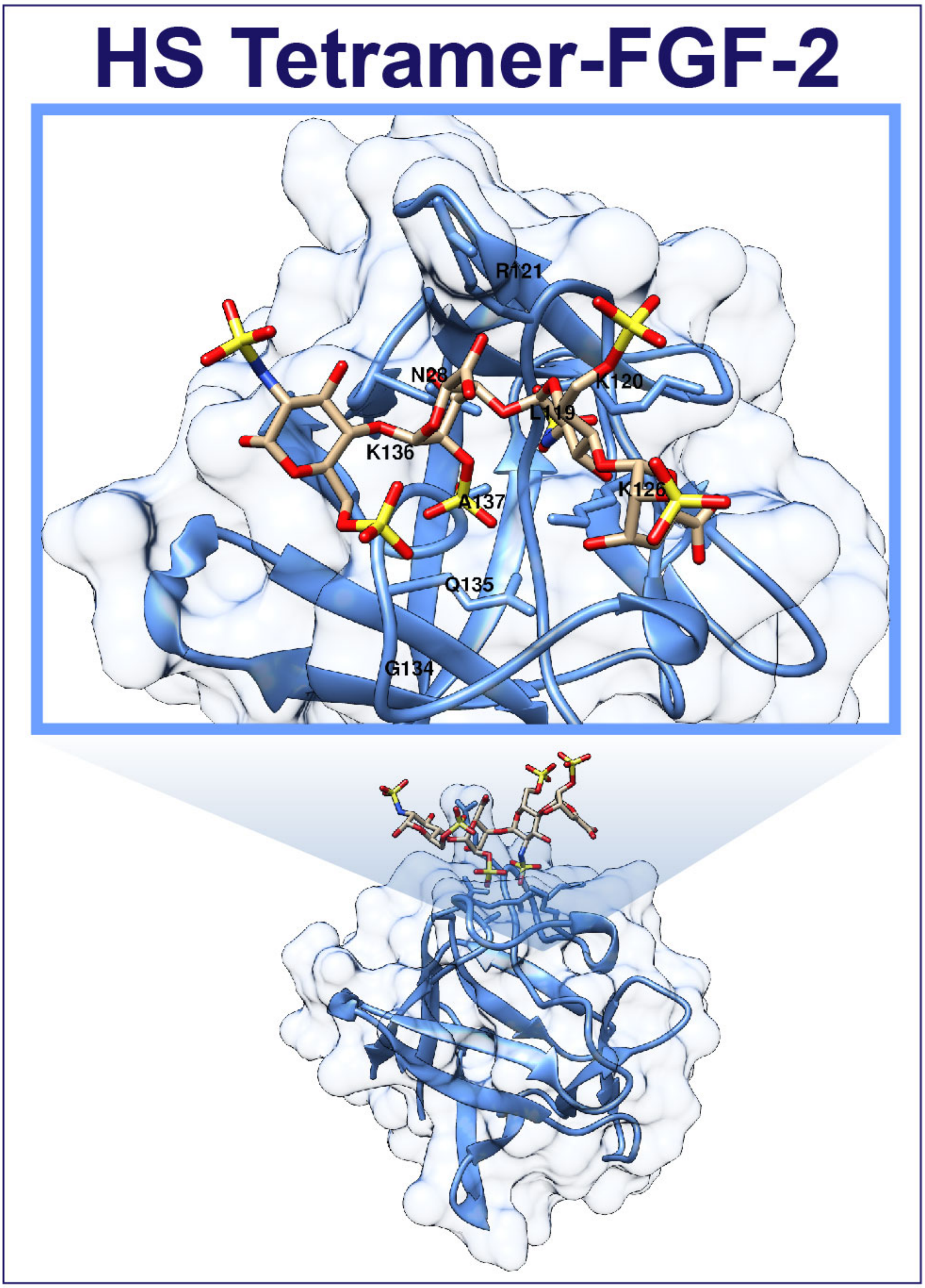

Figure S2.11. HS Tetramer-FGF-2

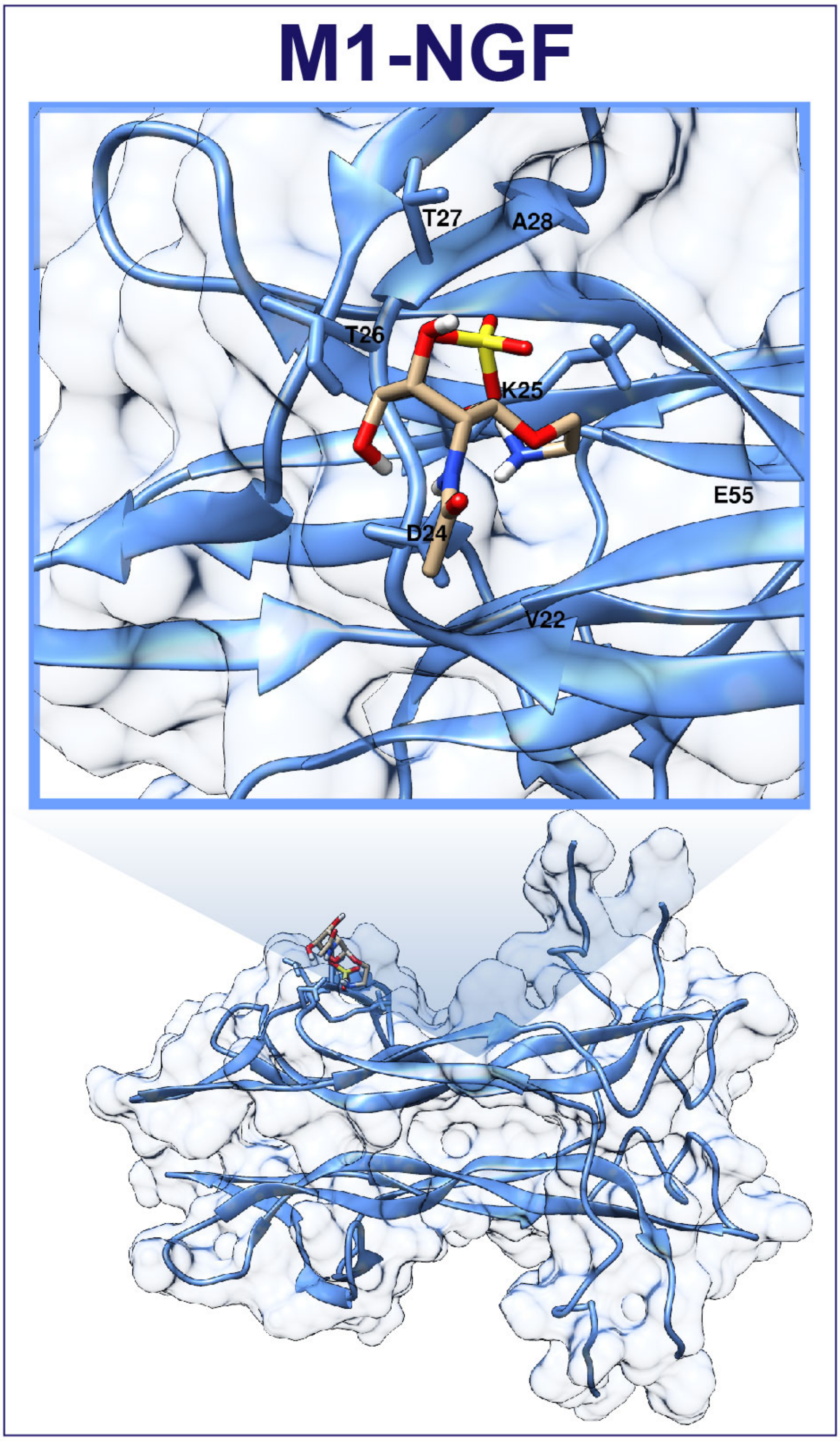

Figure S2.12. M1-NGF

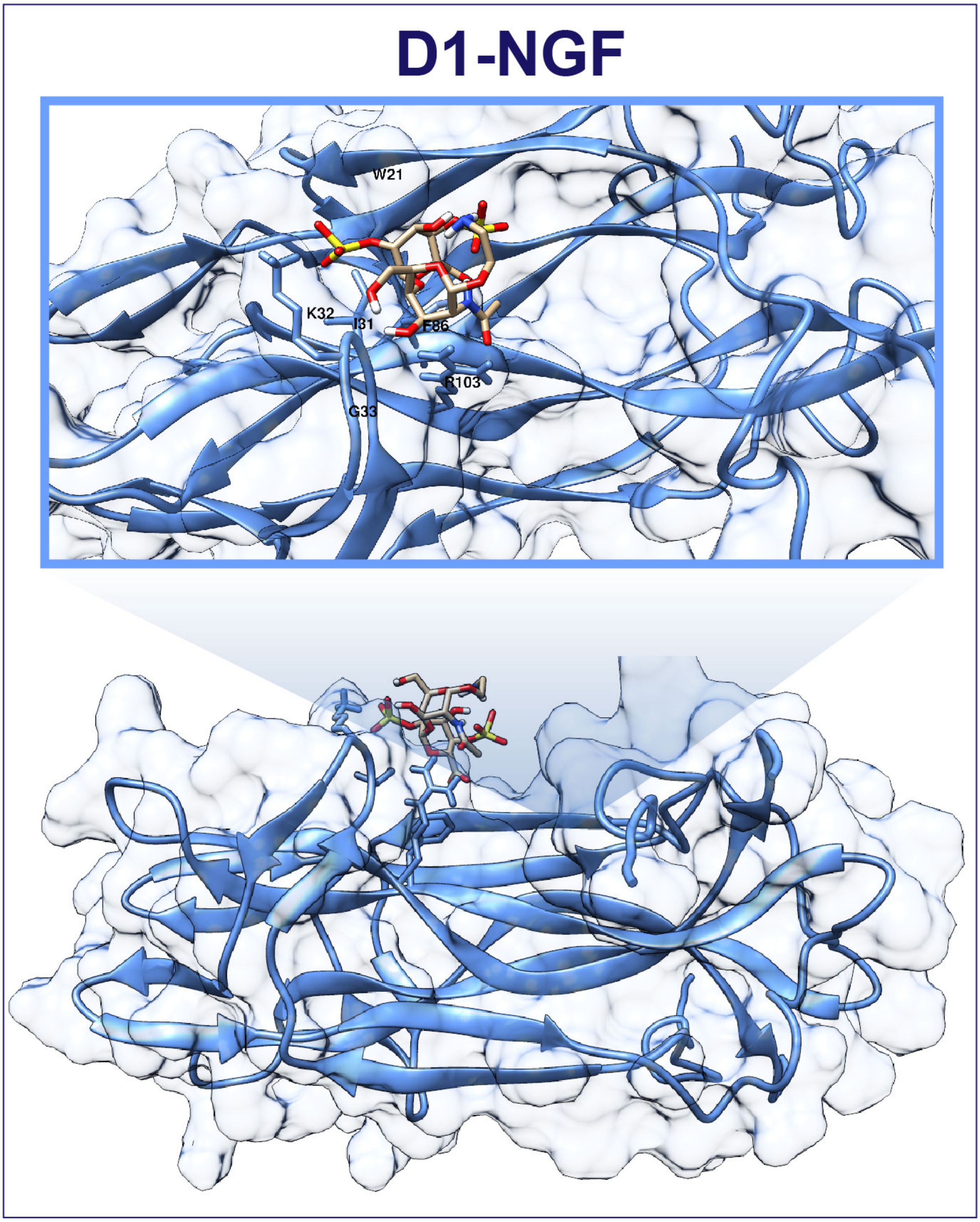

Figure S2.13. D1-NGF

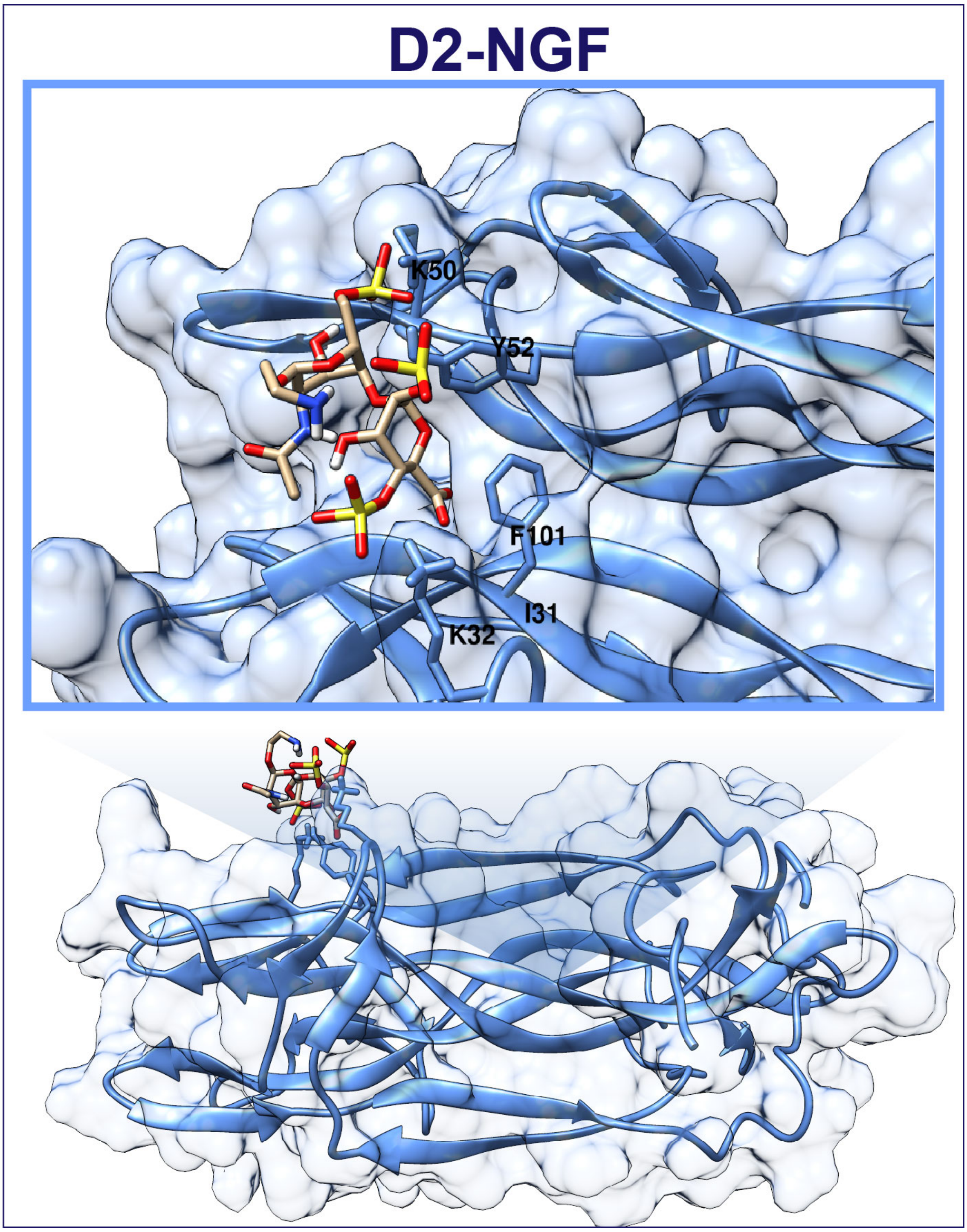

Figure S2.14. D2-NGF

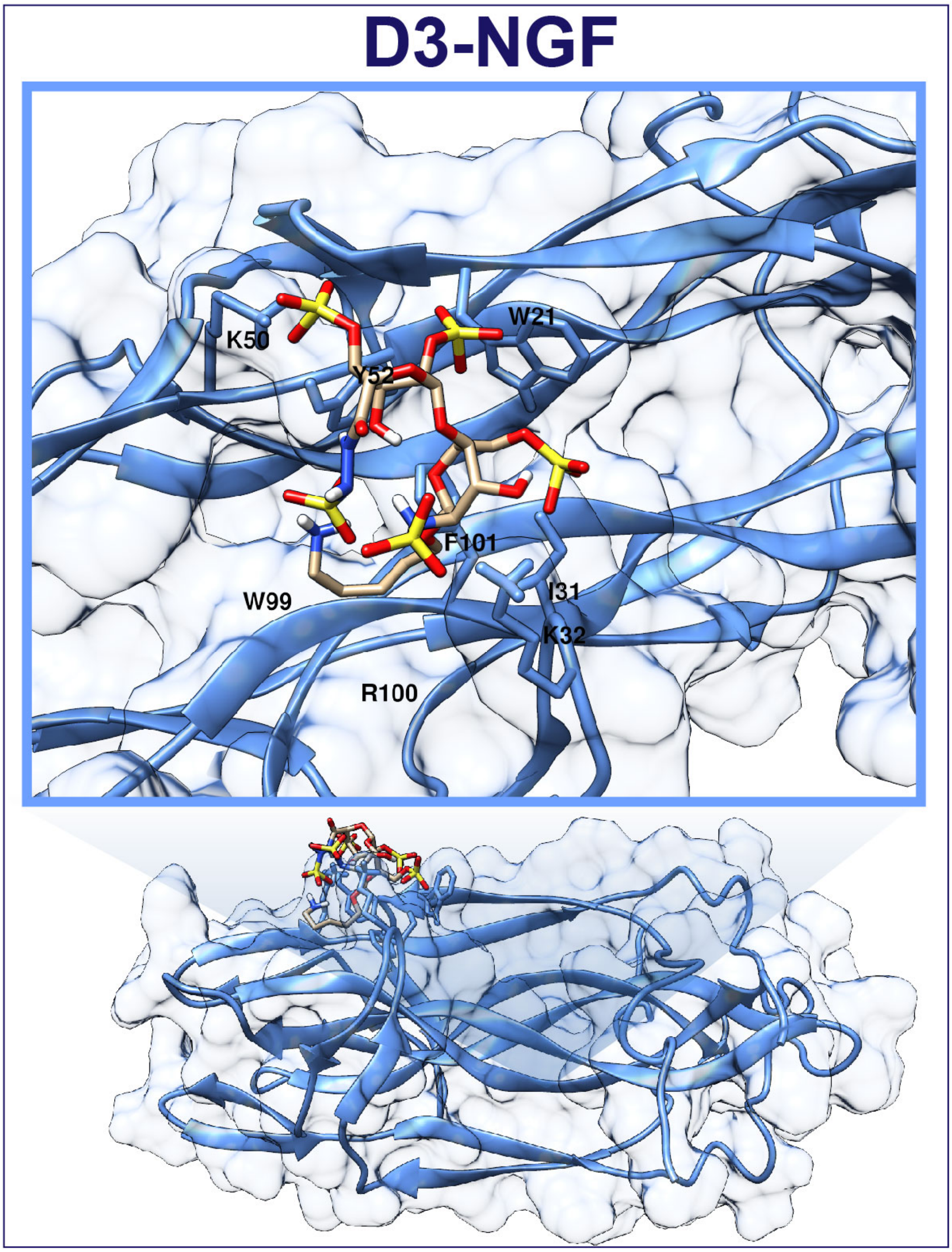

Figure S2.15. D3-NGF

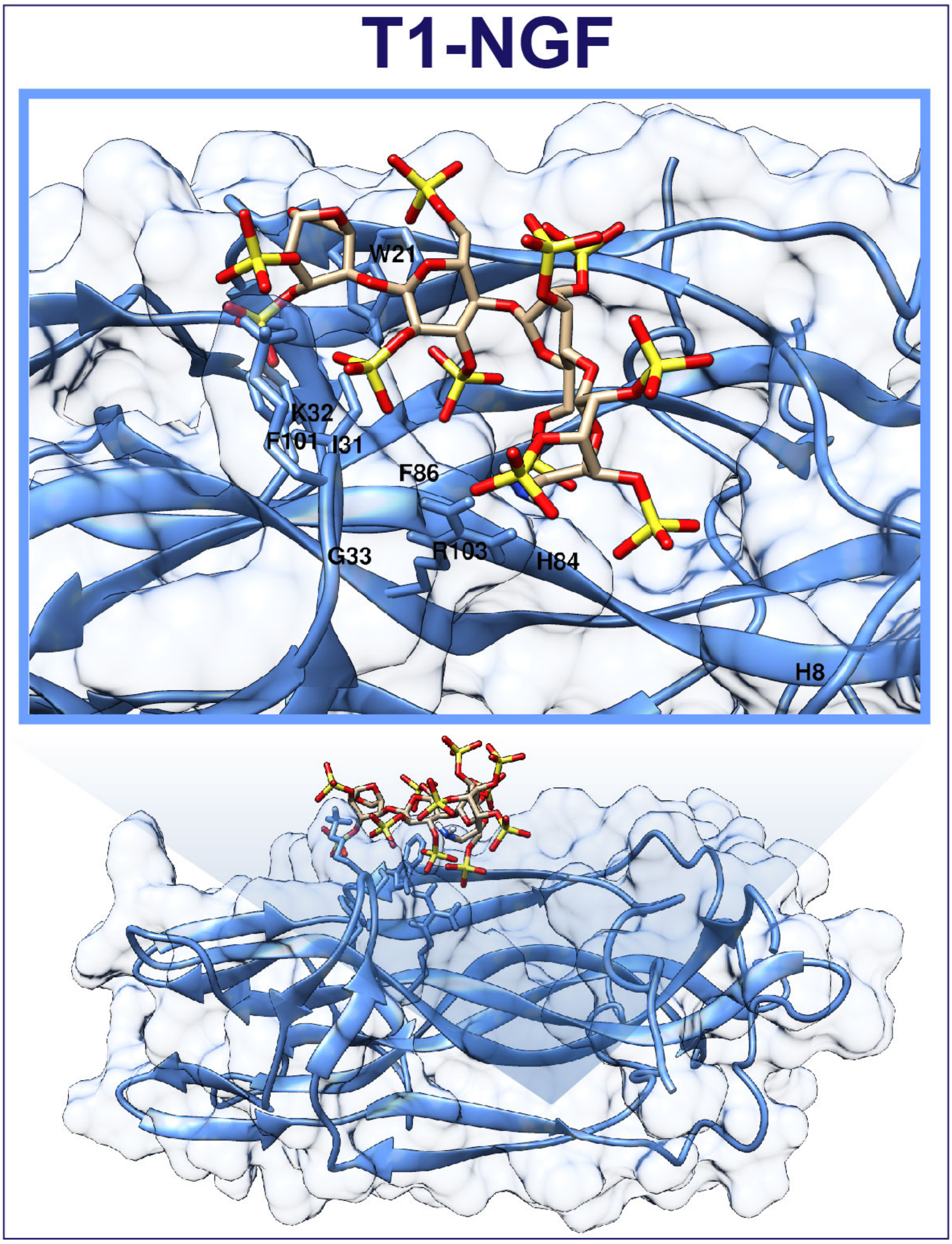

Figure S2.16. T1-NGF

3. Circular Dichroism (CD) Experiments

The circular dichroism (CD) spectra of the samples were recorded using a Chirascan V100 CD spectrophotometer (Applied Photophysics, Leatherhead, UK) at 24.5 °C. Protein solutions (5.0 μM) were prepared in 50 mM Tris-buffered saline (TBS), pH 7.4, and incubated with 15.0 μM of each sugar for 1 hour at 37 °C with shaking at 300 rpm prior to measurement. Far-UV spectra were collected using a 1 mm path length quartz cuvette under the following conditions: a scan rate of 100 nm/min, a bandwidth of 1 nm, and averaging of three consecutive measurements. For thermal denaturation experiments, CD data were acquired from 10 °C to 90 °C, with a temperature increment of 2 °C and a heating rate of 1 °C /min.

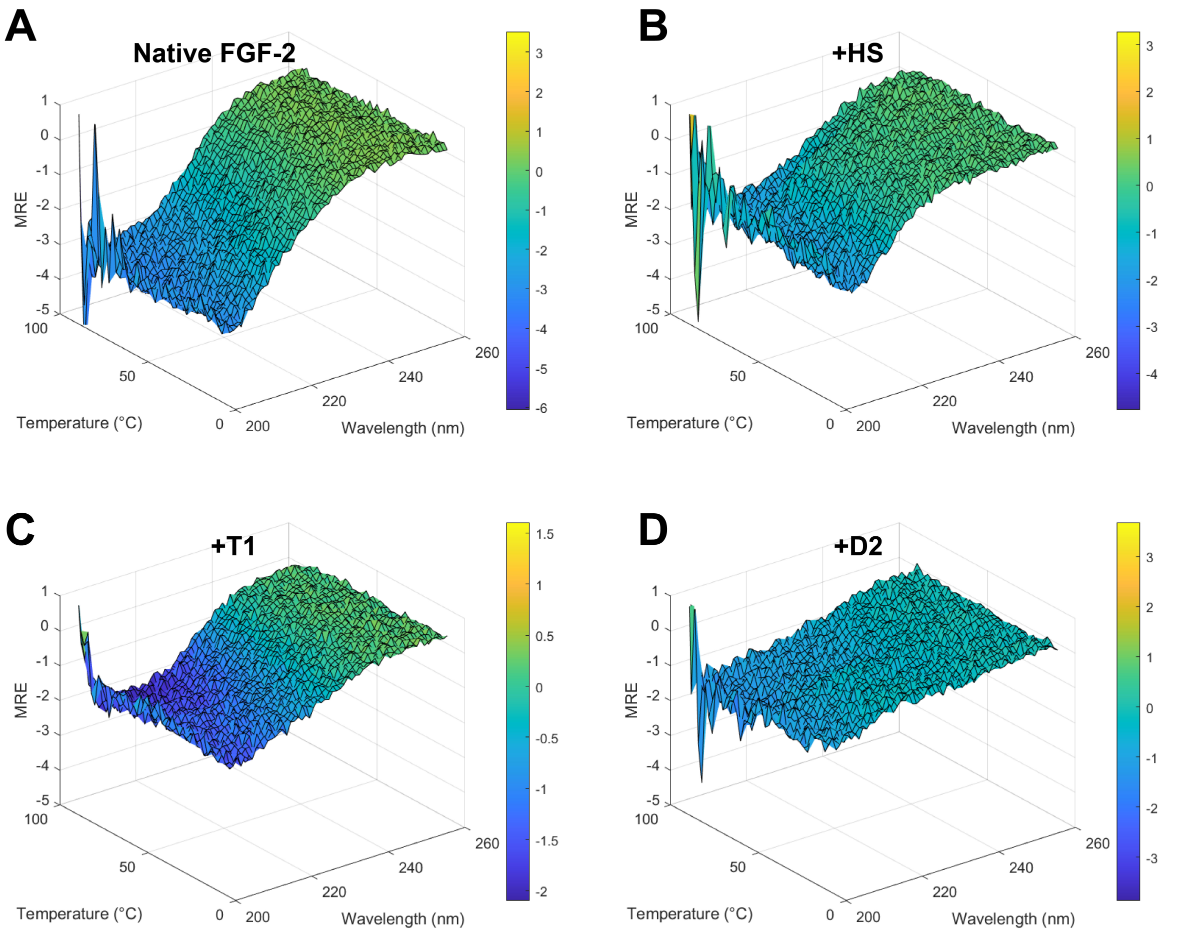

Figure S3.1. Far-UV CD spectra of FGF-2 samples measured at different temperatures **(A)** Native FGF-2, **(B)** FGF-2 + HS, **(C)** FGF-2 + T1, **(D)** FGF-2 + D2.

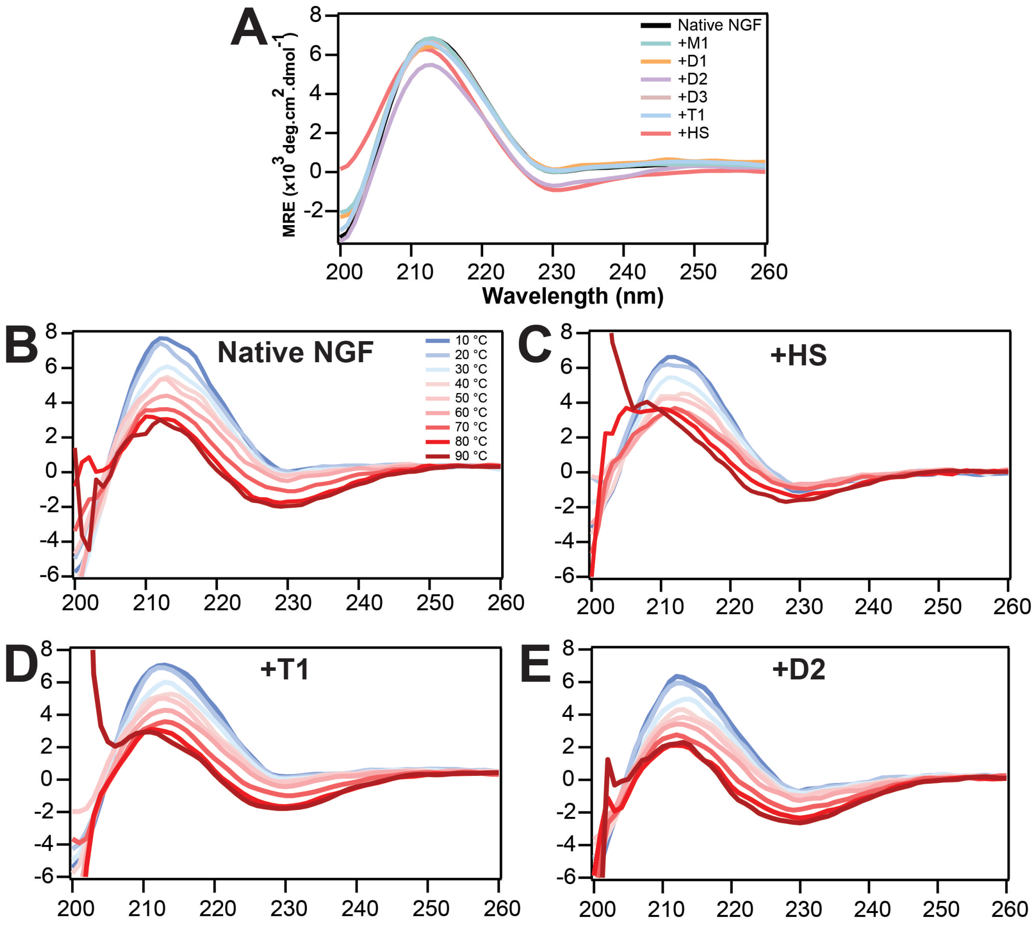

Figure S3.2. Far-UV CD spectra of NGF samples: **(A)** Native NGF and NGF incubated with sugars at R.T. **(B)** Native NGF at different temperatures. **(C)** NGF + HS at different temperatures. **(D)** NGF + T1 at different temperatures. **(E)** NGF + D2 at different temperatures.

4. Biolayer Interferometry (BLI) Experiments

The measurements were recorded on the Gator BLI system (Palo Alto, Canada) at a constant temperature of 25 °C. Before each measurement, the Gator amine-reactive probes were hydrated by pre-wetting them in 1X sterile PBS. In all assays, 1X sterile PBS, pH 7.4, was employed as the running and sample buffer. Borate buffer (100 mM boric acid + 1.0 M NaCl, pH 8.3) was used as an immobilization buffer to ensure the effective immobilization of small negatively charged molecules. The BLI response was recorded as the net BLI shift (nm), calculated by subtracting the signal of reference probes from the response signal obtained with experimental probes.

4.1. Binding Assays

The binding capacity of native HS and HS glycomimetics (**M1**, **D1**, **D2**, **D3**, and **T1**) to various proteins, including key growth factors (FGF-1, FGF-2, NGF) and antithrombin III (AT-III), was evaluated individually. Each sugar (500 μM in borate buffer) was immobilized on the probes

using standard amine coupling chemistry. The probe surface was activated with a freshly prepared mixture of 200 mM EDC and 50 mM NHS dissolved in 100 mM MES buffer containing 500 mM NaCl (pH 4.7) at a 1:1 (v/v) ratio. Residual NHS-activated esters were quenched with 1.0 M ethanolamine (pH 8.5), and the probes were washed with PBS until a stable baseline was achieved. For binding analysis, the sugar-immobilized probes were dipped into solutions containing different concentrations of proteins for 150 seconds (association phase), followed by immersion in fresh PBS for 600 seconds (dissociation phase and baseline stabilization for subsequent cycles). Control probes to assess background signals were prepared identically but without immobilized sugars (borate buffer only), and also sugar-immobilized probes dipped into blank PBS without proteins. Binding responses were recorded in real-time and analyzed to calculate the equilibrium dissociation constants (K_D) using the steady-state affinity curve fitting.

Sugar	K_D
**M1**	Not applicable
**D1**	Not applicable
**D2**	Not applicable
**D3**	Not applicable
**T1**	55 μM
**Native HS**	0.4 μM

Table S4.1. Equilibrium dissociation constants (K_D) of native HS and HS glycomimetics from the interactions with AT-III.

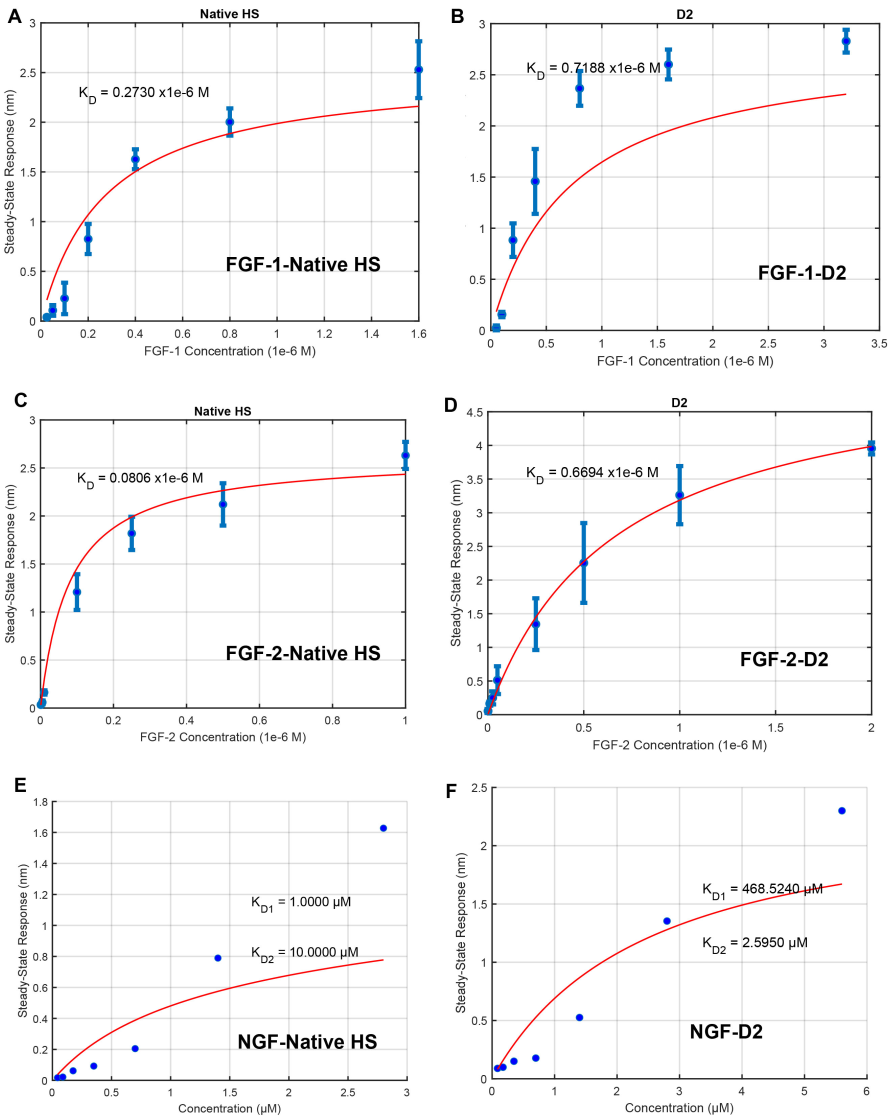

Figure S4.1. Steady state affinity curves to calculate K_D for the binding assay of **(A)** FGF-1-Native HS, **(B)** FGF-1-D2, **(C)** FGF-2-Native HS, **(D)** FGF-2-D2. Data points represent the mean of three independent experiments. Two-site binding model fitting **(E)** NGF-2-Native HS, **(F)** NGF-D2.

4.2. Competition Assays

To evaluate the ability of synthetic HS mimetics to compete with native HS for FGF-2 binding, a solution competition assay was performed to determine apparent IC_{50} values, representing the concentration of the mimetic required to reduce FGF-2 binding by 50% at defined association time (t=240s). Native HS (500 μM) was immobilized on the probes, following the procedure described in the binding assays, until a stable loading signal was achieved. FGF-2, at a fixed concentration of 50 nM, was pre-mixed with various concentrations of the inhibitors **M1**, **D1**, **D2**, **D3**, **T1**, and **H1** for 1 hour. The HS-immobilized probes were then dipped into solutions containing FGF-2 alone (positive control, representing the highest binding

response) or FGF-2 pre-incubated with inhibitors. Negative controls, consisting of mimetics dissolved in PBS without FGF-2, were included to measure non-specific signals. Binding responses were normalized to the positive control, and apparent IC_{50} values were calculated by fitting the inhibition data to a dose-response curve using Igor Pro 6.36. The association and dissociation phases were conducted under similar conditions as described for the binding assays. Experiments were performed in triplicate.

5. Determination of the Anticoagulant Activity of Sugars

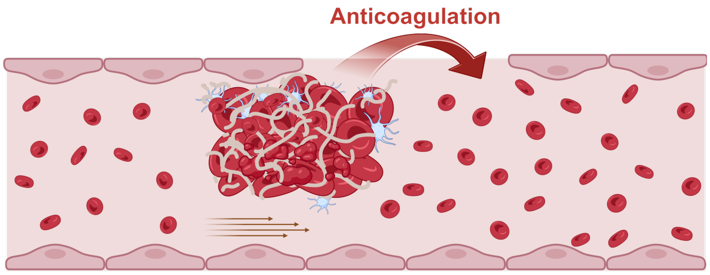

Scheme S11. Schematic representation of anticoagulation in a blood vessel.

5.1. AT-III-Mediated Inhibition of FXa and FIIa

Chromogenic assays were performed to evaluate the inhibition of thrombin, factor IIa (FIIa), and factor Xa (FXa) by unfractionated heparin (UFH), fondaparinux (clinical anticoagulant as a positive control), or HS mimetics. The assay involved a three-step reaction: (i) formation of the AT-III-heparin complex by incubating the test compound (UFH or HS mimetic) with AT-III, (ii) addition of excess FIIa or FXa to the AT-III-heparin complex to form FIIa- or FXa-AT-III-heparin complexes, leaving residual unbound FIIa or FXa, and (iii) reaction of the residual FIIa or FXa with a chromogenic substrate, resulting in the release of a peptide and p-nitroaniline. The release of p-nitroaniline was monitored by measuring colour intensity at 405 nm, which is directly proportional to the activity of residual FIIa or FXa.

i. Heparin + AT-III → [AT-III-Heparin]

ii. [AT-III-Heparin] + [FIIa or FXa (excess)] → [FIIa- or FXa-AT-III-Heparin] + [Residual FIIa or FXa]

iii. [Residual FIIa or FXa] + Chromogenic Substrate → Peptide + *p*-nitroaniline (colour intensity measured at 405 nm)

Varying concentrations of compounds **M1**, **D1**, **D2**, **D3**, **T1**, Fondaparinux, UFH, and heparan sulfate were tested for their anti-Factor IIa activity using the BIOPHEN™ ANTI-IIa kit (2-stage heparin assay, Cat. #HY-220005, Endotell AG, Allschwill, Switzerland). Following the kit instructions, for **M1**, **D1**, **D2**, **D3**, **T1,** and Fondaparinux, the reagents were prepared as

follows: human AT-III (reconstituted in Milli-Q water and diluted with Tris-NaCl-BSA, pH 7.4), purified human thrombin (Factor IIa) (reconstituted in Milli-Q water and diluted with Tris-NaCl-BSA, pH 7.4), and thrombin-specific chromogenic substrate [CS-01(38)] (reconstituted in Milli-Q water and diluted with Tris-NaCl-EDTA, pH 8.4). AT-III (0.025 IU, 40 µL) and the compounds or control at different concentrations (40 µL) were incubated in triplicate for 2 minutes at 37 °C in a 96-well plate (flat-bottom, clear). After adding Factor IIa (0.96 IU, 40 µL), mixing, and incubating at 37 °C for another two minutes, the chromogenic Factor IIa substrate was introduced (0.05 µg, 40 µL), followed by mixing and incubating at 37 °C for another two minutes. The reaction was quenched by adding citric acid solution (2 wt.%, 80 µL) exactly after 2 minutes and mixed. Absorbance at λ=405 nm was recorded against the corresponding blank sample on the BioTek Synergy H1 Multimode Microplate Reader (Agilent Technologies, Inc.). To prepare the blank sample, the test components were mixed in reverse order: citric acid, chromogenic Factor IIa substrate, Factor IIa, AT-III, and the sample. A blank absorbance was subtracted from the test absorbance. For the UFH sample, the same protocol was followed except for the dilution buffer. The reagents were prepared as follows for UFH samples: human AT-III (diluted with Tris-EDTA-NaCl-PEG, pH 8.4), purified human thrombin (Factor IIa) (diluted with Tris-EDTA-NaCl-PEG, pH 8.4), and thrombin-specific chromogenic substrate [CS-01(38)] (diluted with Milli-Q water). Similarly, for the evaluation of anti-factor Xa activity, BIOPHEN™ ANTI-Xa kit (2-stage heparin assay, Cat. #HY-221005, Endotell AG, Allschwill, Switzerland) was used. Following the kit protocol, human AT-III, purified bovine Factor Xa, and Factor Xa-specific chromogenic substrate [CS-11(65)] were reconstituted and prepared in the respective buffers as described above. AT-III (0.04 IU, 40 µL) and the compounds or control at different concentrations (40 µL) were incubated in triplicate for 2 minutes at 37 °C in a 96-well plate (flat-bottom, clear). After adding Factor Xa (0.32 µg, 40 µL), mixing, and incubating at 37 °C for another two minutes, the chromogenic Factor Xa substrate was introduced (0.032 µg, 40 µL). The reaction was quenched by adding citric acid (2 wt.%, 80 µL) after 2 minutes and mixed. Absorbance at λ=405 nm was recorded against the corresponding blank sample on the BioTek Synergy H1 Multimode Microplate Reader (Agilent Technologies, Inc.). To prepare the sample blank, the test components were mixed in reverse order: citric acid, chromogenic Factor Xa substrate, Factor Xa, AT-III, and the sample. A blank absorbance was subtracted from the test absorbance. Measured absorbance values were converted to FIIa or FXa activity (%).

5.2. Activated Partial Thromboplastin Time (aPTT) Measurements

UFH, heparan sulfate, Fondaparinux, and synthetic HS glycomimetics (**M1**, **D1**, **D2**, **D3**, **T1**) were supplemented into pooled rat citrated plasma (obtained from Sigma Cat. #P2516) at

various final concentrations, the same as the ones used in 5.1. Control (no sugar) or test compounds were added to individual aliquots of plasma. Samples were analyzed using the Pacific Hemostasis APTT-XL reagent (0.003% ellagic acid, 0.005% BSA, 0.30% phenol) obtained from Thermo Fisher Scientific, Cat. #100402 according to the manufacturer's instructions. After 5 minutes of incubation at 37 °C , coagulation was initiated with pre-warmed 0.025 M $CaCl_2$, and clotting times were recorded optically on a CoaDATA 4004 coagulometer. All reagents were reconstituted per manufacturer's instructions, and measurements were performed in triplicate on three independent runs. Results are expressed as mean ± SD.

6. Testing HS Glycomimetics on Neural Cell Lines

6.1. Neural Cell Culture and Differentiation

6.1.1. PC12 Cells

PC12 cells (Manassas, VA, USA), derived from a transplantable rat pheochromocytoma, are widely used due to their ability to differentiate in response to NGF (Greene and Rein, 1977a, 1977b). Upon differentiation, these cells extend neurite-like processes, become electrically excitable, and respond to neurotransmitters. PC12 cells were maintained in Dulbecco's Modified Eagle's Medium (DMEM; 4.5 g/L glucose, L-glutamine; Gibco), supplemented with 10% horse serum, 5% fetal bovine serum (FBS), and 1× antibiotic-antimycotic solution (100 U/mL penicillin, 100 µg/mL streptomycin, and 250 µg/mL amphotericin B), and cultured on poly-D-lysine (PDL)-coated Petri dishes in a humidified incubator with 10% CO_2 at 37 °C. On the day of the experiment, PC12 cells were processed as described by Karliner *et al.* [2] Briefly, cells were detached using trypsin and passed through a 40 µm sterile cell strainer to minimize clumping. After centrifugation, the cell pellet was resuspended in 1 mL of complete growth medium. The suspension was then passed 15 times through a sterile 26-gauge syringe needle to achieve a single-cell suspension. Cells were seeded at a density of 10000 cells/cm² in a complete medium. Four hours post-plating, the medium was replaced with a differentiation medium composed of DMEM supplemented with 5% horse serum, 2.5% FBS, and 50 ng/mL NGF. Differentiation medium was refreshed every other day via half-volume medium changes. Cells were fixed after three days of differentiation.

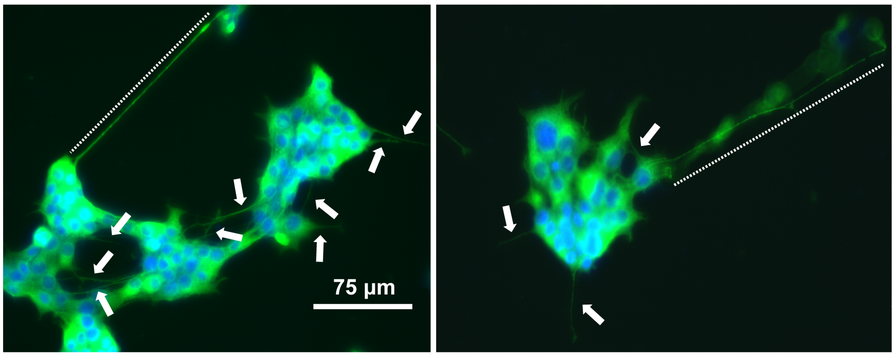

Figure S6.1. Immobilized glycomimetic D2 enhances NGF-mediated neuronal maturation in PC12 cell models. Representative immunofluorescence images of clumped PC12 cells stained with anti-β-tubulin III (green) and DAPI (blue) to visualize neurites and nuclei, respectively. Longest neurites are marked with white dashed lines; white arrows indicate additional neuritic projections.

6.1.2. SH-SY5Y Cells

SH-SY5Y cells (ECACC; Sigma-Aldrich, St. Louis, MO, USA), a subclone of the SK-N-SH neuroblastoma line, were cultured in DMEM/F12 medium supplemented with 10% FBS, 2 mM L-glutamine, 50 U/mL penicillin, and 50 µg/mL streptomycin (all from Gibco, Paisley, UK). Cells were maintained in a humidified incubator at 37 °C with 5% CO_2. The culture medium was changed twice weekly, and cells were passaged at approximately 80% confluence. For differentiation, cells were seeded at a density of 10000 cells/cm² in a complete growth medium. After 4 hours, the medium was replaced with the differentiation medium consisting of DMEM/F12 supplemented with 2.5% FBS and 50 ng/mL NGF. Cells were fixed after three days of treatment.

6.2. Treatment of Cells on Glycomimetic Coated Surfaces

Glycomimetic compounds were applied to the surface of the culture wells the night before cell seeding and incubated at 37 °C overnight. Control wells received an equivalent volume of sterile 1× PBS to match the volume used for that of compounds.

6.3. Immunofluorescence Labeling

PC12 and SH-SY5Y cells were fixed with 4% paraformaldehyde (PFA) for 10 minutes at R.T. to preserve cellular structures. Following fixation, the cells were permeabilized using 0.1% Triton X-100 for 5 minutes. After two washes with 1X phosphate-buffered saline (PBS), non-specific binding sites were blocked by incubating the cells with 5% of Bovine Serum Albumin (BSA) in 1X PBS for 30 minutes at RT. To visualize neurite formation, cells were incubated for

1 hour at R.T. in the dark with an Alexa Fluor 488-conjugated anti-βIII tubulin antibody (Invitrogen), diluted in blocking buffer at a dilution of 1:300. After primary antibody incubation, cells were washed three times with PBS to remove unbound antibody. Nuclei were counterstained with DAPI (1 µg/mL in PBS) for 5 minutes, followed by two additional PBS washes. Finally, fluorescence imaging was performed using an EVOS M5000 Imaging System microscope equipped with a FITC/GFP filter set, with a 40X (0.75 NA) air objective.

6.4. Image Analysis

Neurite outgrowth was assessed by manually tracing β-tubulin III–positive projections using NeuronJ plugin of ImageJ. Tracing was performed on 12-15 randomly selected images per condition. The total neurite length per image was calculated by summing all traced neurites. Neurite lengths were recorded in micrometers. Individual neurite lengths were extracted and plotted to assess distribution. For per-image summary, the total neurite length per image was used as a biological replicate (n = 12 per group).

7. Testing HS Glycomimetics on Dissociated Hippocampal Cultures

7.1. Preparation of Dissociated Hippocampal Cultures

Postnatal (P2-P3) Wistar rat pups were sacrificed to obtain dissociated primary hippocampal cell cultures, on which the experiments were performed. All procedures were conducted in accordance with Italian law (decree 26/14) and EU guidelines (2007/526/CE and 2010/63/UE). The study was authorized by the Italian Ministry of Health (authorization number: 22DAB.N.1Z8) and approved by local veterinary authorities. After hippocampus isolation, cells were enzymatically and mechanically dissociated, resuspended in Neurobasal culture medium supplemented with B27, plated onto poly-L-ornithine–coated glass coverslips (0.01% of concentration) at densities of 80000 cells/mL and 130000 cells/mL for immunofluorescence and electrophysiological experiments, respectively; then maintained in a humified incubator (37 °C, 5% CO_2), as reported in Cellot *et al.* with minor modifications.[3]

7.2. Treatment of Dissociated Hippocampal Cultures

All treatments were performed using the concentrations reported in Table S7.1. All treatment solutions were constantly stirred at 37 °C for 2 hours before being directly added to the culture medium. Control samples were added with the same volume of sterile PBS 1X of that used for the other treatments.

Group	Final Concentration
**Control**	Sterile PBS
**FGF-2**	FGF-2 at 40 ng/mL
**D2**	**D2** at 1 µM
**D2+FGF-2**	FGF-2 at 40 ng/mL + **D2** at 1 µM

Table S7.1. Experimental groups and the treatment conditions.

For immunofluorescence labelling experiments, cultures were treated at 0 days of differentiation *in vitro*, 2 hours after cell seeding. For electrophysiology, cultures were treated at 7 days of differentiation *in vitro,* a developmental stage at which neurons are reconnecting and forming active synapses. After treatment, cells were gently shaken to ensure homogeneous distribution of substances and incubated for 24 hours in a humidified incubator (37 °C, 5% CO_2) before performing experiments.

7.3. Immunofluorescence Labeling

Hippocampal cultures were washed in PBS solution and then fixed with 4% paraformaldehyde (PFA) for 20 minutes. Cells were then processed for immunofluorescence staining. Permeabilization was performed using 1% Triton X-100 for 30 minutes, followed by blocking with 5% FBS in PBS for 30 minutes at room temperature. Samples were incubated with the primary antibody rabbit polyclonal anti-β-tubulin III (Sigma, 1:250 dilution) for 60 minutes at room temperature. After PBS washes, cells were incubated for 60 minutes with the secondary antibody Alexa Fluor 594 goat anti-rabbit (Invitrogen, 1:500 dilution). Nuclear counterstaining was performed using DAPI (Invitrogen, 1:500 dilution). Samples were mounted in VECTASHIELD (Vector Laboratories) and imaged using a confocal microscope (Nikon C2-si Laser Scanning Confocal Microscope) with a 40X (0.75 NA) air objective. Fluorescence images were analysed by using Fiji software to manually measure the number of neurites extending from the cell body and the length of the longest neurite of each neuron.

7.4. Electrophysiological recordings and analysis

Electrophysiological activity was recorded from single hippocampal neurons visually identified with an upright Nikon microscope equipped with differential interference contrast optics and an infrared video camera, by using a patch clamp amplifier (Multiclamp 700A, Axon Instruments, Whipple Rd, Union City, CA, USA). Recordings were performed at room temperature using an extracellular physiological solution composed (in mM) as follows: 150 NaCl, 4 KCl, 1 $MgCl_2$, 2 $CaCl_2$, 10 HEPES, 10 glucose, pH 7.4. The whole-cell patch clamp technique was used in voltage-clamp mode. Patch electrodes were pulled from borosilicate glass capillaries and had a resistance of 4-7 MΩ when filled with an intracellular solution containing (in mM) 120 K gluconate, 20 KCl, 10 HEPES, 10 EGTA, 2 $MgCl_2$, and 2 Na_2ATP

(pH 7.3, osmolarity adjusted to 300 mOsm). Data were transferred to a computer hard disk after digitization with an A/D converter (Digidata 1322, Molecular Devices, San Jose, CA, USA). Data acquisition (digitized at 20 kHz and filtered at 2 kHz) was performed with pClamp 10.7 software (Molecular Devices, USA). Cell capacitance (93 ± 8 pF in FGF-2, n=18 cells, and 100 ± 8 pF in **D2**+FGF-2, n=21 cells), and input resistance (298 ± 57 MΩ in FGF-2 and 225 ± 30 MΩ in **D2**+FGF-2) were recorded in voltage clamp mode and measured online with the membrane test feature of pClamp software, while resting membrane potential was measured in current clamp mode at I=0 (-51 ± 2 mV in FGF-2, and -55 ± 1 mV in **D2**+FGF-2). No statistically significant differences were observed in these parameters between the two treatments. Spontaneous synaptic activity was recorded in voltage clamp mode from cells kept at a holding potential of −58 mV. Values of membrane potential were corrected for a liquid junction potential of ~16 mV (calculated with Clampex software, version 10; Molecular Devices, USA). The stability of the patch was checked by repetitively monitoring the input and series resistance during the experiments. Cells exhibiting 15% changes were excluded from the analysis. The series resistance, <20 MΩ, was not compensated. For each trace, the amplitude and frequency of sPSCs were evaluated offline with Clampfit event detection software (version 10; Molecular Devices, USA). This exploits a detection algorithm based on sliding templates to collect sPSCs on the basis of their kinetic properties.

7.5. Statistical Analysis

The Gaussian distribution of data was assessed through the Shapiro-Wilk test. Since the data was not distributed as in a Gaussian distribution, non-parametric analysis was used. In detail, for immunostaining data, the Kruskal-Wallis test was performed, followed by a two-stage linear step-up procedure of Benjamini, Krieger, and Yekutieli. For electrophysiology, the Mann-Whitney test was used. P values were considered statistically significant when less than 0.05. Data are reported as mean with the standard error of the mean (SEM). n is the number of analysed cells.

8. References

(1) Zhu, S.; Li, J.; Loka, R. S.; Song, Z.; Vlodavsky, I.; Zhang, K.; Nguyen, H. M. Modulating Heparanase Activity: Tuning Sulfation Pattern and Glycosidic Linkage of Oligosaccharides. *J Med Chem* **2020**, *63* (8), 4227–4255. https://doi.org/10.1021/acs.jmedchem.0c00156.

(2) Karliner, J.; Merry, D. E. Differentiating PC12 Cells to Evaluate Neurite Densities through Live-Cell Imaging. *STAR Protoc* **2023**, *4* (1), 101993. https://doi.org/10.1016/j.xpro.2022.101993.

(3) Cellot, G.; Toma, F. M.; Kasap Varley, Z.; Laishram, J.; Villari, A.; Quintana, M.; Cipollone, S.; Prato, M.; Ballerini, L. Carbon Nanotube Scaffolds Tune Synaptic Strength in Cultured Neural Circuits: Novel Frontiers in Nanomaterial–Tissue Interactions. *The Journal of Neuroscience* **2011**, *31* (36), 12945–12953. https://doi.org/10.1523/JNEUROSCI.1332-11.2011.

9. Spectral Data

^{1}H NMR of **2** at 400 MHz, D_2O

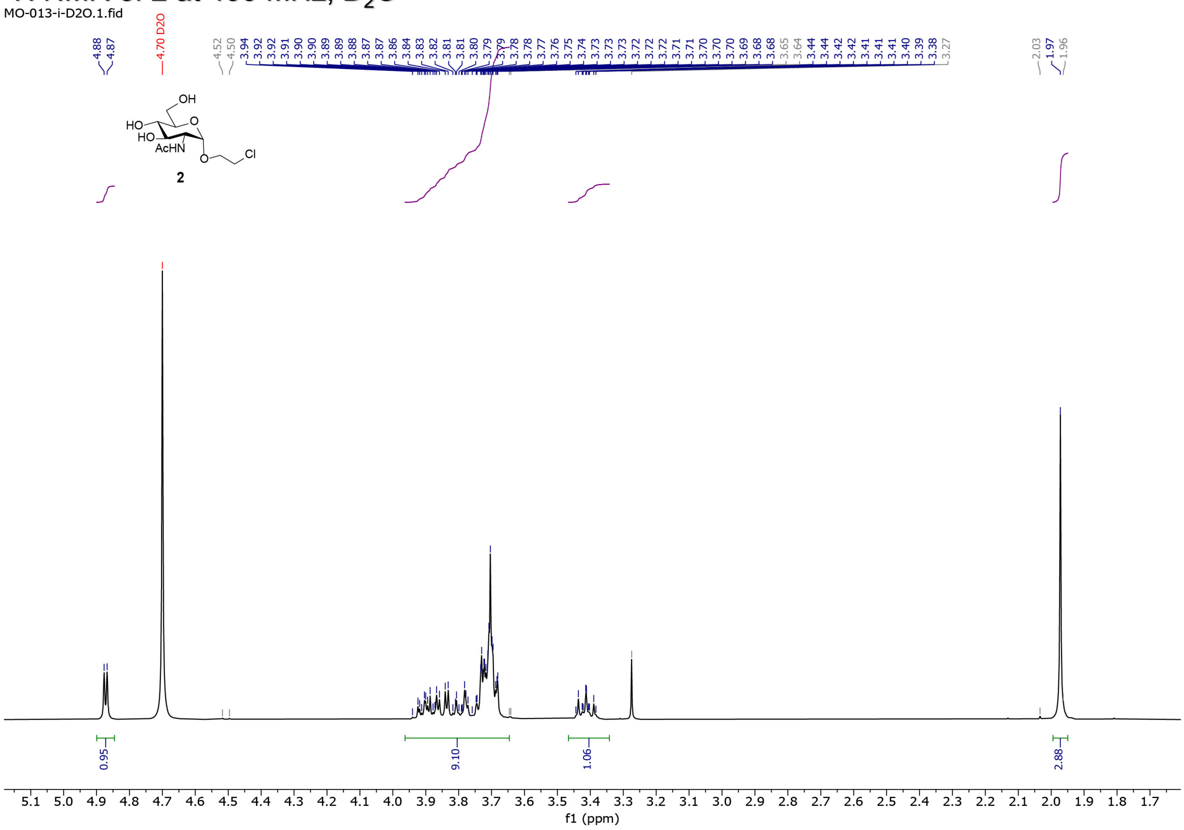

^{13}C NMR of **2** at 101 MHz, D_2O

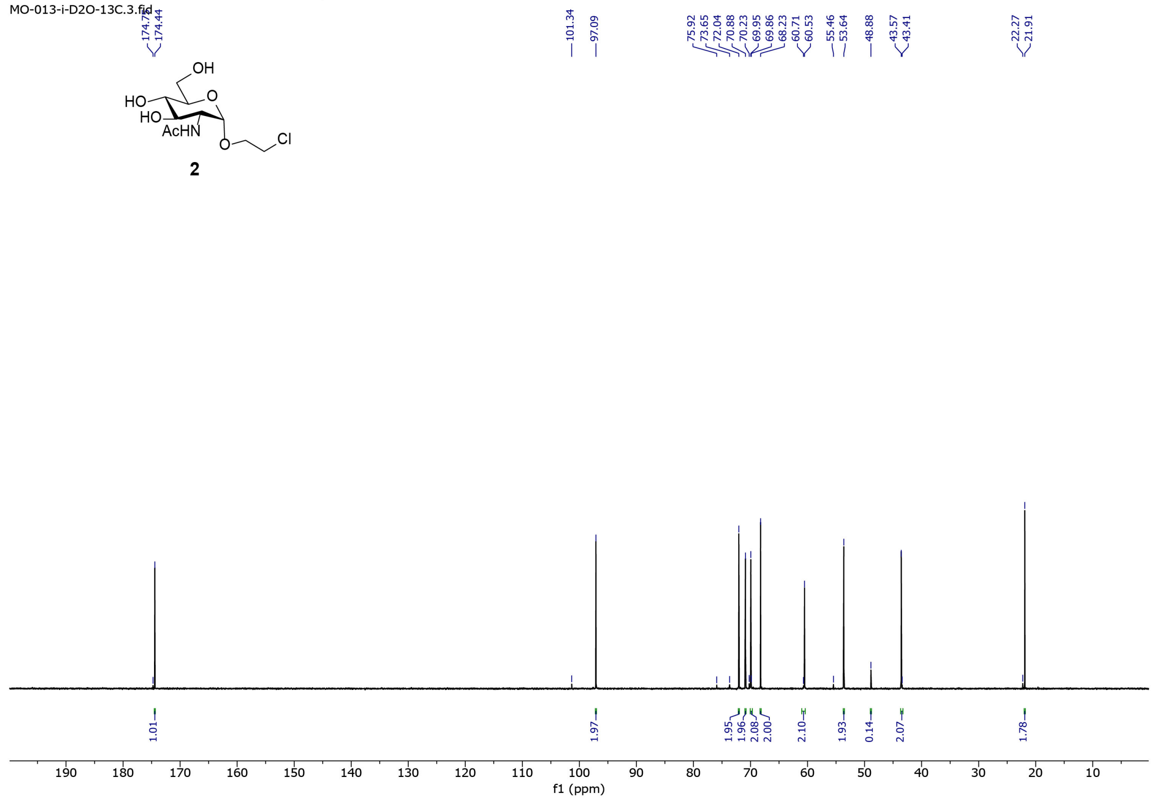

Mass Analysis of **2**

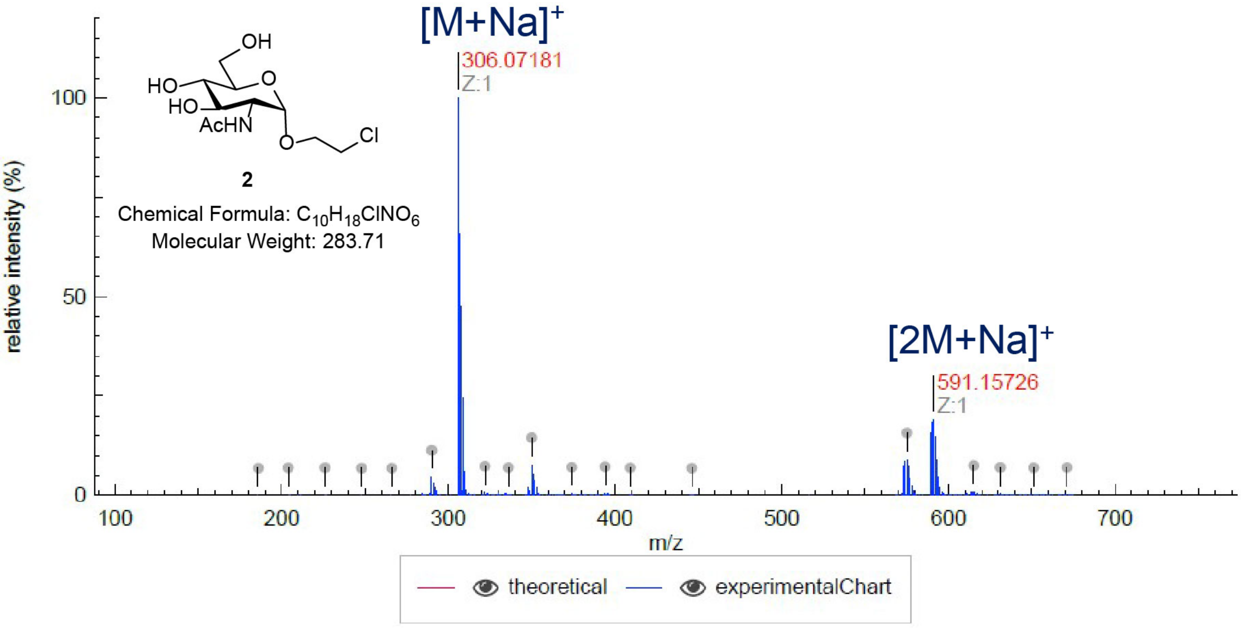

¹H NMR of **3** at 400 MHz, MeOD-d4

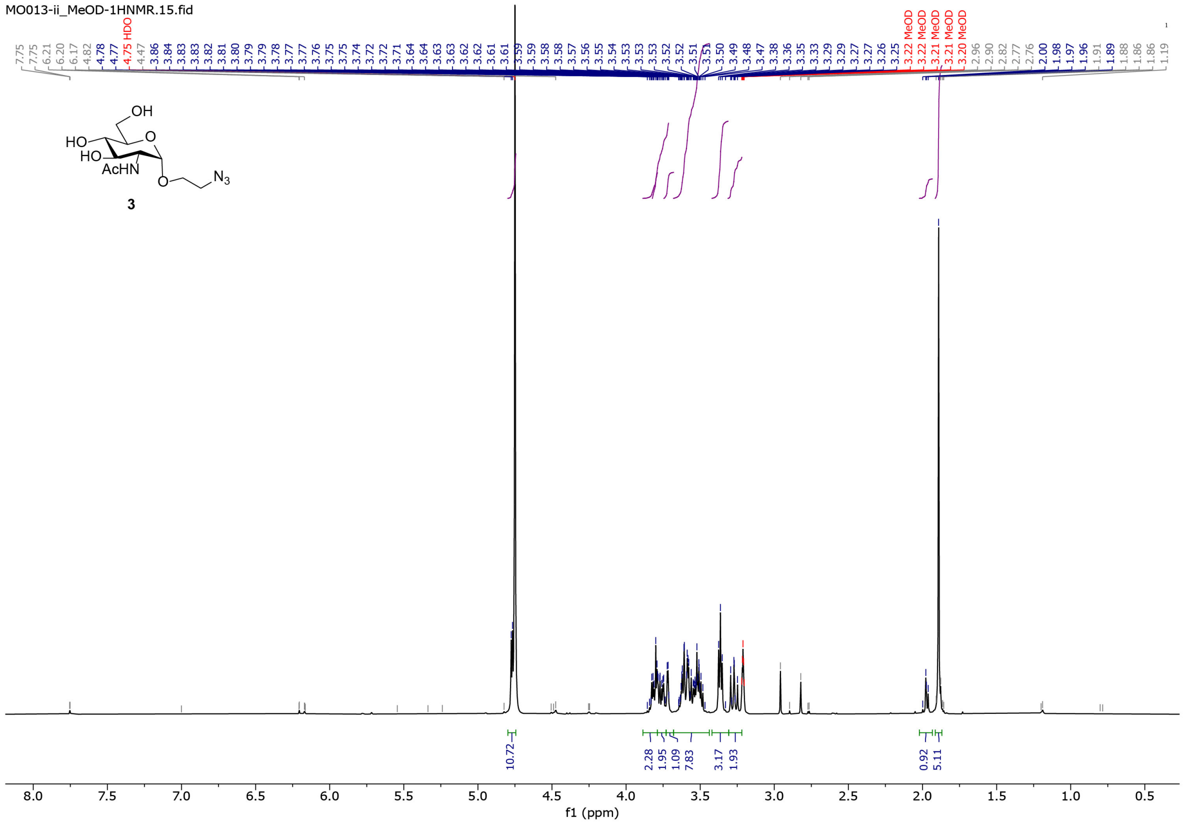

^{13}C NMR of **3** at 101 MHz, MeOD-d4

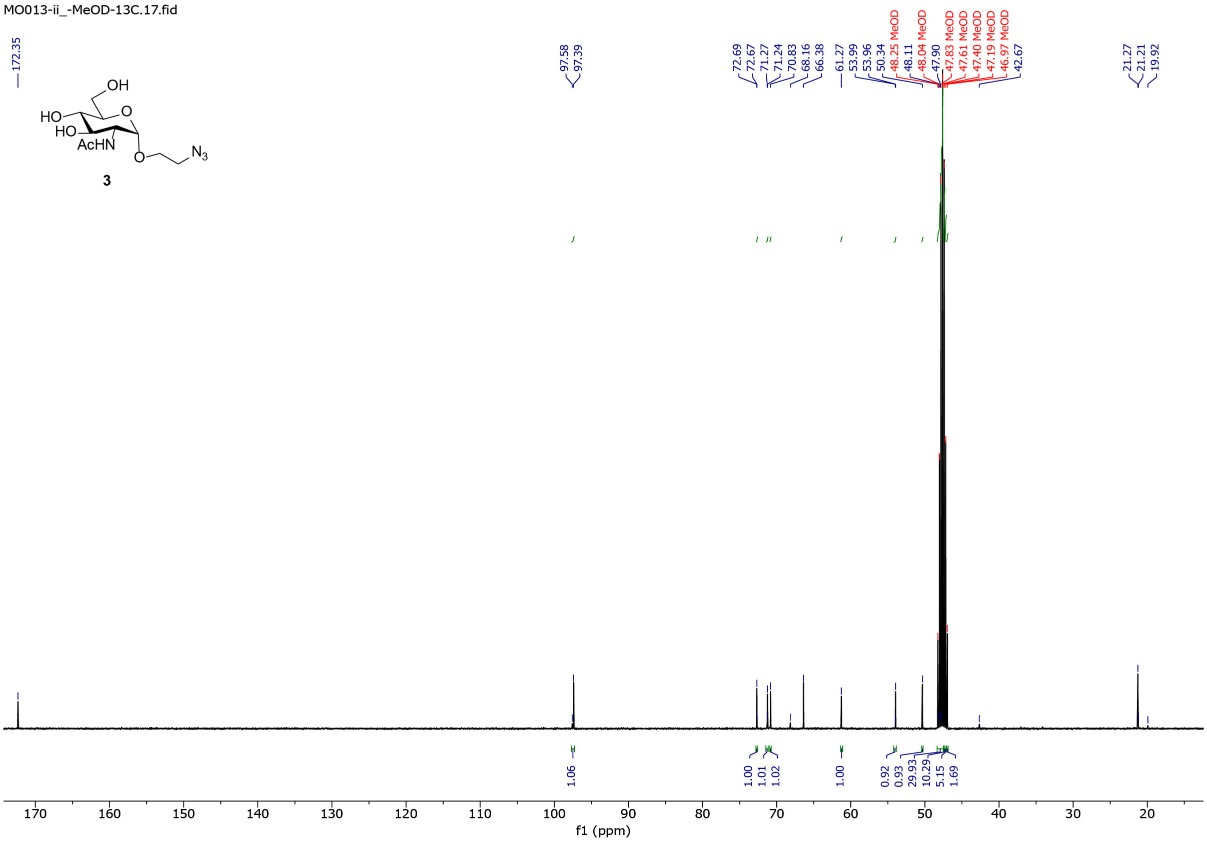

Mass Analysis of **3**

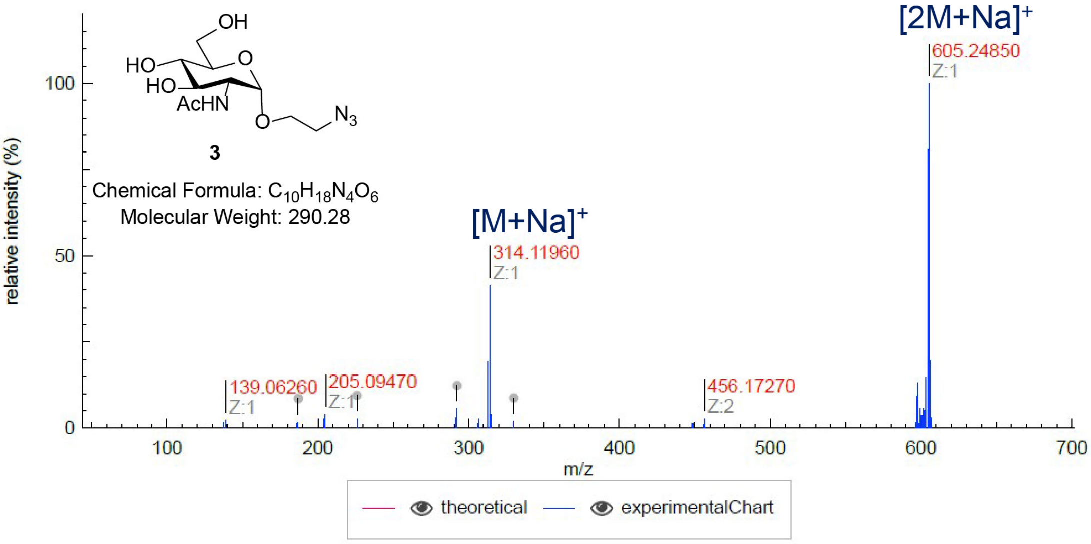

^{1}H NMR of **4** at 400 MHz, $CDCl_3$

MO-014-1HNMR-CDCl3.1.fid

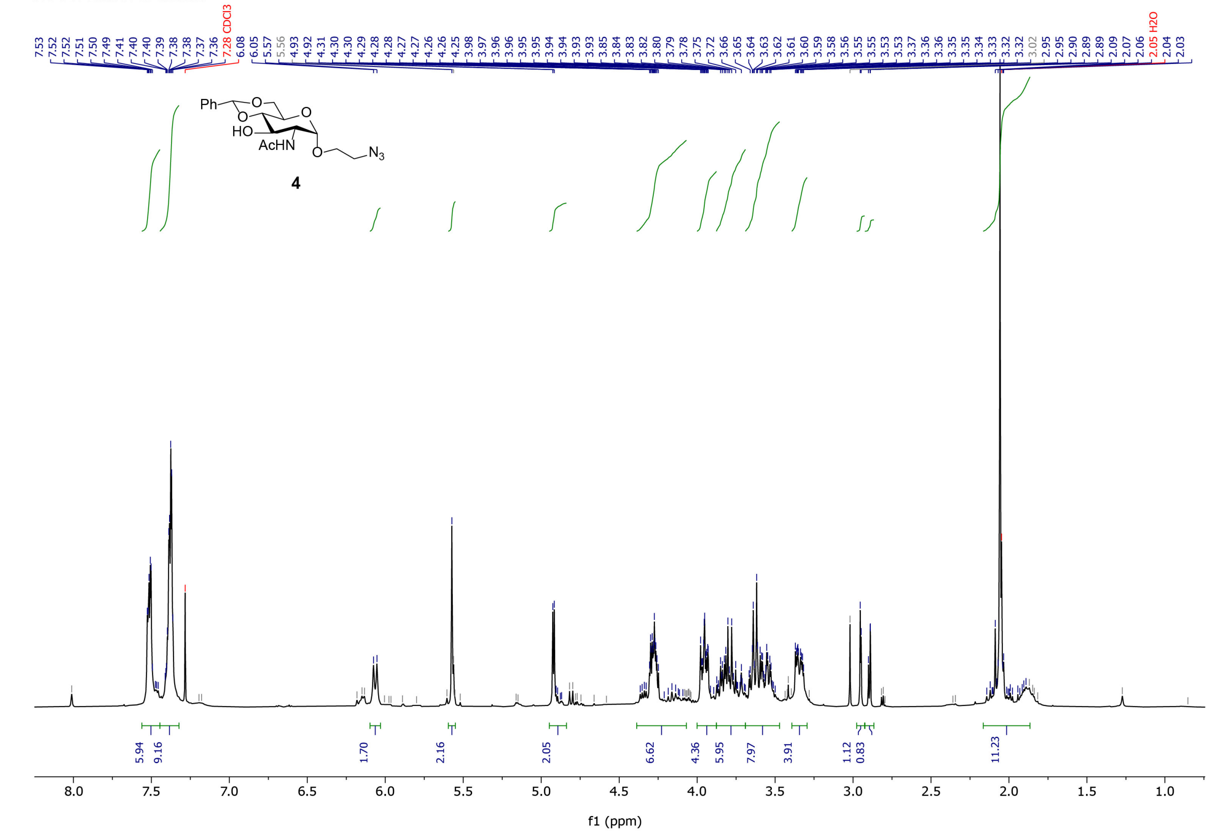

^{13}C NMR of **4** at 101 MHz, $CDCl_3$

MO-014-13C-CDCl3.7.fid

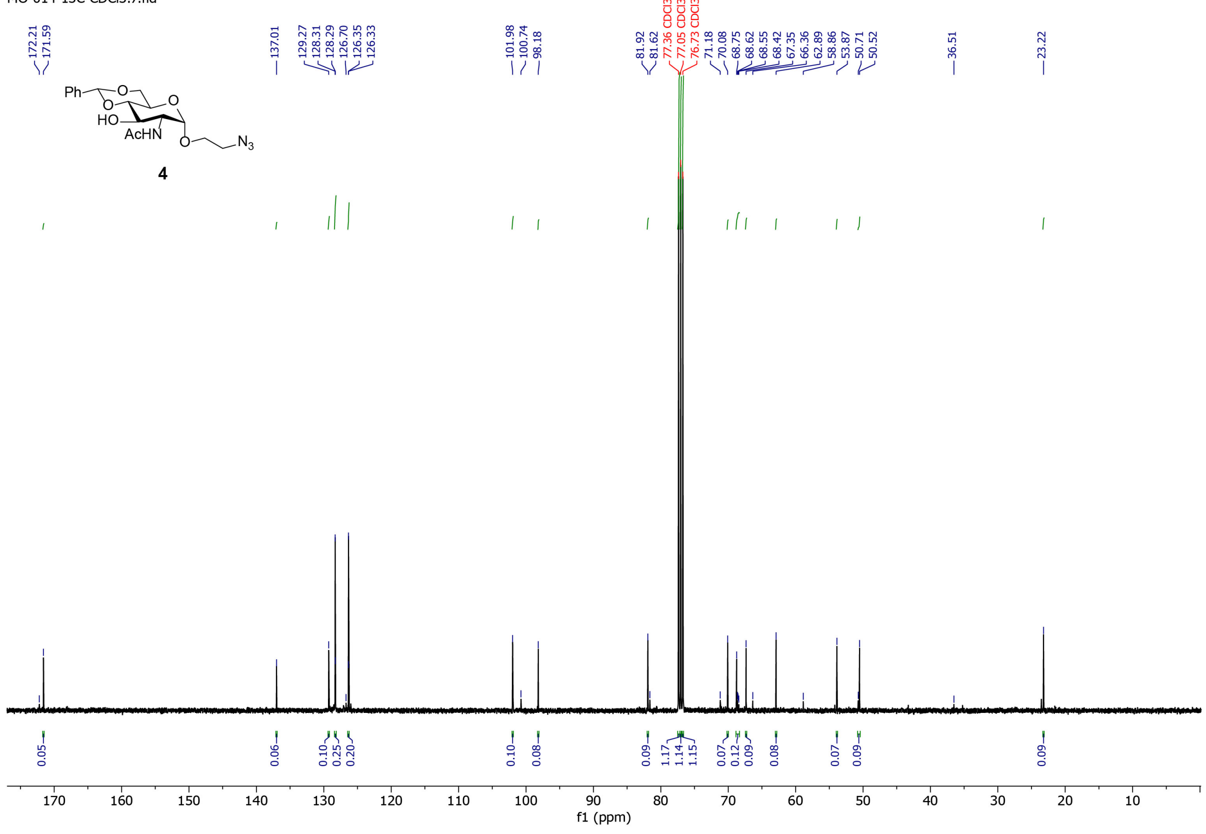

Mass Analysis of **4**

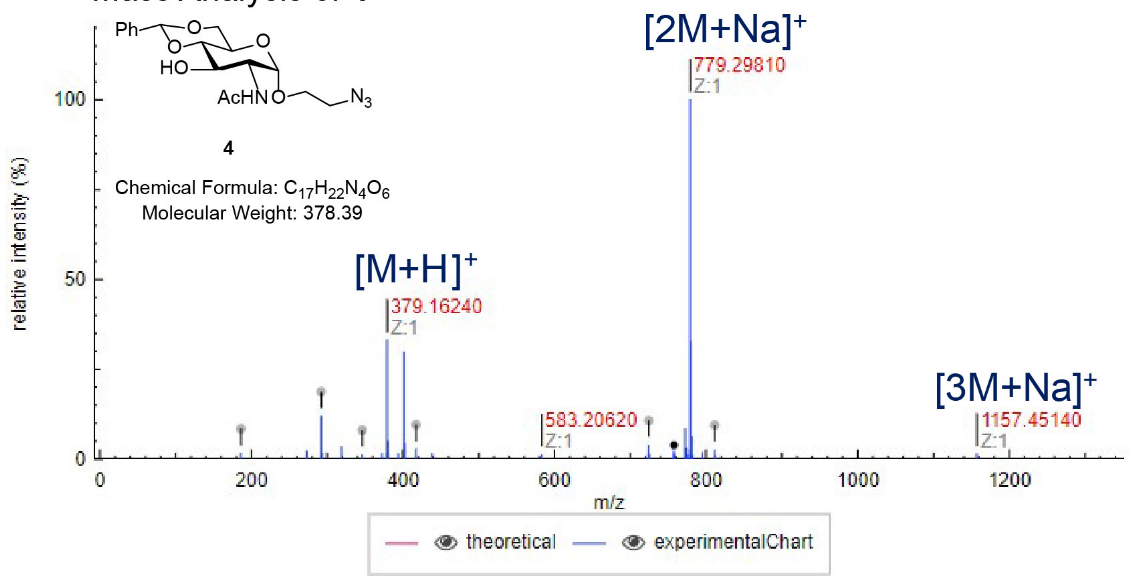

^{1}H NMR of **5** at 400 MHz, $CDCl_3$

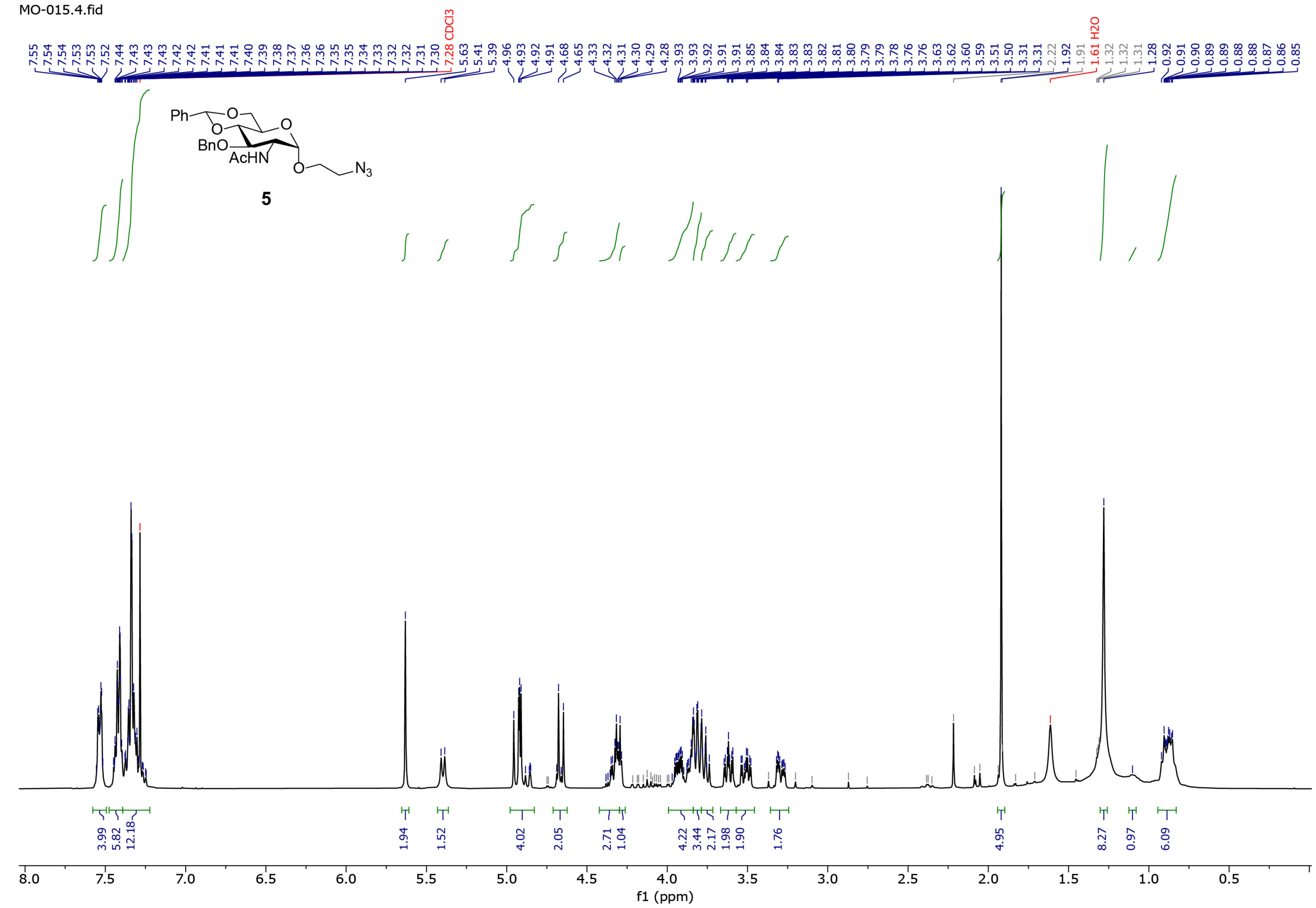

^{13}C NMR of **5** at 101 MHz, $CDCl_3$

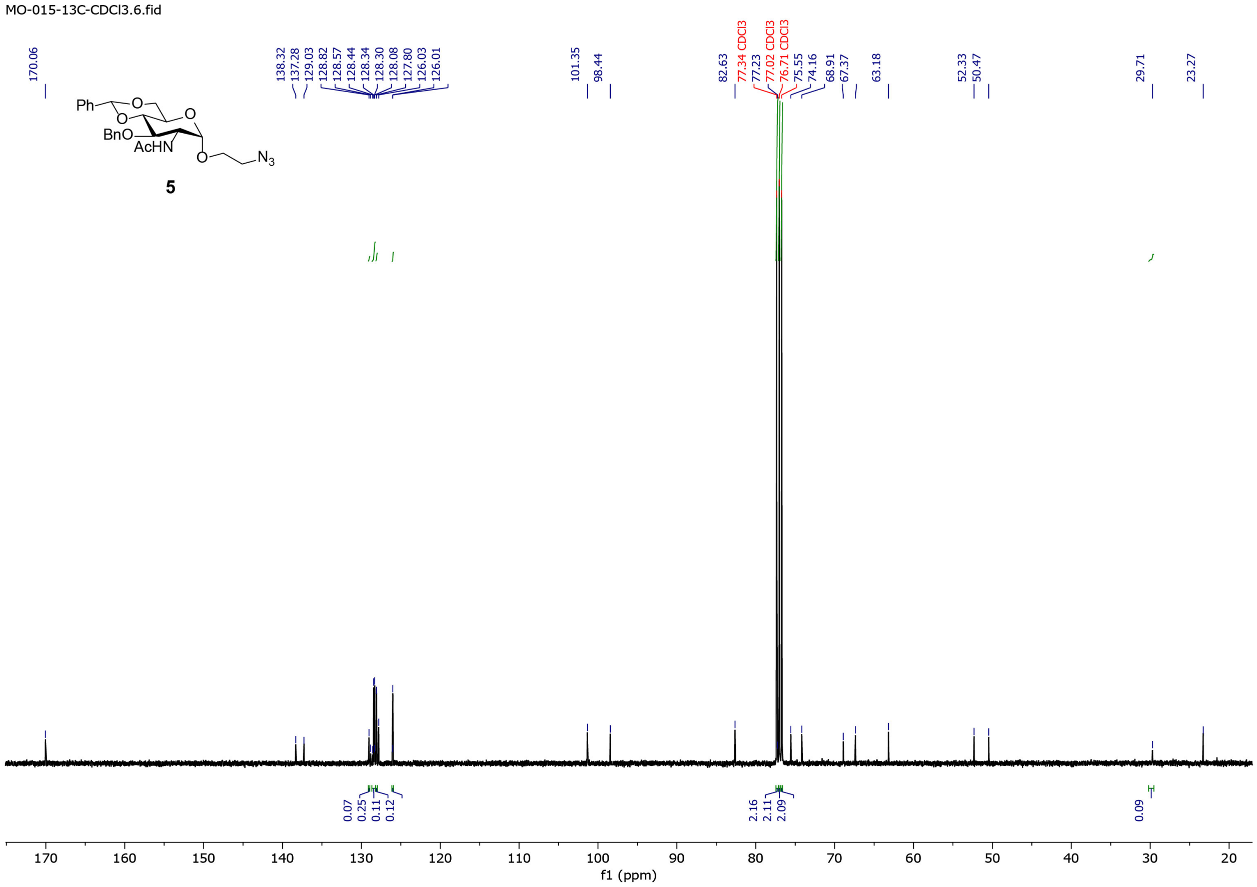

Mass Analysis of **5**

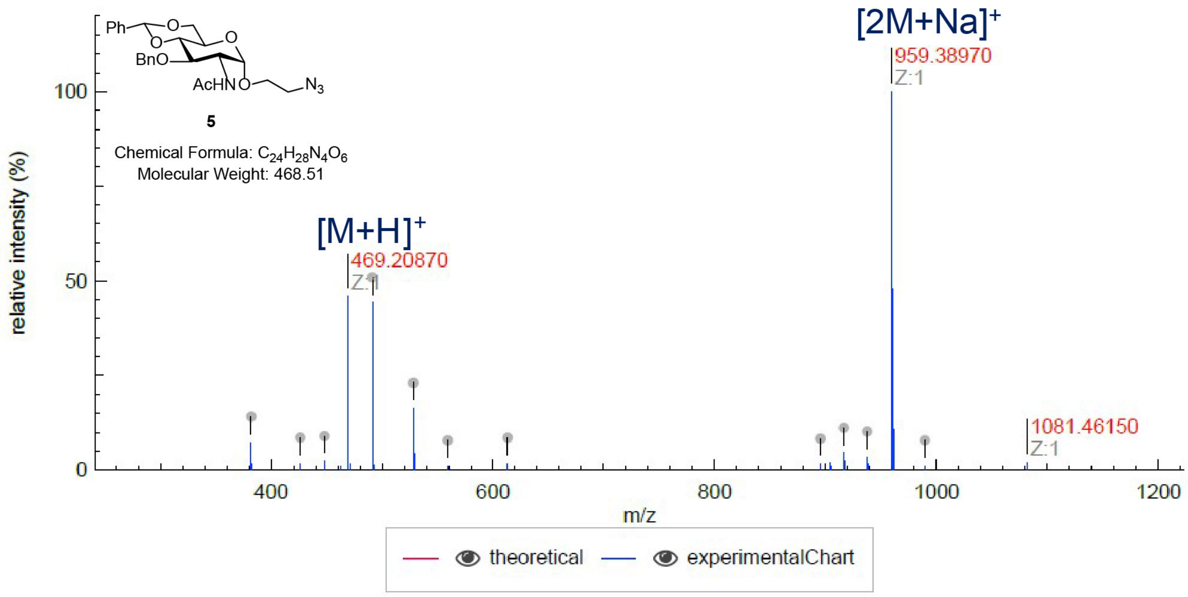

¹H NMR of **6** at 400 MHz, MeOD-d4

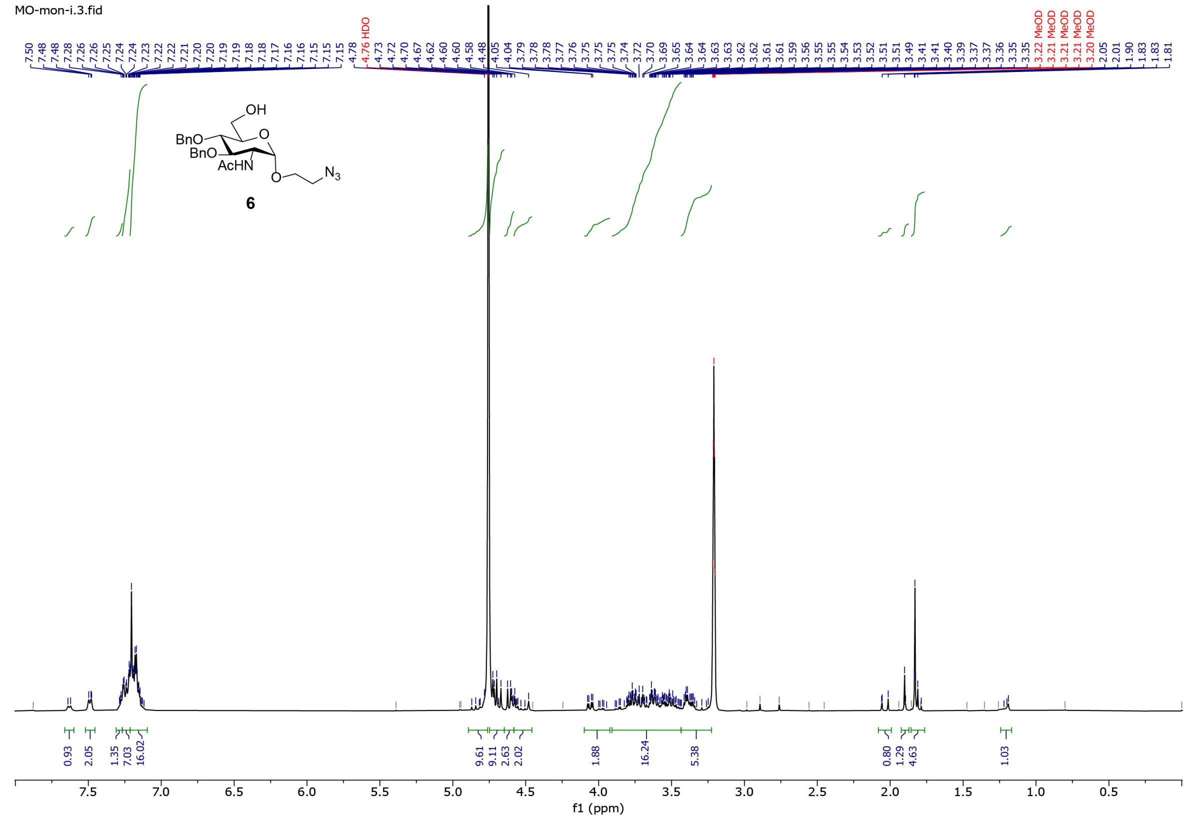

¹³C NMR of **6** at 101 MHz, MeOD-d4

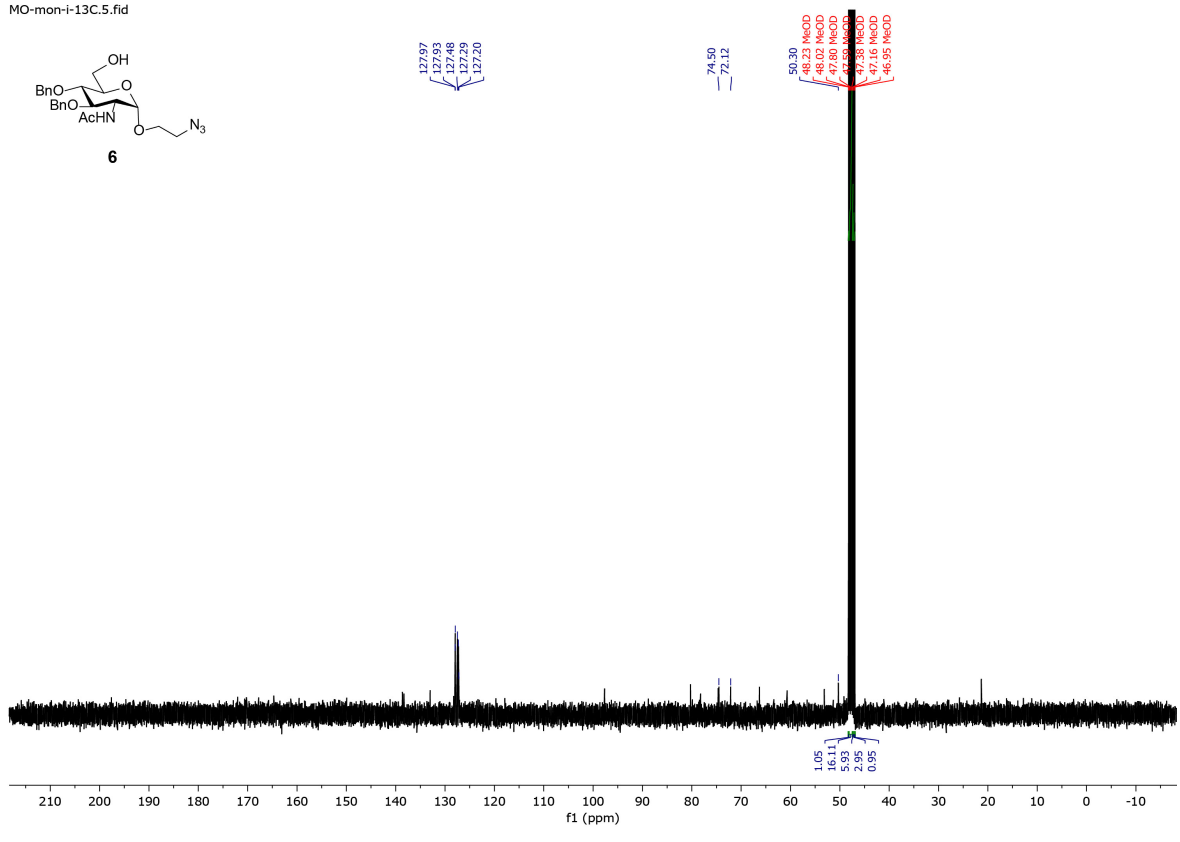

Mass Analysis of **6**

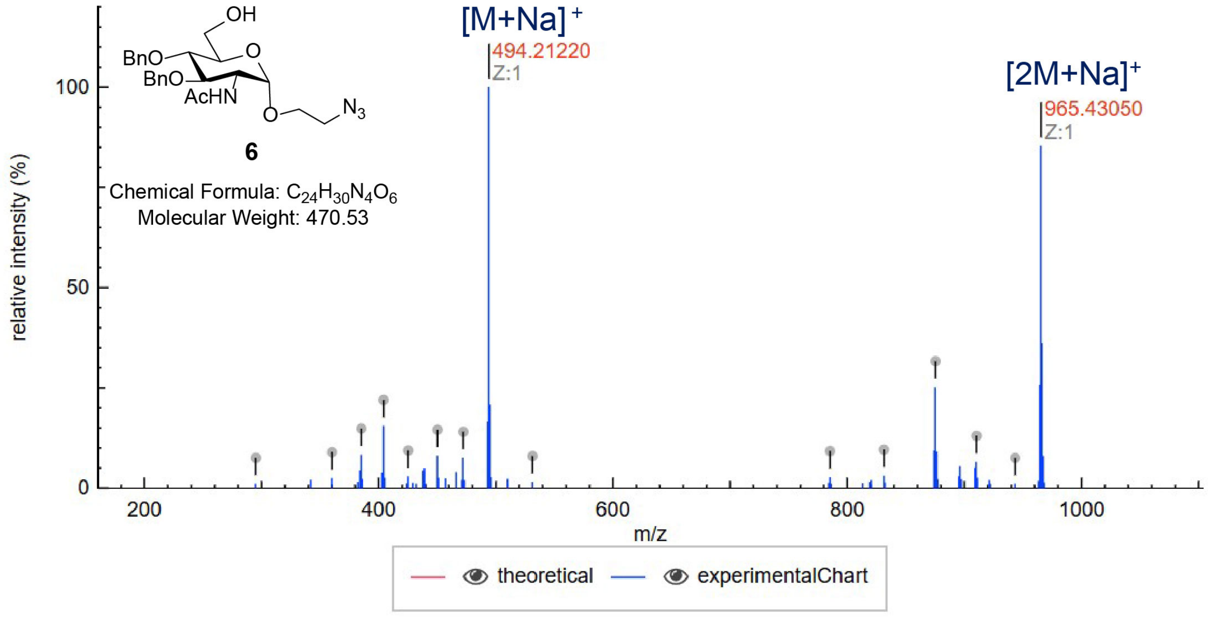

¹H NMR of **7** at 400 MHz, MeOD-d4

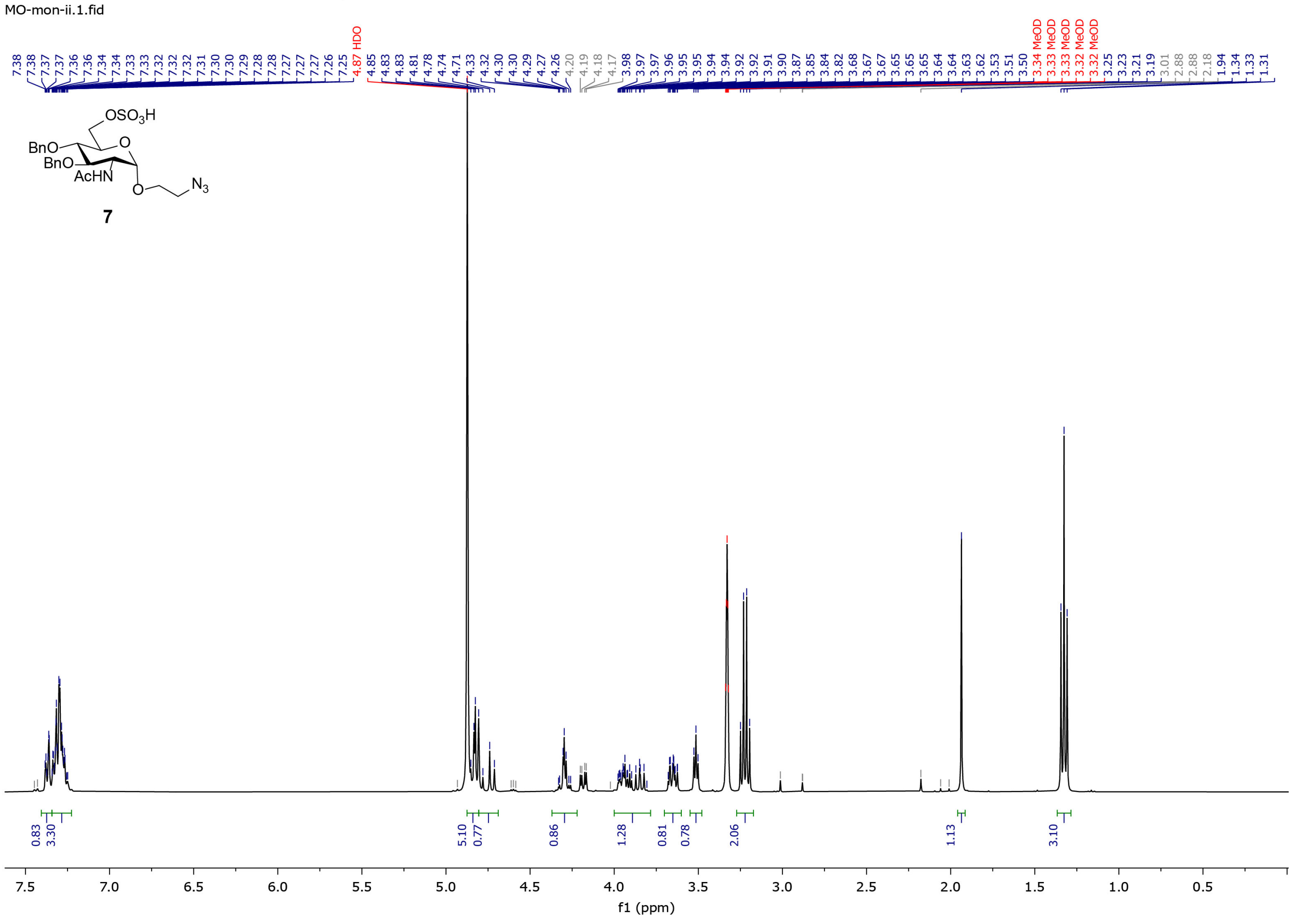

¹³C NMR of **7** at 101 MHz, MeOD-d4

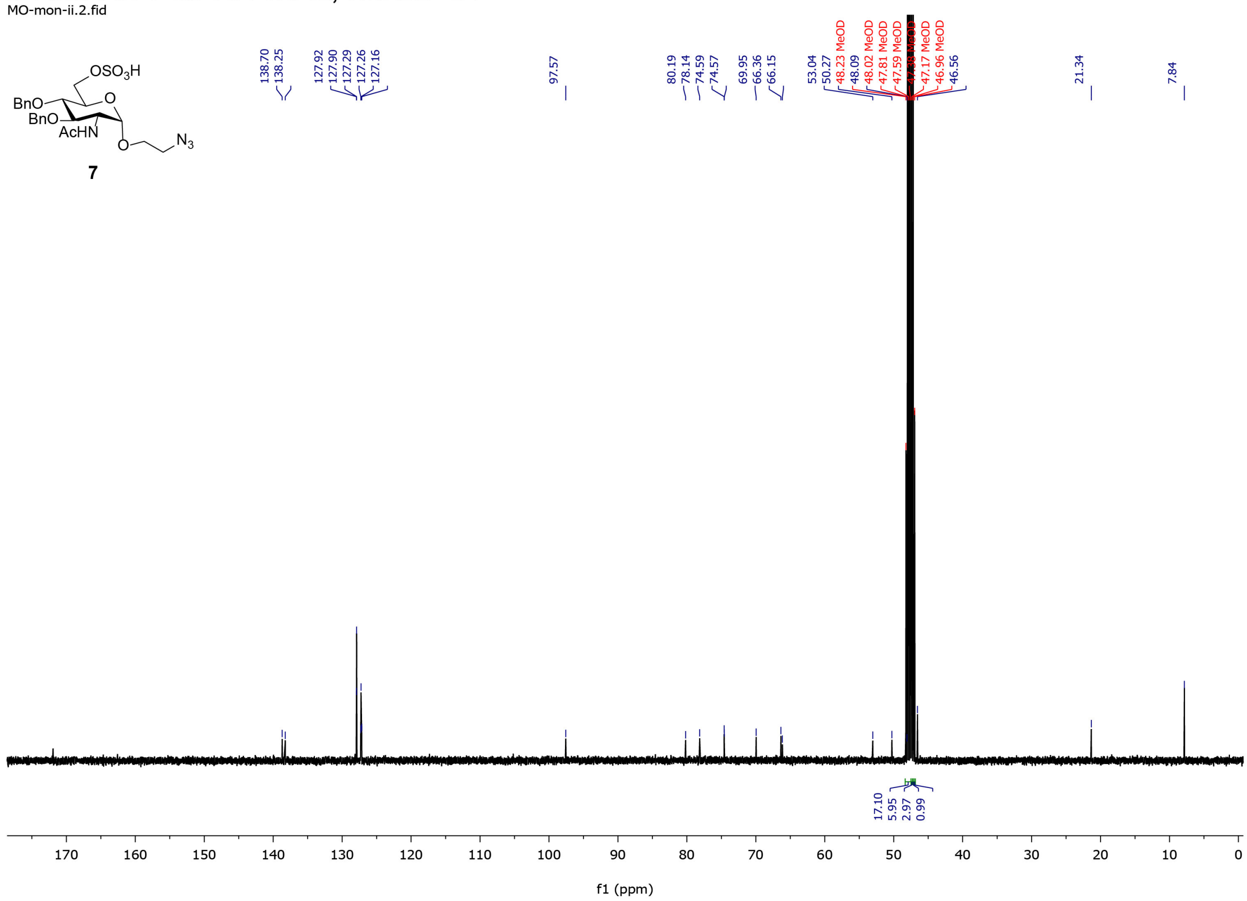

Mass Analysis of **7**

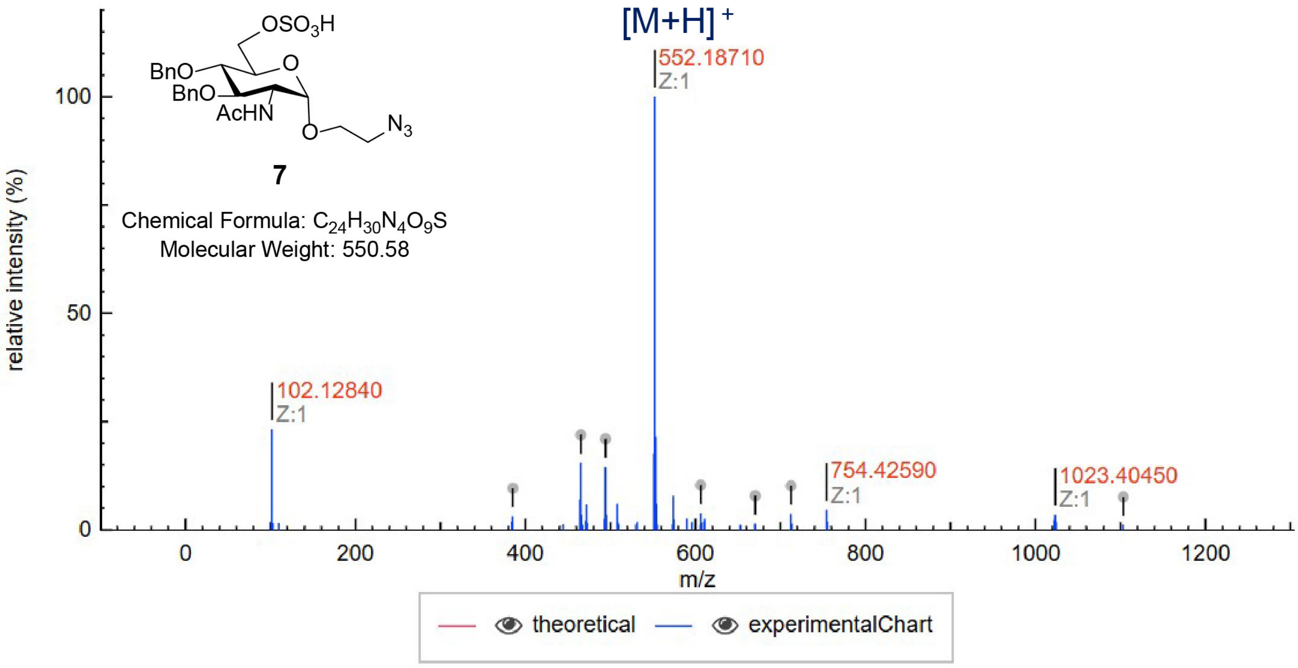

^{1}H NMR of **M1** at 400 MHz, D_2O

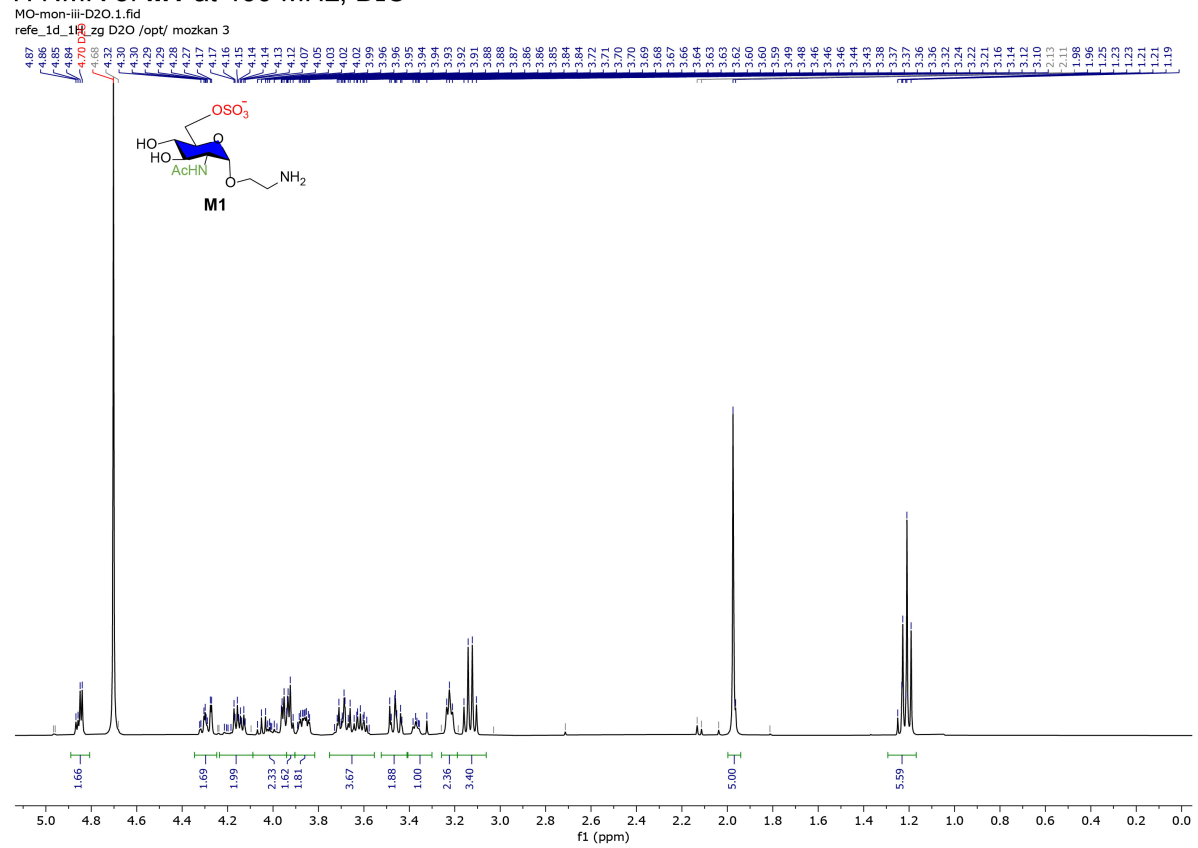

^{13}C NMR of **M1** at 101 MHz, D_2O

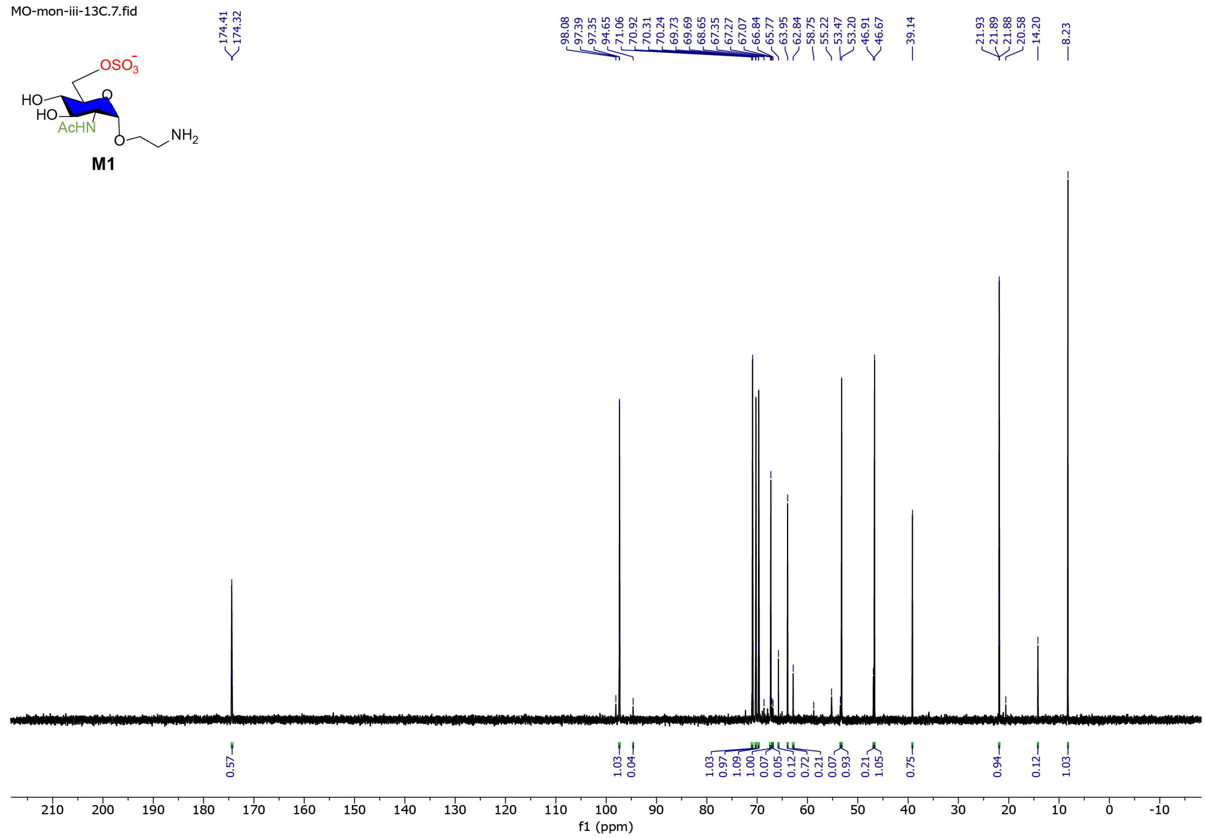

Mass Analysis of **M1**

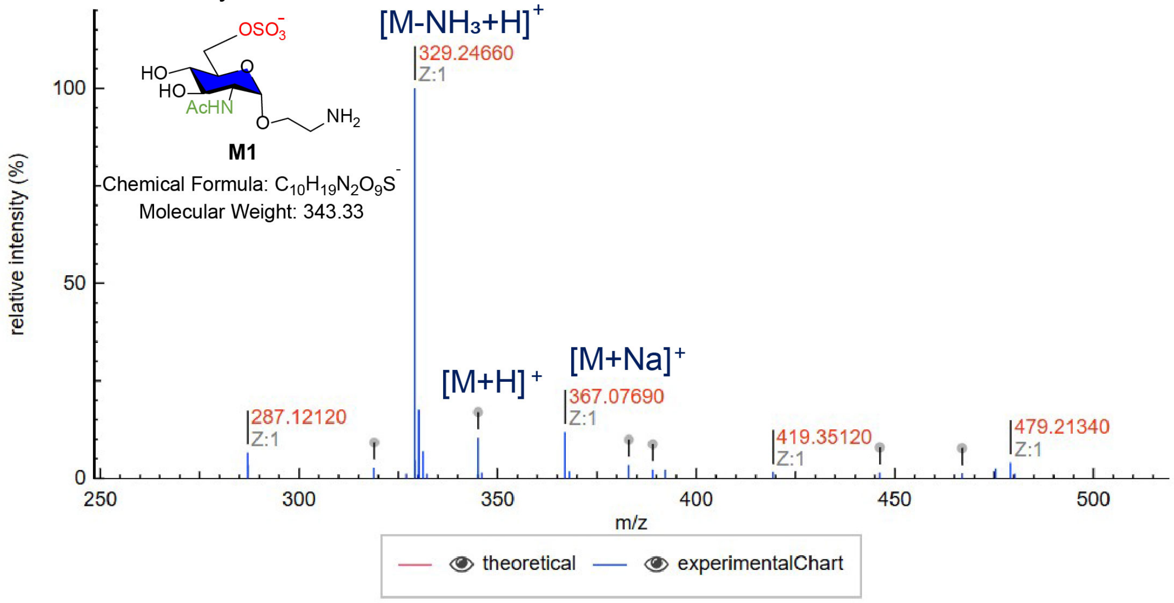

^{1}H NMR of **8** at 400 MHz, $CDCl_3$

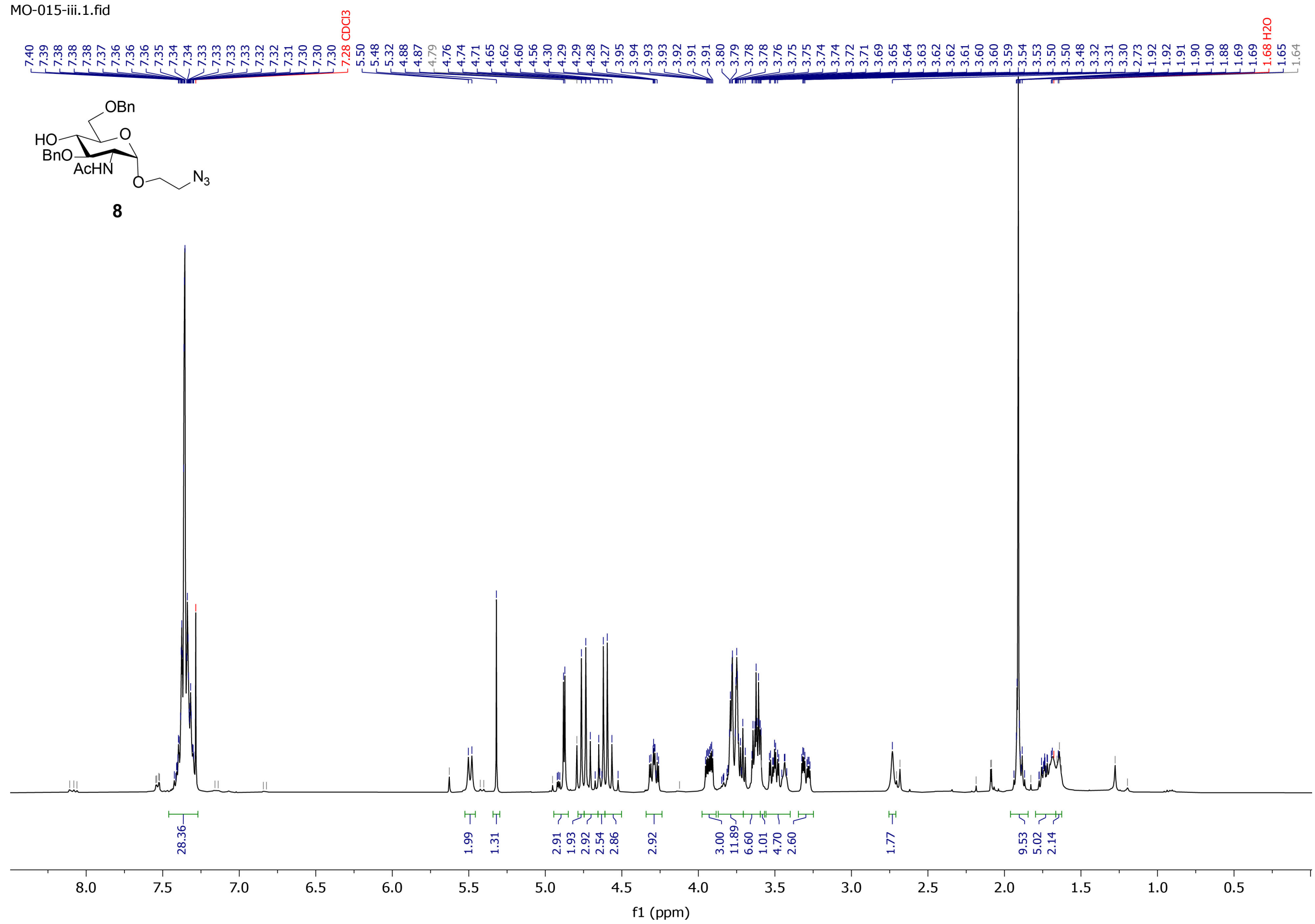

¹³C NMR of **8** at 101 MHz, CDCl₃

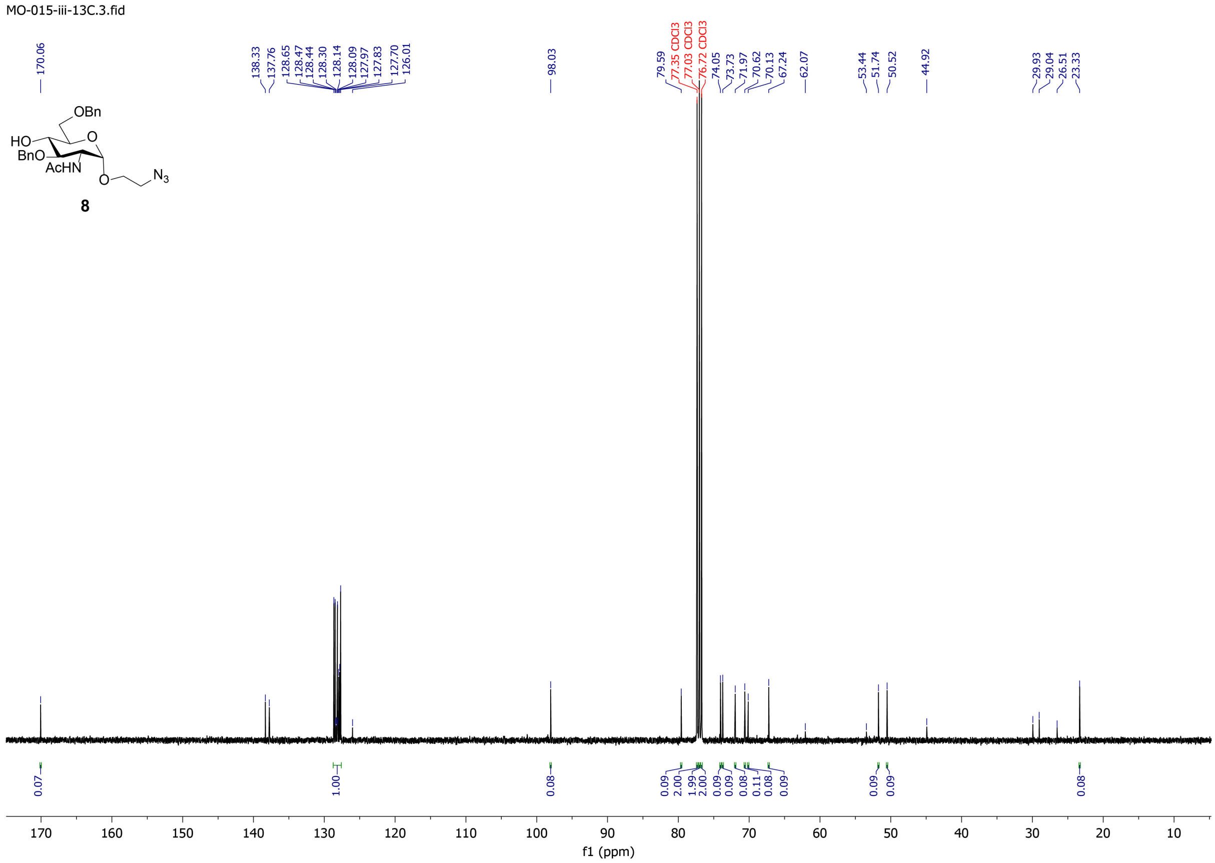

Mass Analysis of **8**

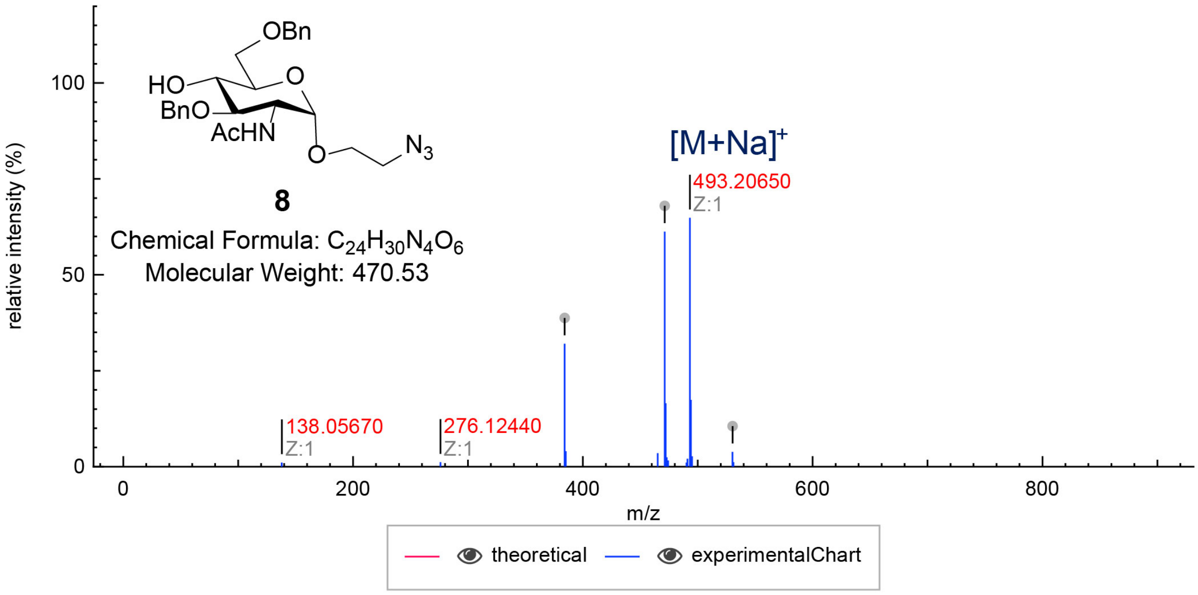

^{1}H NMR of **9** at 400 MHz, $CDCl_3$

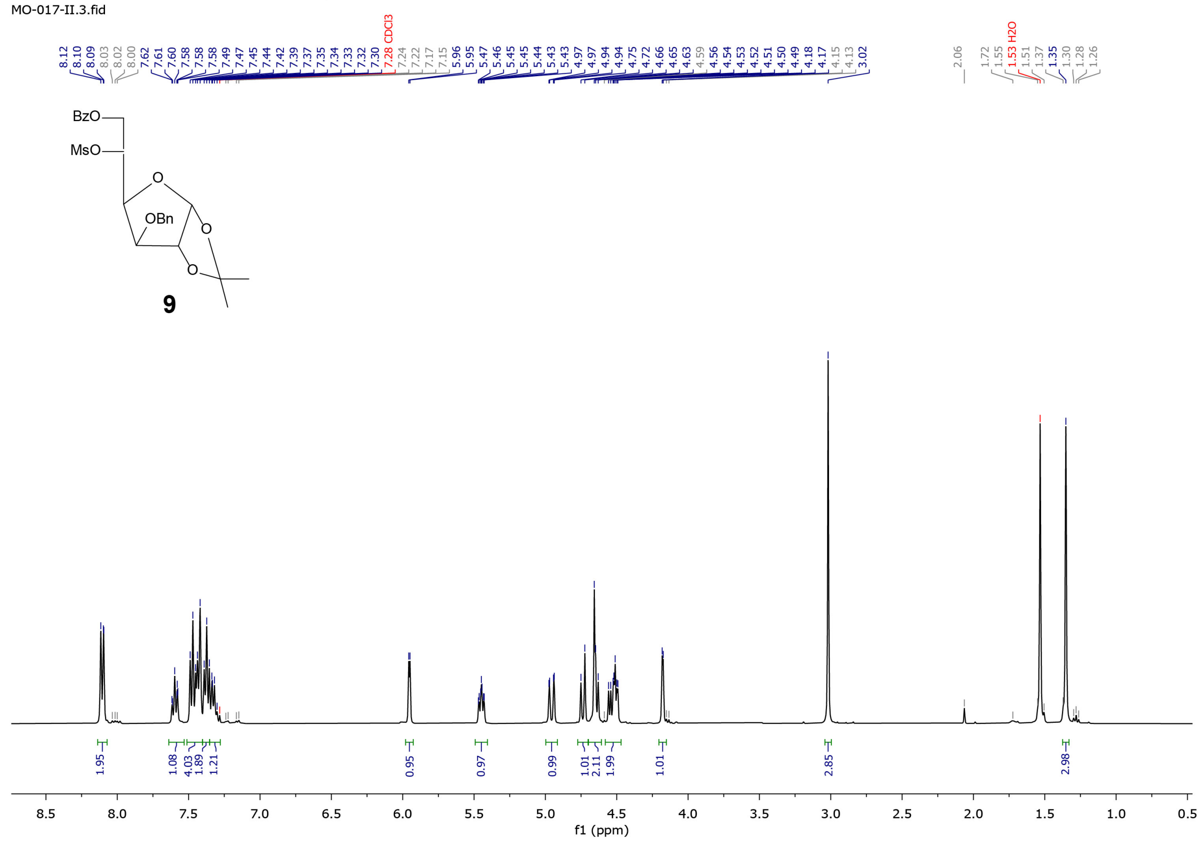

^{13}C NMR of **9** at 101 MHz, $CDCl_3$

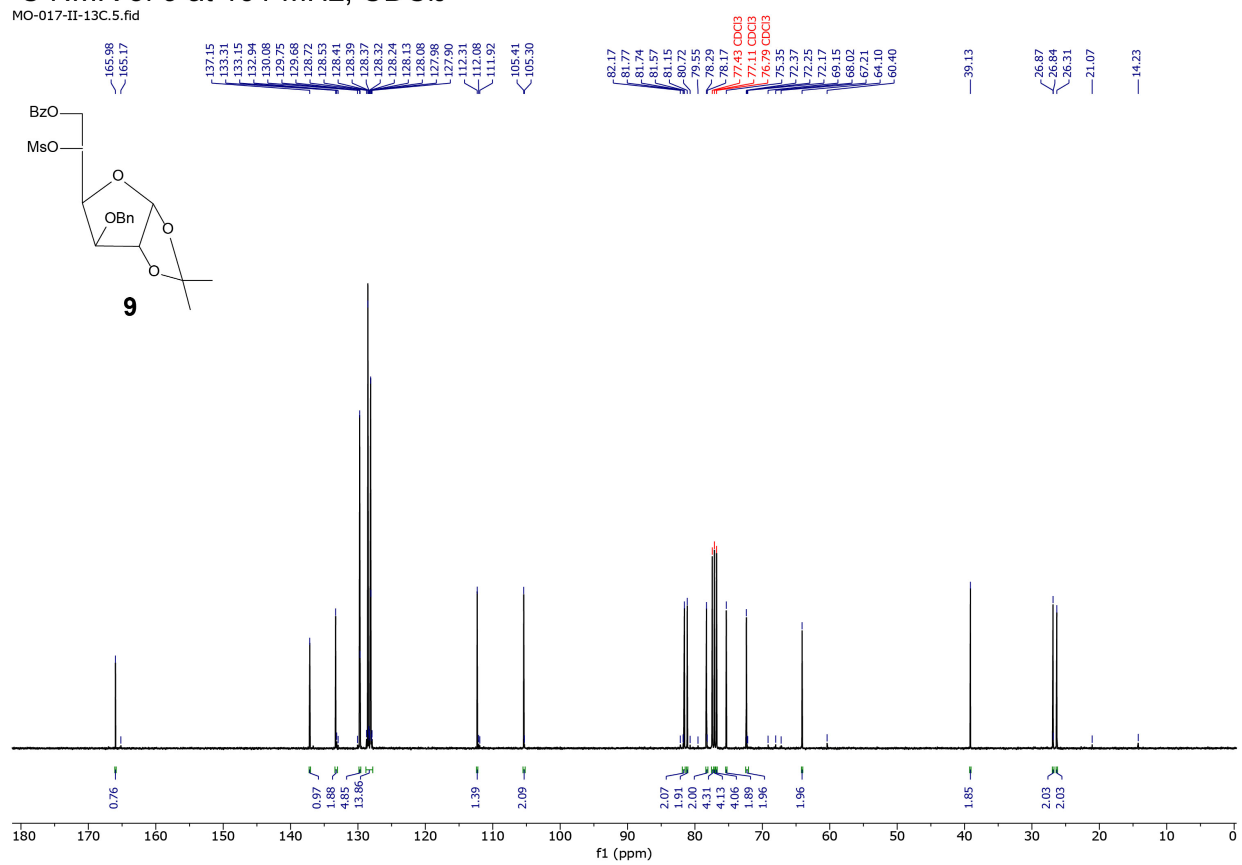

Mass Analysis of **9**

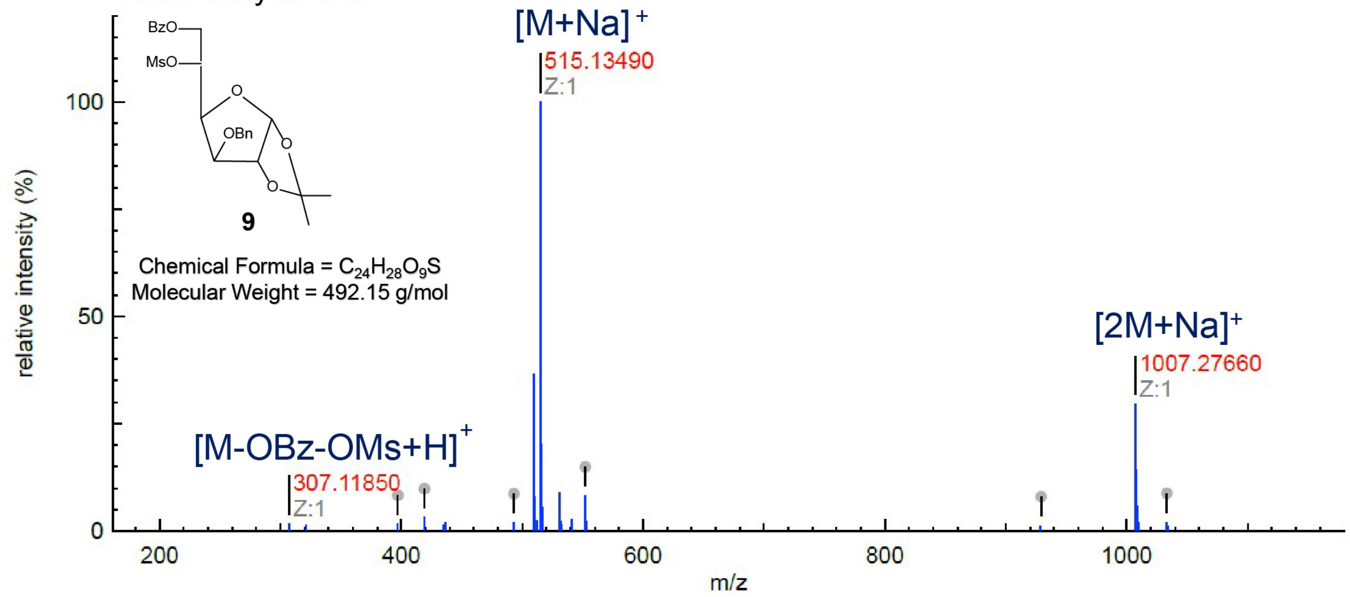

^{1}H NMR of **10** at 400 MHz, $CDCl_3$

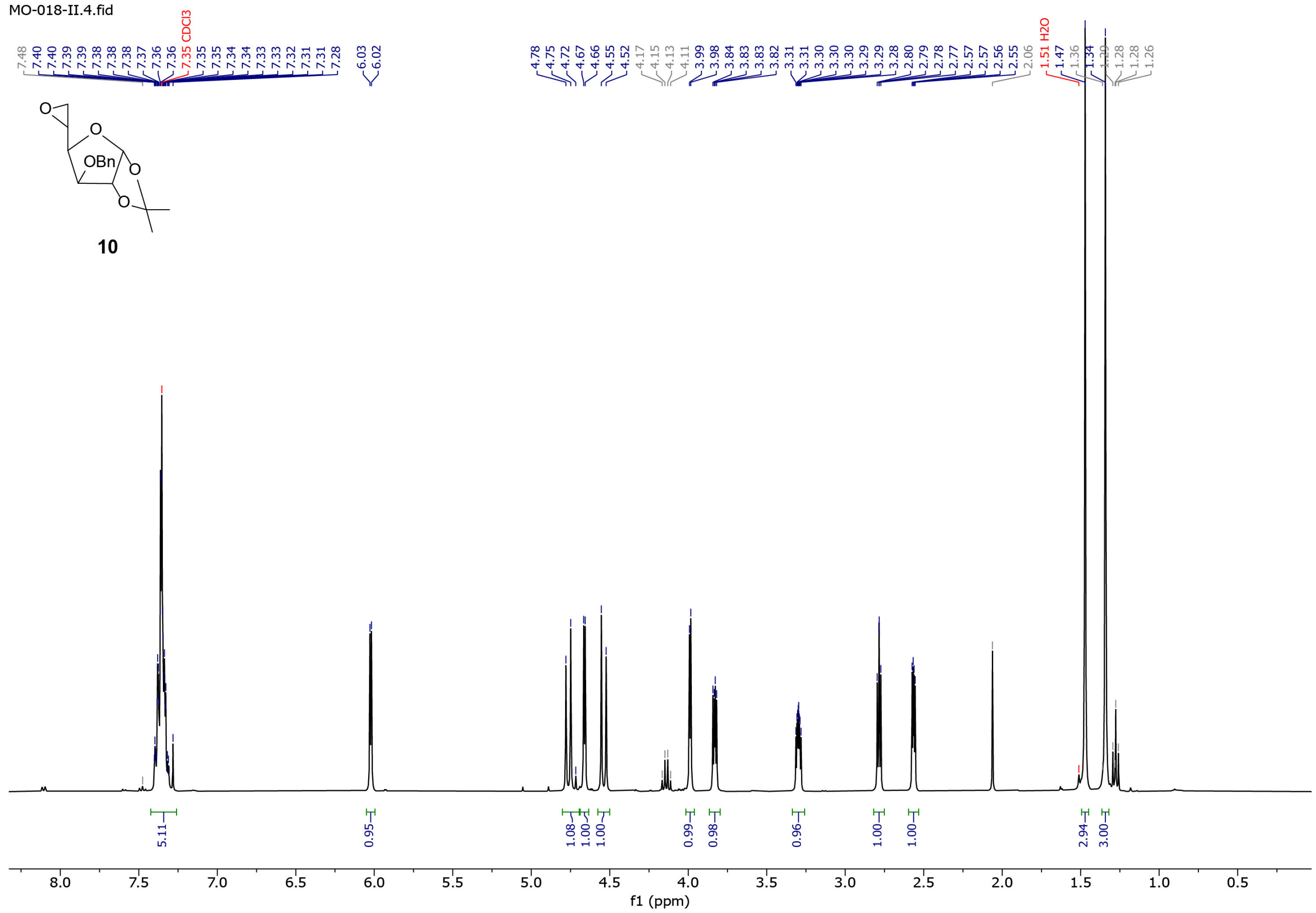

^{13}C NMR of **10** at 101 MHz, $CDCl_3$

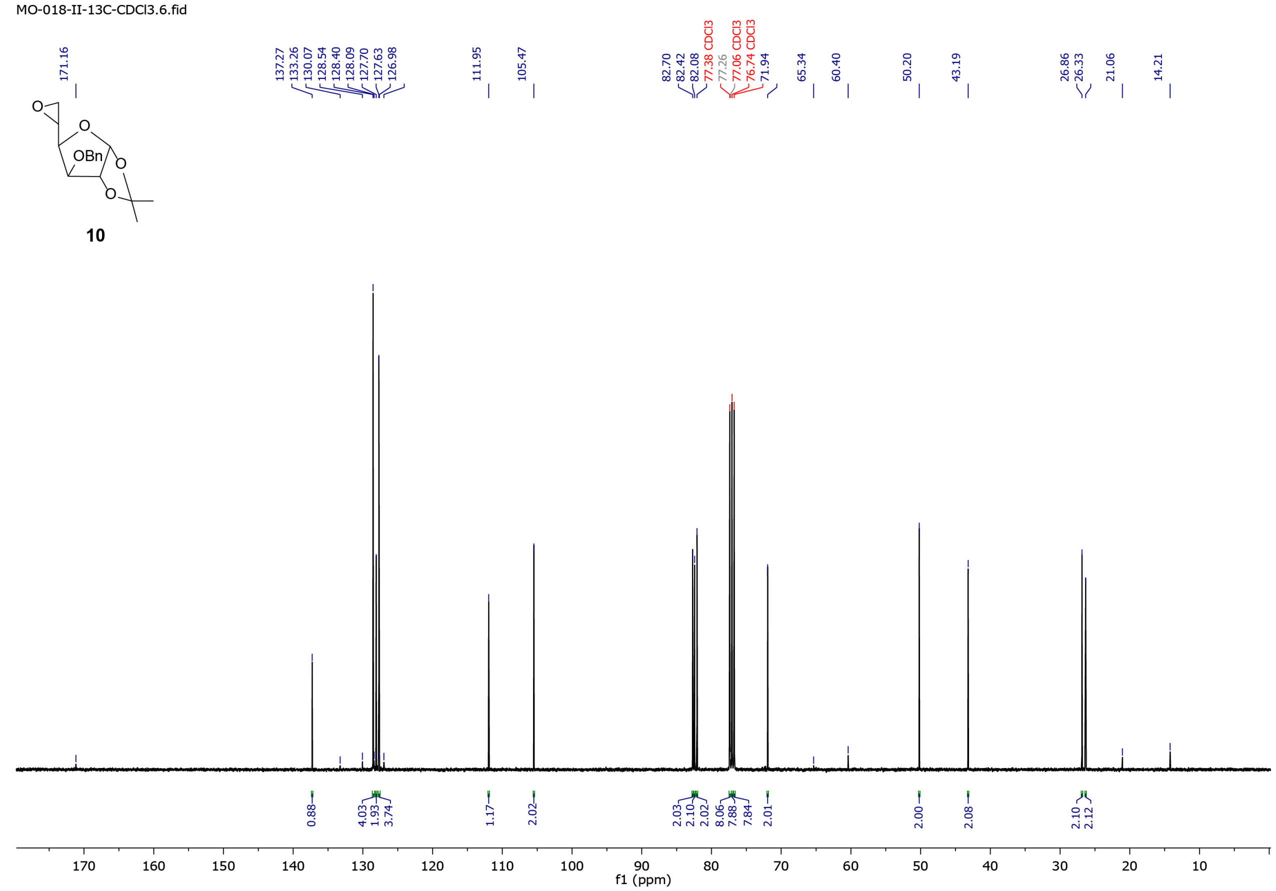

Mass Analysis of **10**

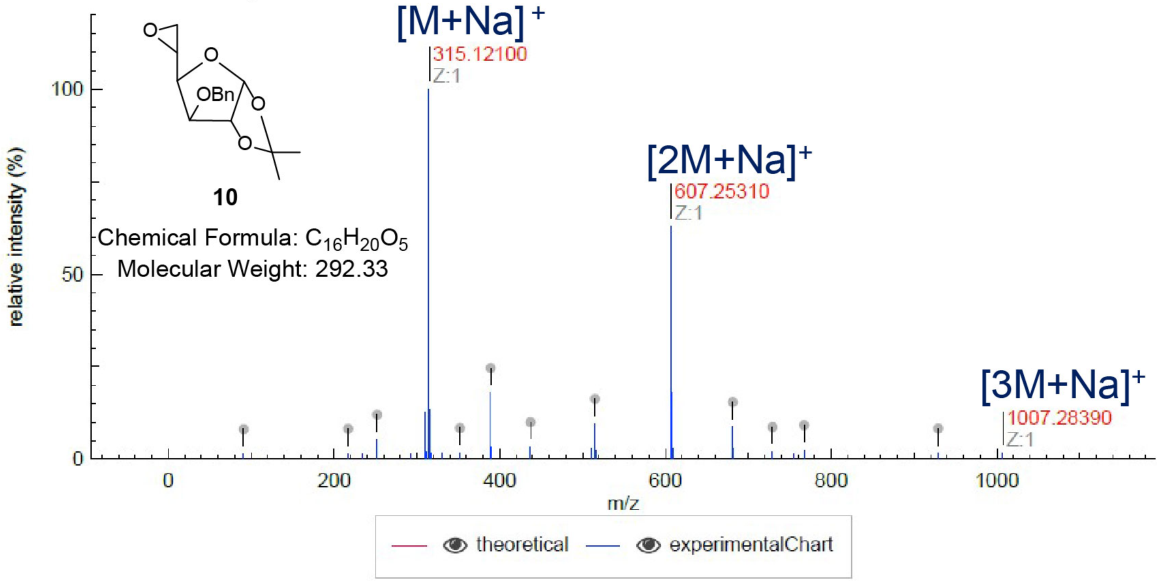

^{1}H NMR of **11** at 400 MHz, $CDCl_3$

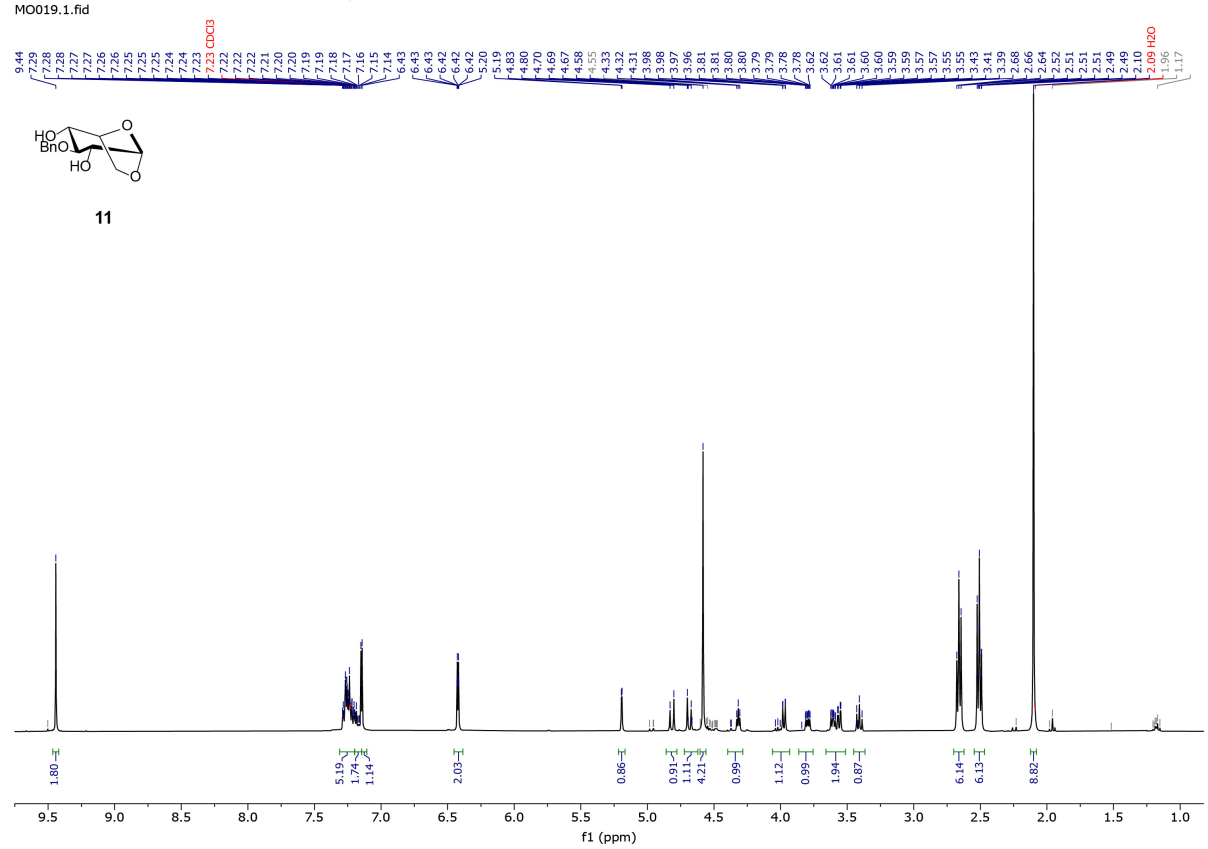

^{13}C NMR of **11** at 101 MHz, $CDCl_3$

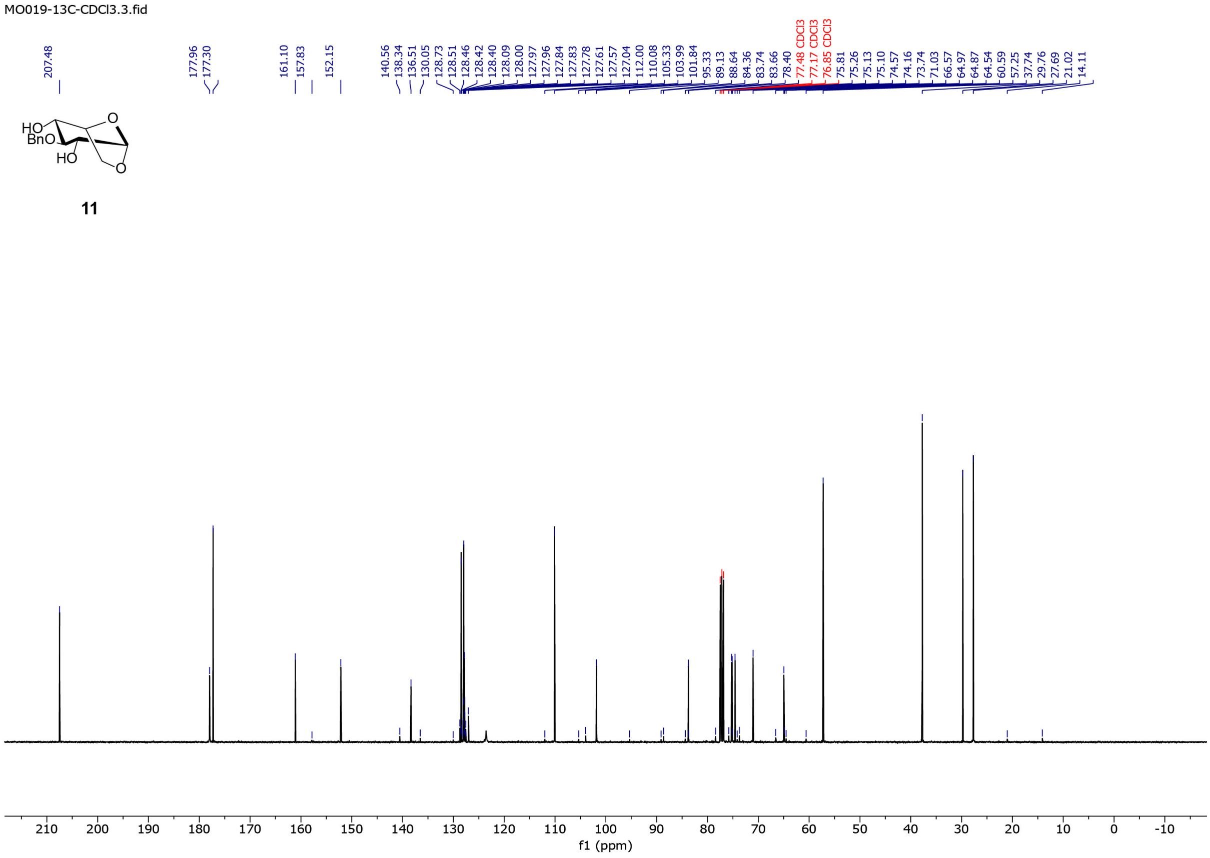

Mass Analysis of **11**

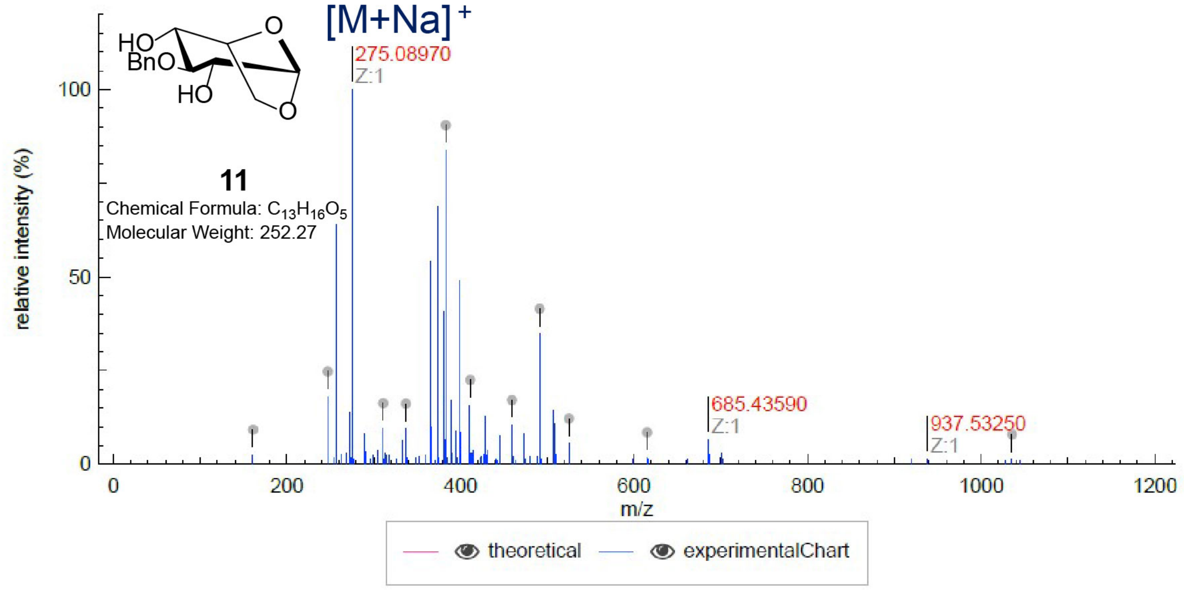

^{1}H NMR of **12** at 400 MHz, $CDCl_3$

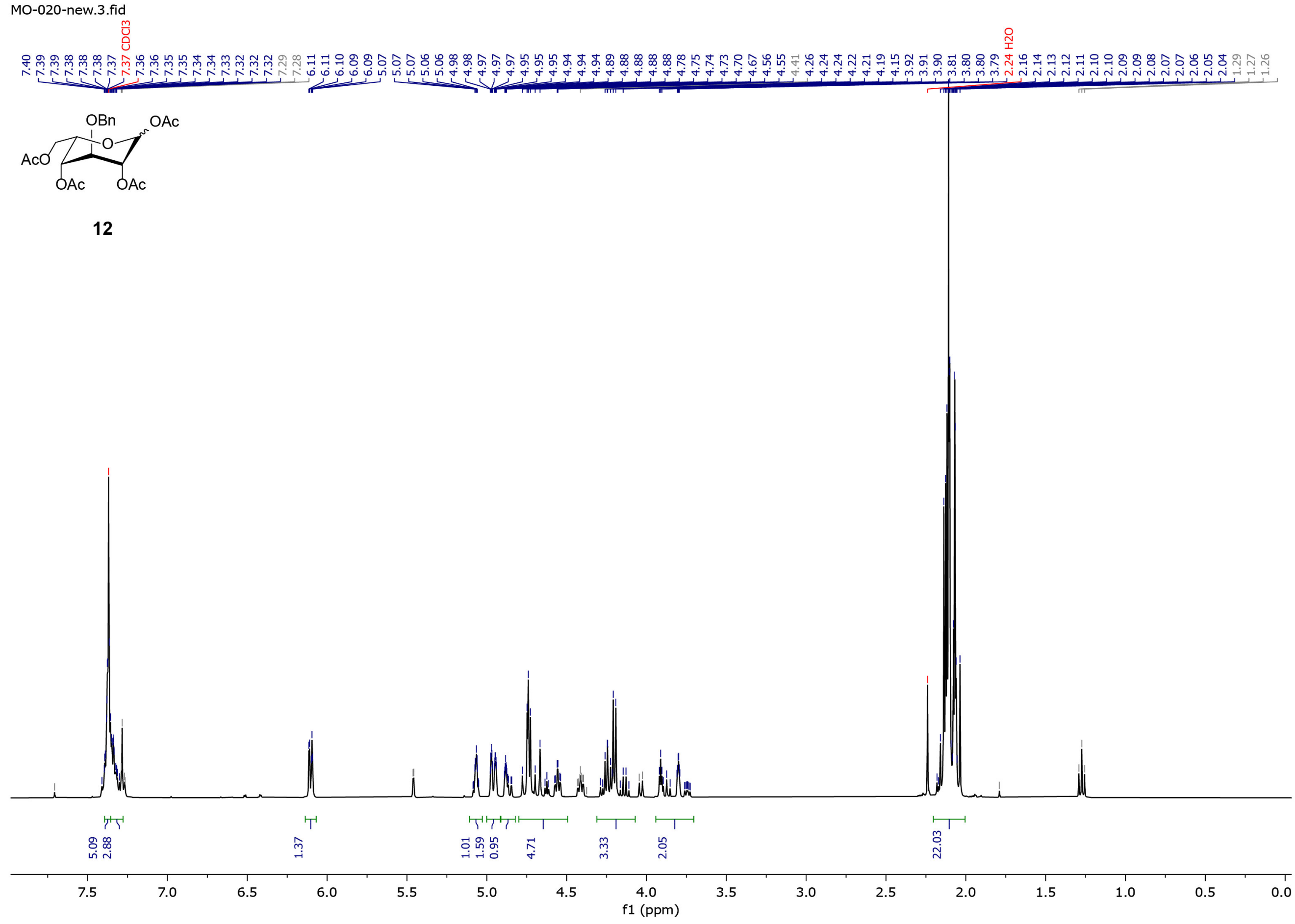

^{13}C NMR of **12** at 101 MHz, $CDCl_3$

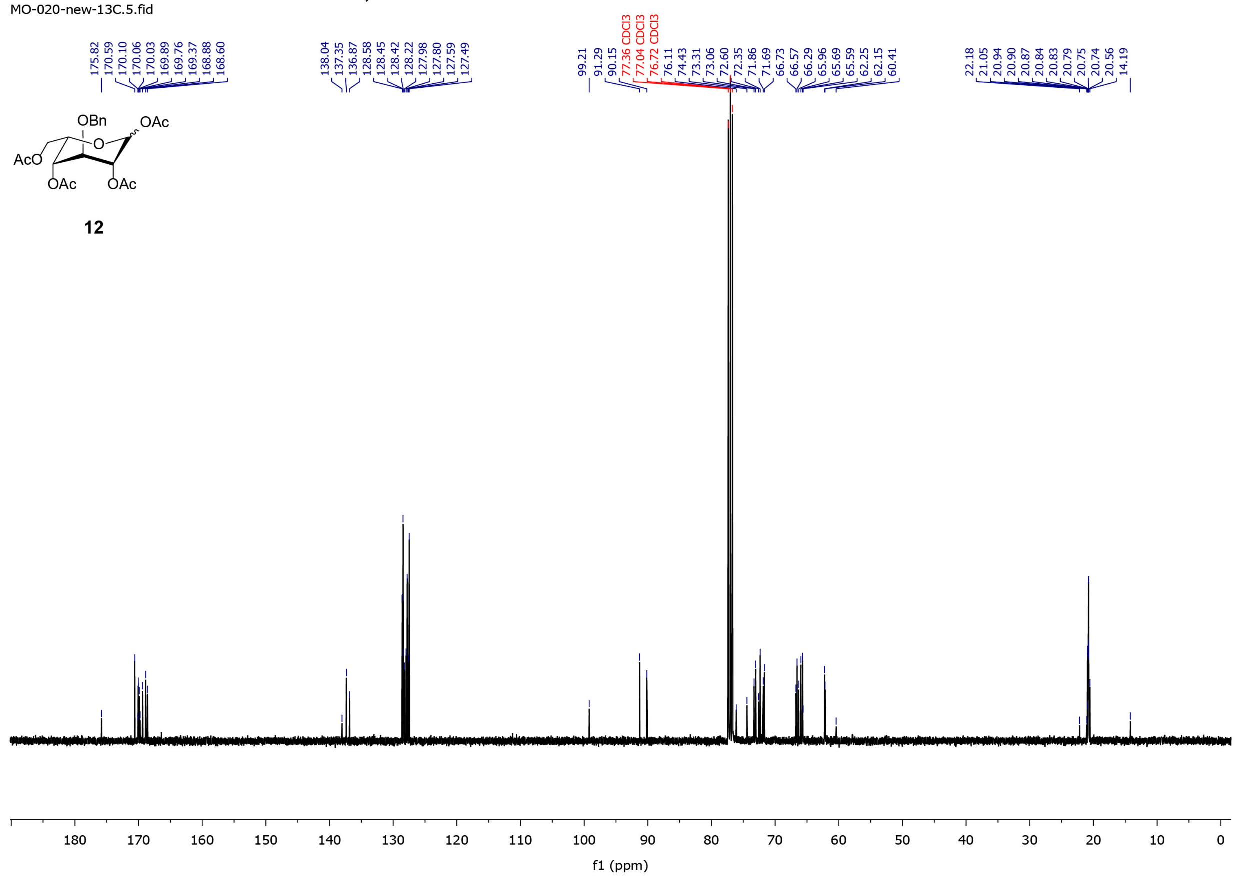

Mass Analysis of **12**

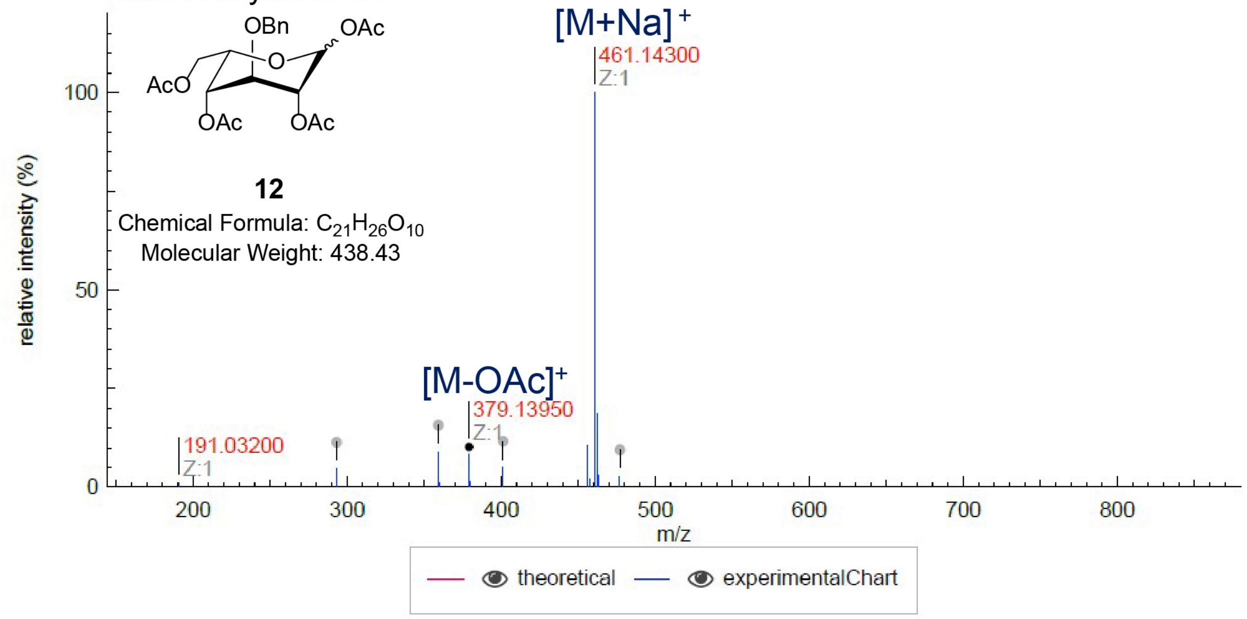

^{1}H NMR of **13** at 400 MHz, $CDCl_3$

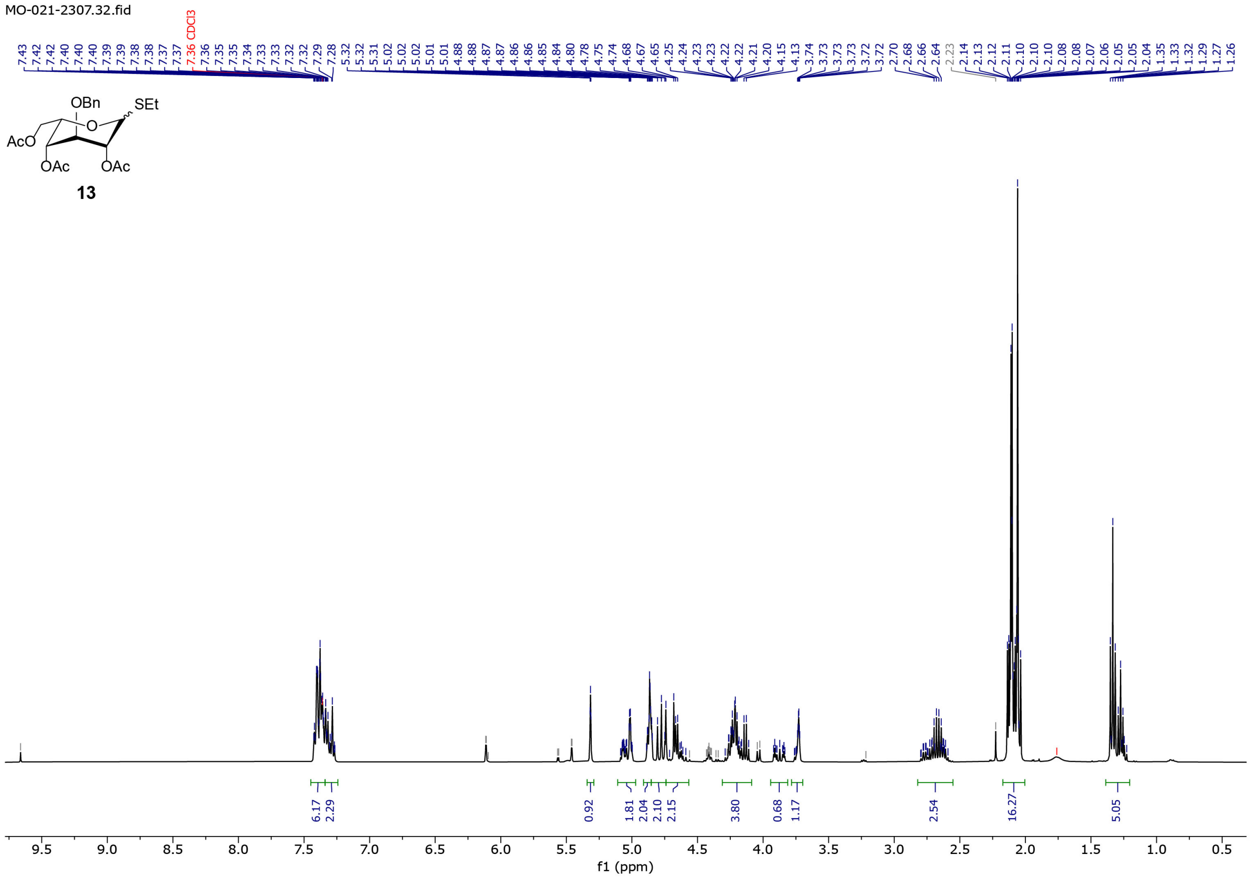

^{13}C NMR of **13** at 101 MHz, $CDCl_3$

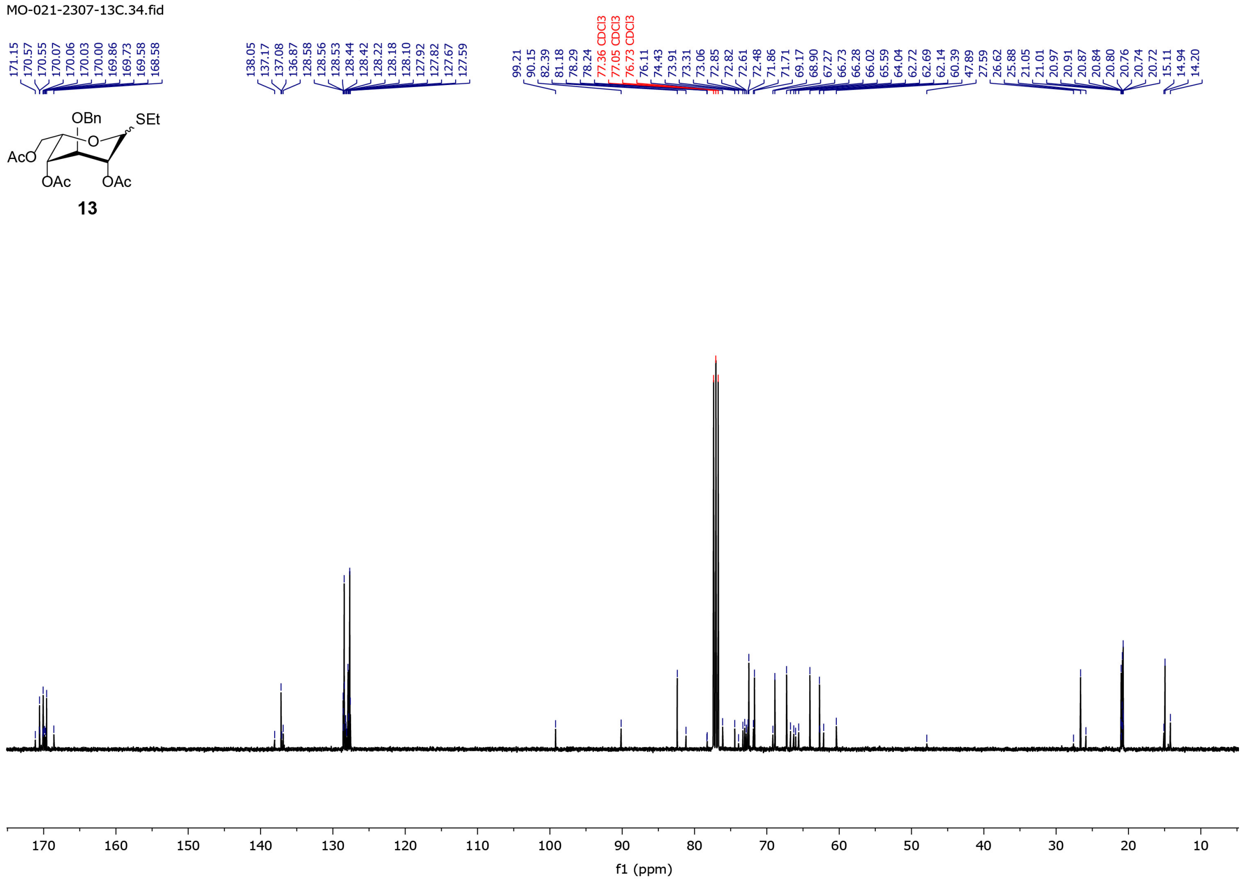

Mass Analysis of **13**

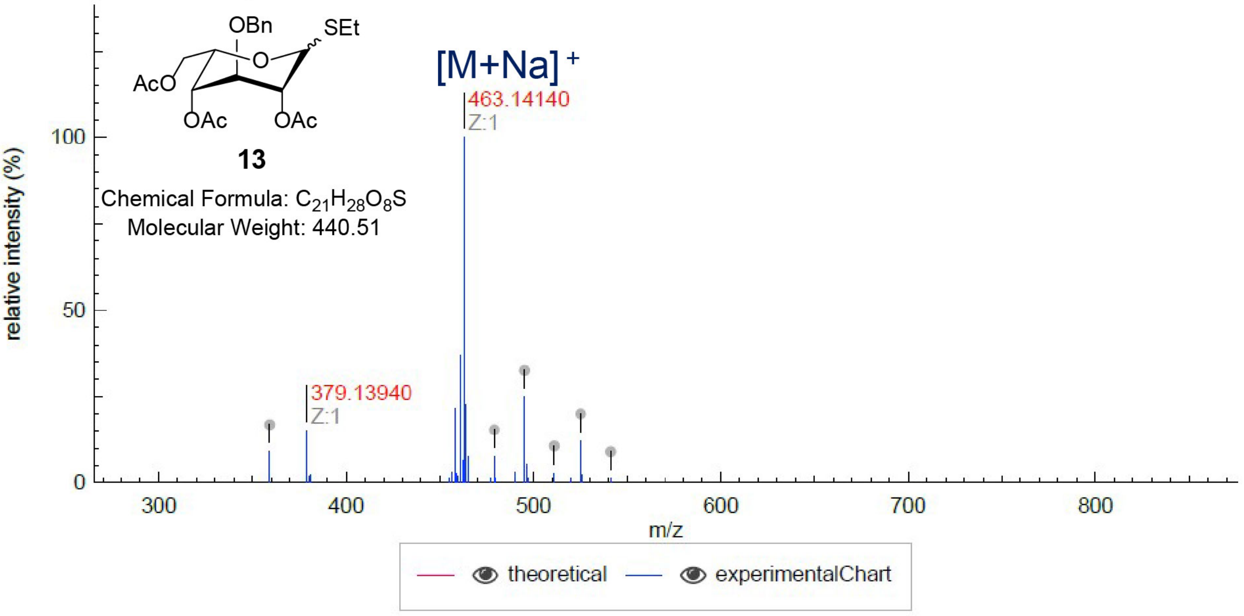

^{1}H NMR of **14** at 400 MHz, $CDCl_3$

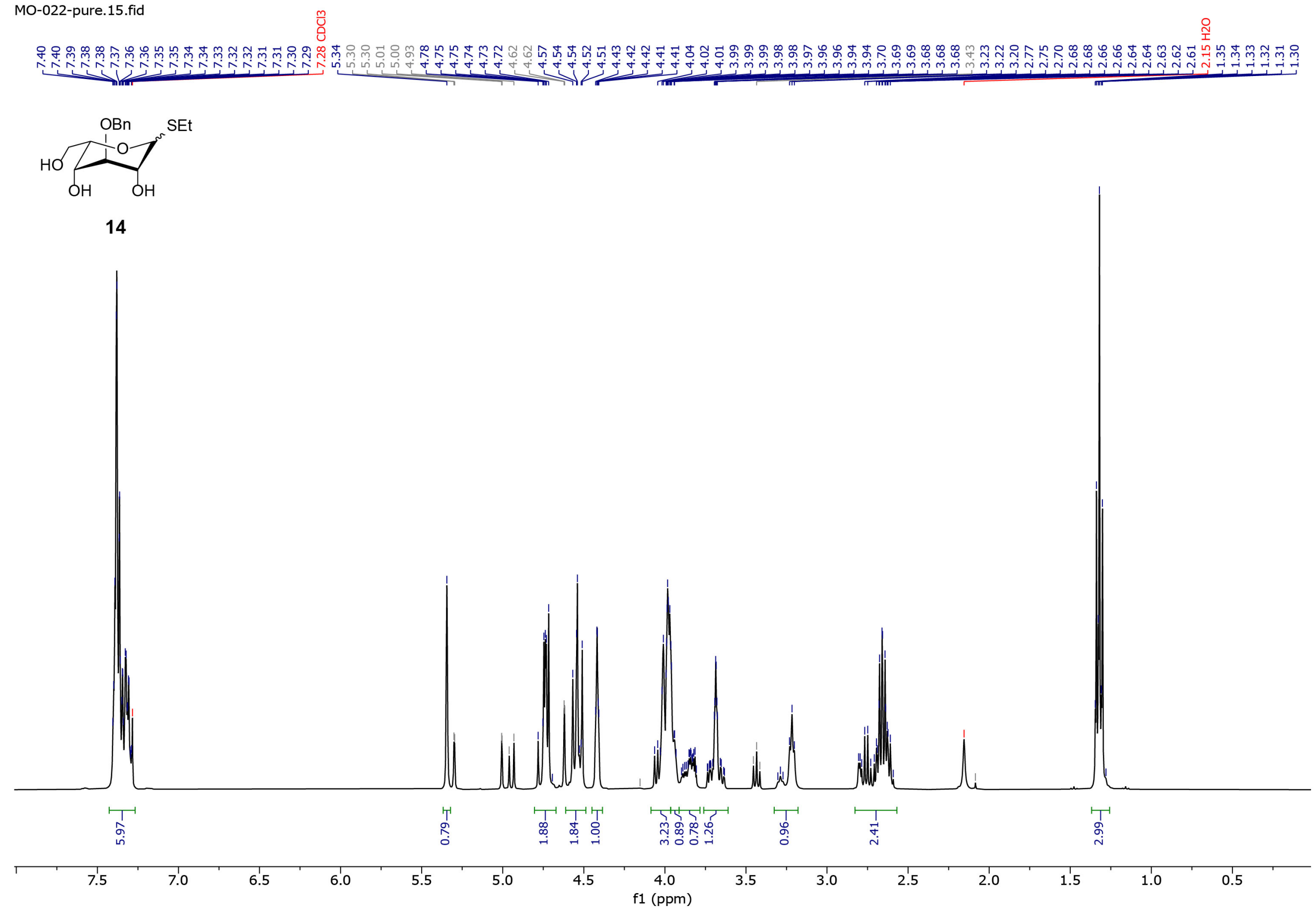

^{13}C NMR of **14** at 101 MHz, $CDCl_3$

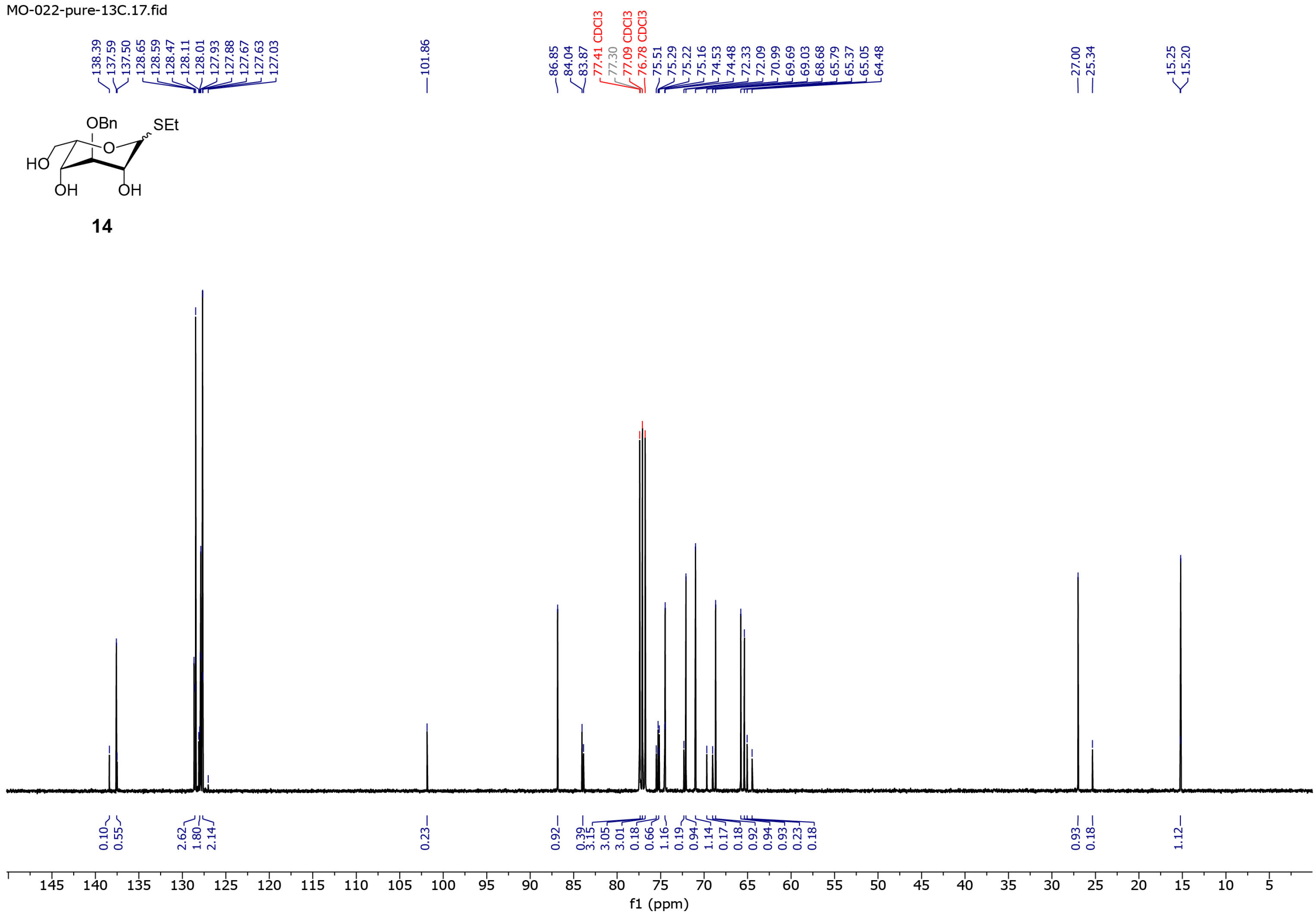

Mass Analysis of **14**

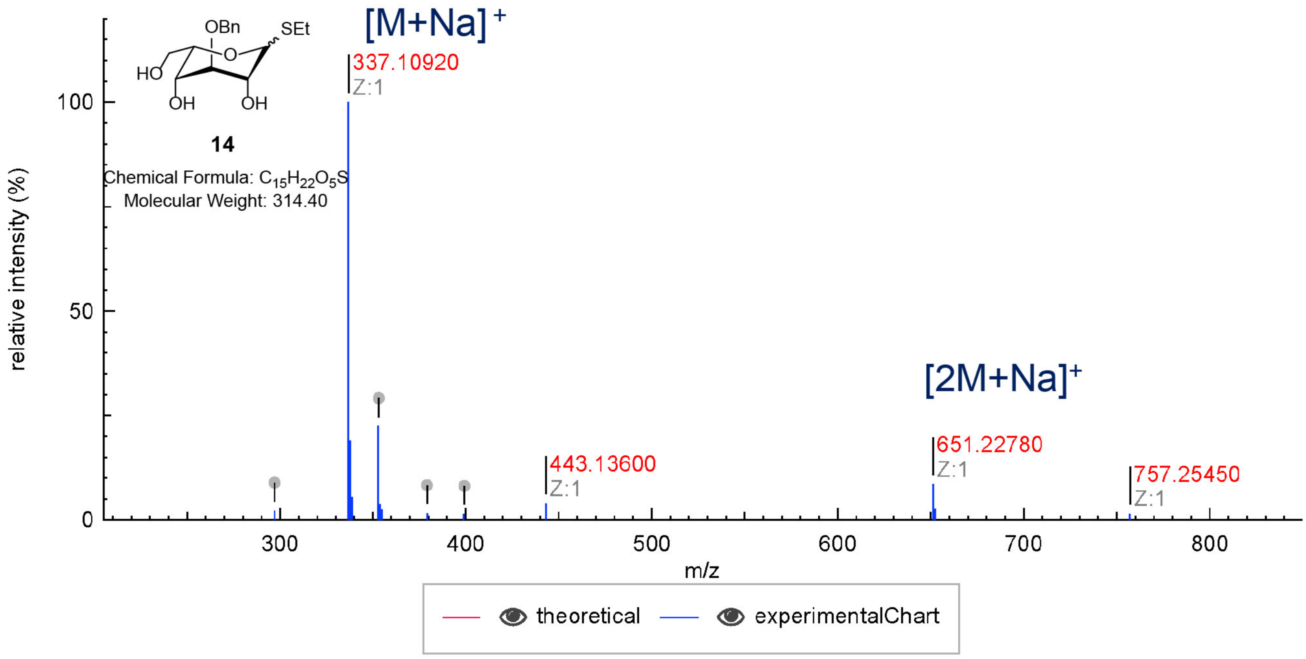

^{1}H NMR of **15** at 400 MHz, $CDCl_3$

MO-023-2607.1.fid

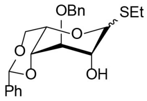

15

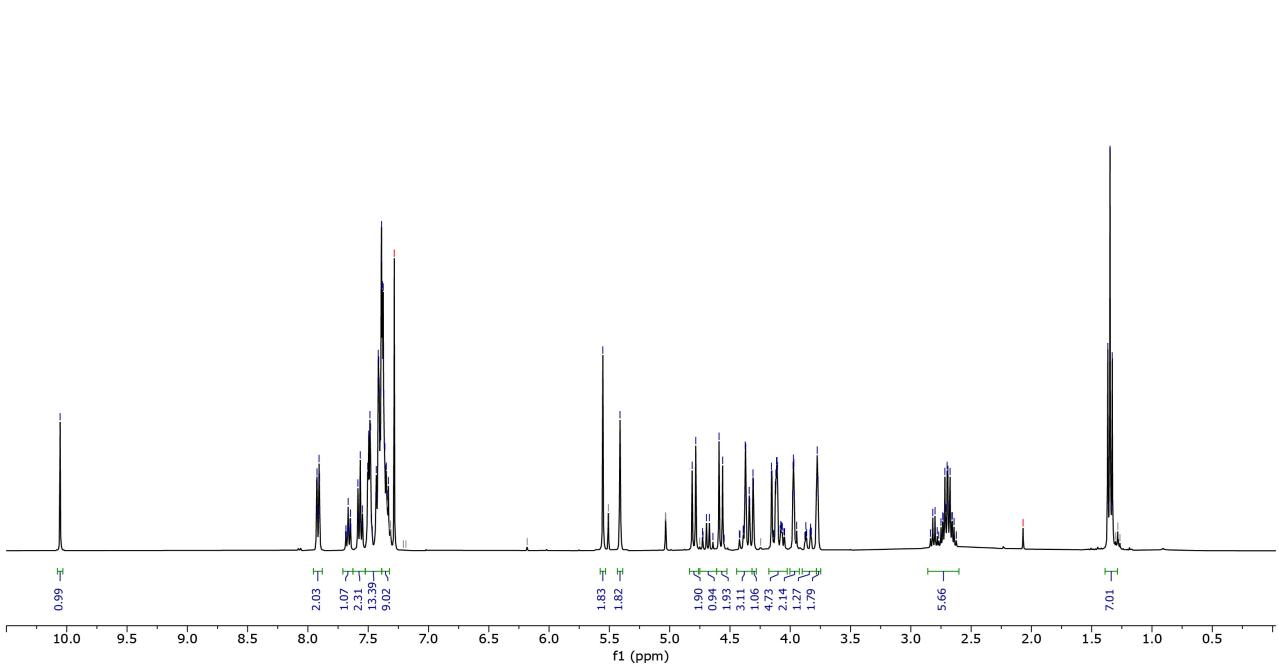

^{13}C NMR of **15** at 101 MHz, $CDCl_3$

MO-023-2607-13C.3.fid

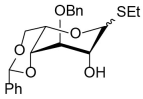

15

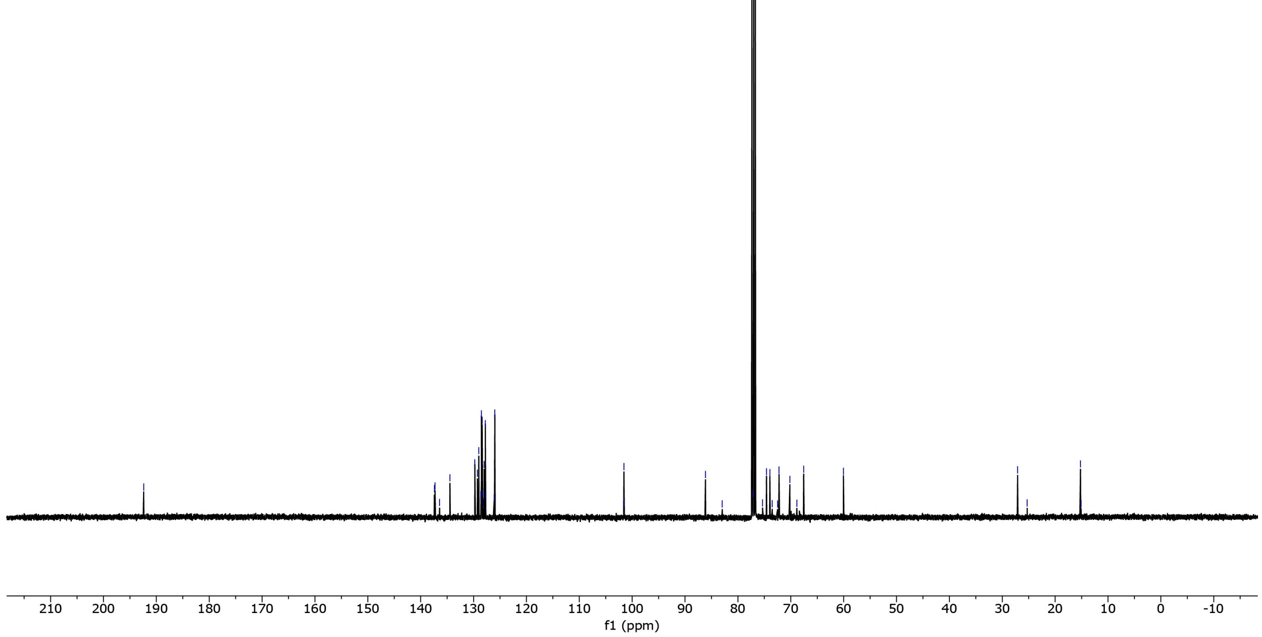

Mass Analysis of **15**

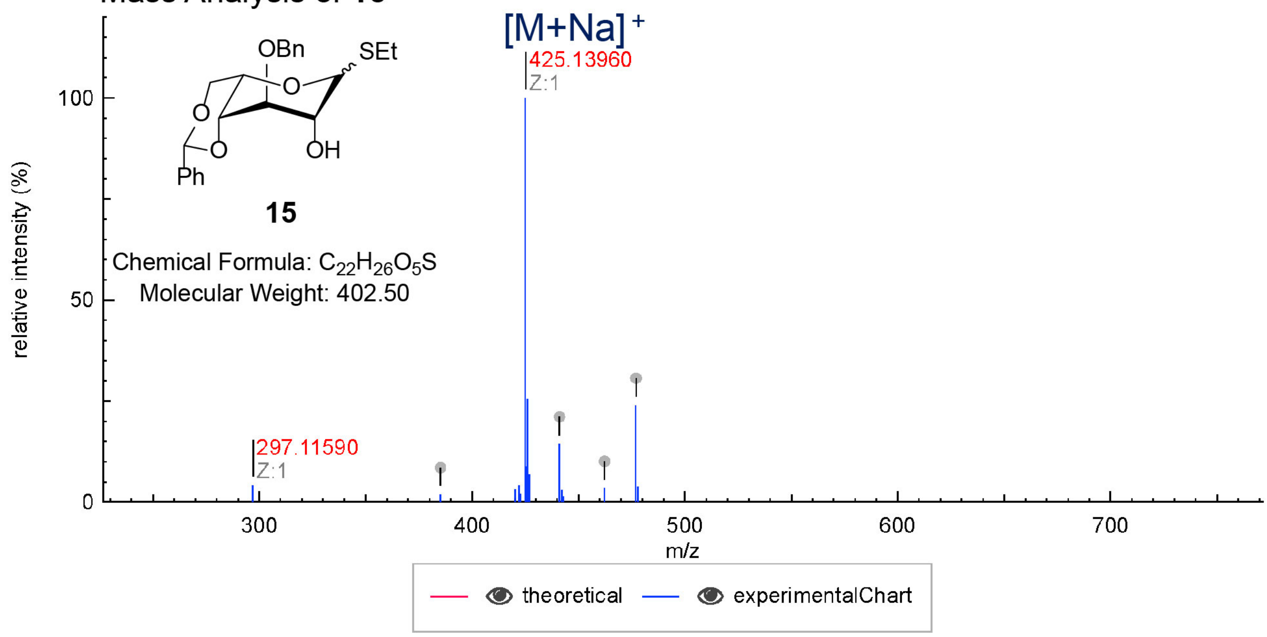

^{1}H NMR of **16** at 400 MHz, $CDCl_3$

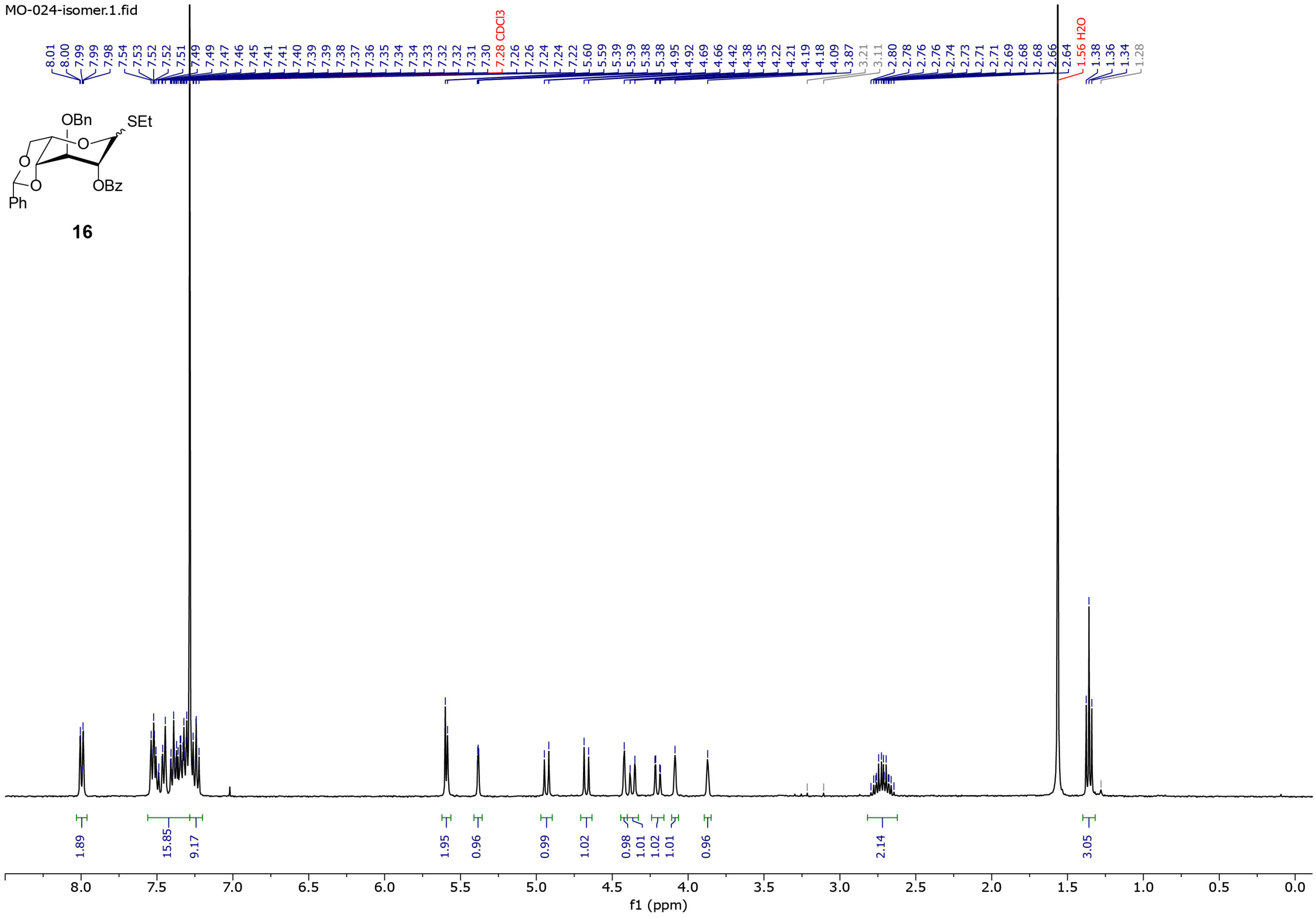

^{13}C NMR of **16** at 101 MHz, $CDCl_3$

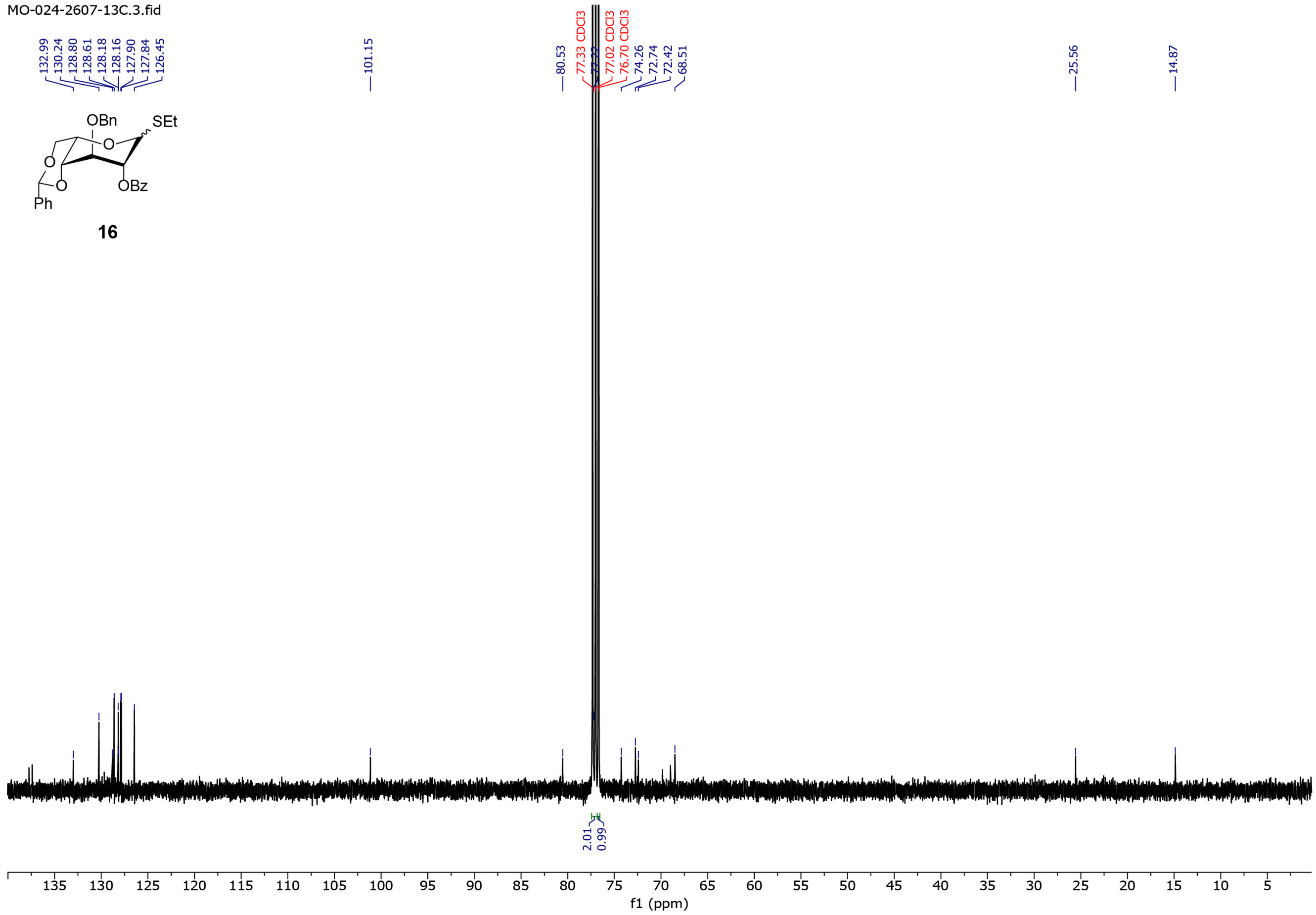

Mass Analysis of **16**

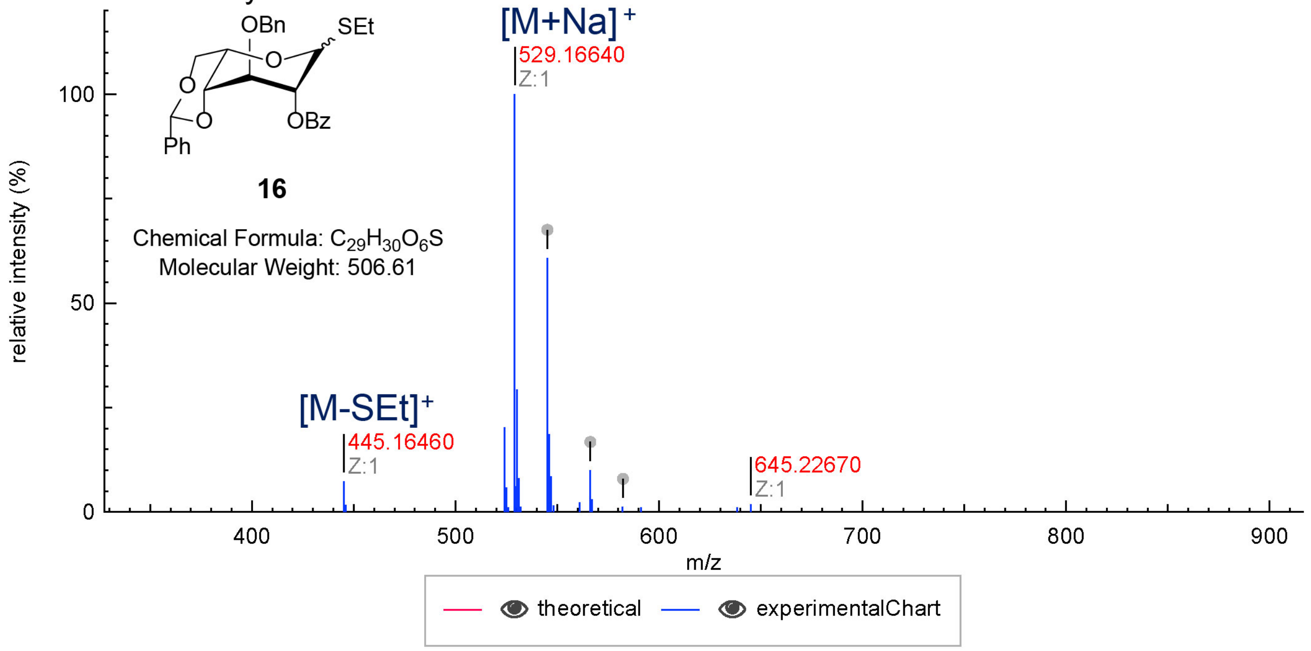

^{1}H NMR of **17** at 400 MHz, $CDCl_3$

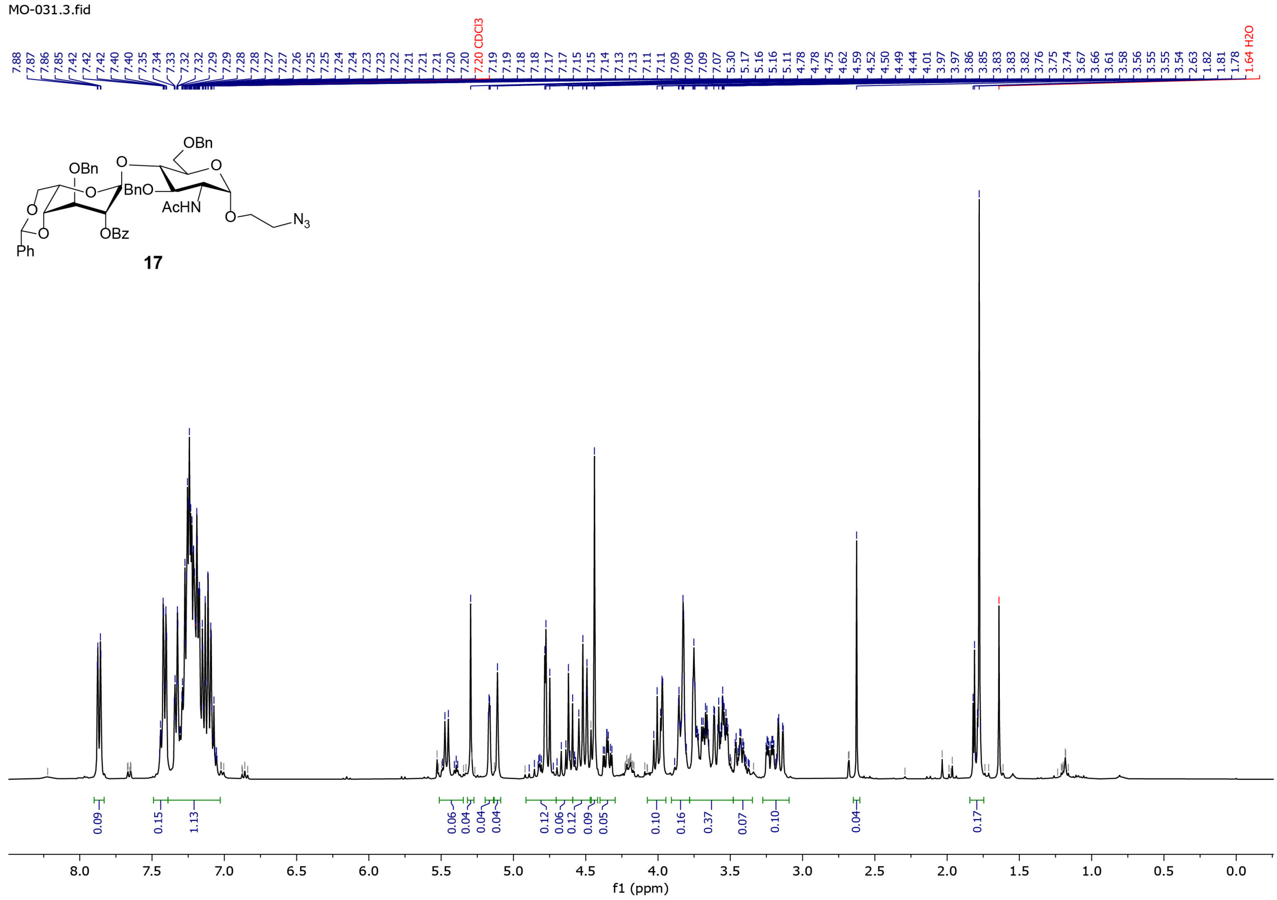

^{13}C NMR of **17** at 101 MHz, $CDCl_3$

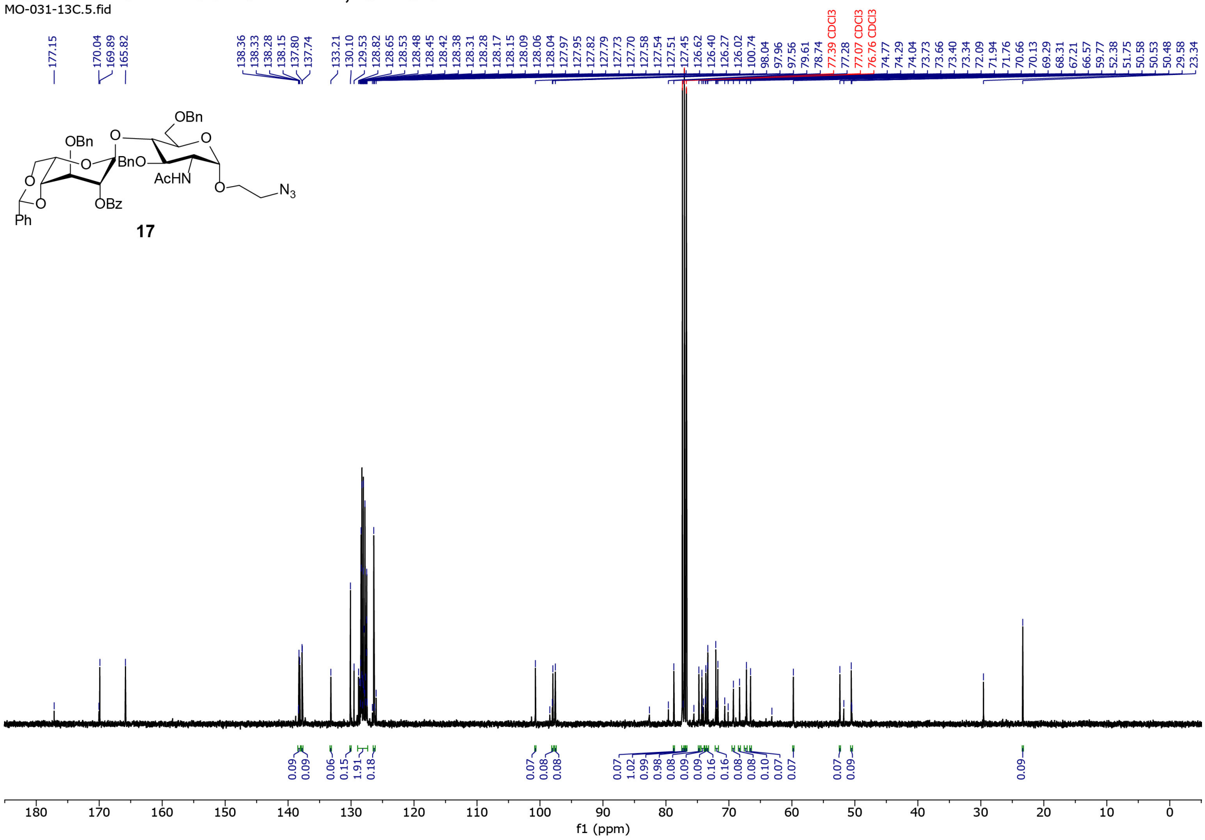

Mass Analysis of **17**

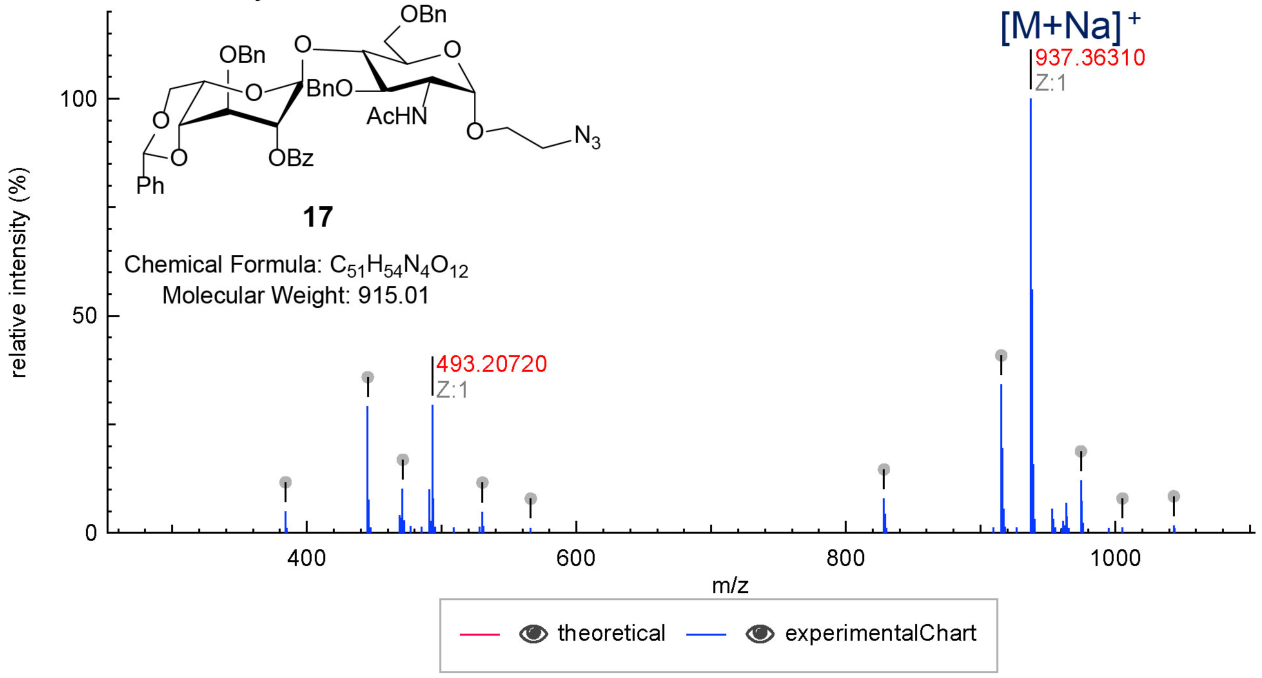

^{1}H NMR of **18** at 400 MHz, $CDCl_3$

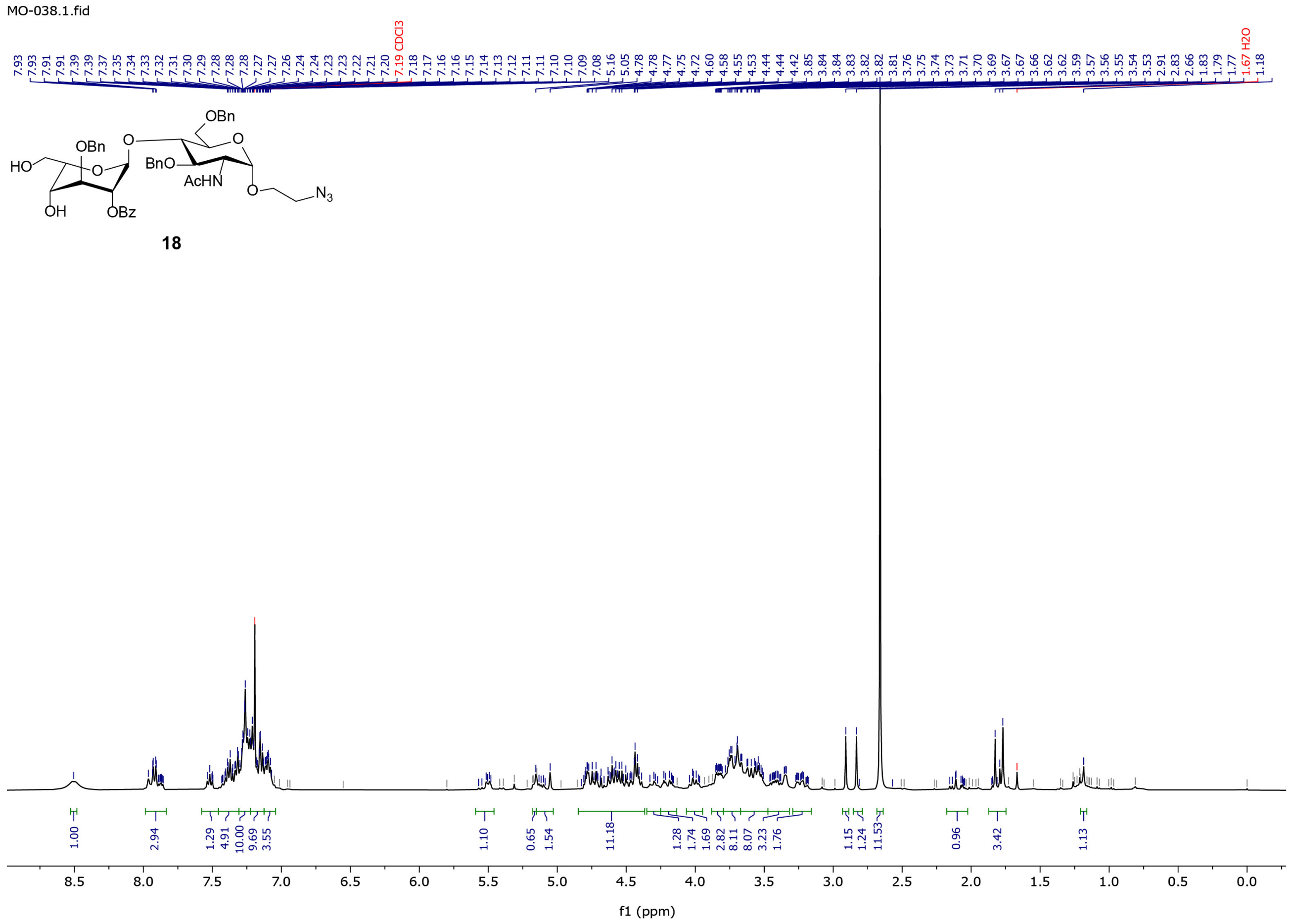

^{13}C NMR of **18** at 101 MHz, $CDCl_3$

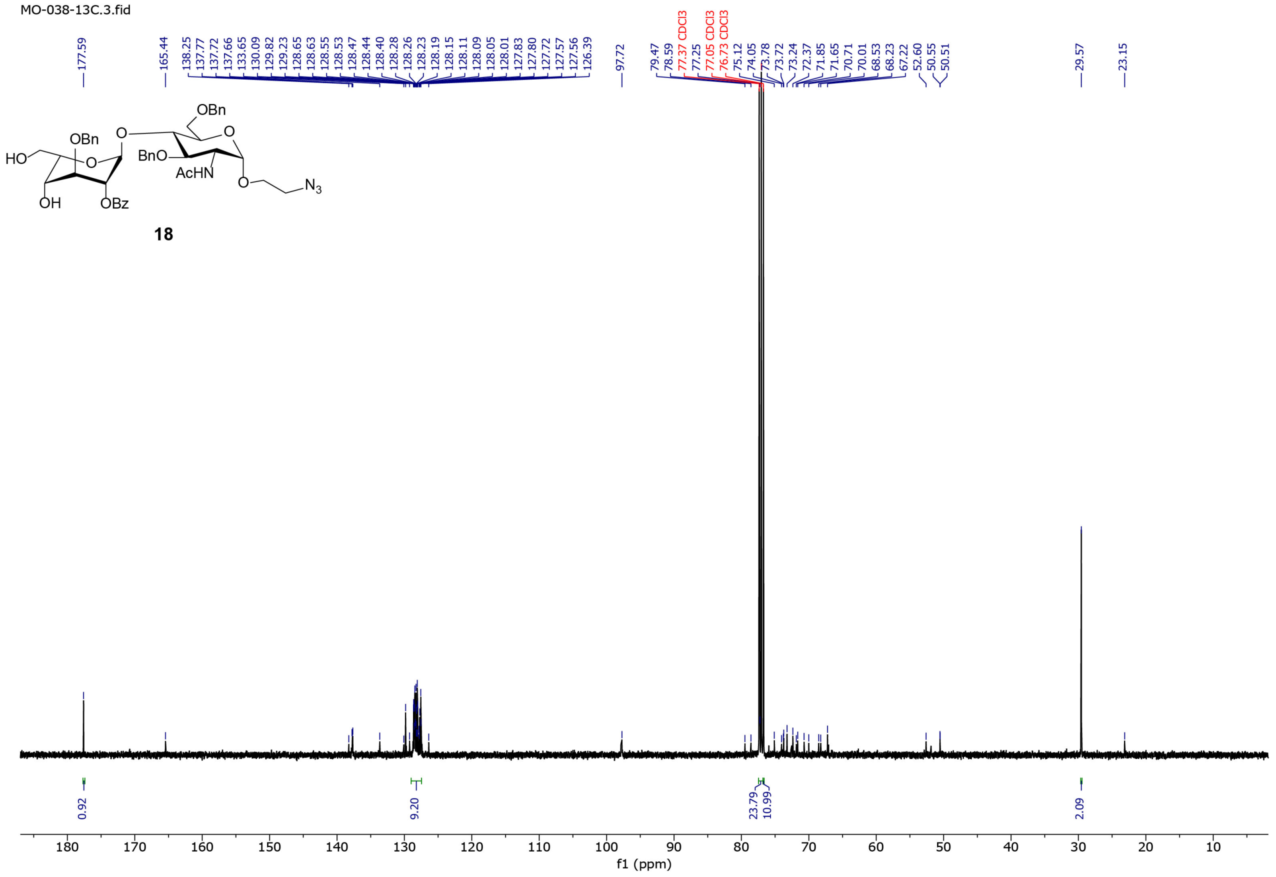

^1H-^1H COSY NMR of **18** at 400 MHz, $CDCl_3$

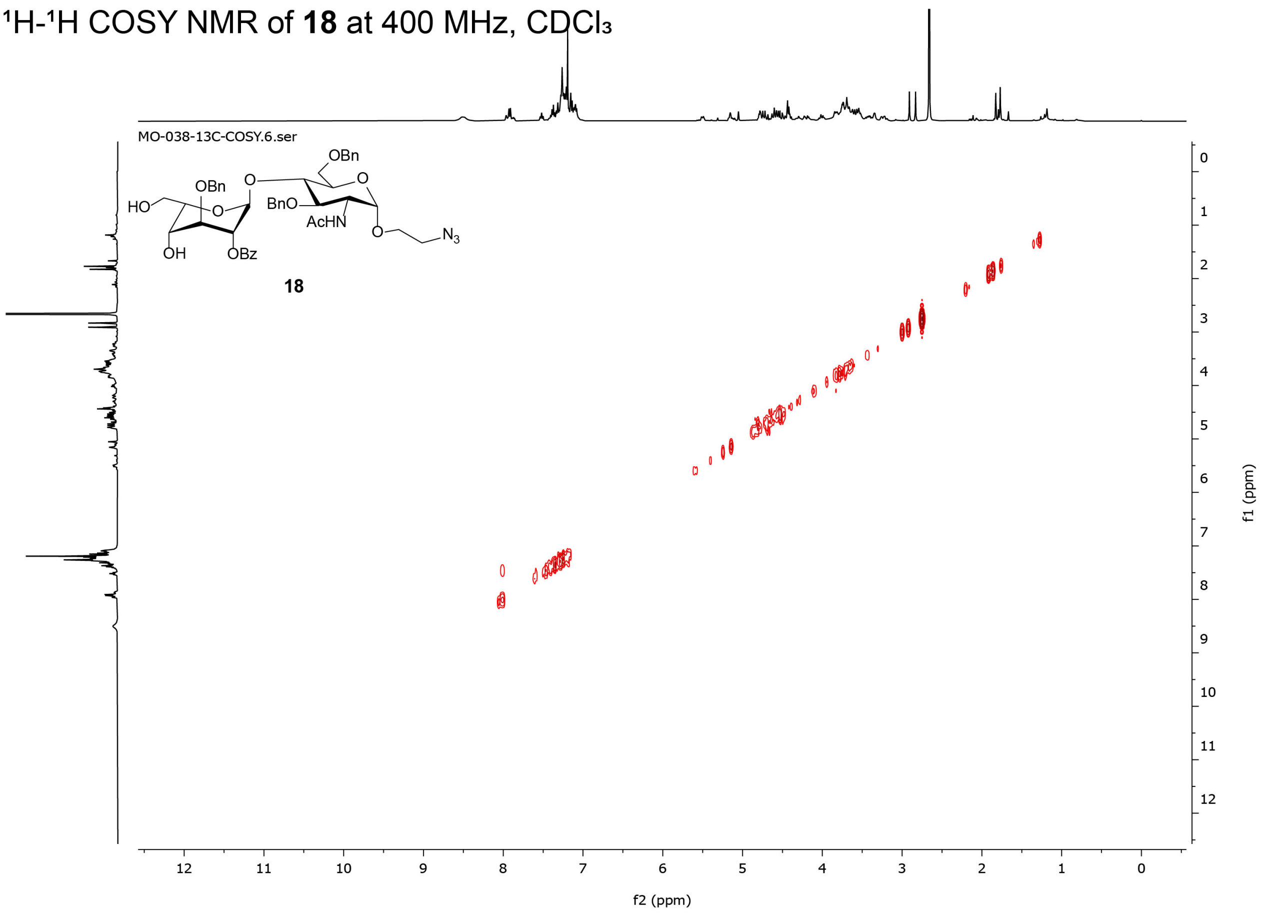

Mass Analysis of **18**

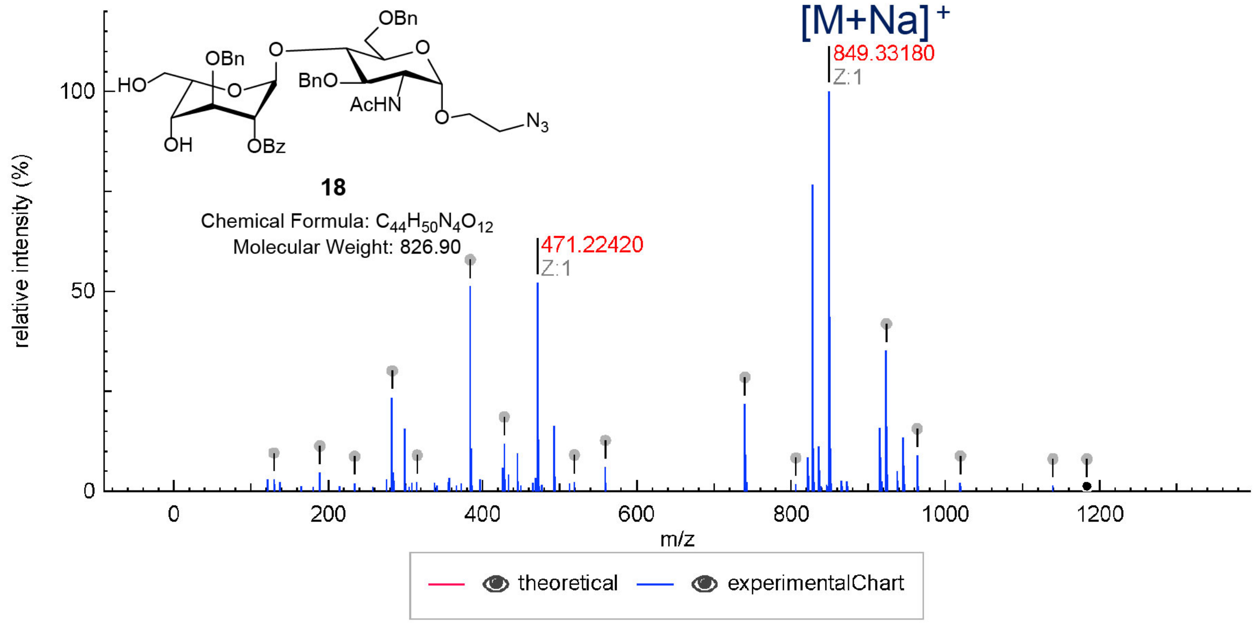

¹H NMR of **20** at 400 MHz, CDCl₃

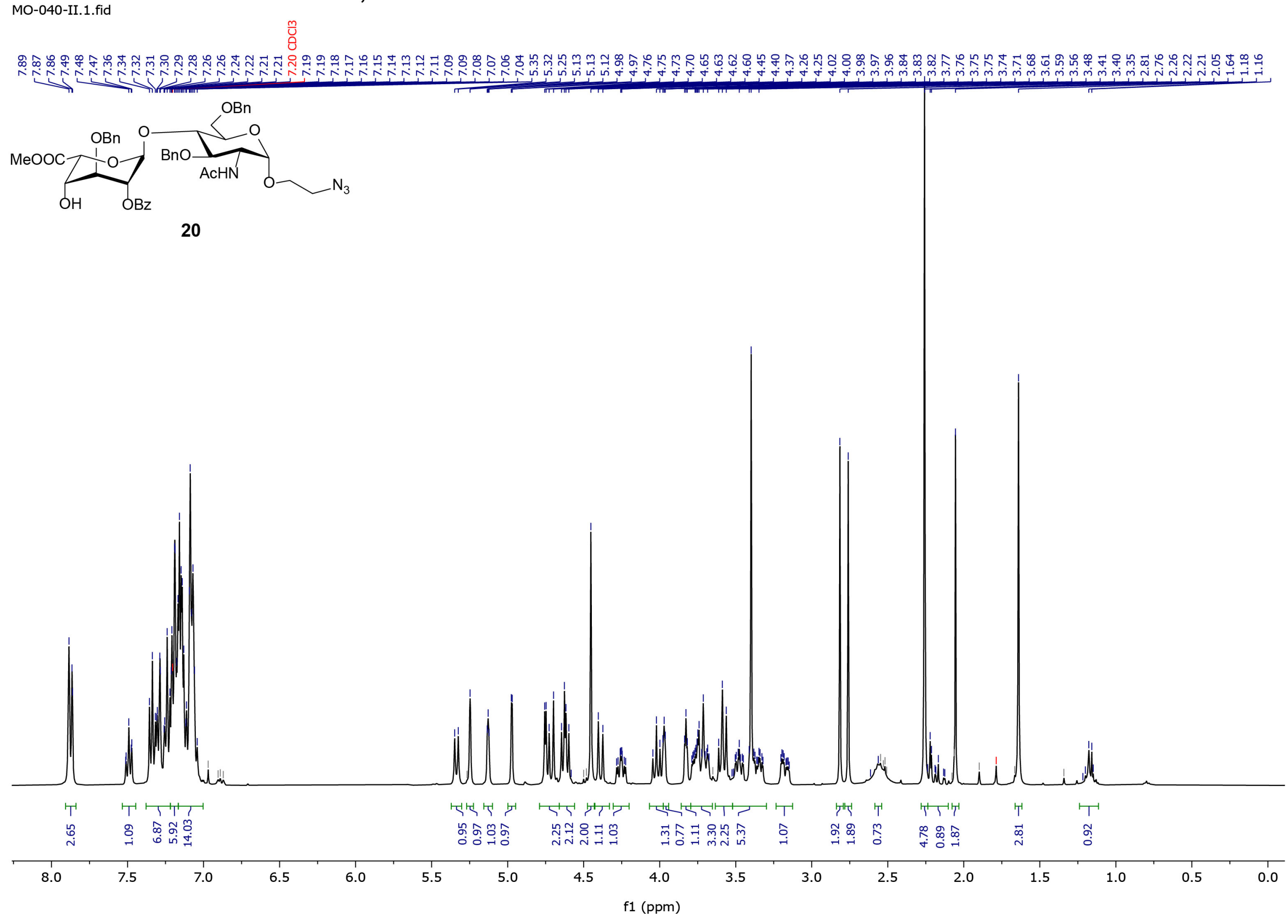

^{13}C NMR of **20** at 101 MHz, $CDCl_3$

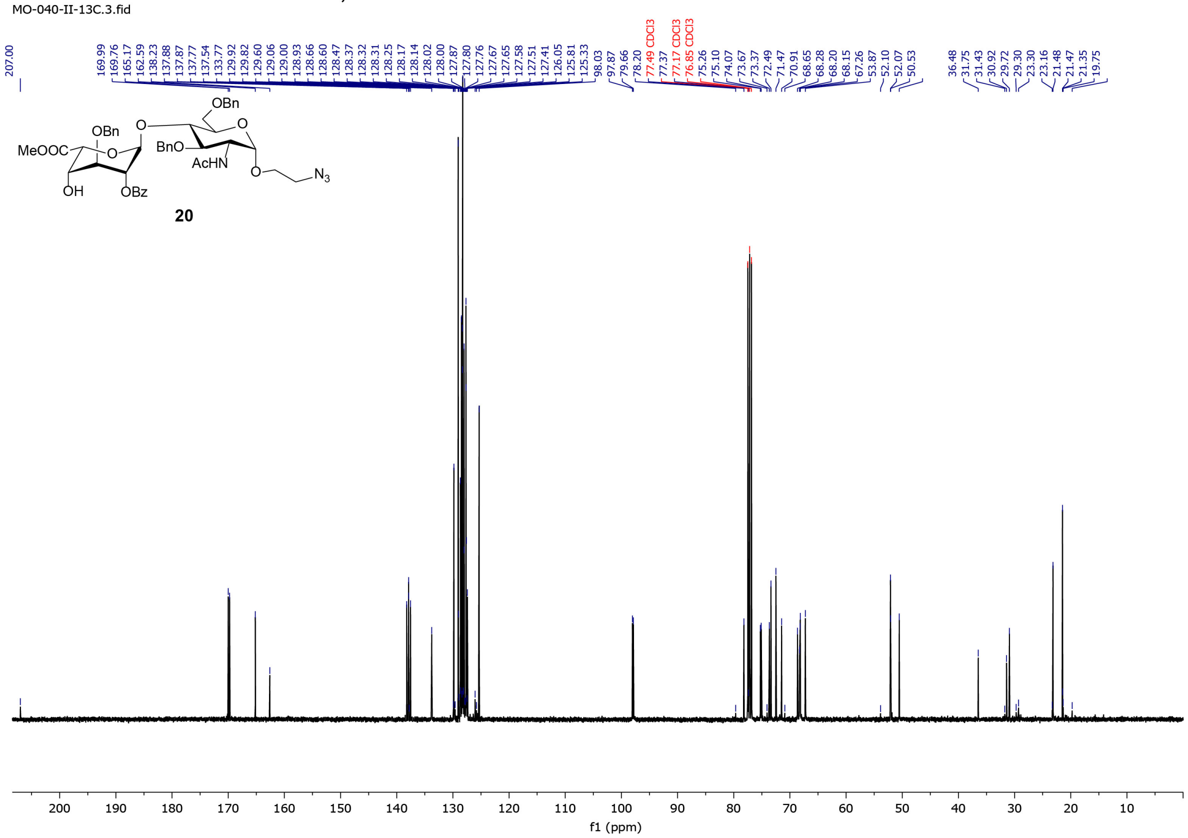

Mass Analysis of **20**

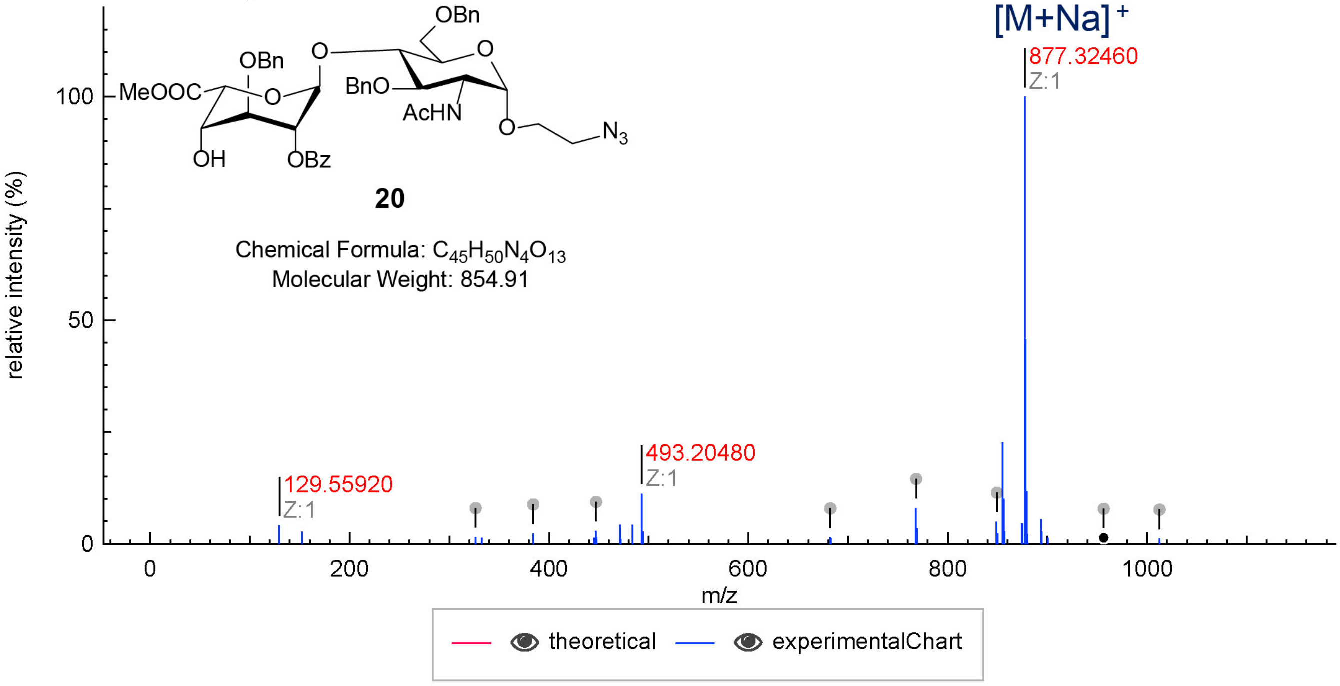

Mass Analysis of **21**

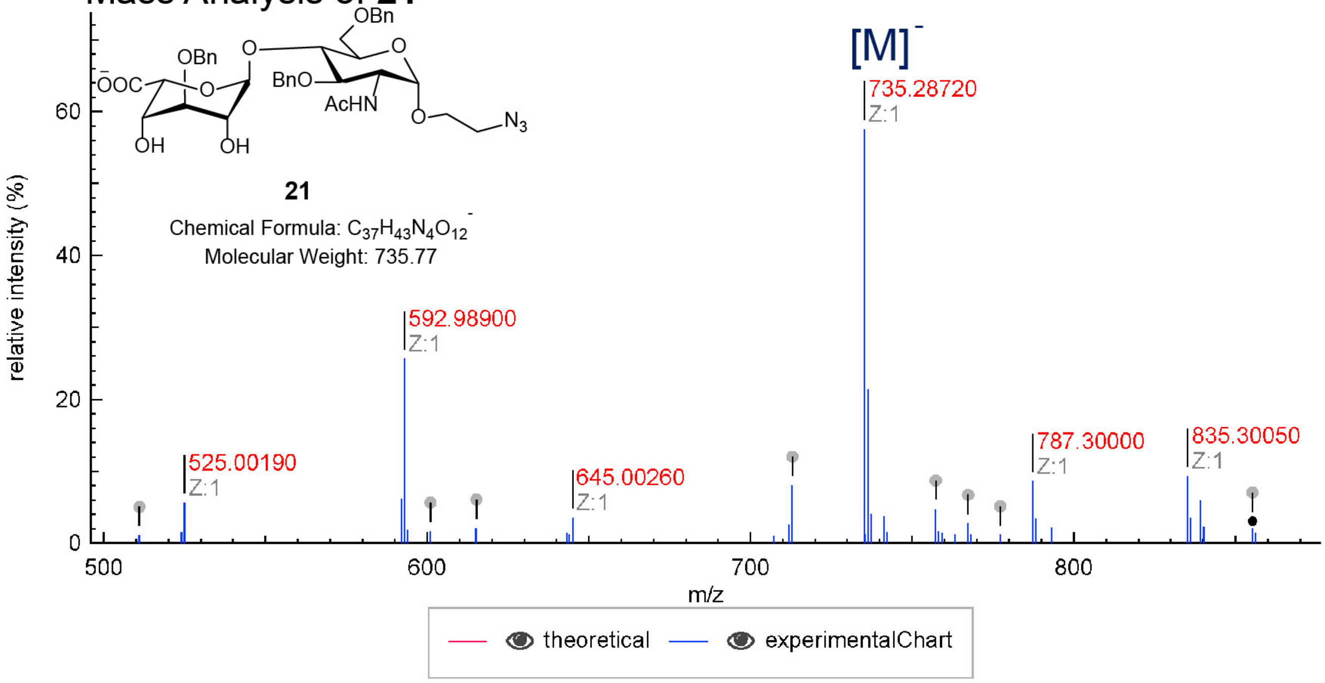

Mass Analysis of **22**

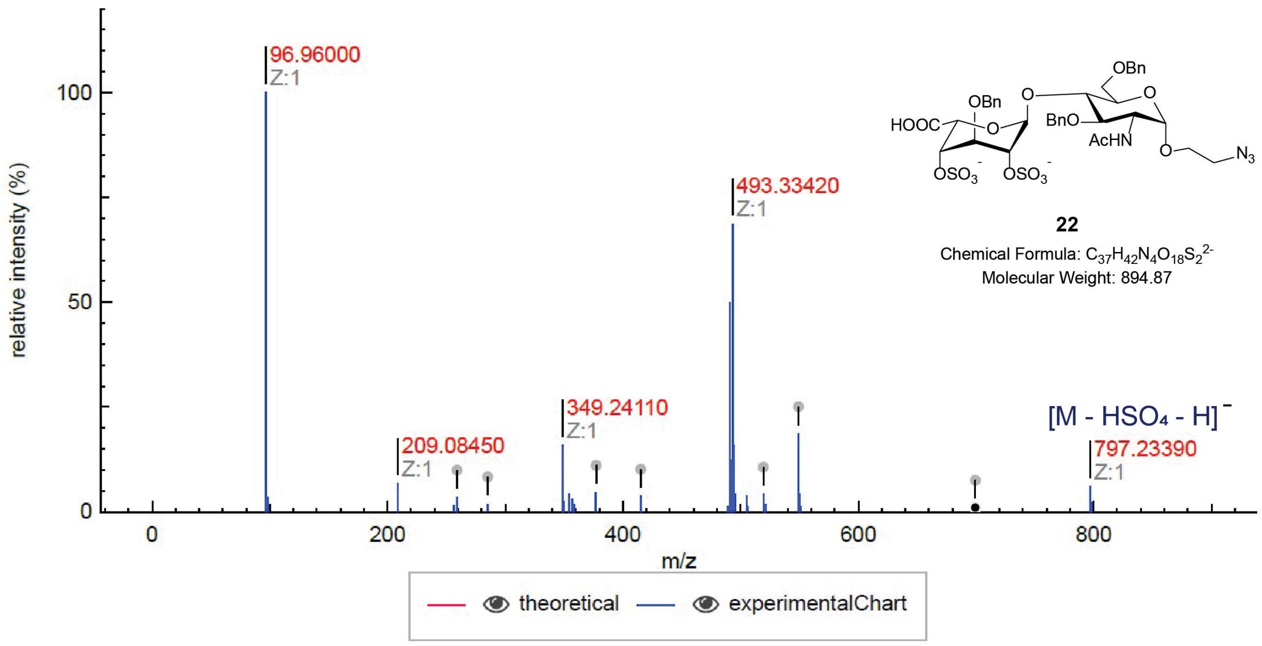

^{1}H NMR of **D1** at 600 MHz, D_2O

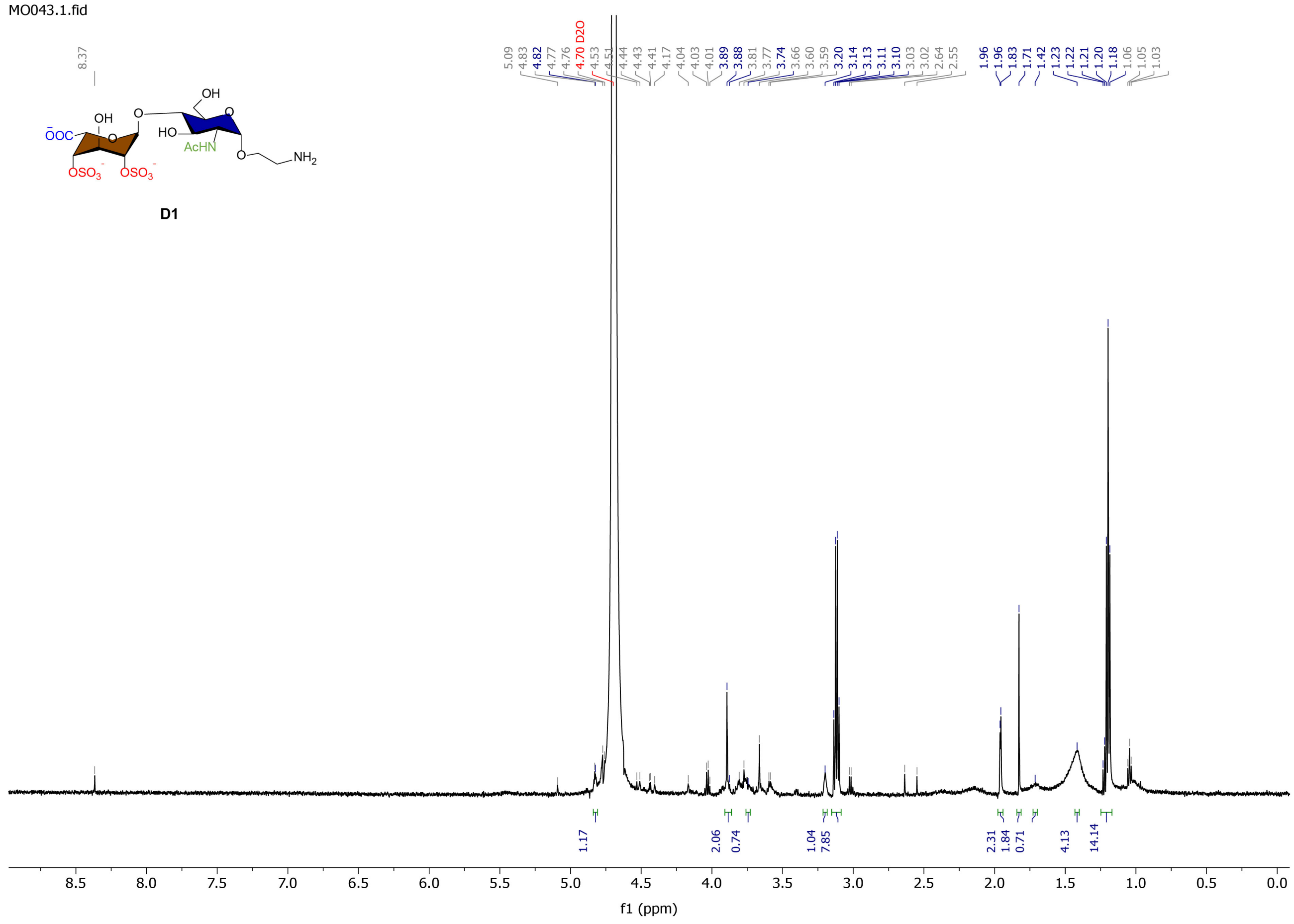

Mass Analysis of **D1**

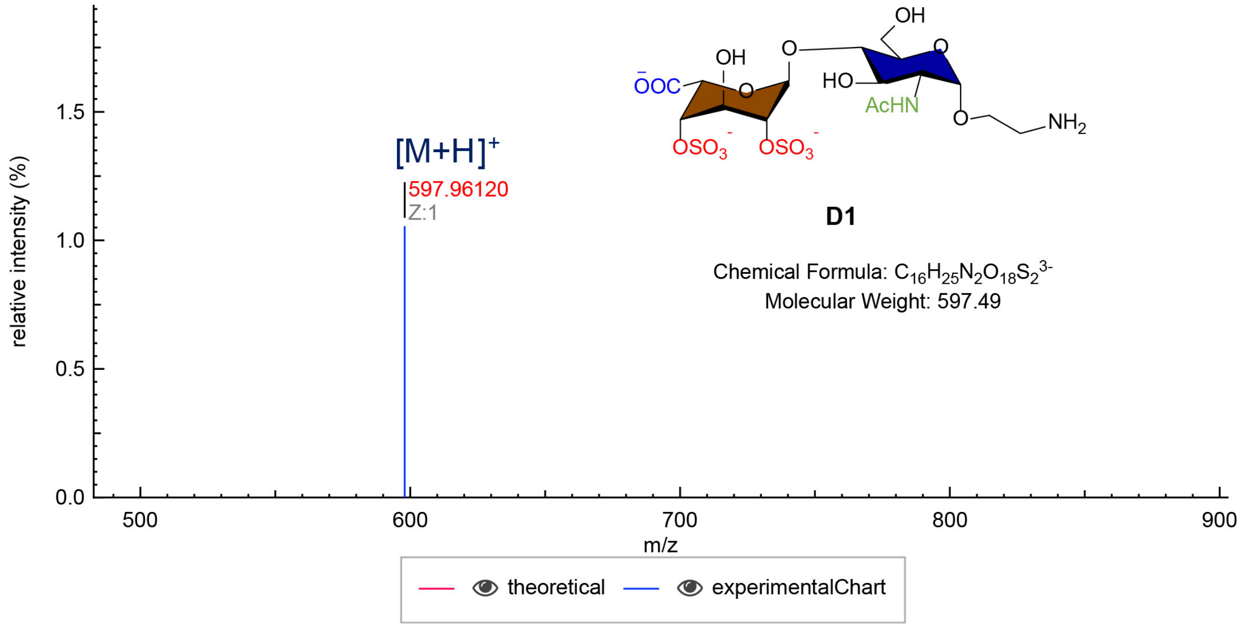

^{1}H NMR of **23** at 400 MHz, $CDCl_3$

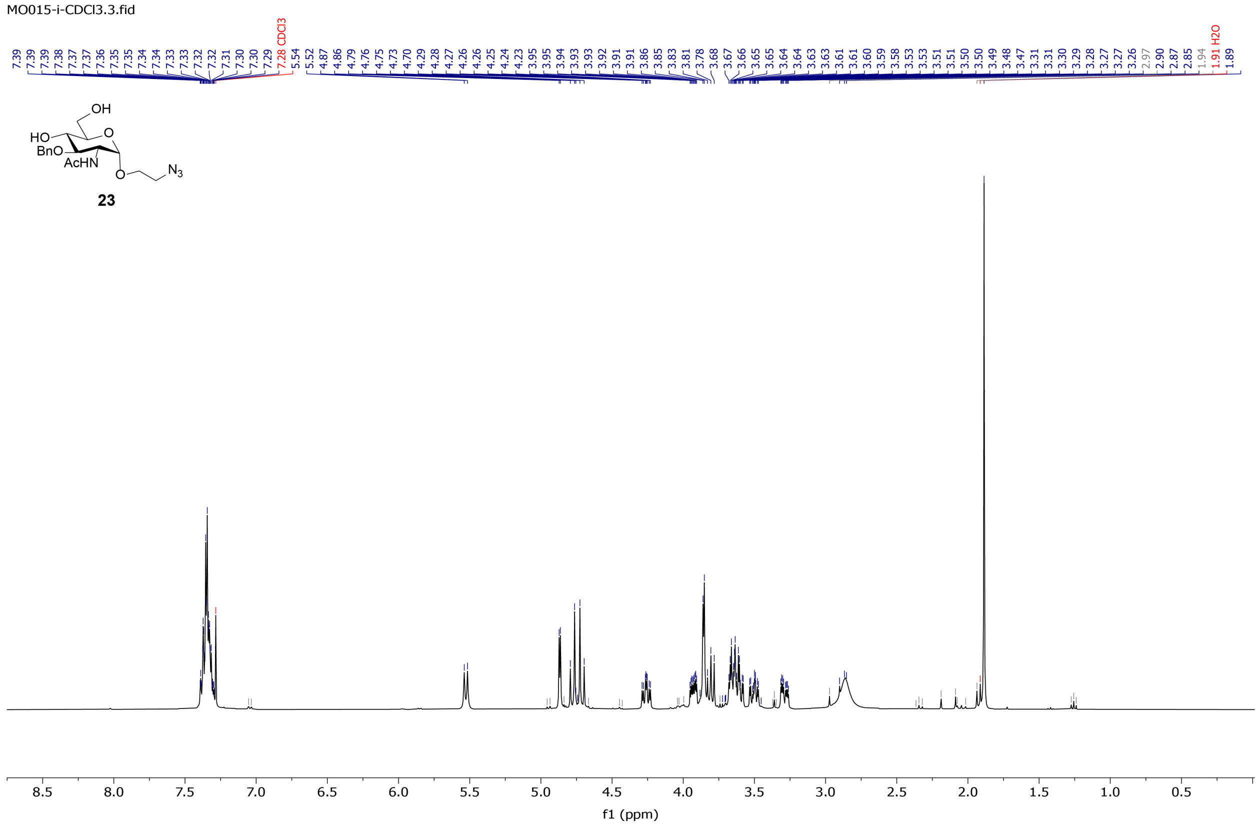

^{13}C NMR of **23** at 101 MHz, $CDCl_3$

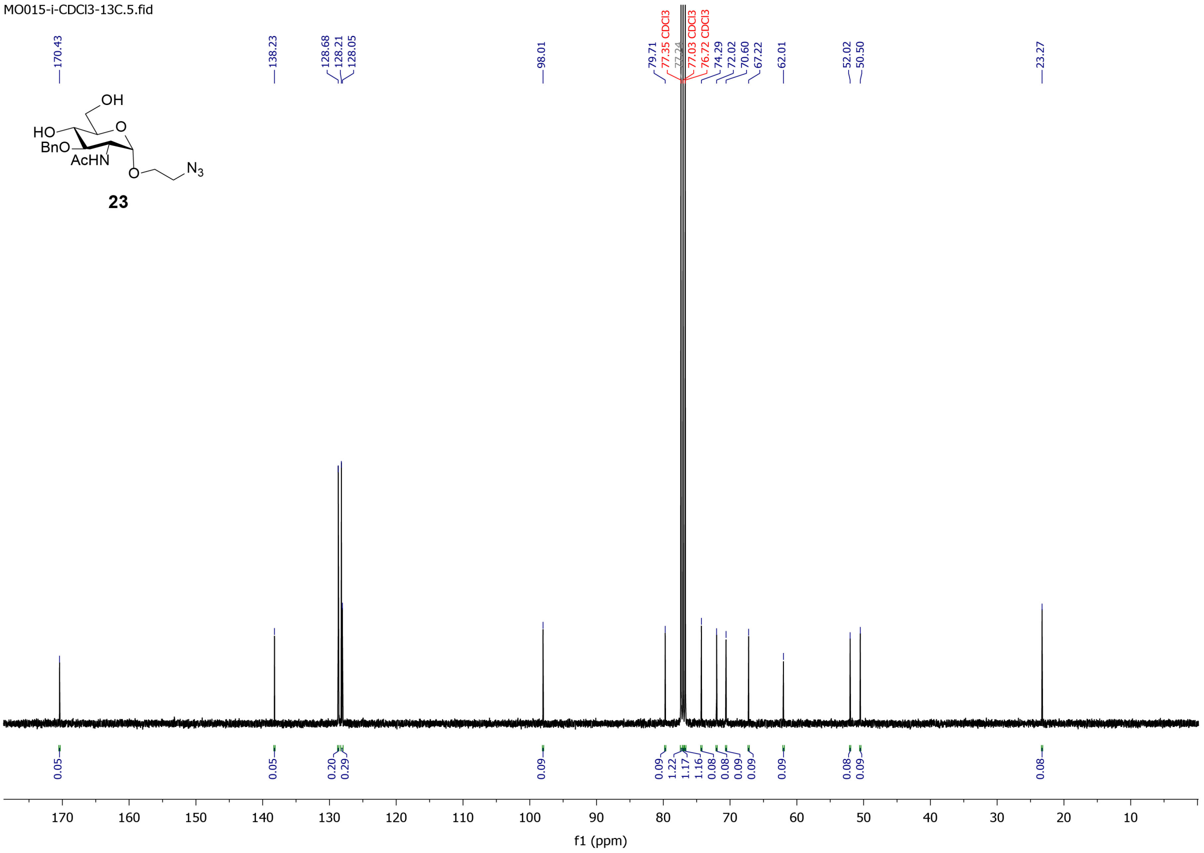

Mass Analysis of **23**

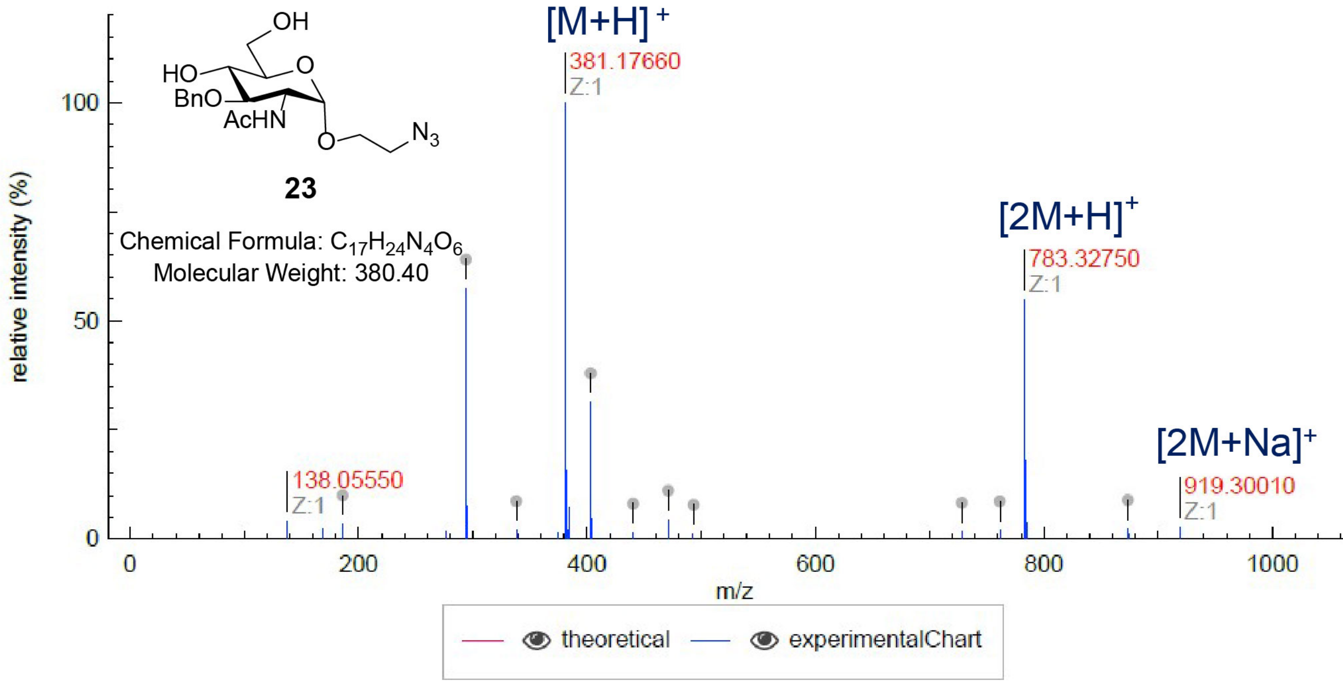

Mass Analysis of **NapCH(OMe)₂**

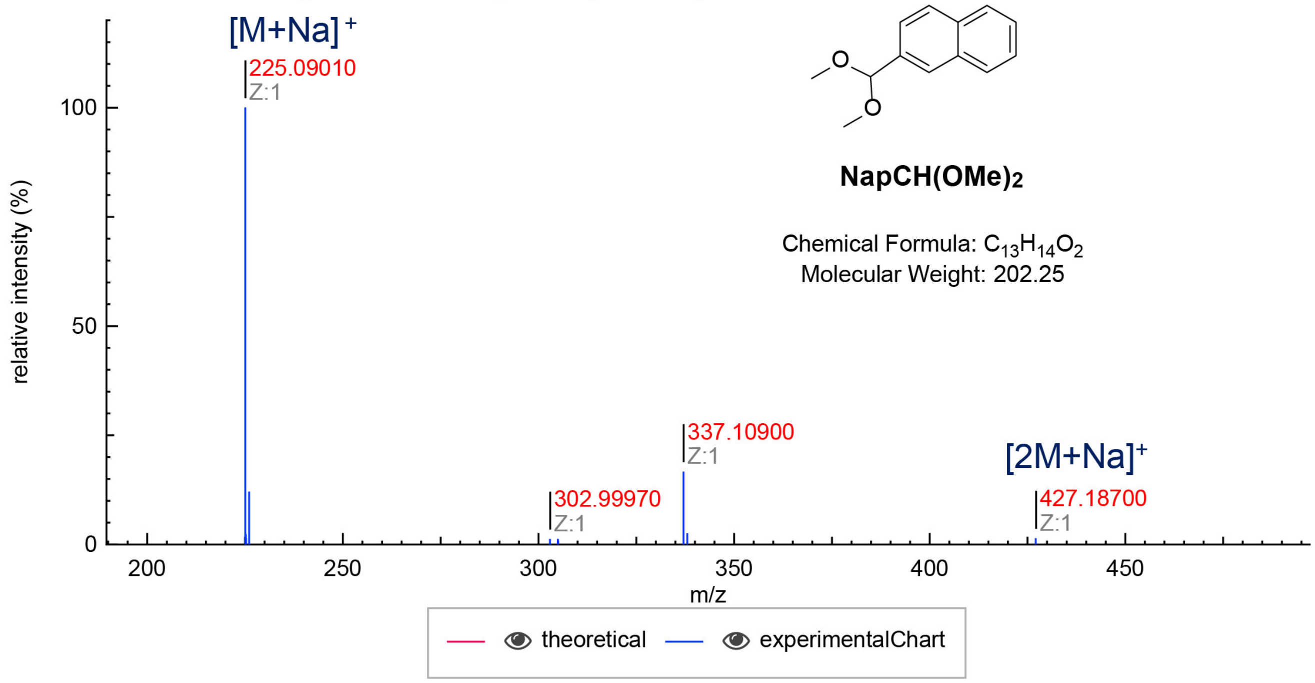

^{1}H NMR of **24** at 400 MHz, $CDCl_3$

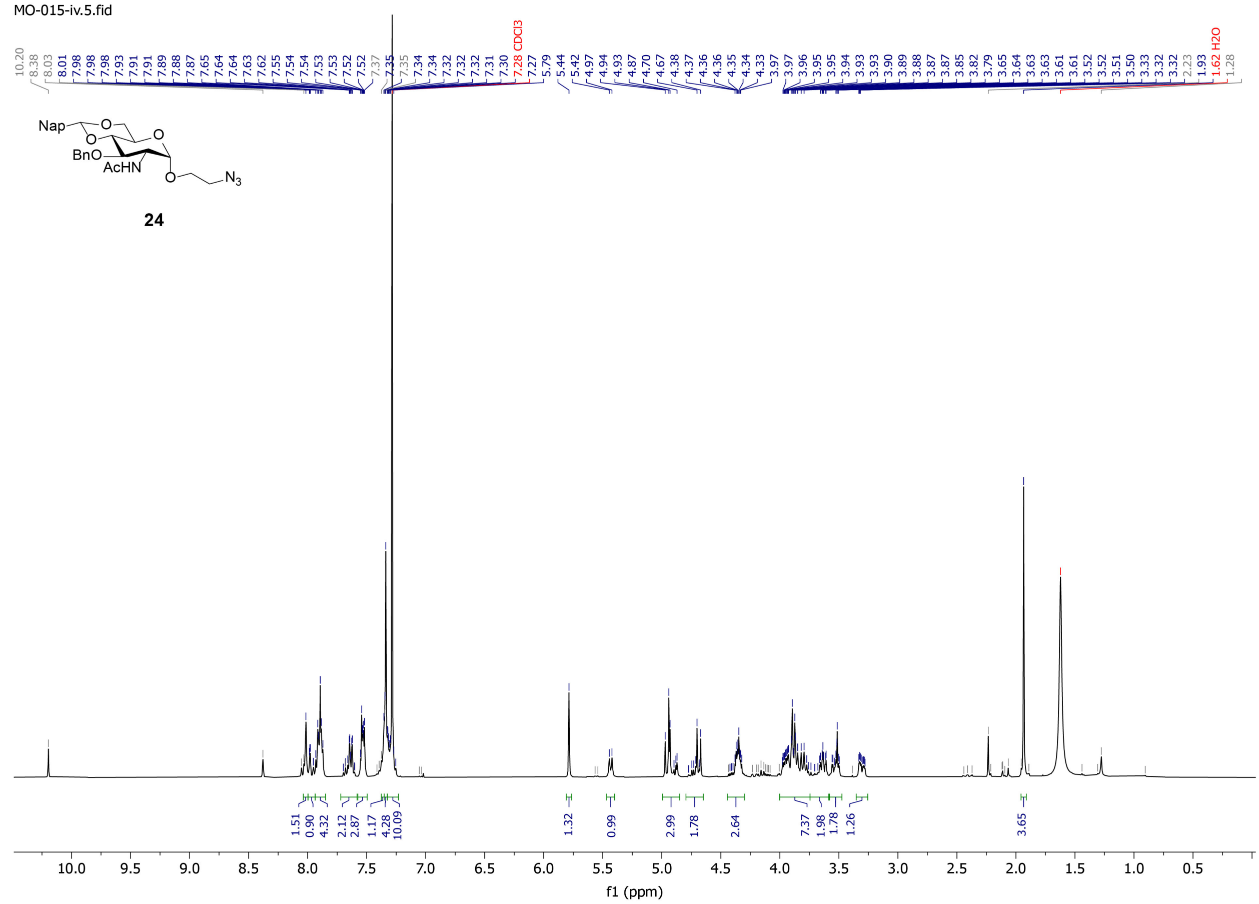

^{13}C NMR of **24** at 101 MHz, $CDCl_3$

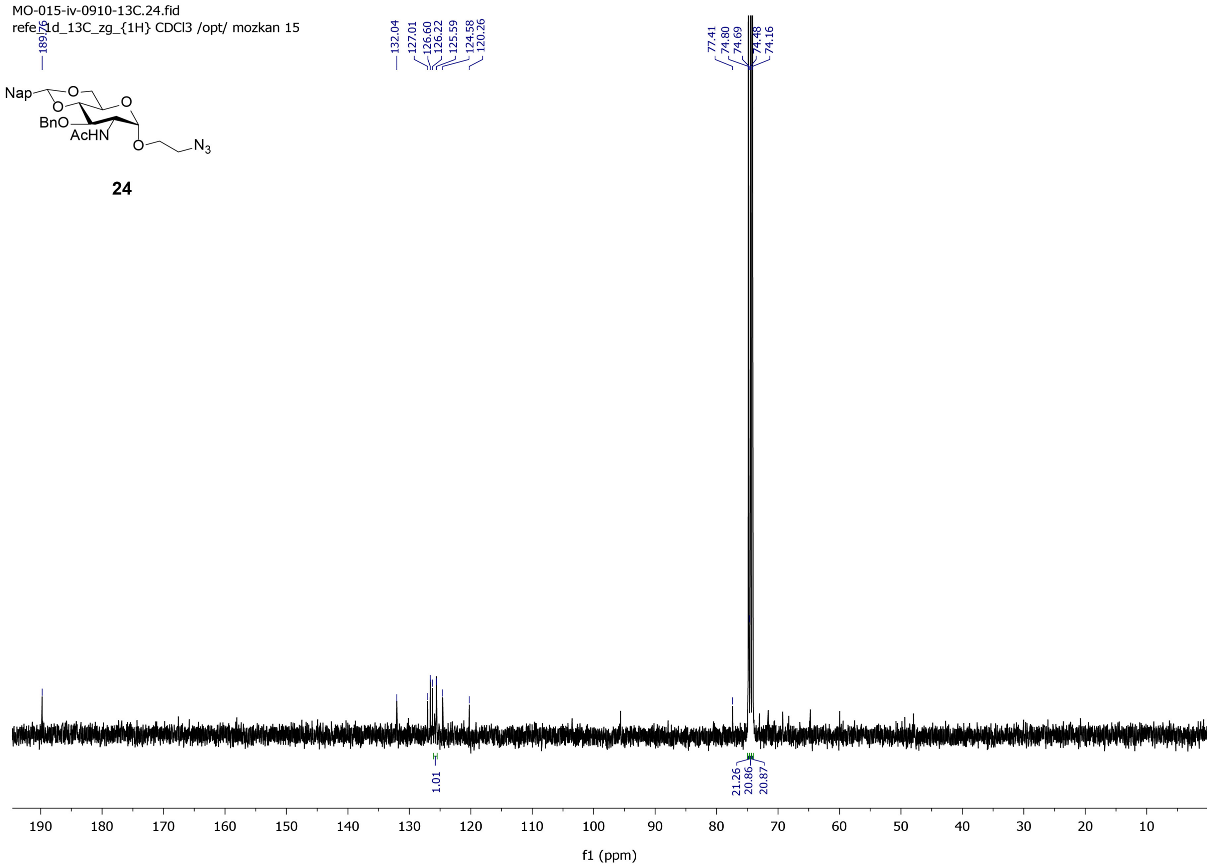

Mass Analysis of **24**

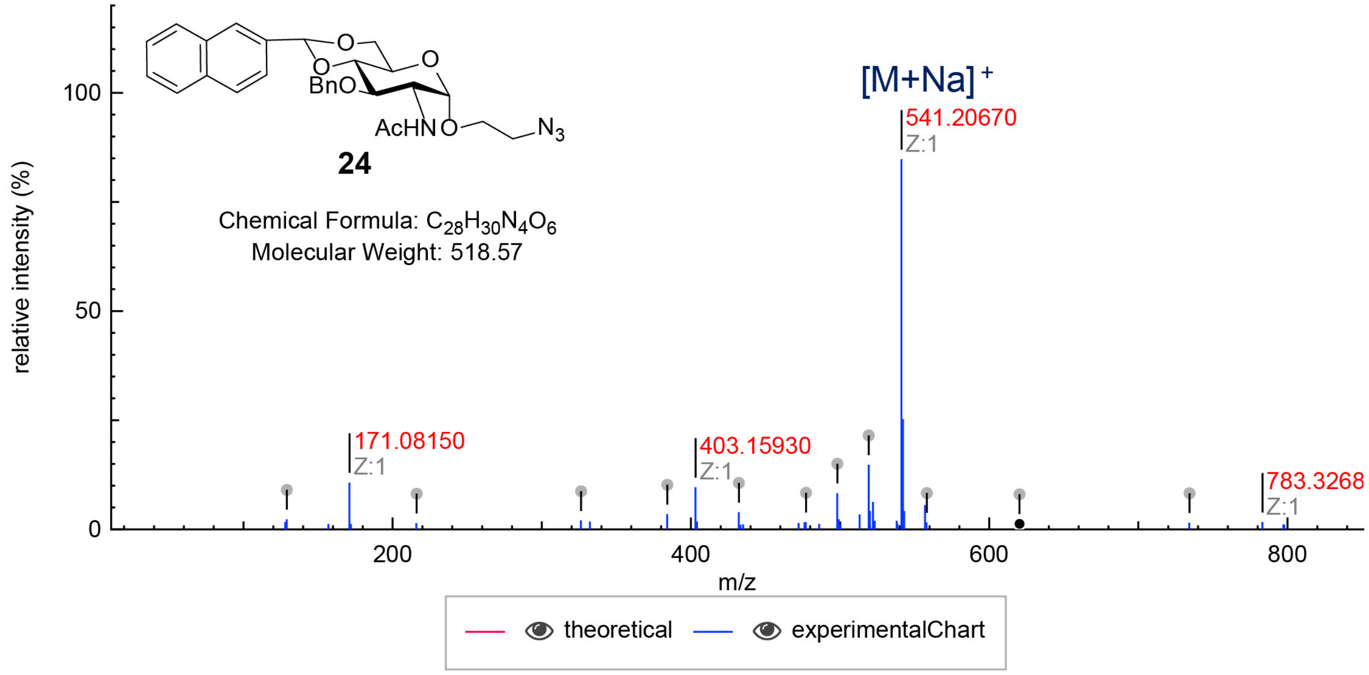

¹H NMR of **25** at 400 MHz, $CDCl_3$

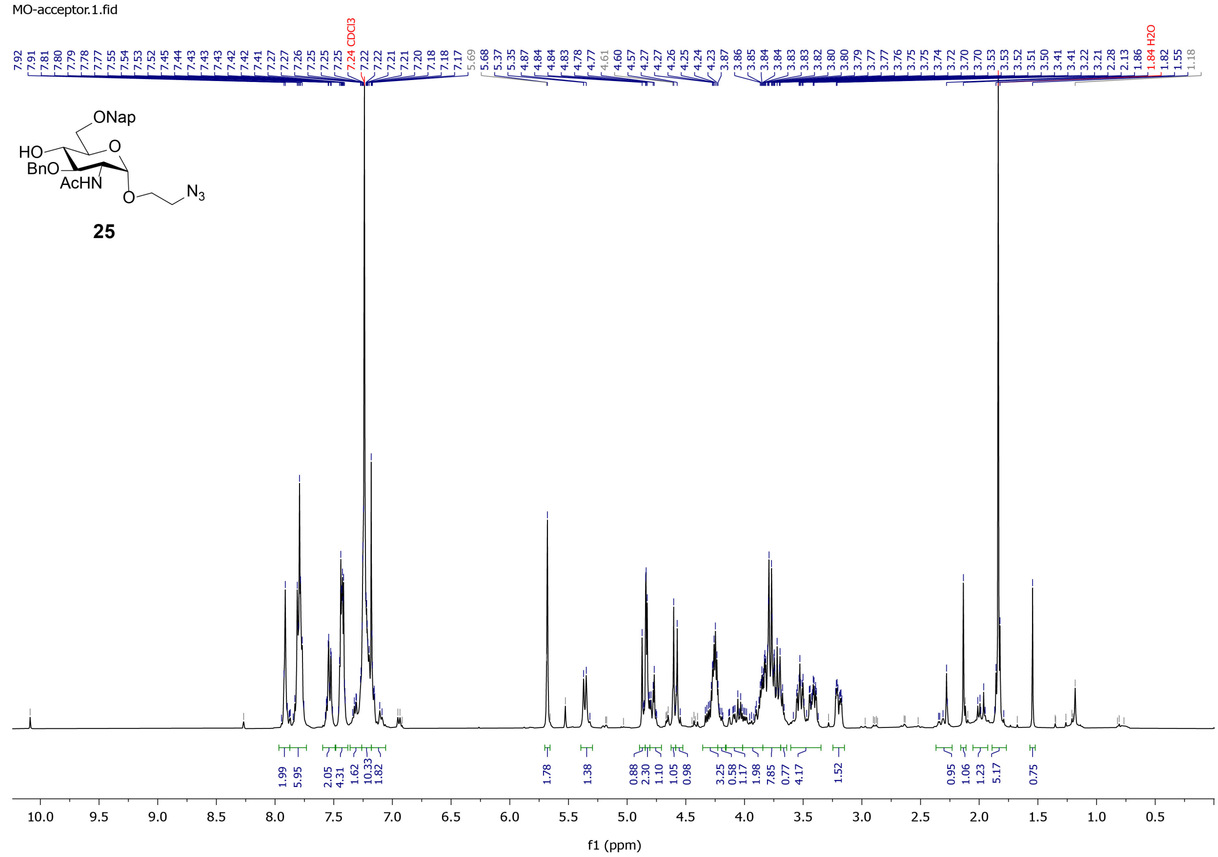

¹³C NMR of **25** at 101 MHz, $CDCl_3$

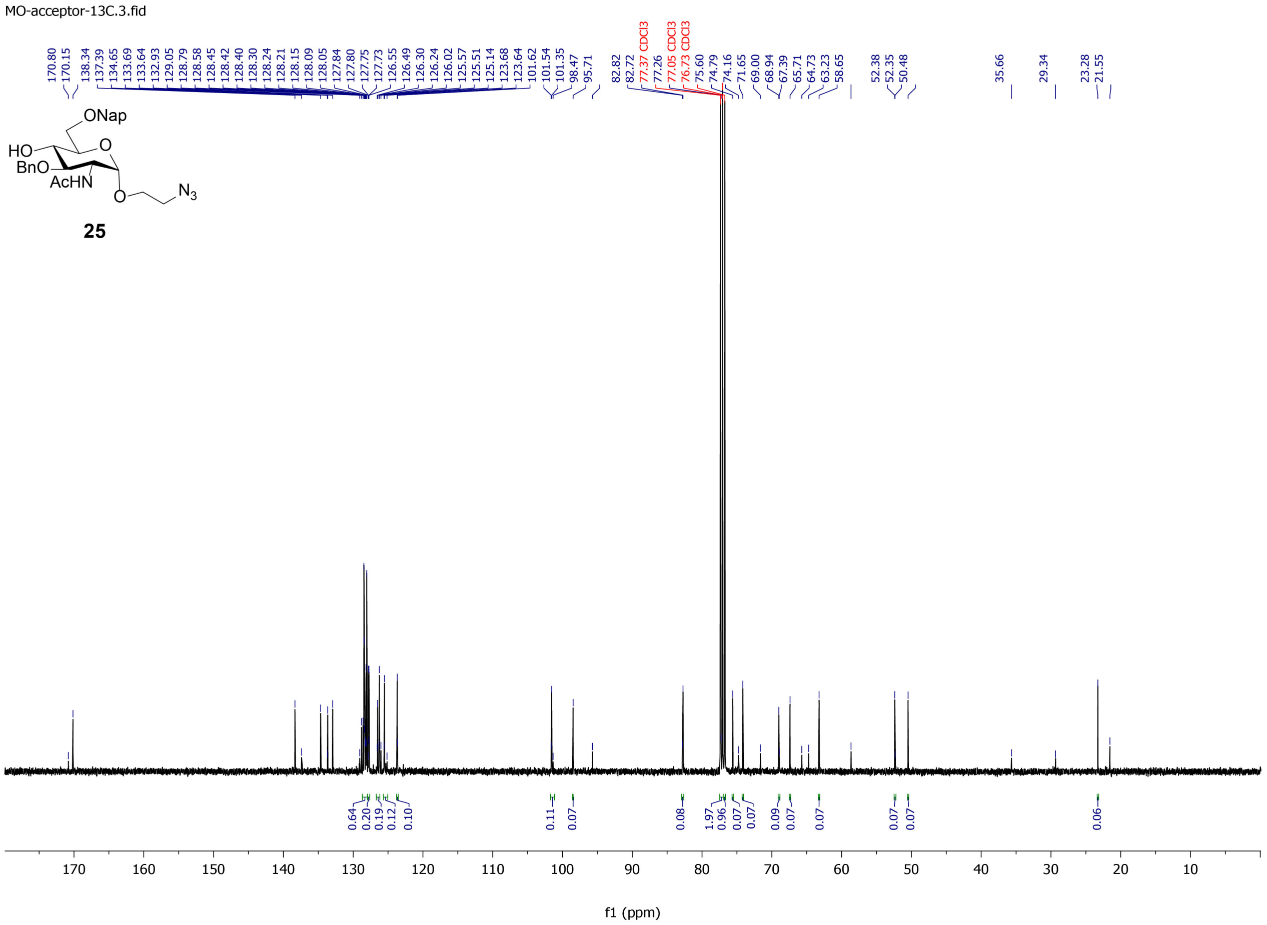

Mass Analysis of **25**

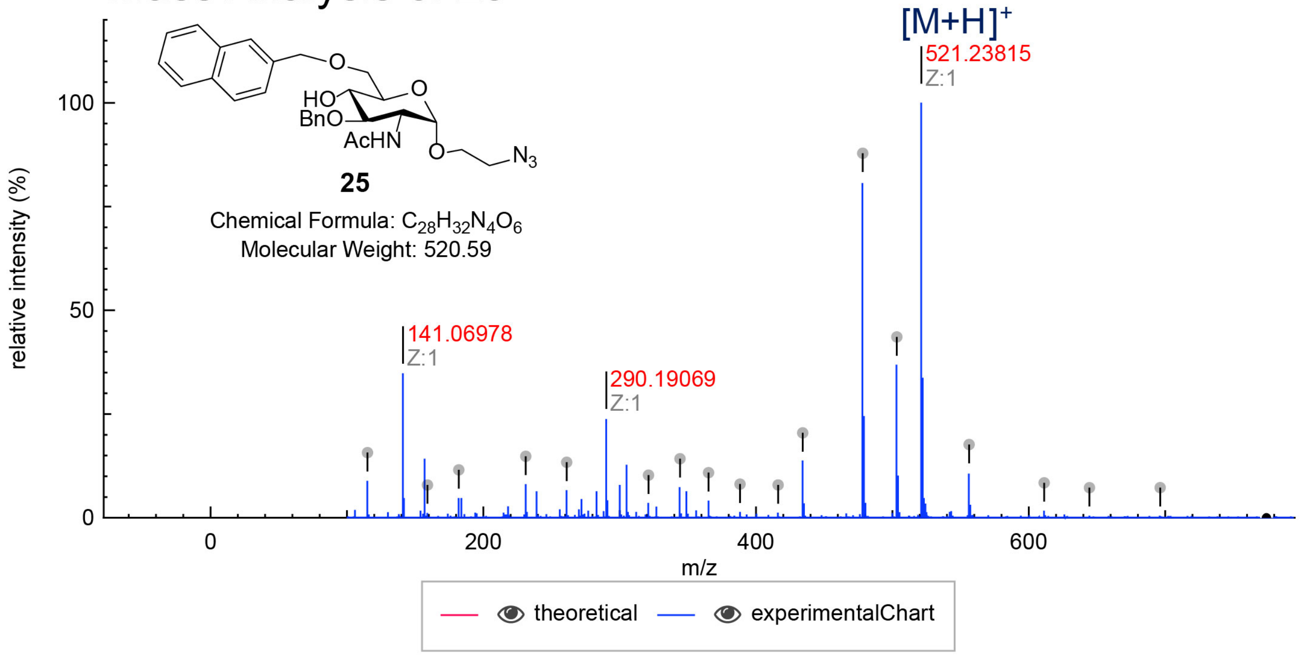

^{1}H NMR of **26** at 400 MHz, $CDCl_3$

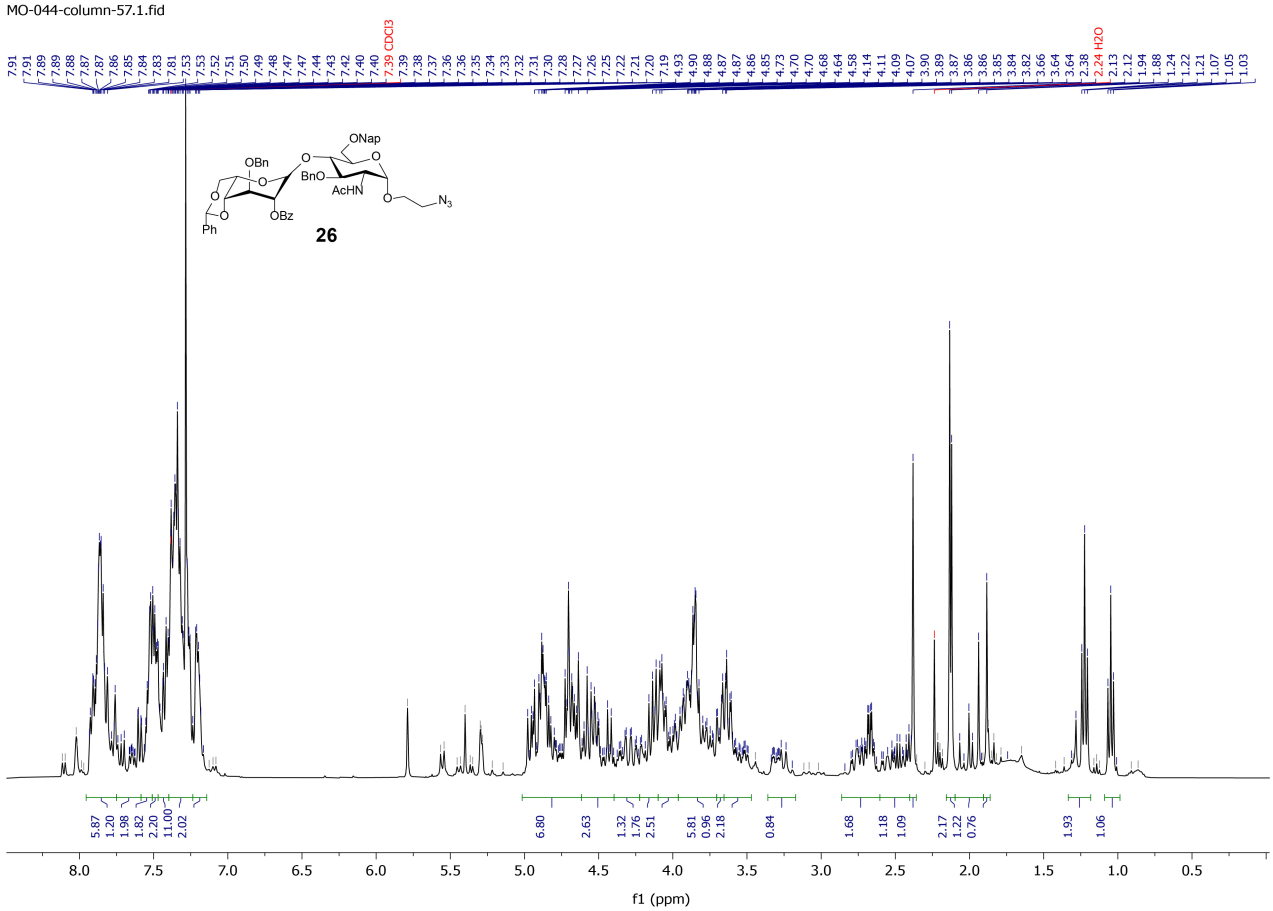

^{13}C NMR of **26** at 101 MHz, $CDCl_3$

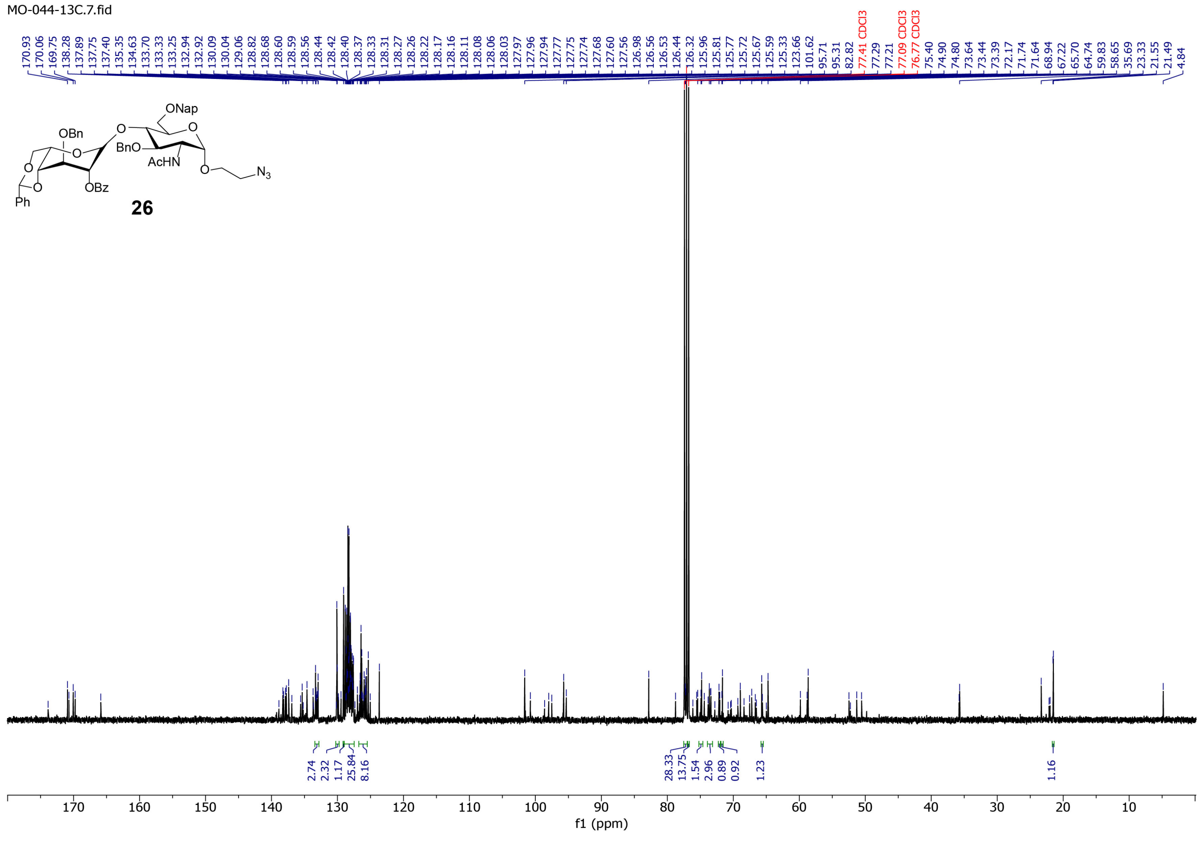

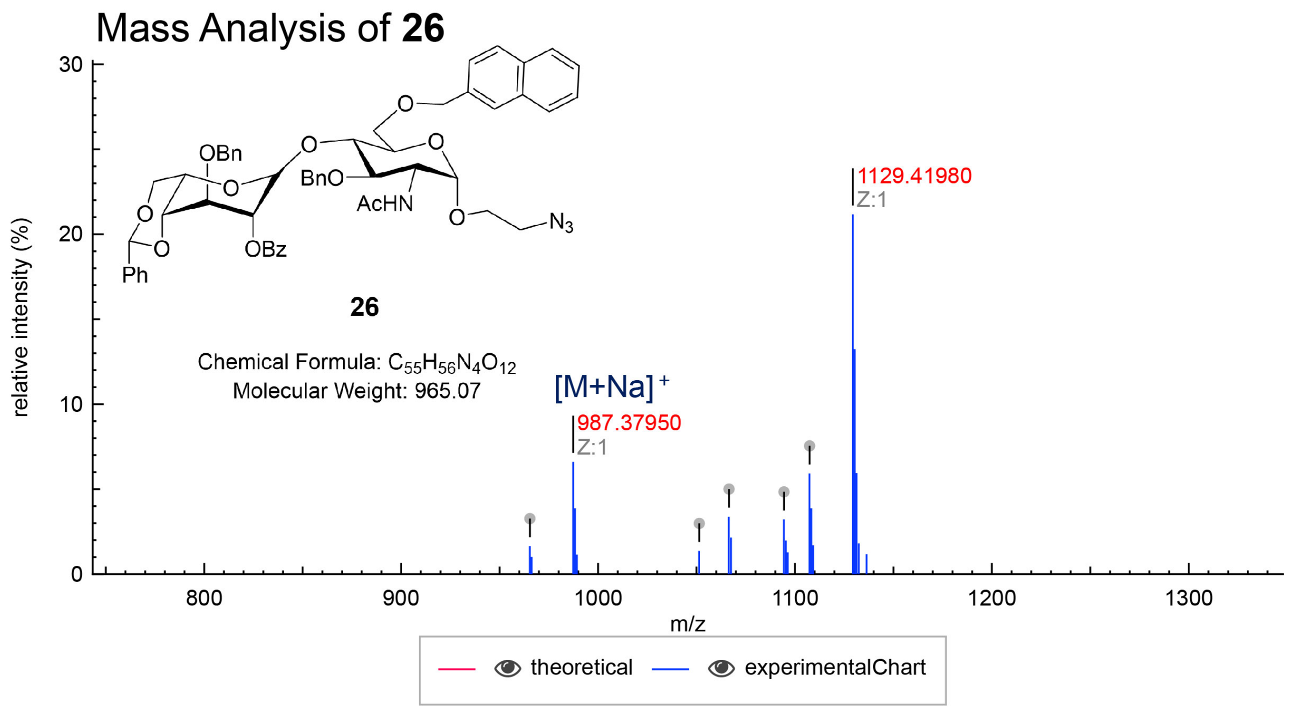
Mass Analysis of 26
OBn
BnO
AcHN
OBz
Ph
N3
26
Chemical Formula: C55H56N4O12
Molecular Weight: 965.07
[M+Na]+
987.37950
Z:1
1129.41980
Z:1
relative intensity (%)
m/z
theoretical
experimentalChart

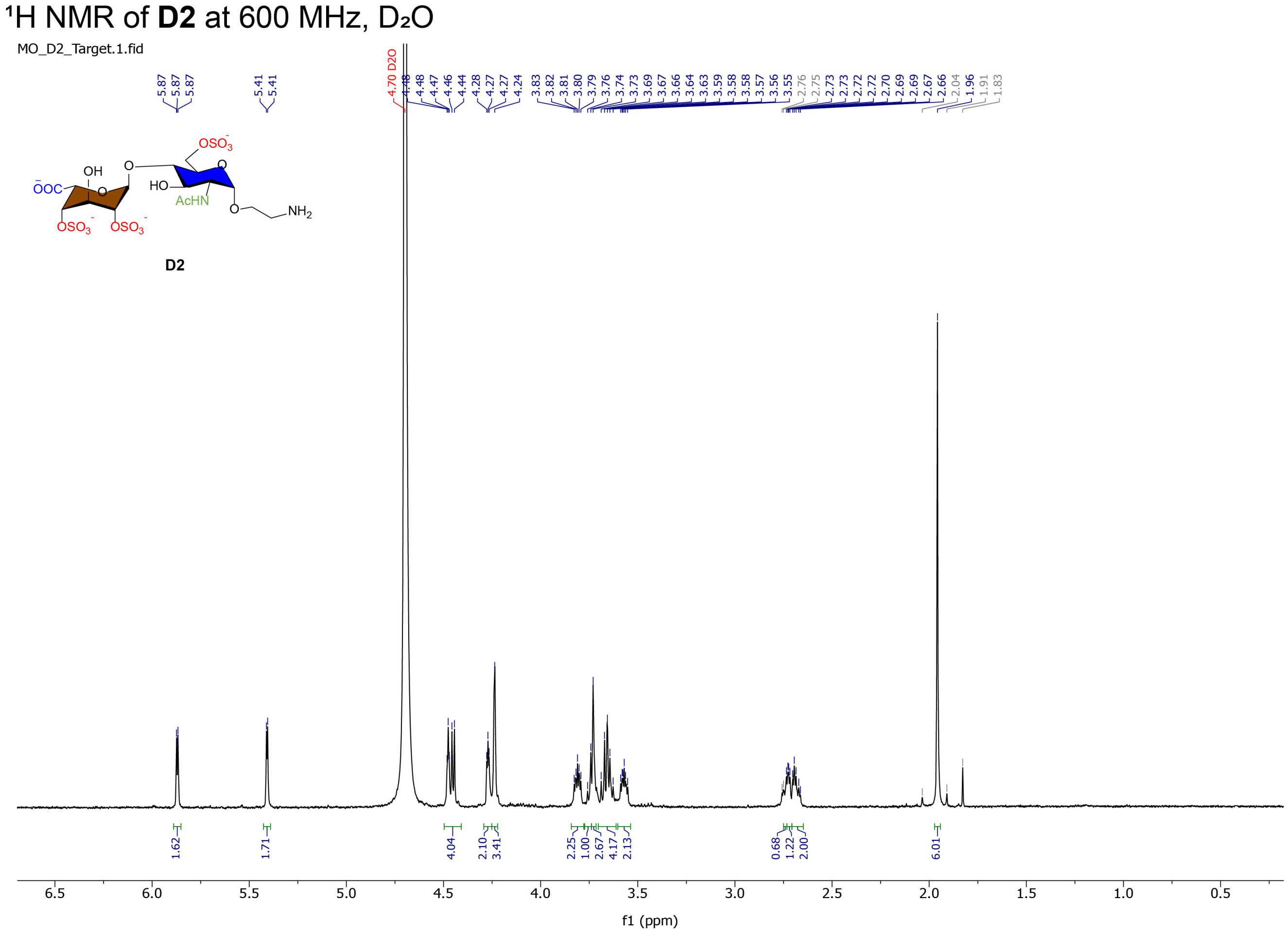
1H NMR of D2 at 600 MHz, D2O
MO_D2_Target.1.fid
D2
AcHN
NH2
f1 (ppm)

¹³C NMR of **D2** at 151 MHz, D_2O

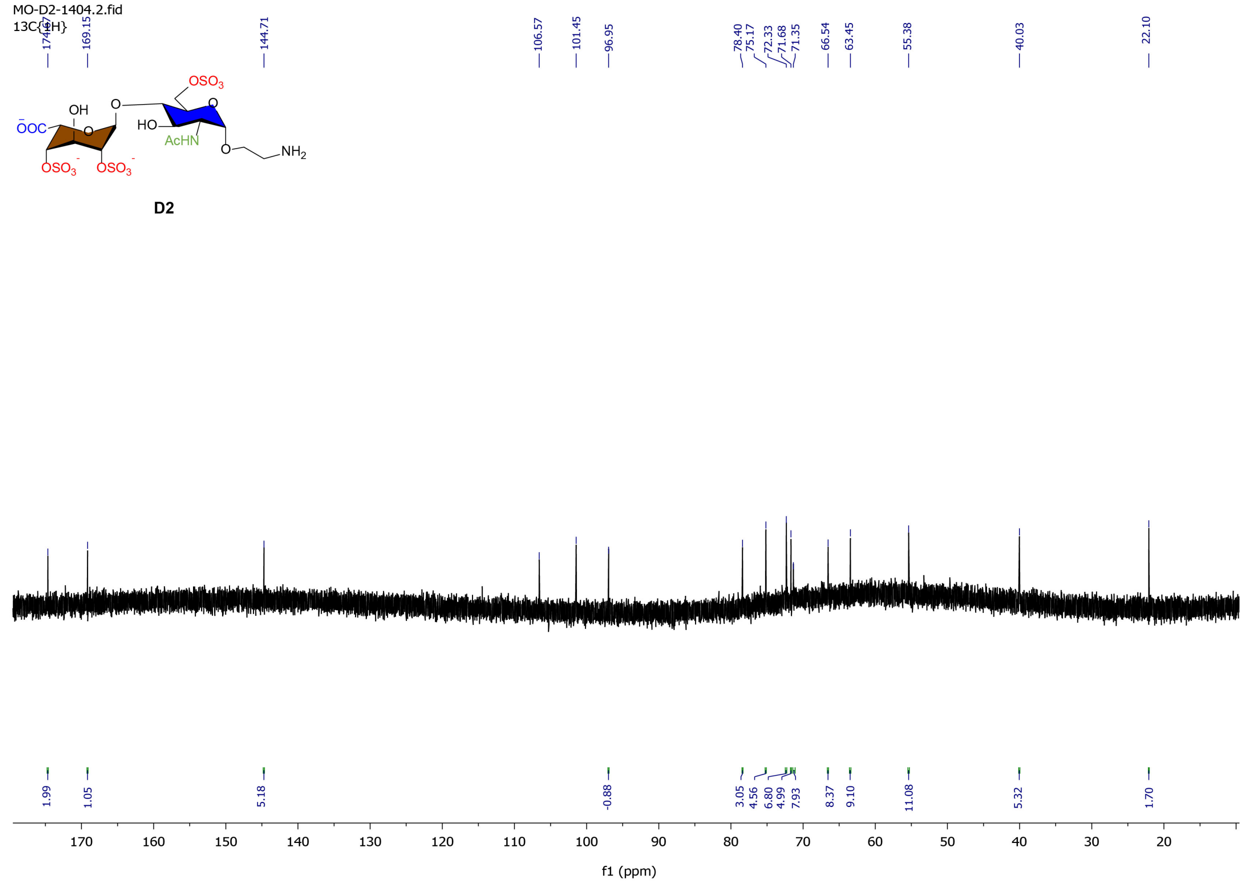

¹H-¹H COSY NMR of **D2** at 600 MHz, D_2O

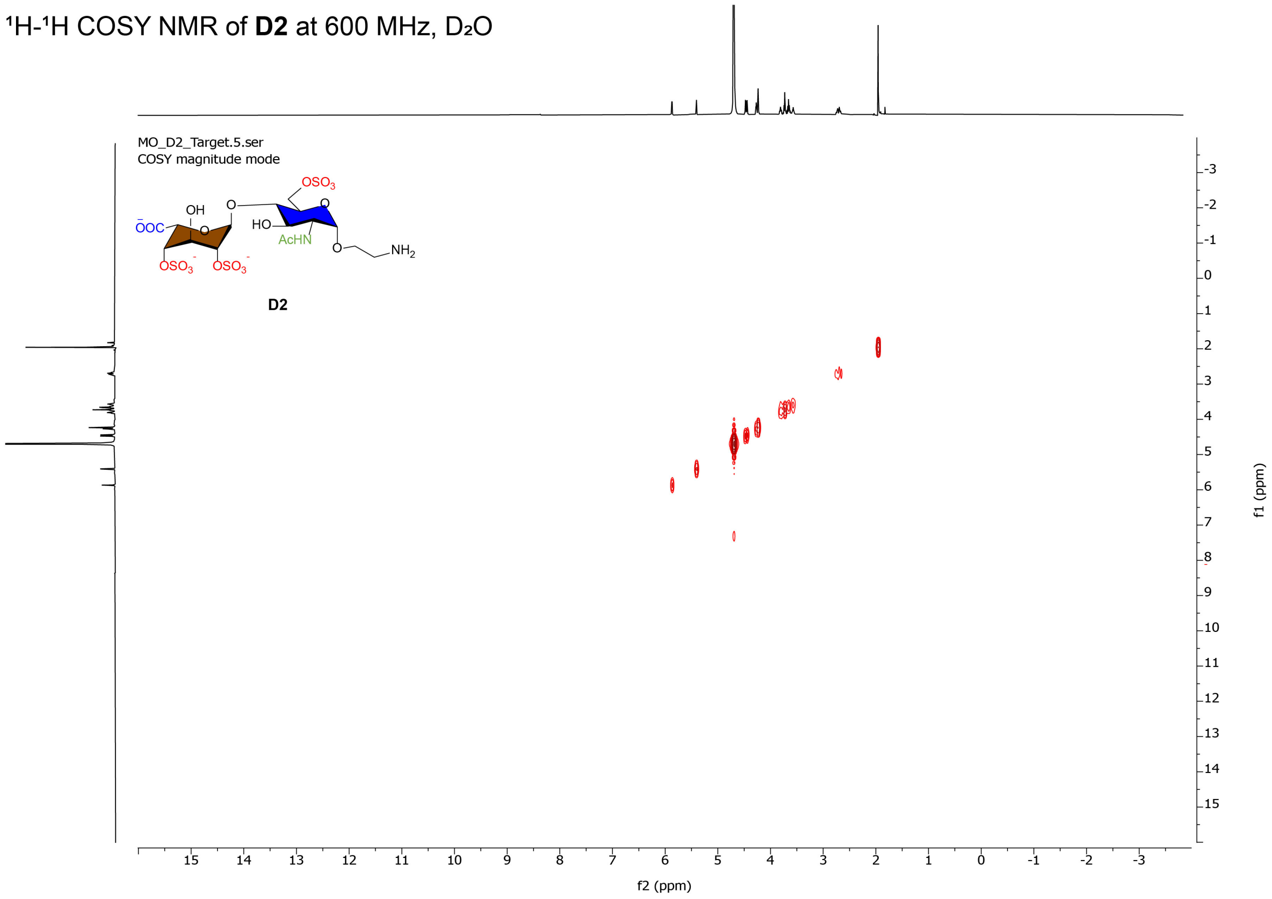

^1H-^{13}C HSQC NMR of **D2**, D_2O

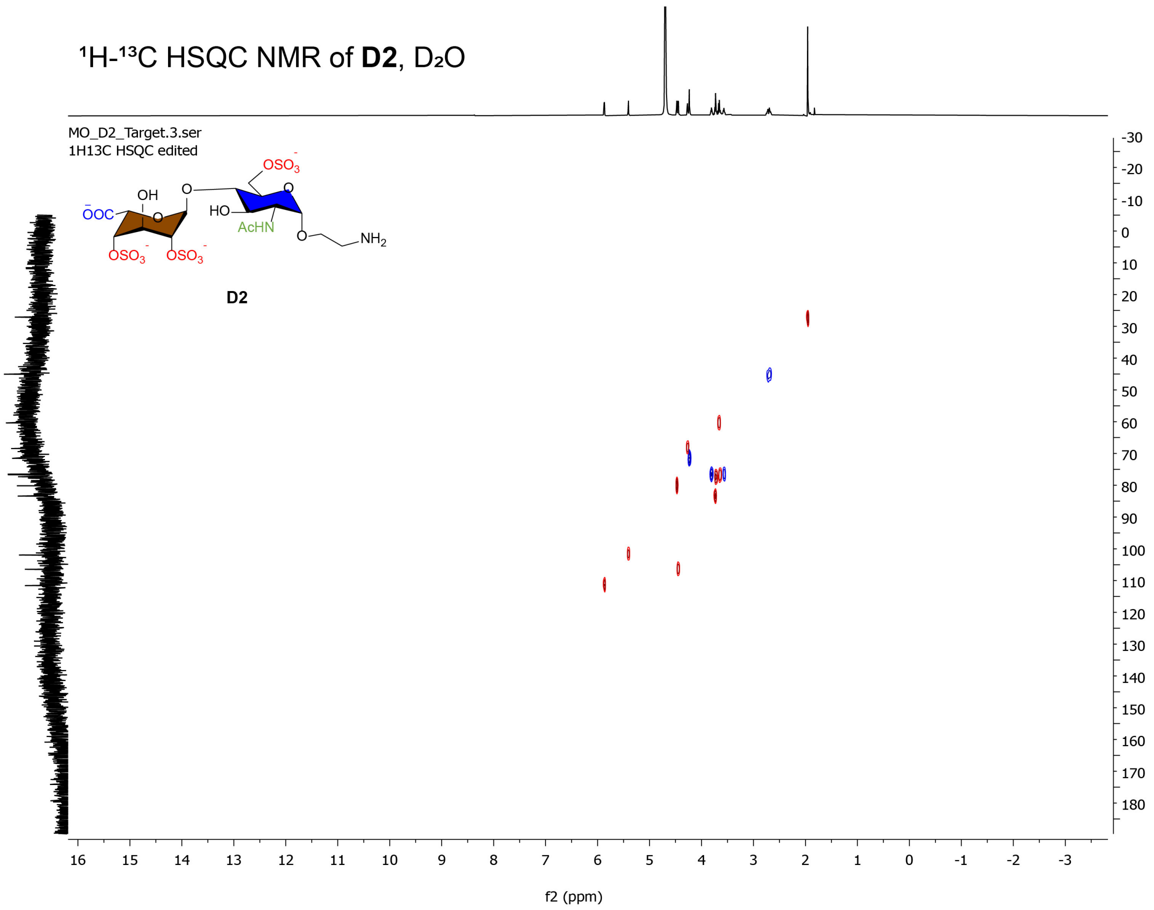

^1H NMR of **34** at 400 MHz, $CDCl_3$

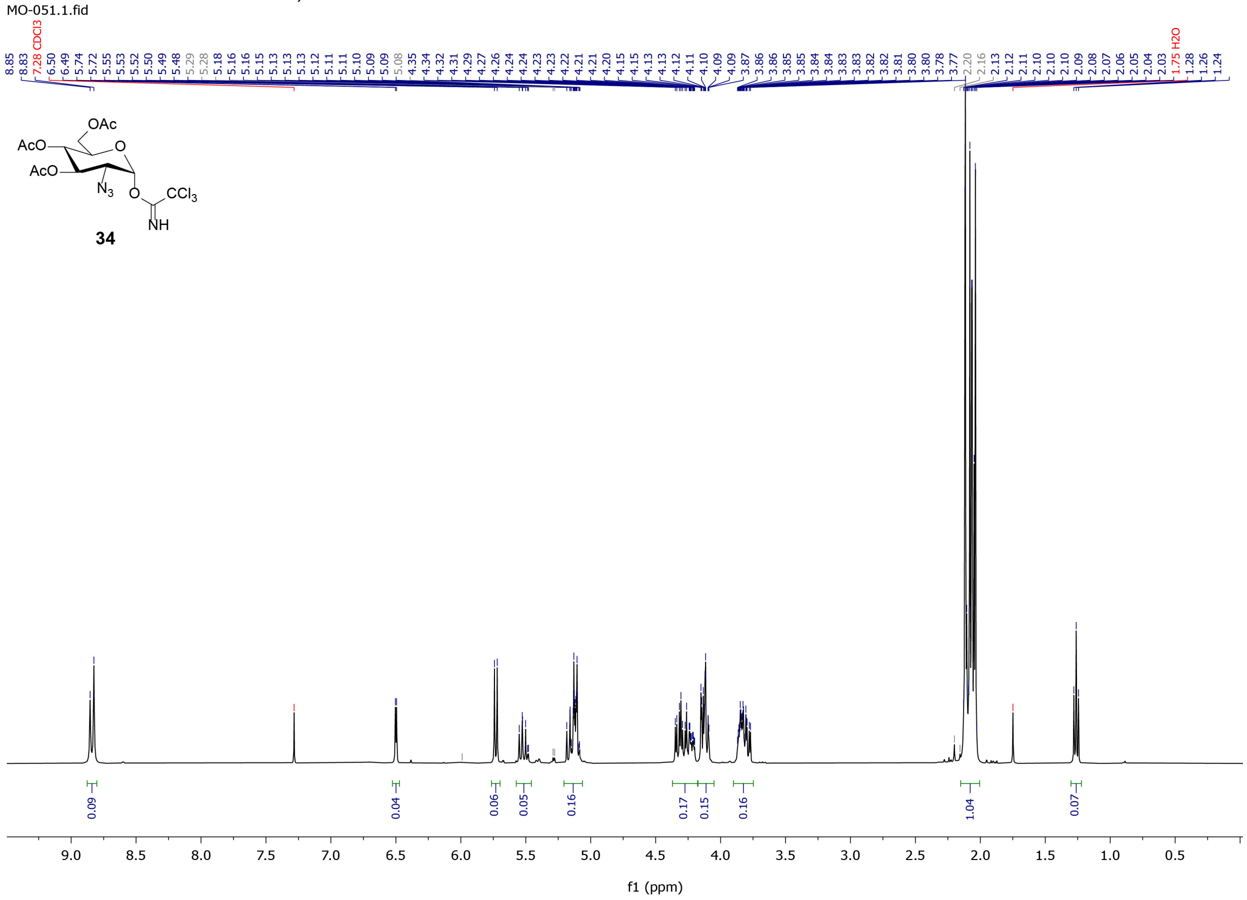

^{13}C NMR of **34** at 101 MHz, $CDCl_3$

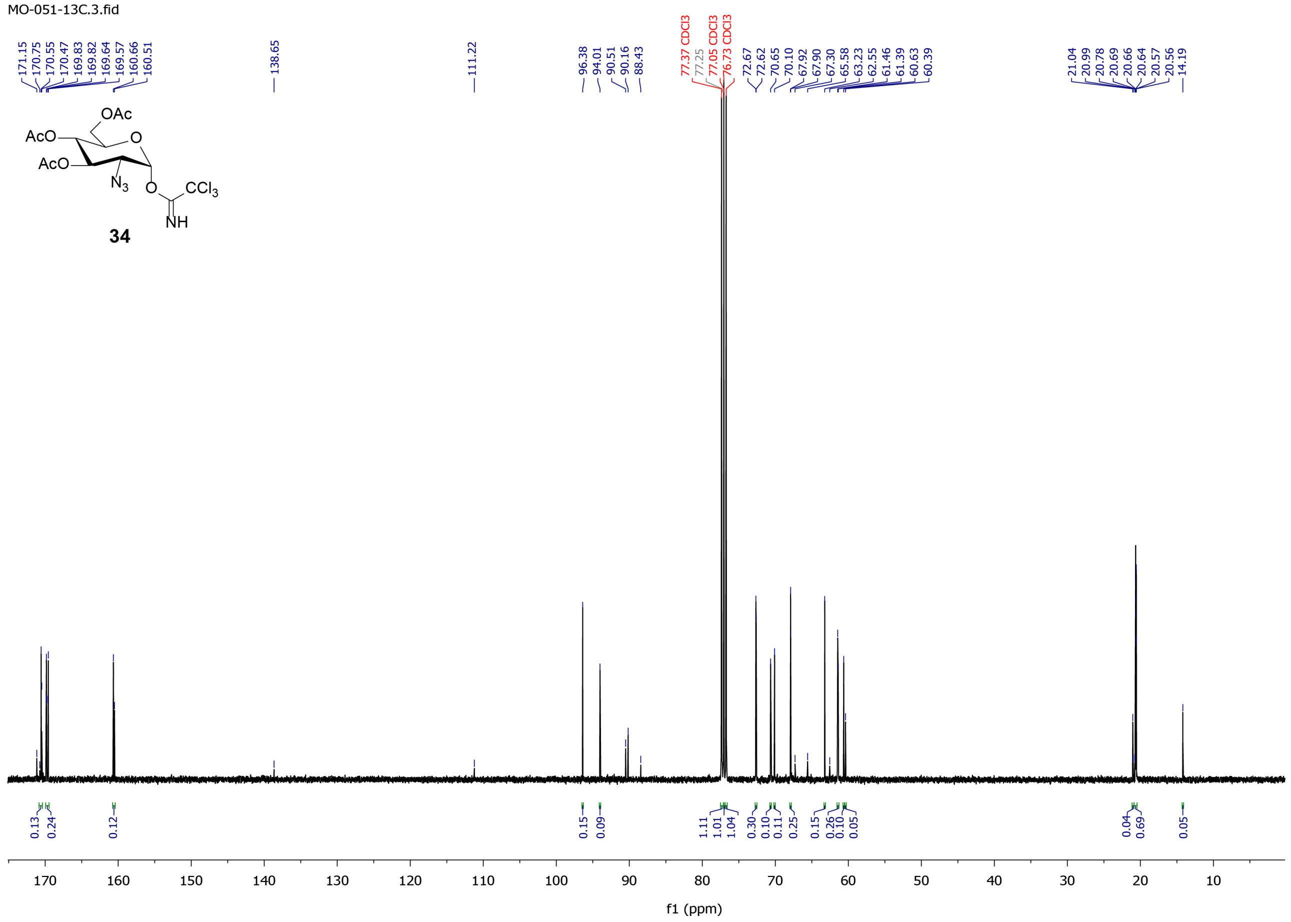

Mass Analysis of **34**

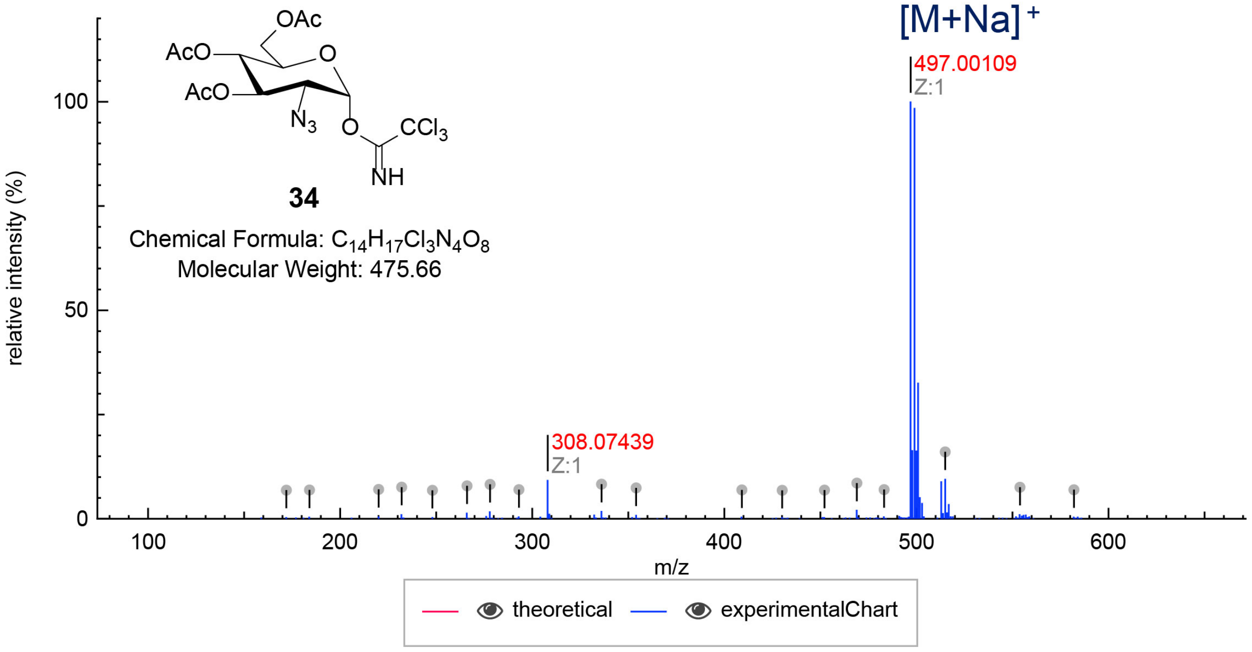

^{1}H NMR of **38** at 400 MHz, $CDCl_3$

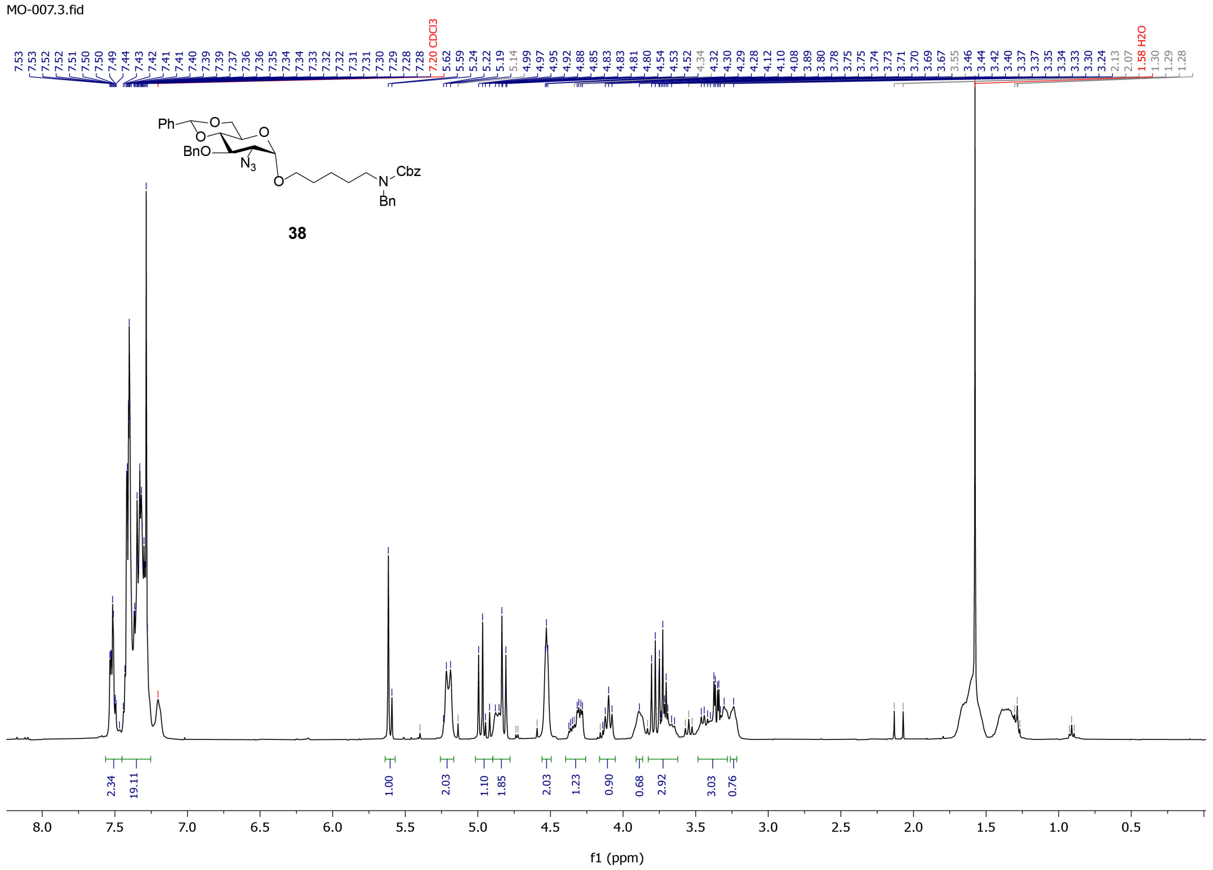

^{13}C NMR of **38** at 101 MHz, $CDCl_3$

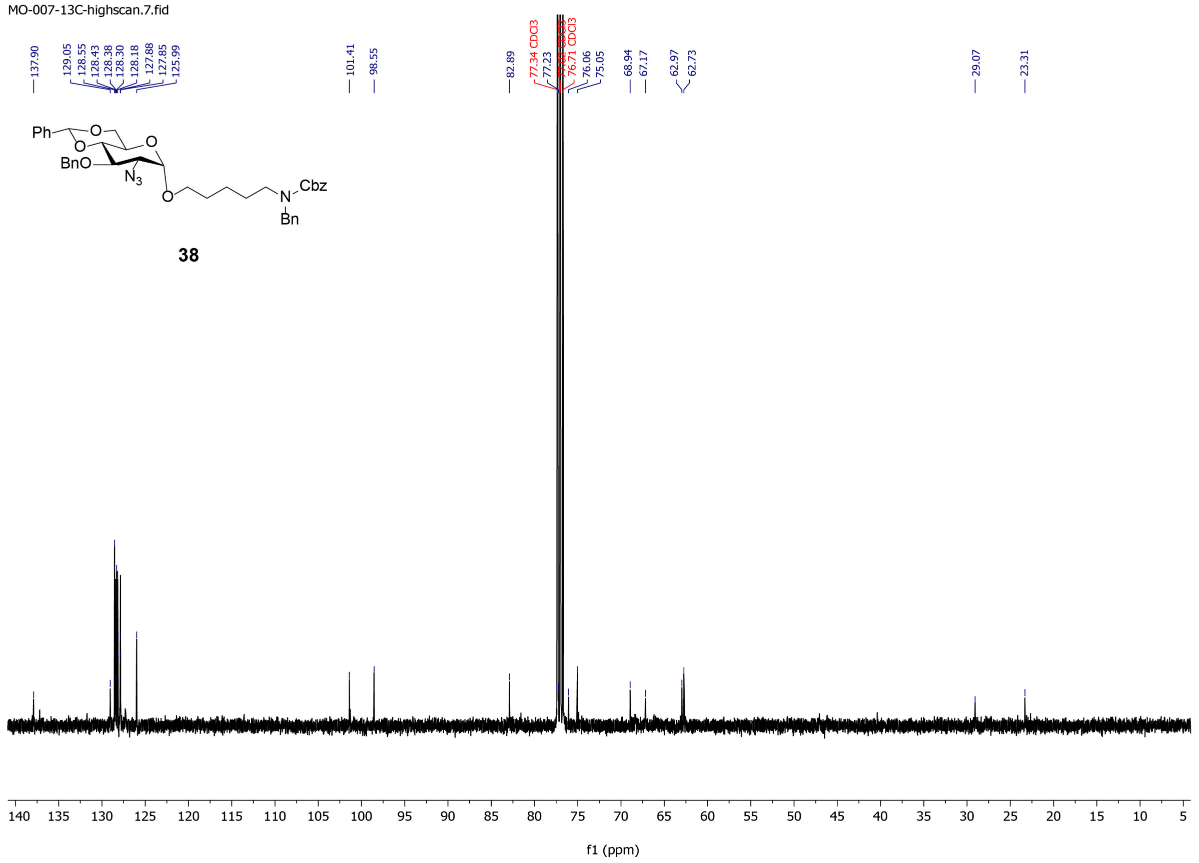

Mass Analysis of **38**

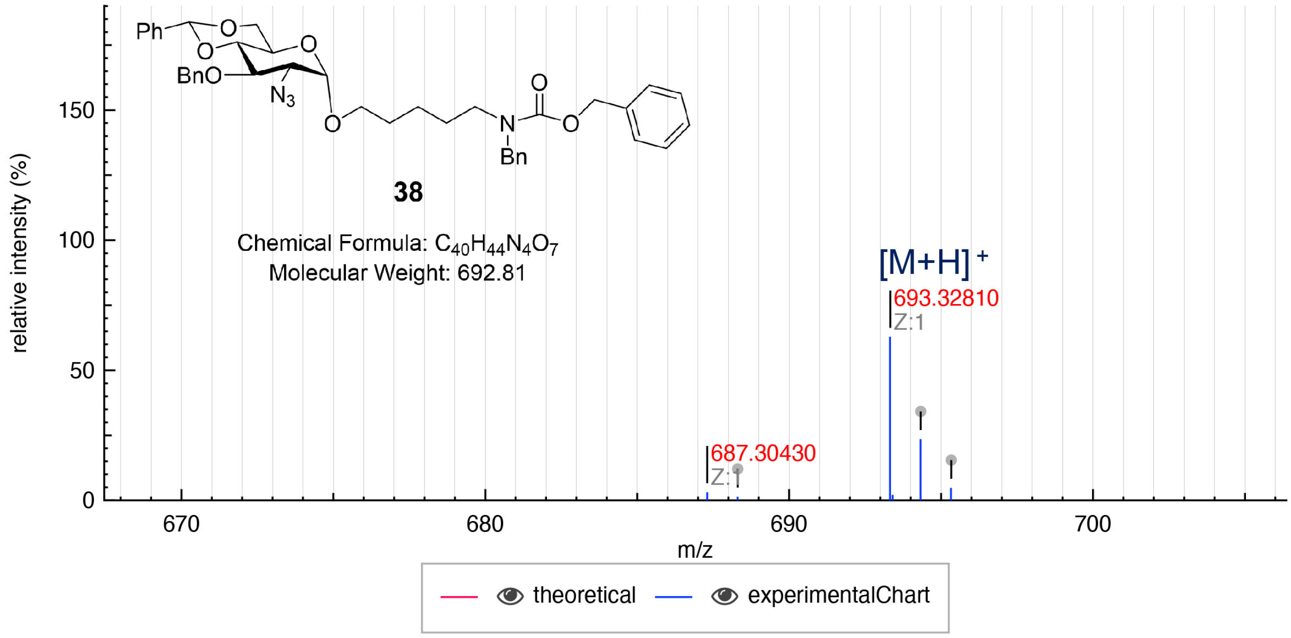

¹H NMR of **41** at 600 MHz, CDCl₃

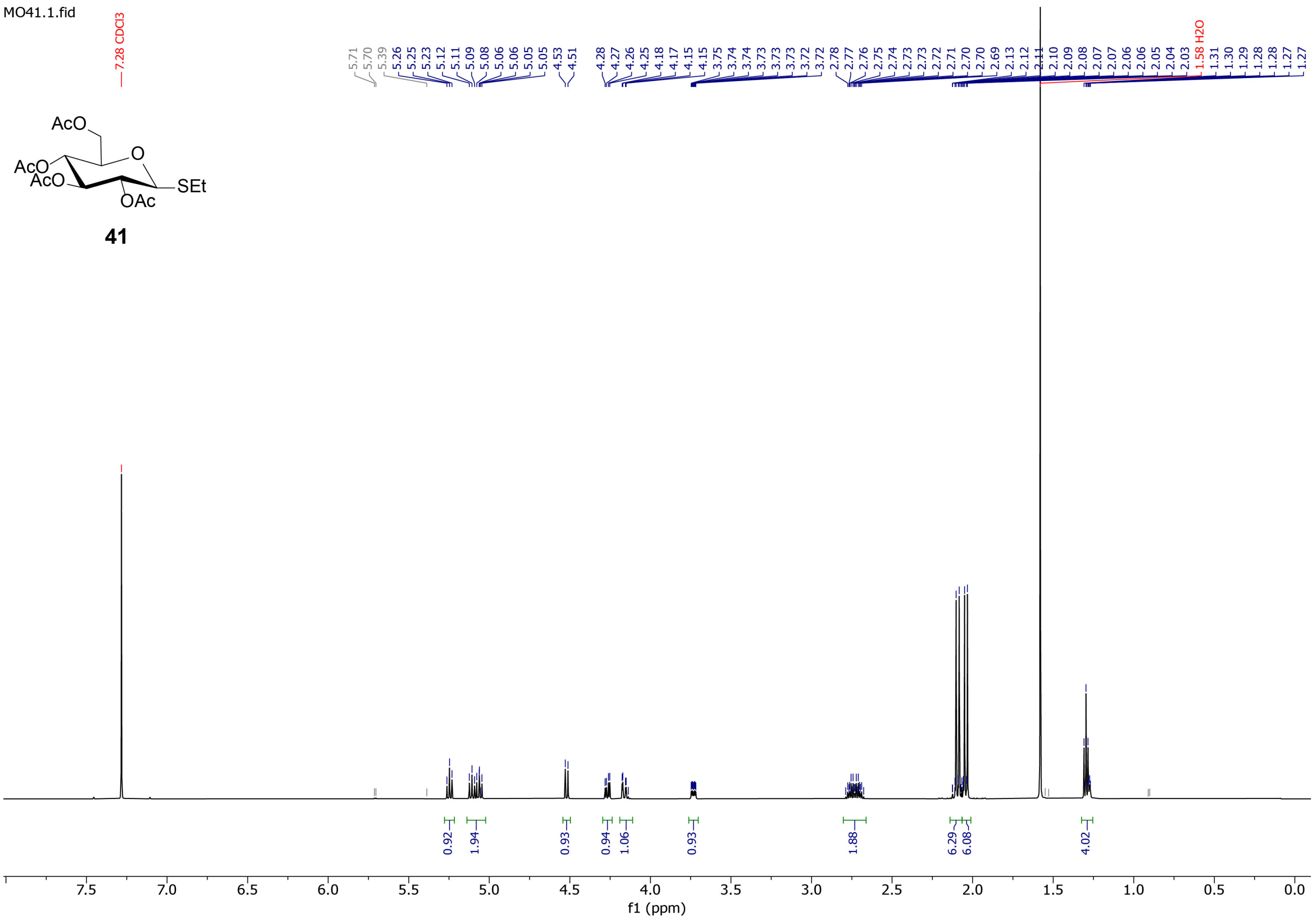

^{13}C NMR of **41** at 151 MHz, $CDCl_3$

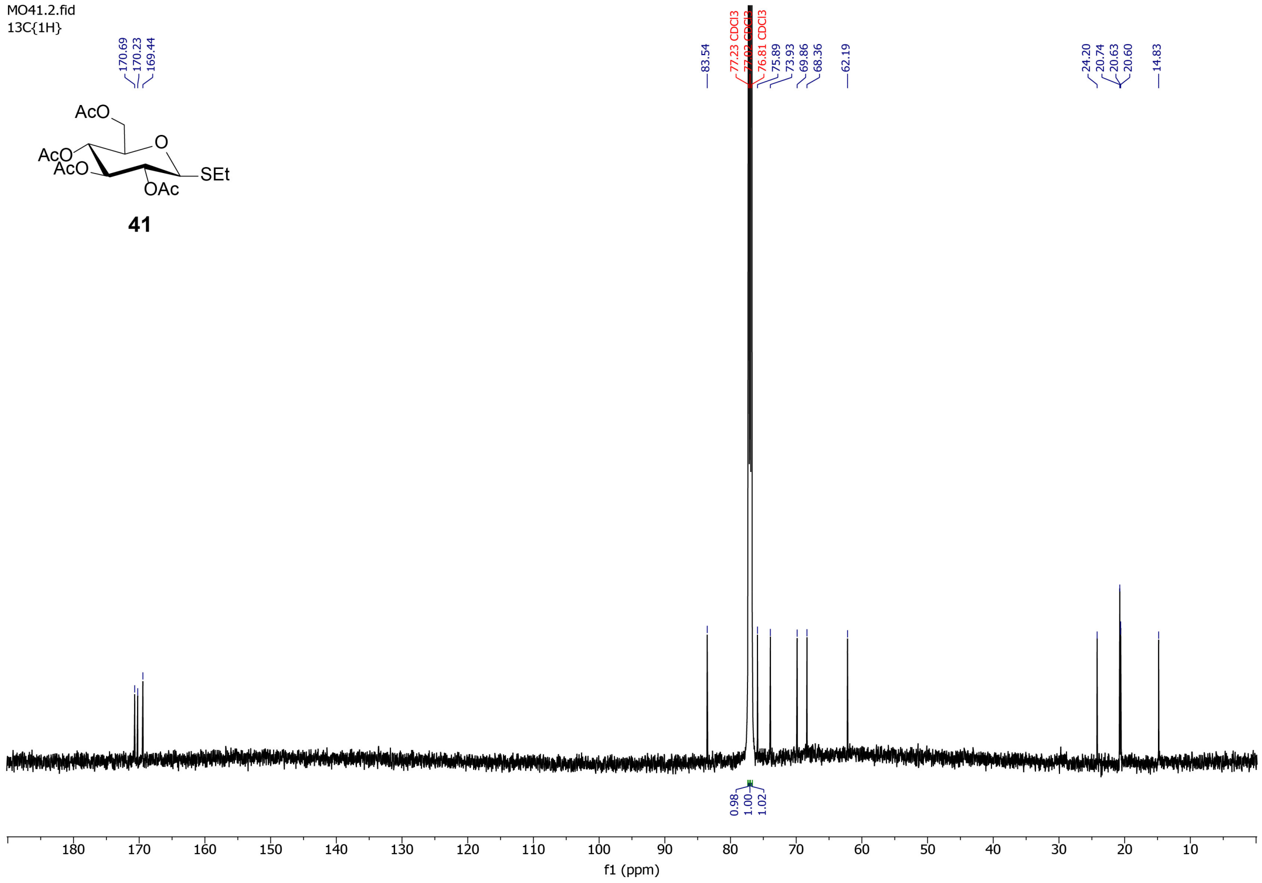

Mass Analysis of **41**

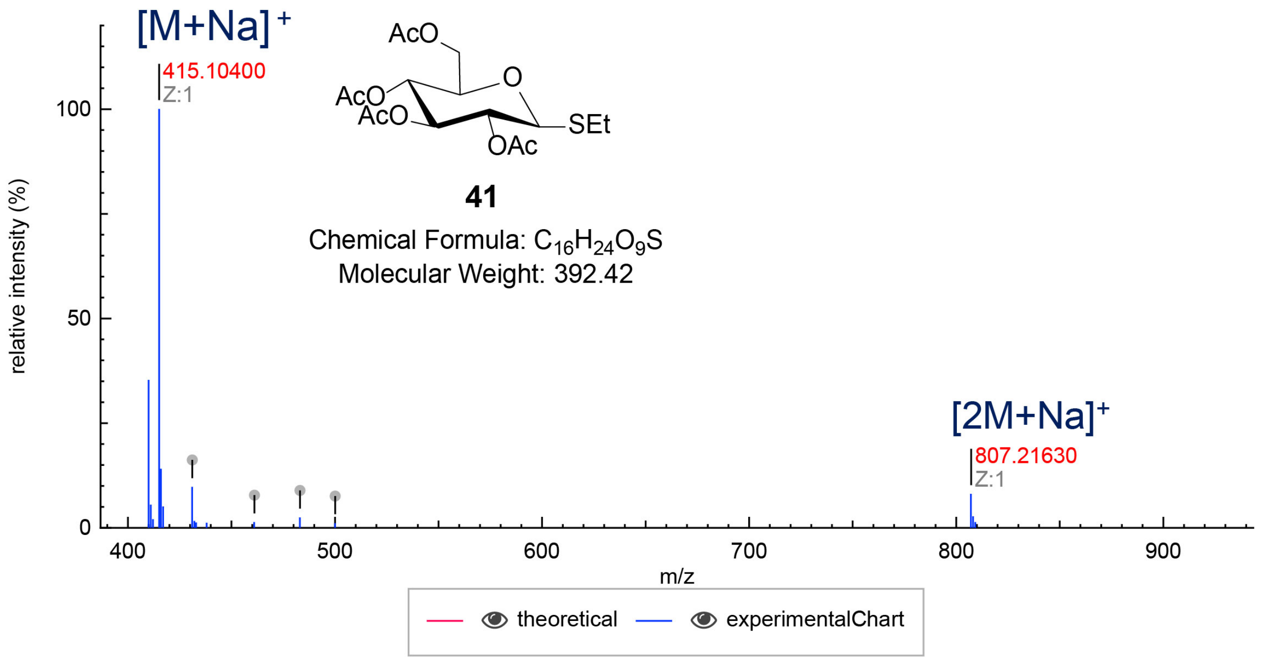

Mass Analysis of **42**

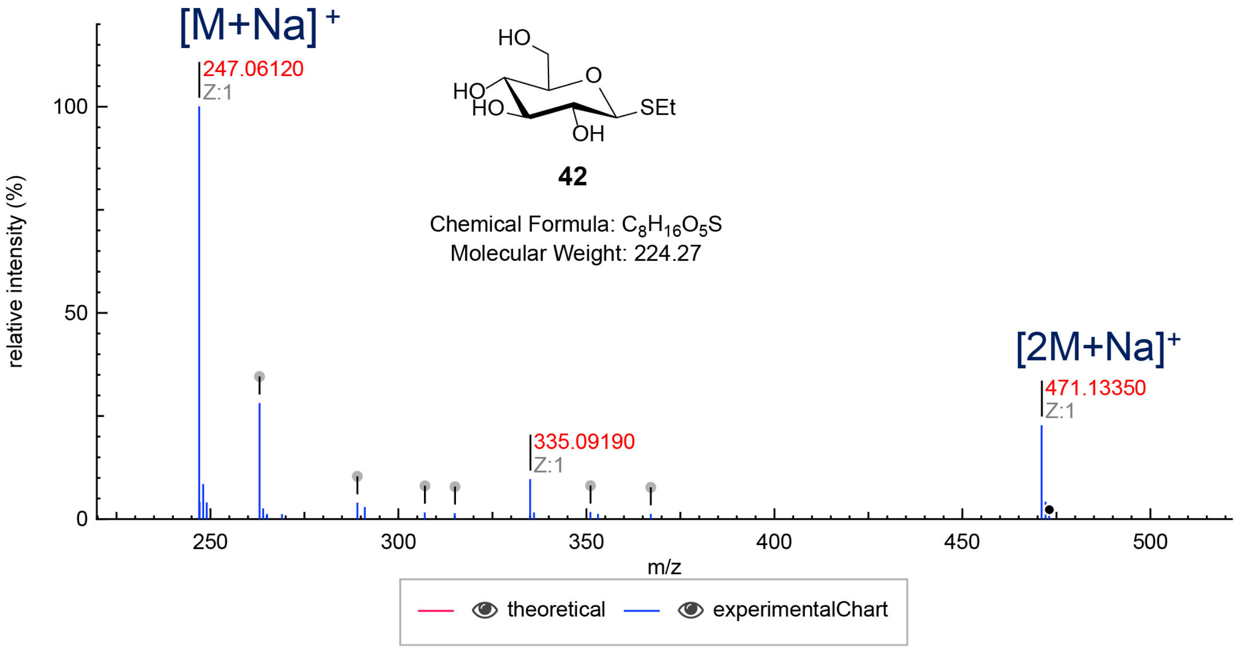

¹H NMR of **43** at 600 MHz, $CDCl_3$

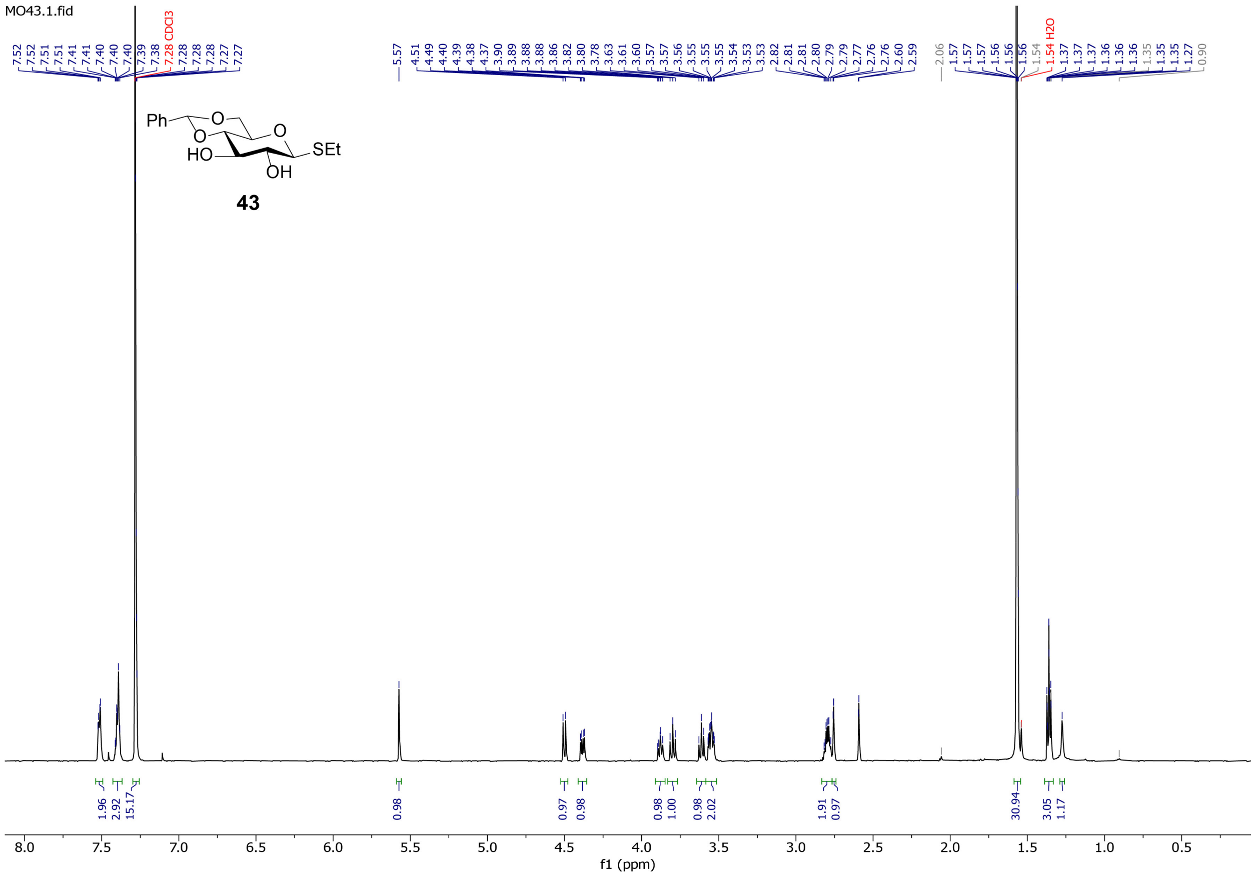

^{13}C NMR of **43** at 151 MHz, $CDCl_3$

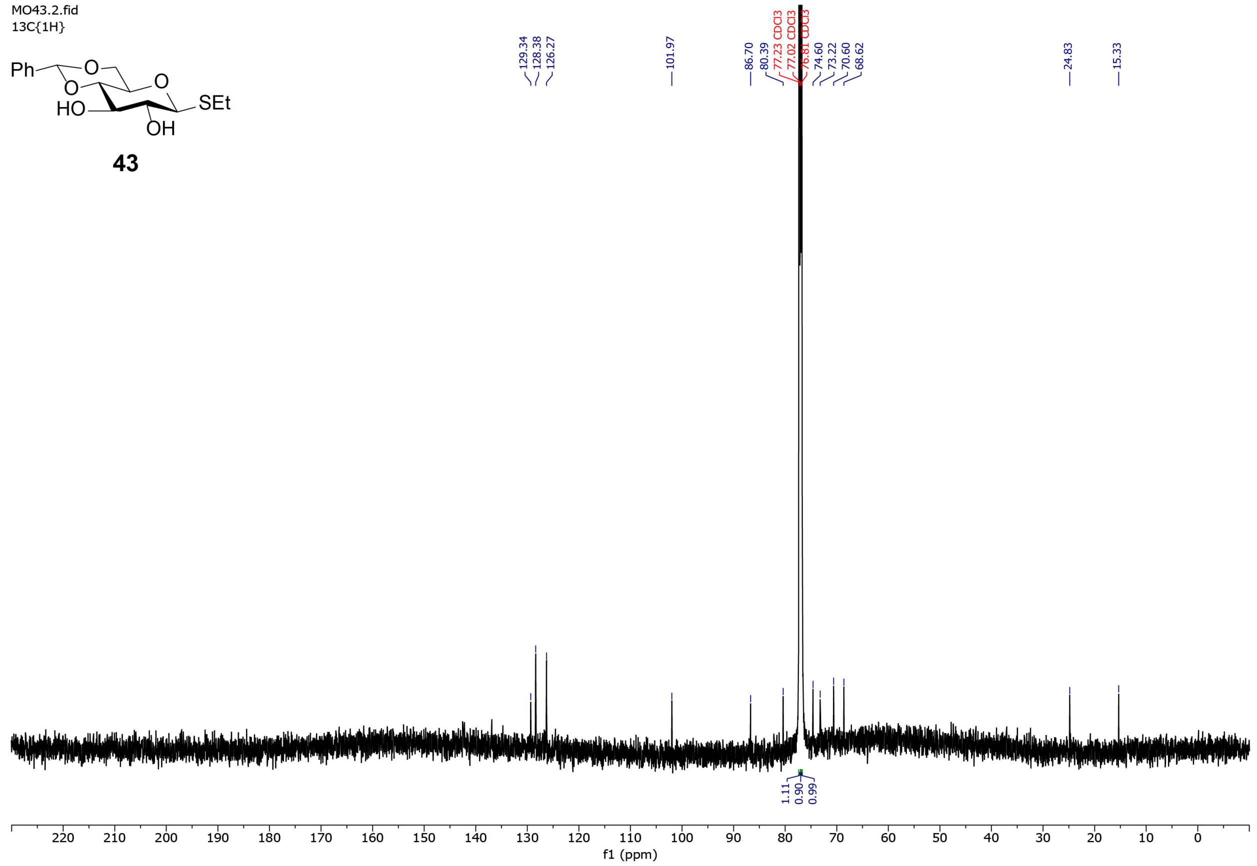

Mass Analysis of **43**

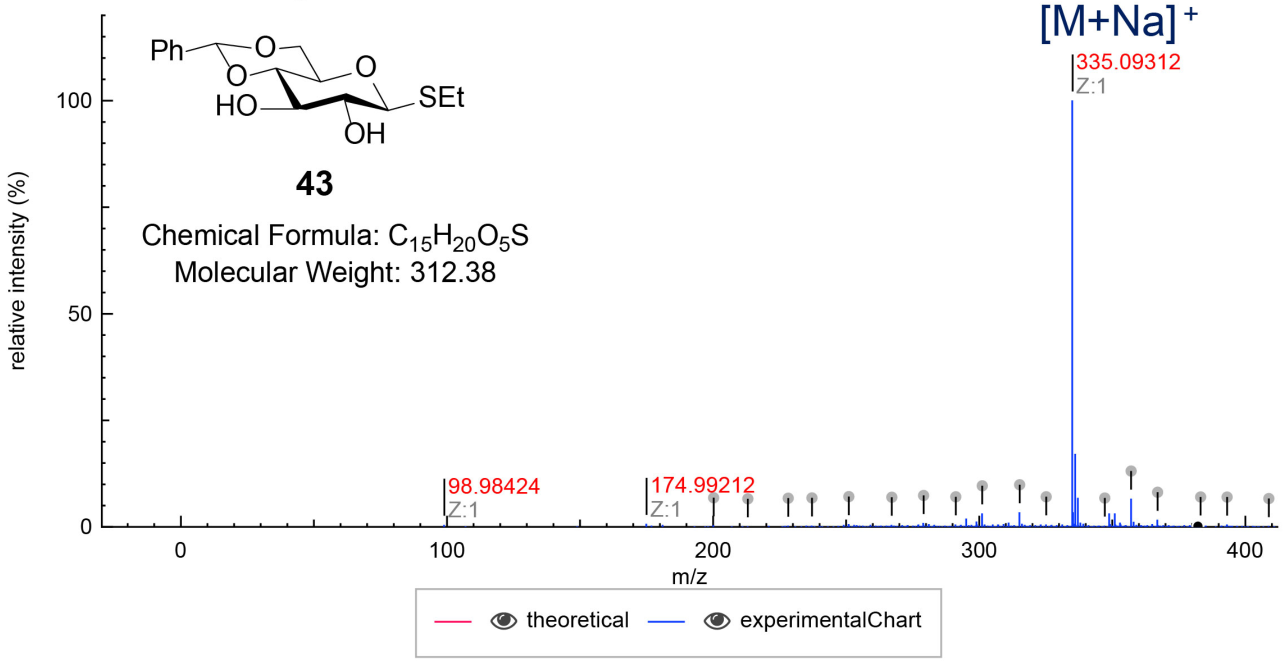

^1H NMR of **44** at 600 MHz, $CDCl_3$

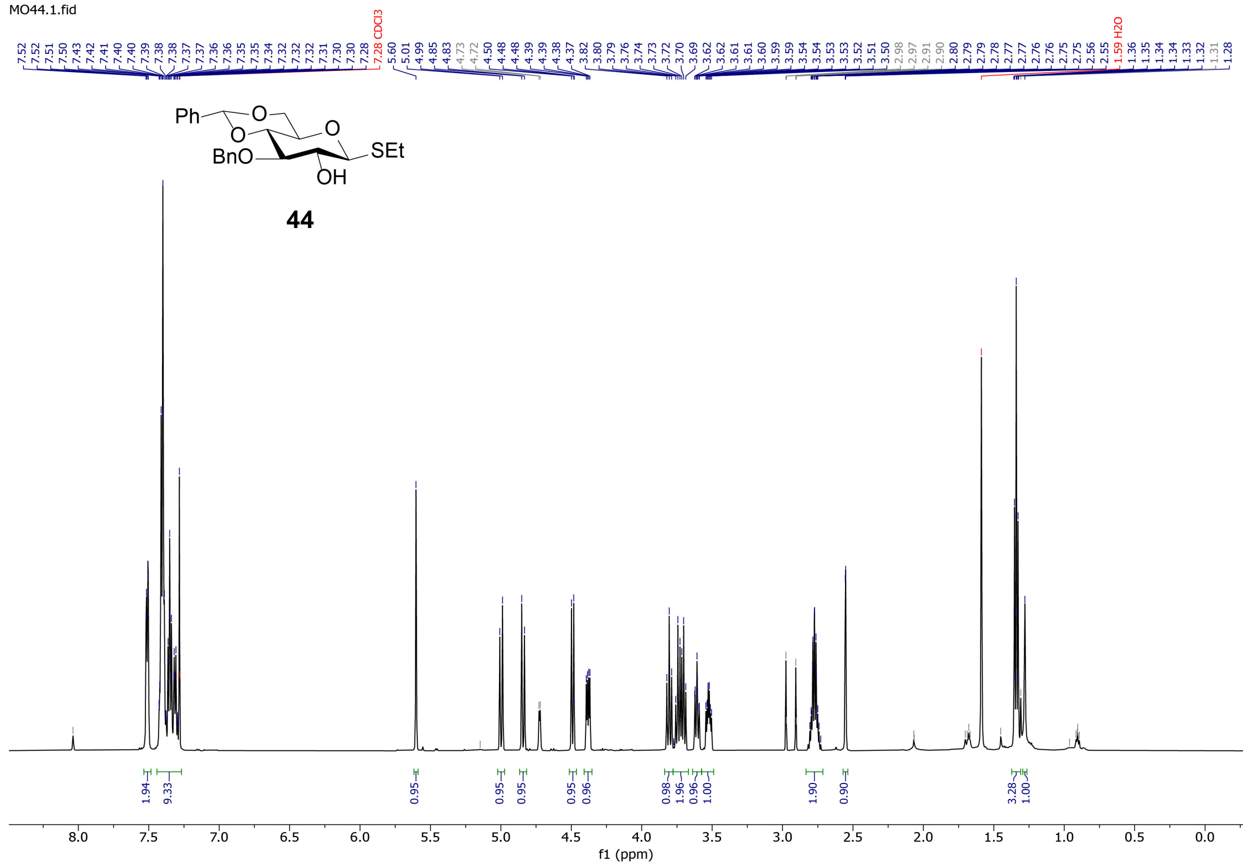

^{13}C NMR of **44** at 151 MHz, $CDCl_3$

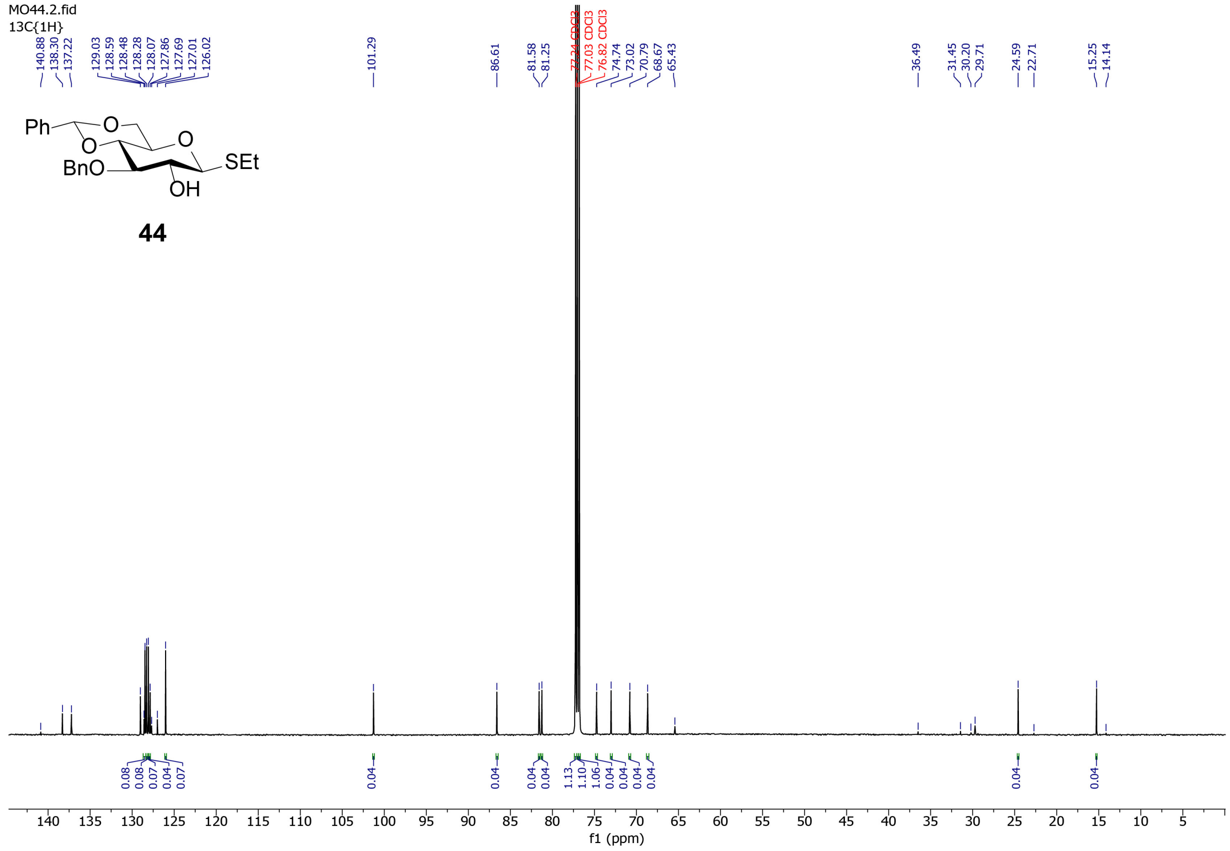

Mass Analysis of **44**

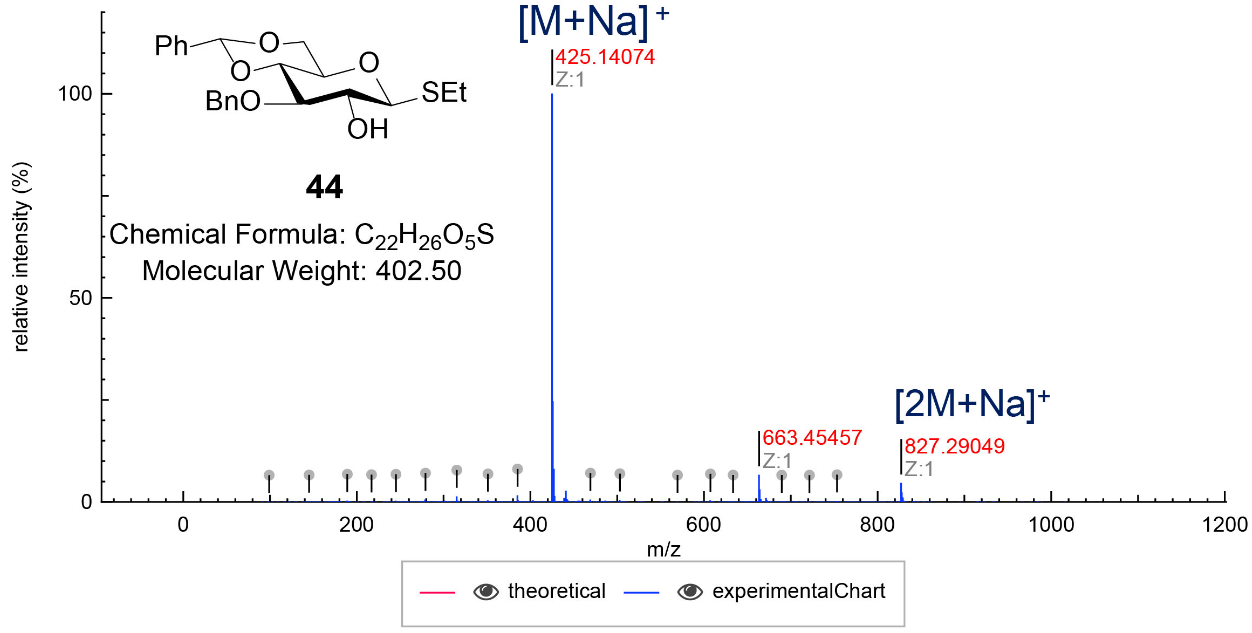

¹H NMR of **45** at 600 MHz, $CDCl_3$

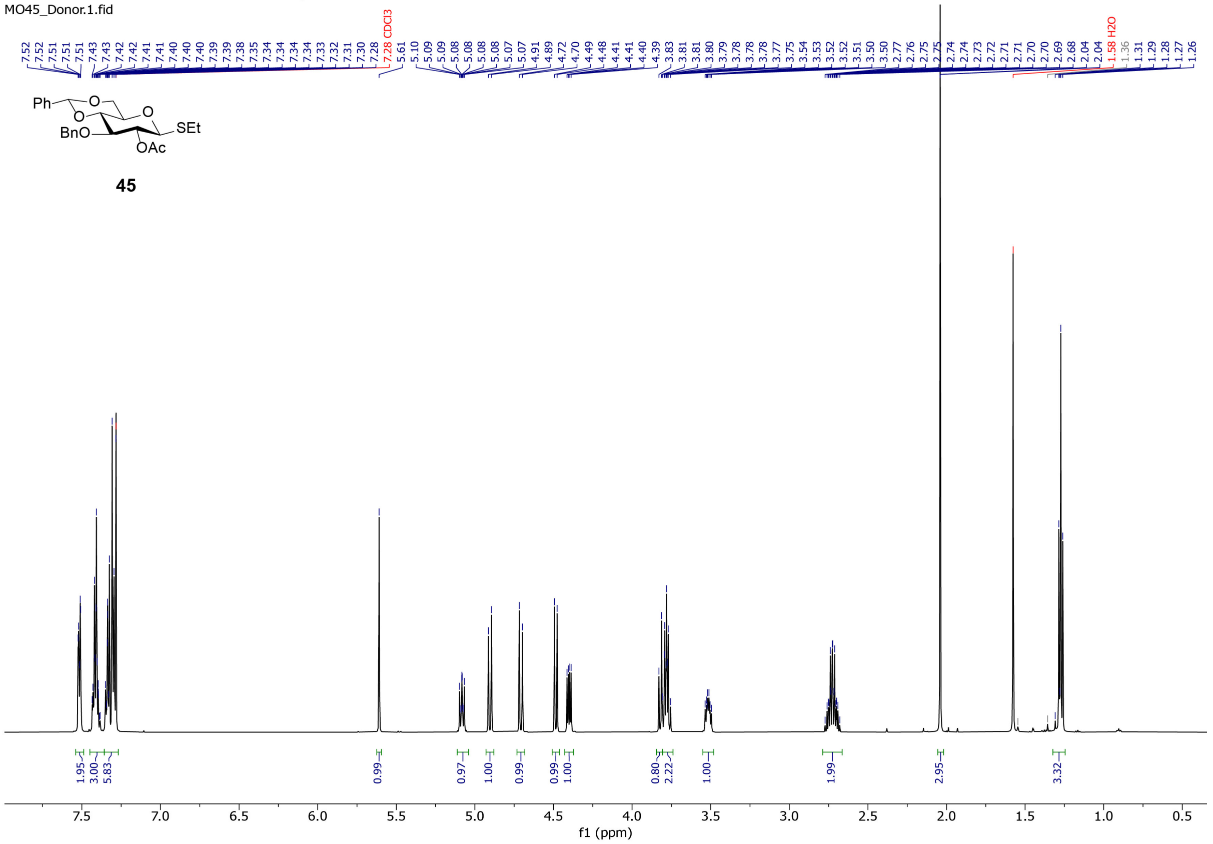

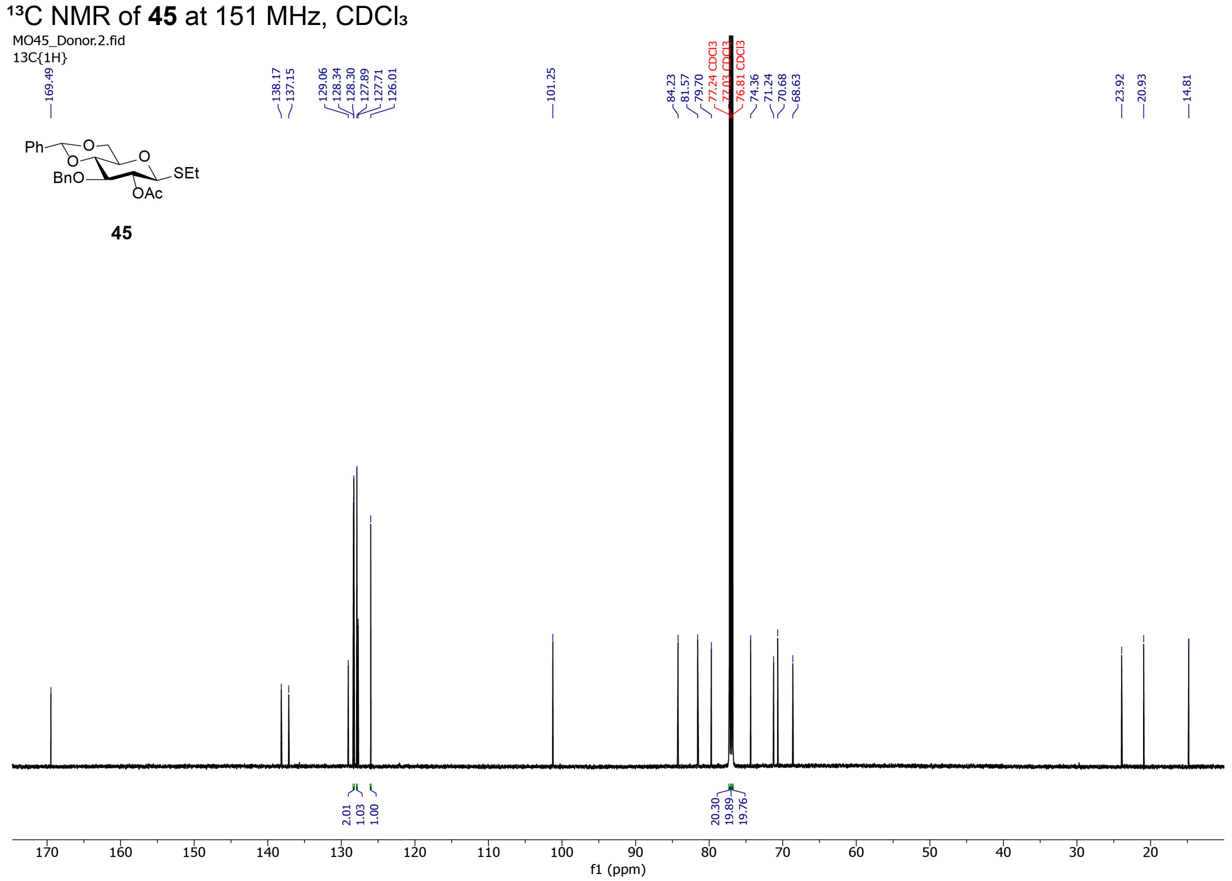

13C NMR of 45 at 151 MHz, CDCl3
MO45_Donor.2.fid
13C{1H}
169.49
138.17
137.15
129.06
128.34
128.30
127.89
127.71
126.01
101.25
84.23
81.57
79.70
77.24 CDCl3
77.03 CDCl3
76.81 CDCl3
74.36
71.24
70.68
68.63
23.92
20.93
14.81
Ph
O
O
O
BnO
SEt
OAc
45
2.01
1.03
1.00
20.30
19.89
19.76
170
160
150
140
130
120
110
100
90
80
70
60
50
40
30
20
f1 (ppm)

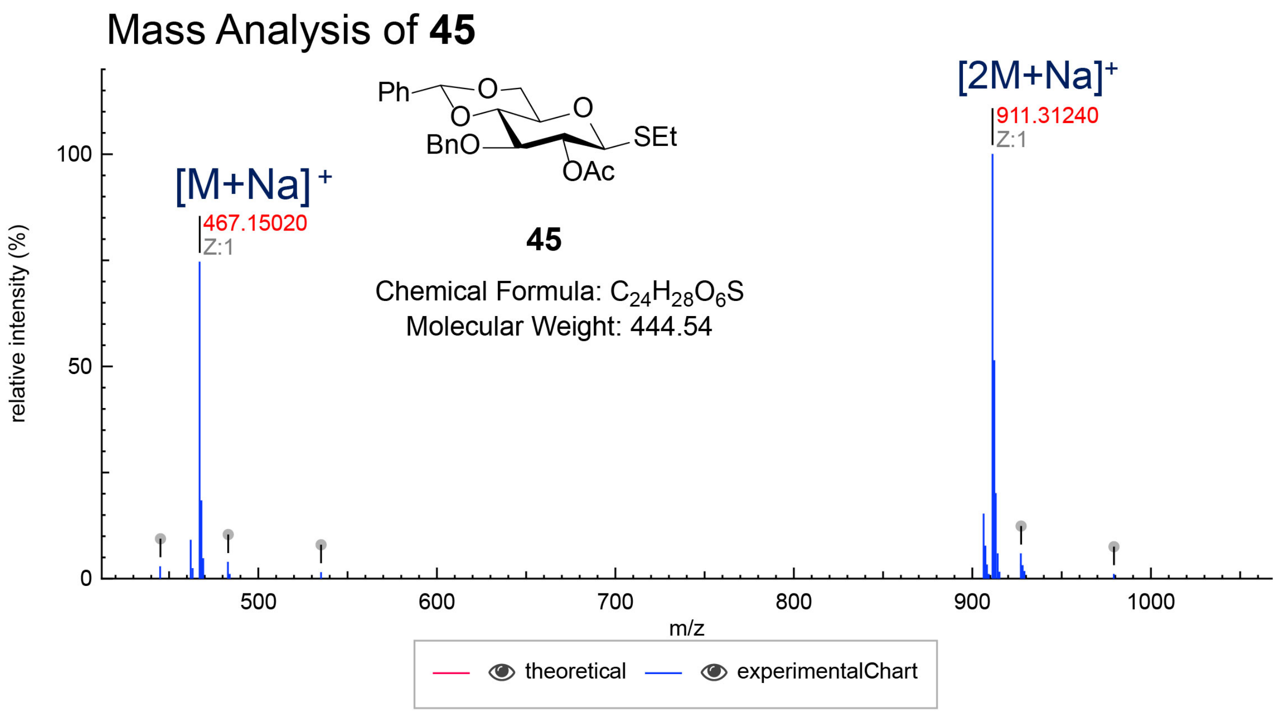

Mass Analysis of 45
Ph
O
O
O
BnO
SEt
OAc
45
Chemical Formula: C24H28O6S
Molecular Weight: 444.54
[M+Na]+
467.15020
Z:1
[2M+Na]+
911.31240
Z:1
relative intensity (%)
100
50
0
500
600
700
800
900
1000
m/z
theoretical
experimentalChart

^{1}H NMR of **46** at 600 MHz, $CDCl_3$

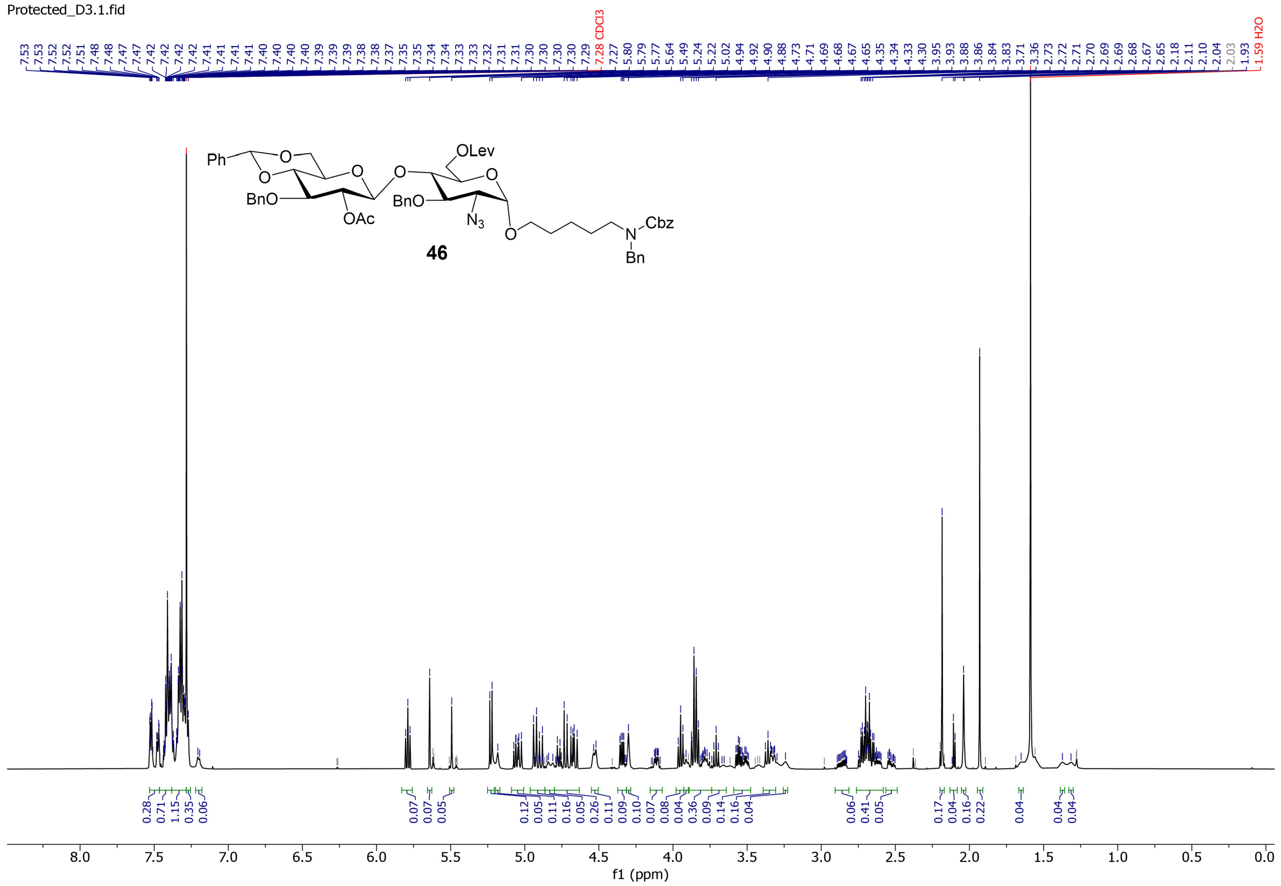

^{13}C NMR of **46** at 151 MHz, $CDCl_3$

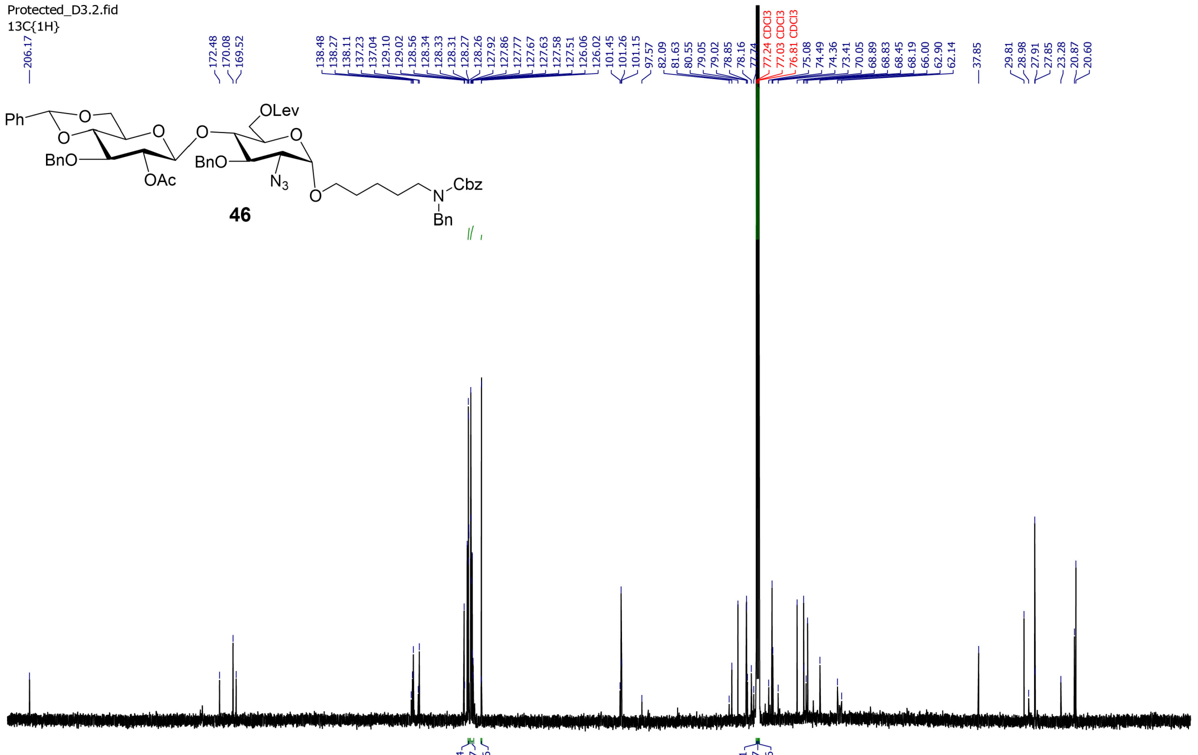

Mass Analysis of **46**

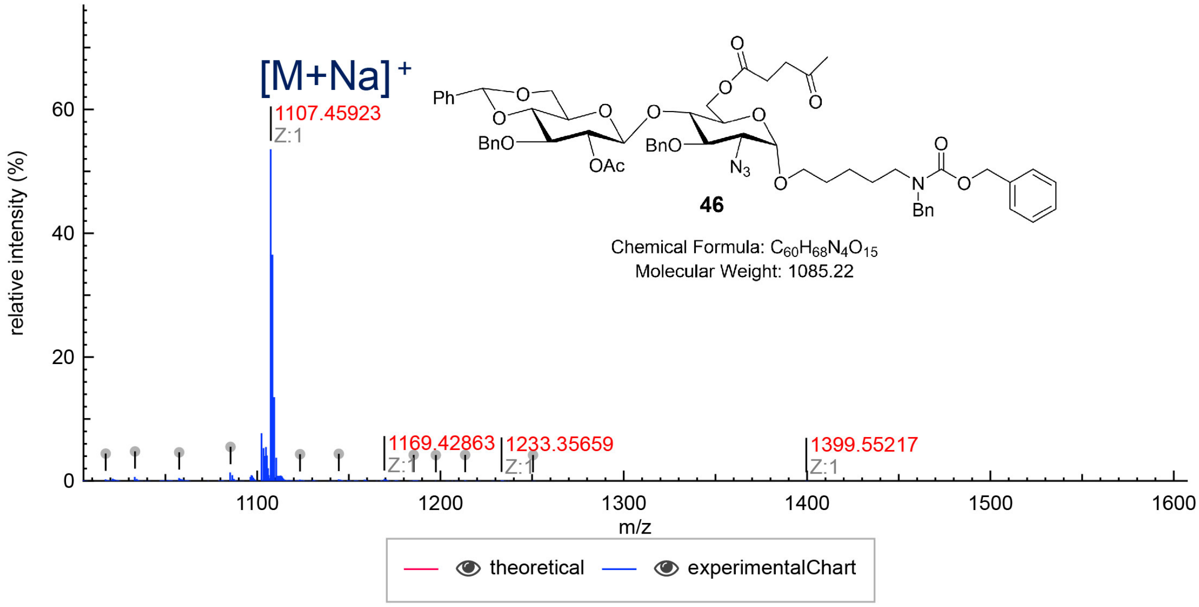

¹H NMR of **D3** at 400 MHz, D_2O

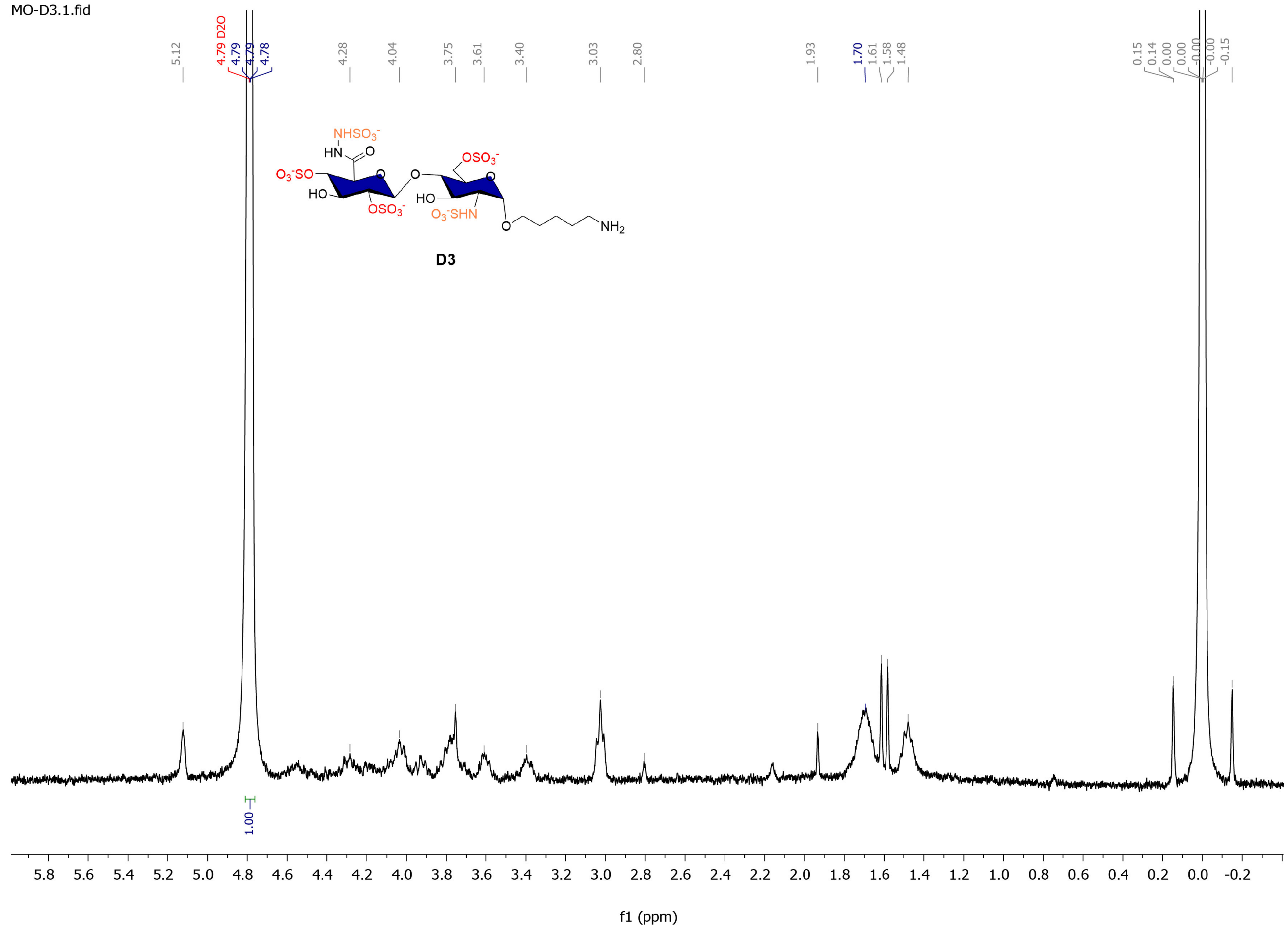

^{1}H NMR of **53** at 400 MHz, N,N-Dimethylformamide-d7

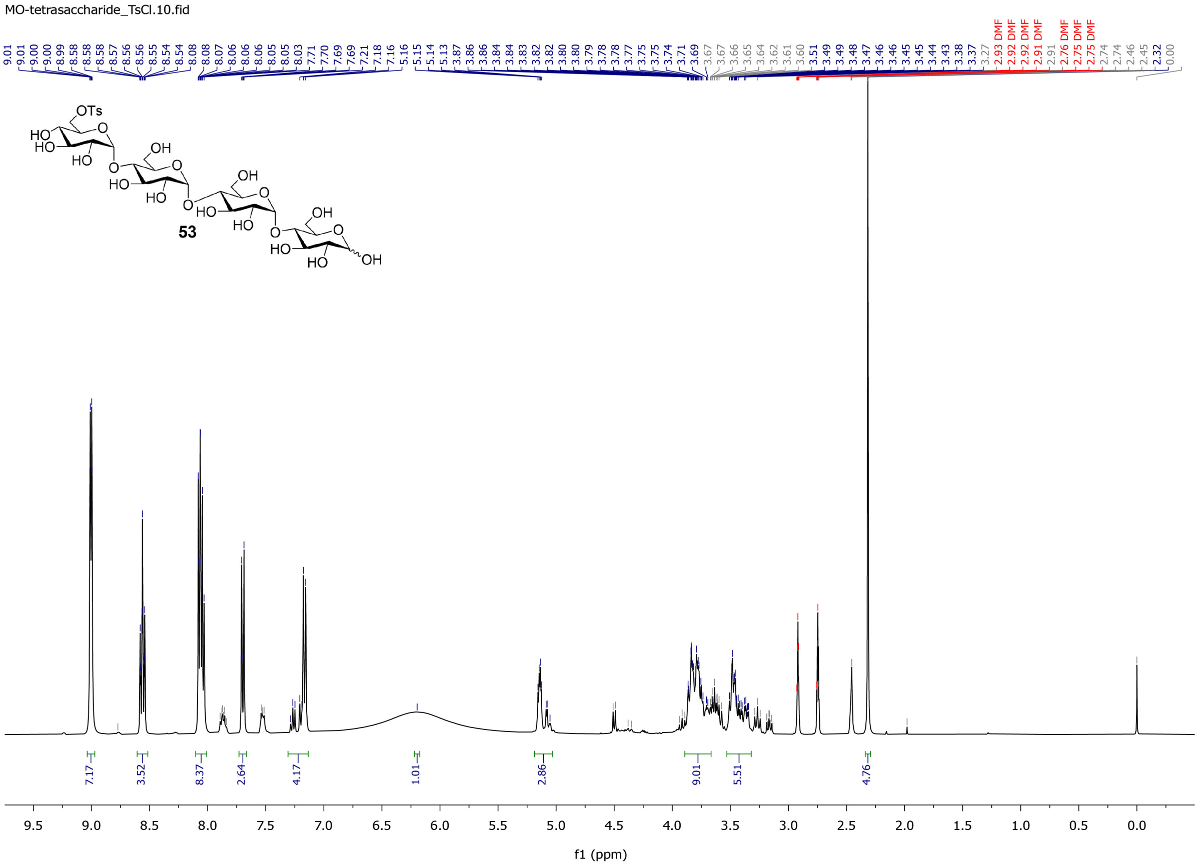

Mass Analysis of **53**

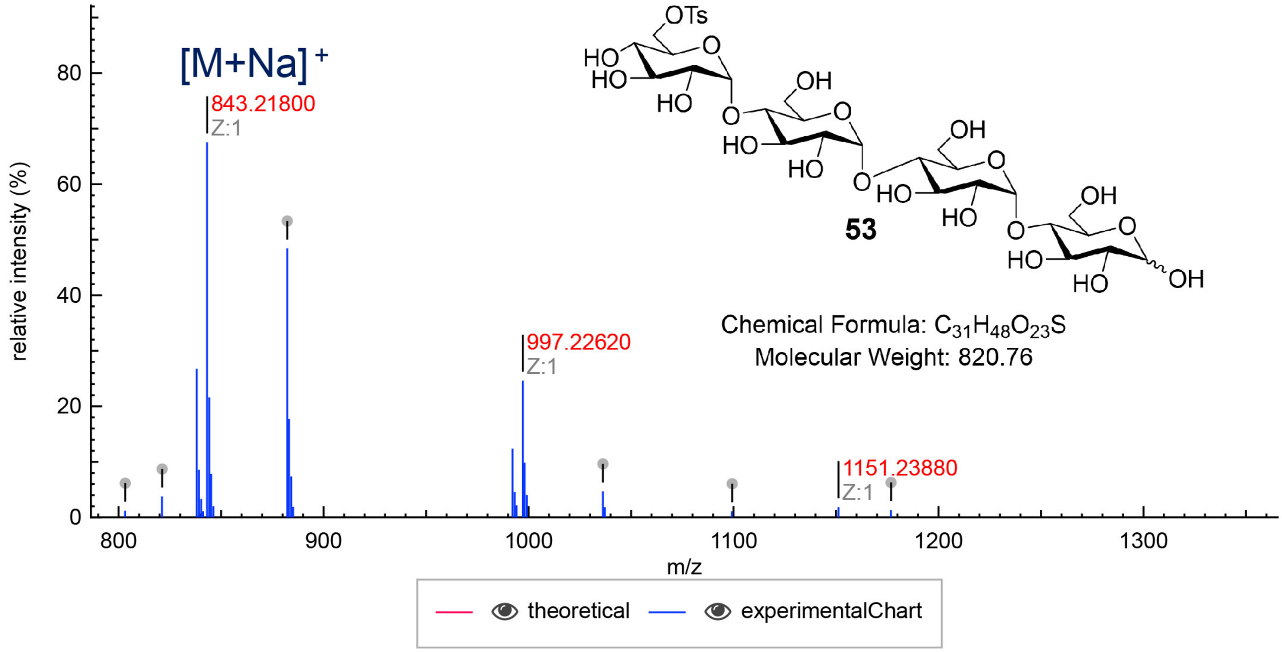

^{1}H NMR of **54** at 400 MHz, D_2O

MO_tetrasaccharide_N3.4.fid

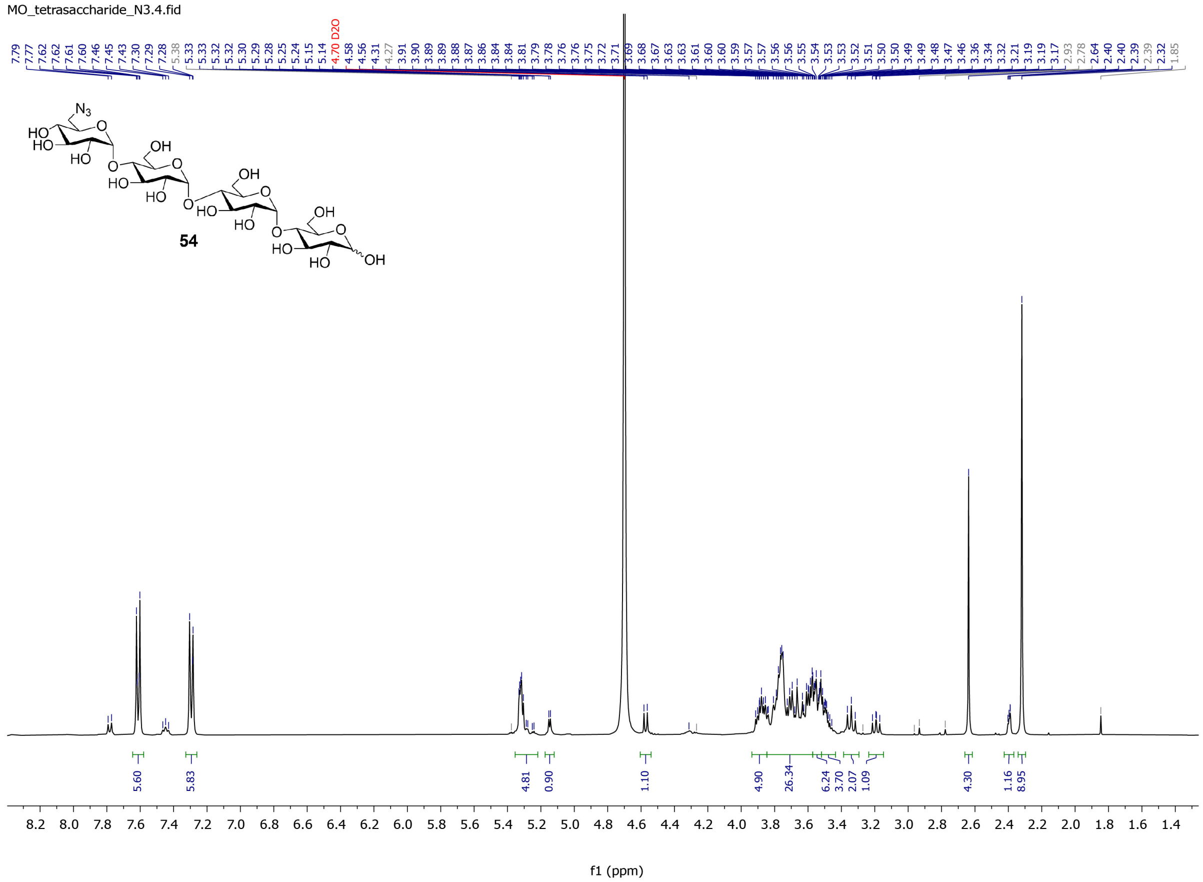

^{1}H NMR of **54** at 101 MHz, D_2O

MO_tetrasaccharide_N3-13C.6.fid

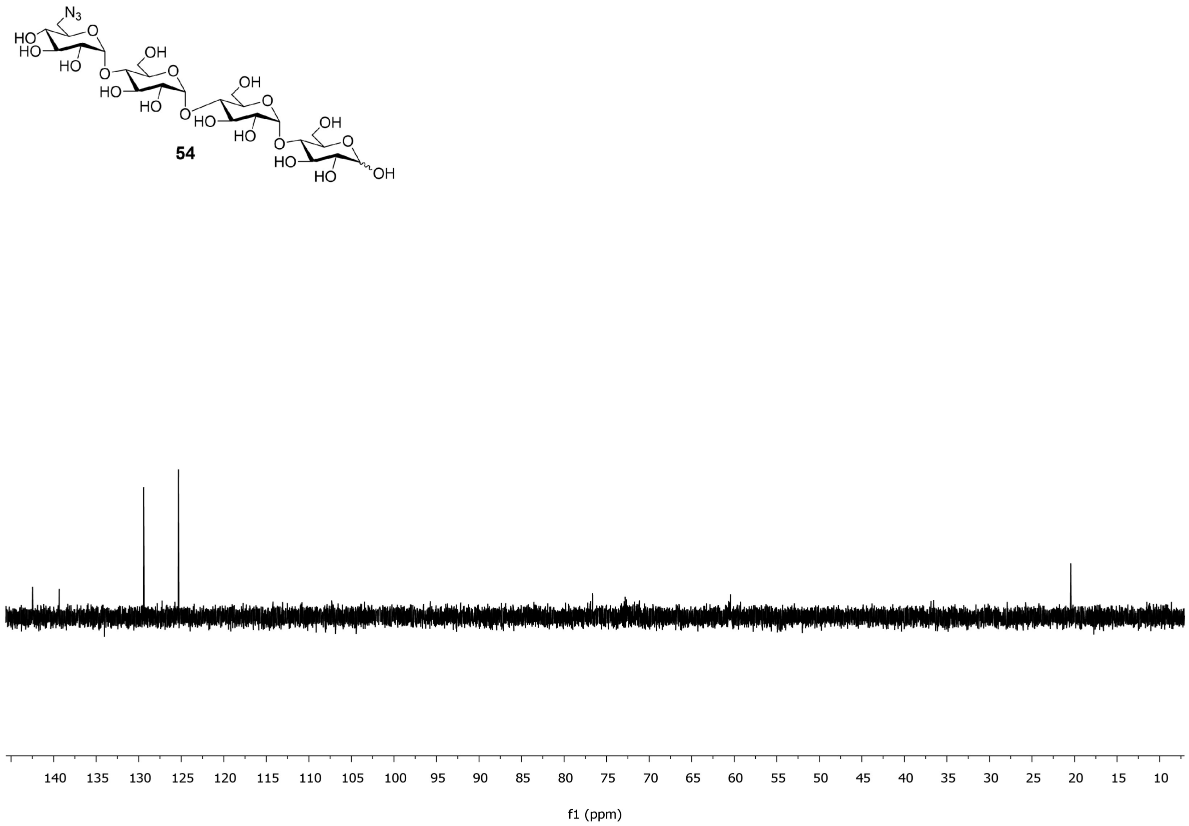

^{1}H NMR of **T1** at 400 MHz, D_2O

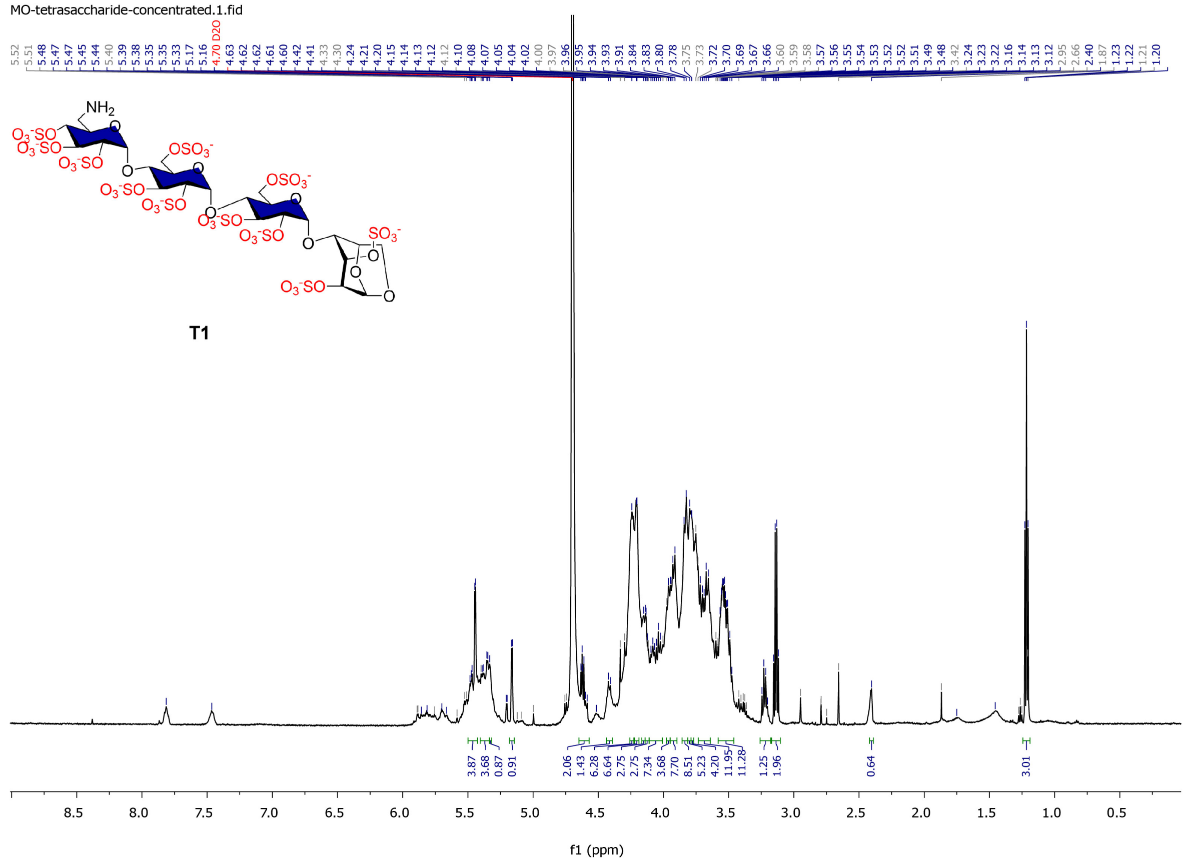

^{13}C NMR of **T1** at 101 MHz, D_2O

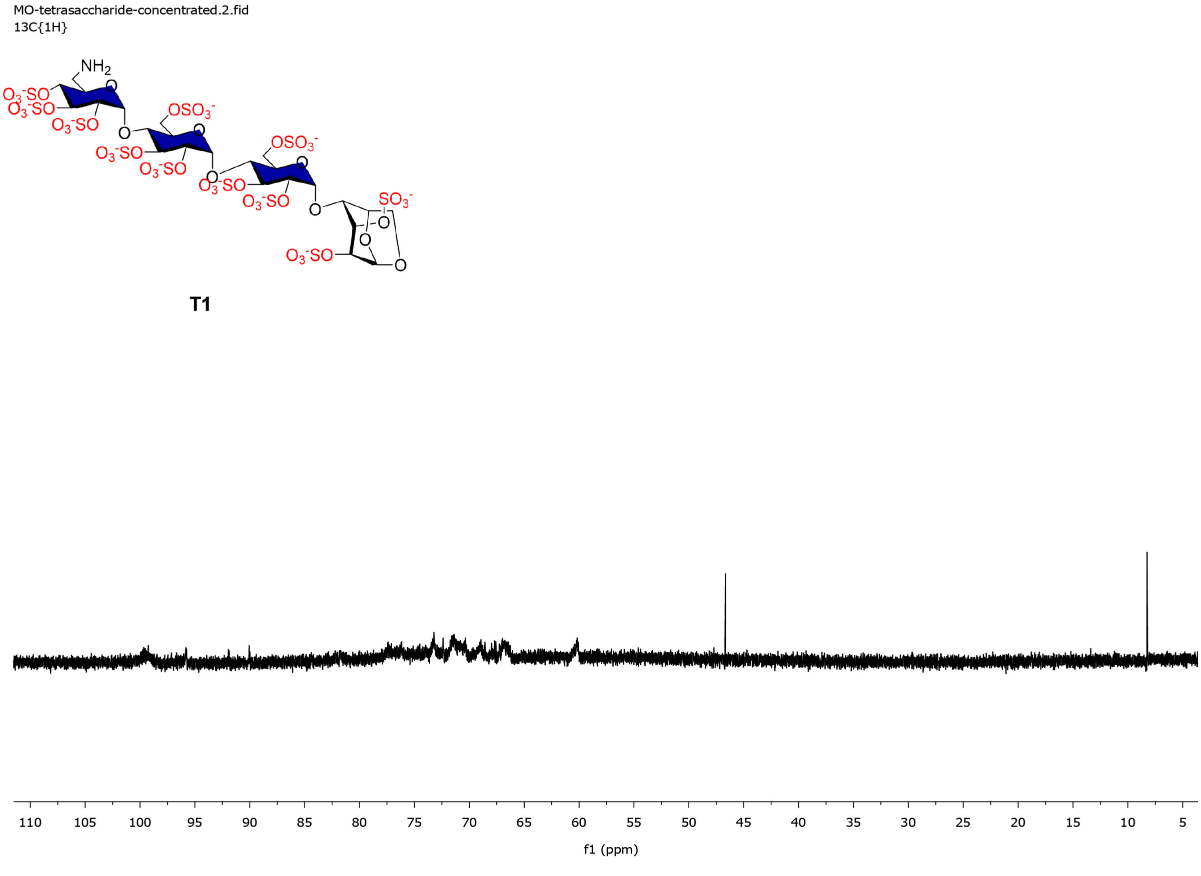

^{1}H-^{1}H COSY NMR of **T1** at 400 MHz, D_2O

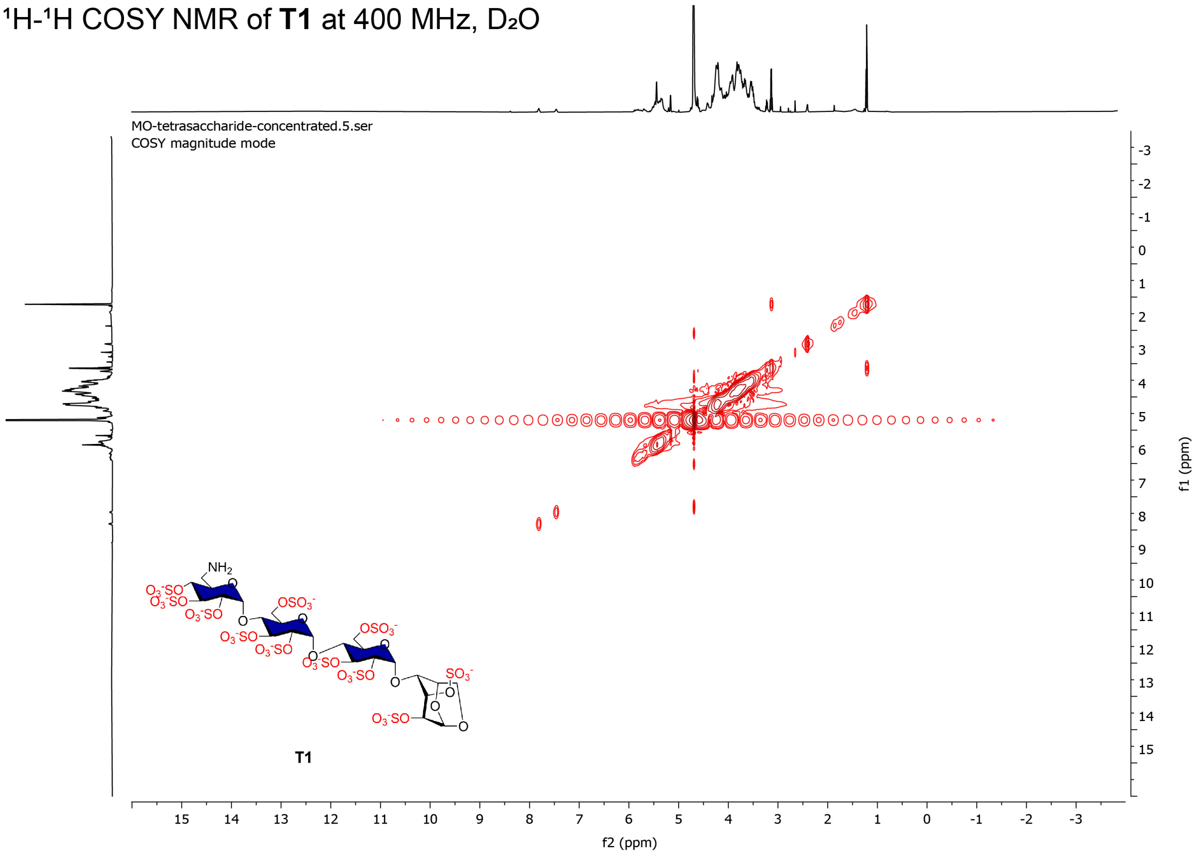

^{1}H-^{13}C HSQC NMR of **T1**, D_2O

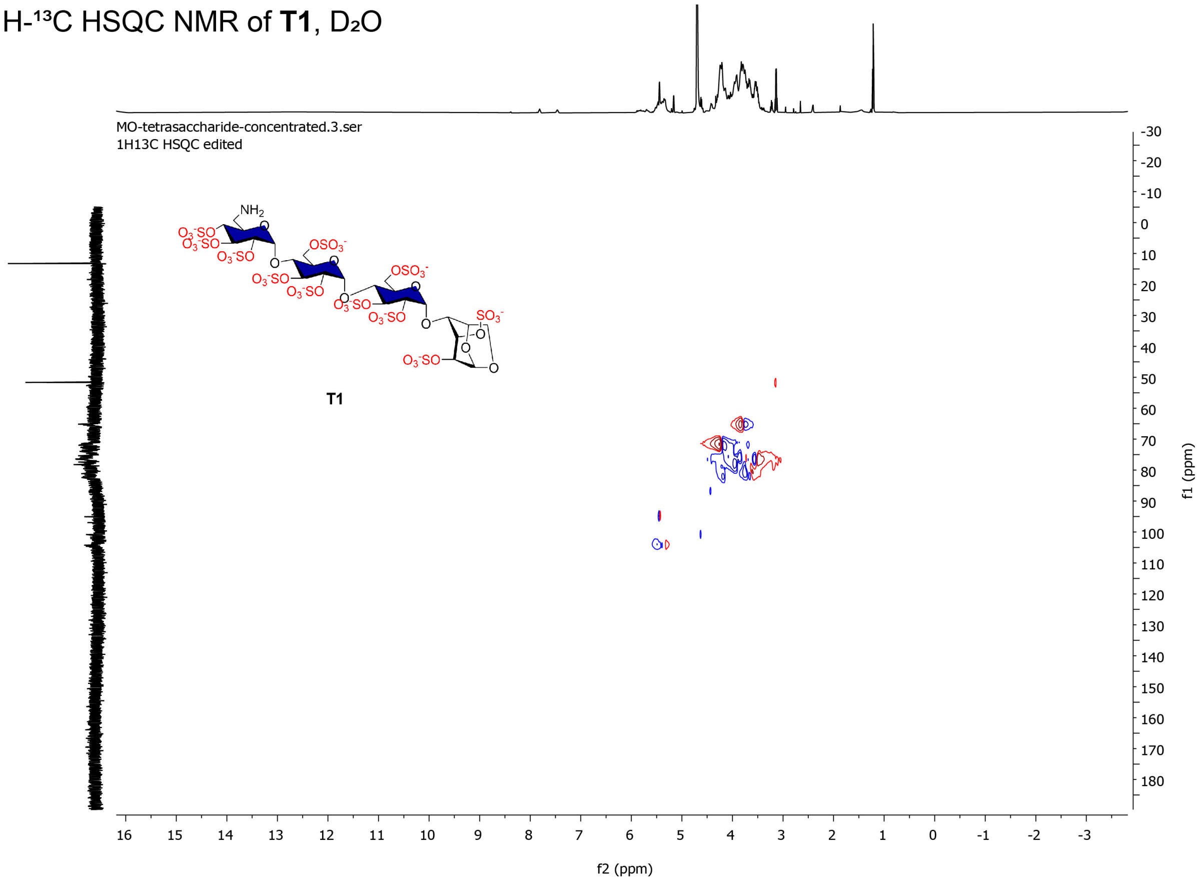

^{1}H-^{13}C HMBC NMR of **T1**, D_2O

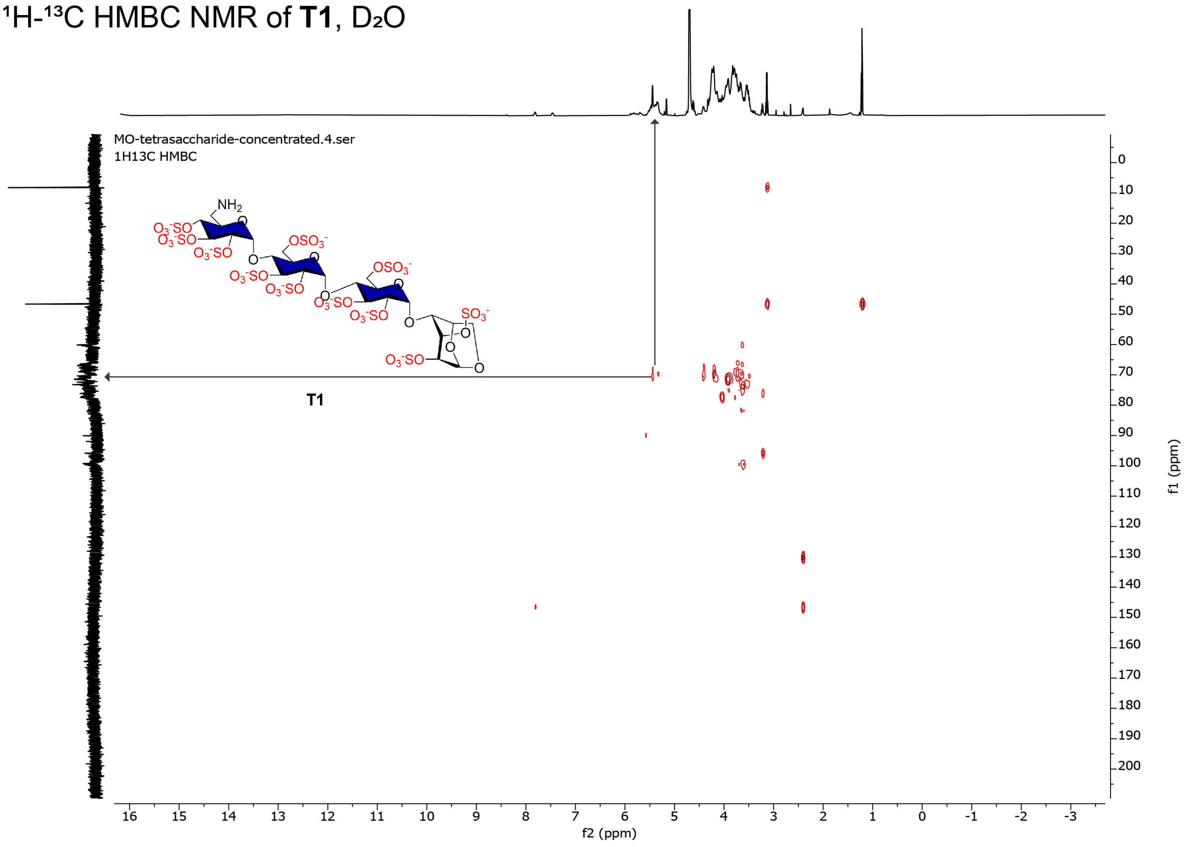

Supplement: Supplementary file 1 [file ja5c13142_si_001.pdf]
